# Photoinduced Intermolecular Radical Hydroalkylation of Olefins via Ligated Boryl Radicals-Mediated Halogen Atom Transfer

**DOI:** 10.1021/acs.orglett.4c02034

**Published:** 2024-07-01

**Authors:** Ting Wan, Łukasz W. Ciszewski, Davide Ravelli, Luca Capaldo

**Affiliations:** † Flow Chemistry Group, van’t Hoff Institute for Molecular Sciences (HIMS), University of Amsterdam, 1098 XH Amsterdam, The Netherlands; ‡ The Research Center of Chiral Drugs, Innovation Research Institute of Traditional Chinese Medicine, Shanghai University of Traditional Chinese Medicine, Shanghai 201203, China; § PhotoGreen Lab, Department of Chemistry, University of Pavia, 27100 Pavia, Italy; ∥ SynCat Lab, Department of Chemistry, Life Sciences and Environmental Sustainability, University of Parma, 43124 Parma, Italy

## Abstract

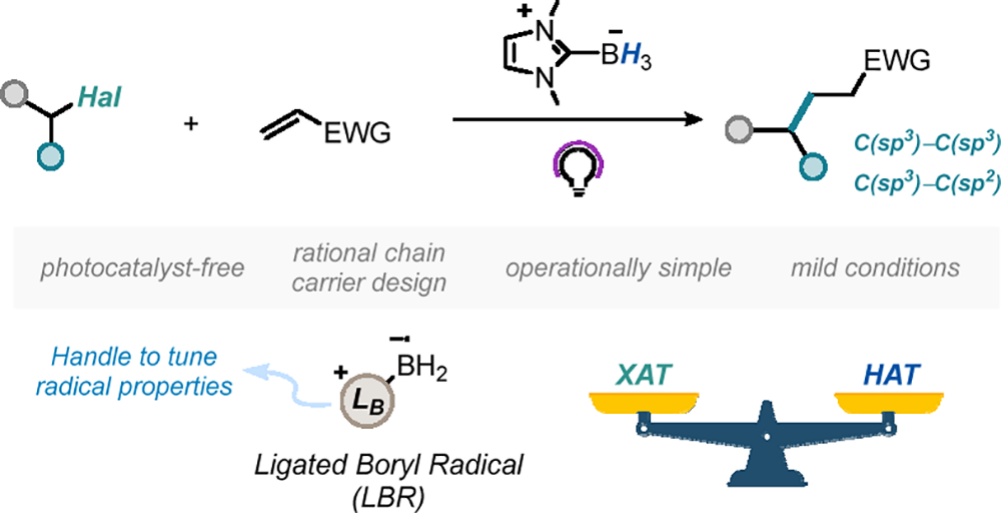

Light-mediated Halogen-Atom Transfer (XAT) has become a significant methodology in contemporary synthesis. Unlike α-aminoalkyl and silyl radicals, ligated boryl radicals (LBRs) have not been extensively explored as halogen atom abstractors. In this study, we introduce NHC-ligated boranes as optimal radical chain carriers for the intermolecular reductive radical hydroalkylation and hydroarylation of electron-deficient olefins by using direct UV-A light irradiation. DFT analysis allowed us to rationalize the critical role of the NHC ligand in facilitating efficient chain propagation.

Halogen-Atom Transfer (XAT) stands as a versatile and programmable approach to generate carbon-centered radicals.^1^ Historically, Menapace first showed that stannyl radicals are very efficient halogen abstractors because of both favorable thermodynamic and kinetic factors, which marked the dawn of an incredibly rich chemistry via radical manifolds.^2^ However, concerns about the toxicity of organotin compounds and a recent emphasis on Green Chemistry have led to an ever-increasing interest in alternatives, preparing the ground for a transition from the Tyranny of Tin^3^ to the Democracy of Photons.^4^ In fact, visible-light photocatalysis has emerged as a milder technique for carbon-centered radical generation via XAT by silyl,^5^ α-aminoalkyl,^6^ and (ligated) boryl radicals.^7^ Alternative strategies for the generation of halogen abstractors have been devised as well, including an electrochemical manifold.^8^

Intriguingly, the observation that some XAT-based strategies may proceed through radical chain propagation^7c,9^ stimulated the emergence of photocatalyst-free manifolds for XAT. One notable example in this regard is the reductive radical hydroalkylation of olefins (RRH), also known as Giese reaction. Seminal works from the groups of Giese,^10^ Renaud,^11^ and Zard^12^ showed that a complex choreography of elementary steps is needed to achieve such transformations efficiently (Scheme 1A).^13^ Inspired by the work of Chatgilialoglu,^14^ Gaunt documented the RRH of olefins with alkyl iodides using tris(trimethylsilyl)silane (TTMS, (Me_3_Si)_3_SiH) as a chain carrier (Scheme 1B).^15^ More recently, Noël extended this approach to alkyl bromides by adopting the flow technology.^16^ In these cases, efficient chain propagation was obtained by tuning the stoichiometry of the starting materials and the chain carrier or by increasing the reaction temperature.

**Scheme 1:**
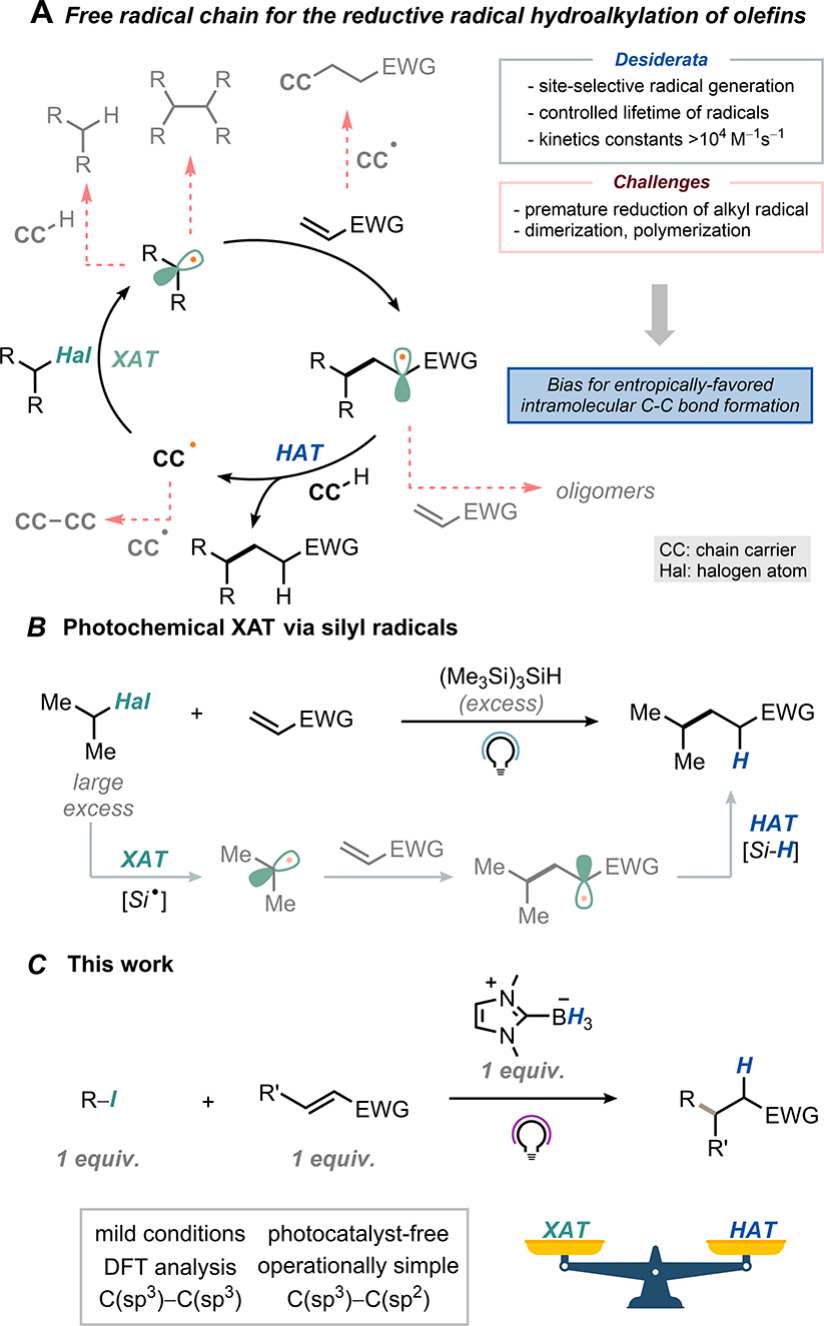
(A) General Features of Intermolecular RRH Reactions via Radical Chain; (B) TTMS as a Radical Chain Carrier for the Photochemical RRH of Olefins; (C) This Work

In a seminal work, Roberts proposed ligated boryl radicals (LBRs; L_B_-BR_2_^•^, L_B_: Lewis base)^17^ as chain carriers for radical hydroalkylation via XAT in a thermal domain.^18^ Therein, the authors noted that L_B_ was crucial to propagate the chain: the reaction was impeded when amine-ligated boranes were used, whereas it proceeded with phosphine-ligated boranes. Unfortunately, due to the harsh conditions (heating, peroxides) required for LBR generation, yields were low, and the approach was not generally applicable. More recently, Ryu proposed a photochemical variant capitalizing on borohydrides.^19^ In these two instances, a very large excess of chain carrier was still needed (>5 equiv).

In view of the above, we wondered if tuning the L_B_ of LBR could be an effective strategy to obtain a smooth radical chain to achieve *intermolecular* RRH without the need for superstoichiometric agents or high temperatures. Herein, we describe the use of NHC-ligated boranes^20^ for the successful implementation of this hypothesis (Scheme 1C) for C(sp^3^)–C(sp^3^) and C(sp^3^)–C(sp^2^) bond formation.

Our studies commenced with the radical hydroalkylation of dibutyl maleate (**2a**) in acetonitrile using 2 equiv of iodocyclohexane (**1a**) as organic halide to form dibutyl 2-cyclohexylsuccinate (**3**), upon exposure to 390 nm for 12 h (Table 1).^21^ A diverse set of easily accessible ligated boranes (**B1**–**5**, 1 equiv) was evaluated under those reaction conditions, and we found that only NHC-ligated borane **B1** promoted the targeted reactivity (Table 1, entry 1: 75% ^1^H NMR yield). While other ligated boranes (**B2**–**5**) did not afford **3** (entry 2), we noticed that when the pyridine-ligated borane **B2** was used, **2a** was quantitatively reduced to dibutyl succinate. Next, the green solvent ethyl acetate proved to be the best medium for this reaction (see Section 5 in SI), allowing us to obtain **3** in excellent yield (entry 3: 92% ^1^H NMR yield). Gratifyingly, when equimolar amounts of **1a**, **2a** and **B1** were used, the reaction remained efficient (entry 4: 89%). Control experiments revealed that the presence of both **B1** and light was essential for the observed reactivity (entries 5 and 6, respectively). Moreover, heating the mixture to 60 °C in the dark or using blue light (λ = 456 nm) did not lead to any appreciable amounts of **3** (entries 7–8). Notably, when the radical acceptor **2a** was absent, **1a** was partly reduced to cyclohexane (entry 9), which is in line with previous reports.^22^

**Table 1:**
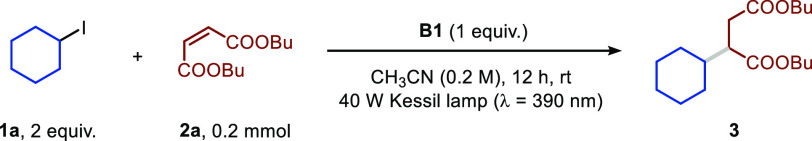

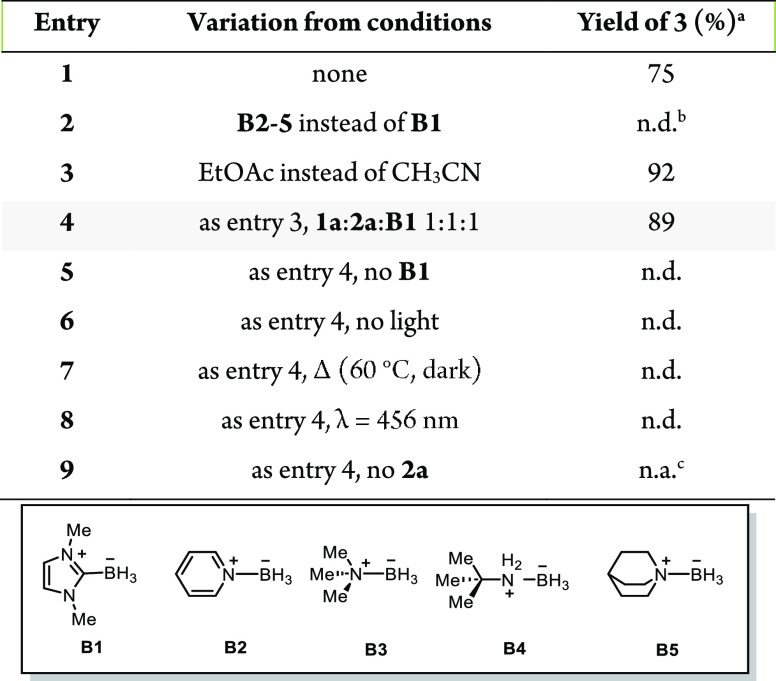
Optimization of the Reaction Conditions

a ^1^H NMR yields are given using CH_2_Br_2_ as internal standard.

b When **B2** was used, quantitative reduction of **2a** to dibutyl succinate was observed; in all the other cases a consumption <20% of **2a** was consistently observed.

c Formation of cyclohexane was observed. n.d.: not detected. n.a.: not applicable.

With these optimized conditions in hand, we evaluated the generality of this photoinduced LBR-mediated hydroalkylation protocol (Scheme 2). We were pleased to find that a diverse set of tertiary, secondary, and primary alkyl iodides were all effective and yielded the targeted adducts in good to excellent yields. Model compound **3** was obtained in an 81% yield after isolation. Similarly, compounds **4** and **5** deriving from the reaction between dibutyl maleate (**2a**) and 2-iodopropane or 2-iodobutane, respectively, were prepared in very good yields (80–87%). Next, we subjected various primary alkyl iodides to our reaction conditions and found that the length of the alkyl chain did not impact the reactivity: products **6**–**8** were all obtained in good yields (78–93%). Next, we turned our attention to the activation of iodomethane, especially in view of the limited documentation regarding the generation of methyl radicals from this precursor.^23^ Interestingly, the hydromethylation of **2a** proceeded smoothly (**9**, 65%), although an excess of the iodoalkane was required. The use of deuterated iodomethane led to **9-*****d***_***3***_ in a 67% yield after isolation. Other primary organic iodides with substituents in the β-position were activated by the boryl radical, and the corresponding adducts could be isolated in good yields (**10**–**11**, 55–77%). Notably, 1-chloro-3-iodopropane could be selectively cleaved at the C–I bond, yielding adduct **12** in 75% yield. Various tertiary alkyl iodides were also competent reaction partners in our hydroalkylation protocol, resulting in the formation of the corresponding products (**13**–**15**, 70–84%). We also successfully activated an aryl iodide to access the corresponding highly aggressive aryl radical, which was trapped by ethyl acrylate to yield product **16** in serviceable yield.^24^ On the one hand, formation of the reduced arene (*ca*. 10%) was observed, suggesting that the high reactivity of the aryl radical negatively impacts chain propagation. On the other hand, this finding hints the possibility to exploit LBR-mediated XAT to also forge C(sp^2^)–C(sp^3^) bonds.

**Scheme 2:**
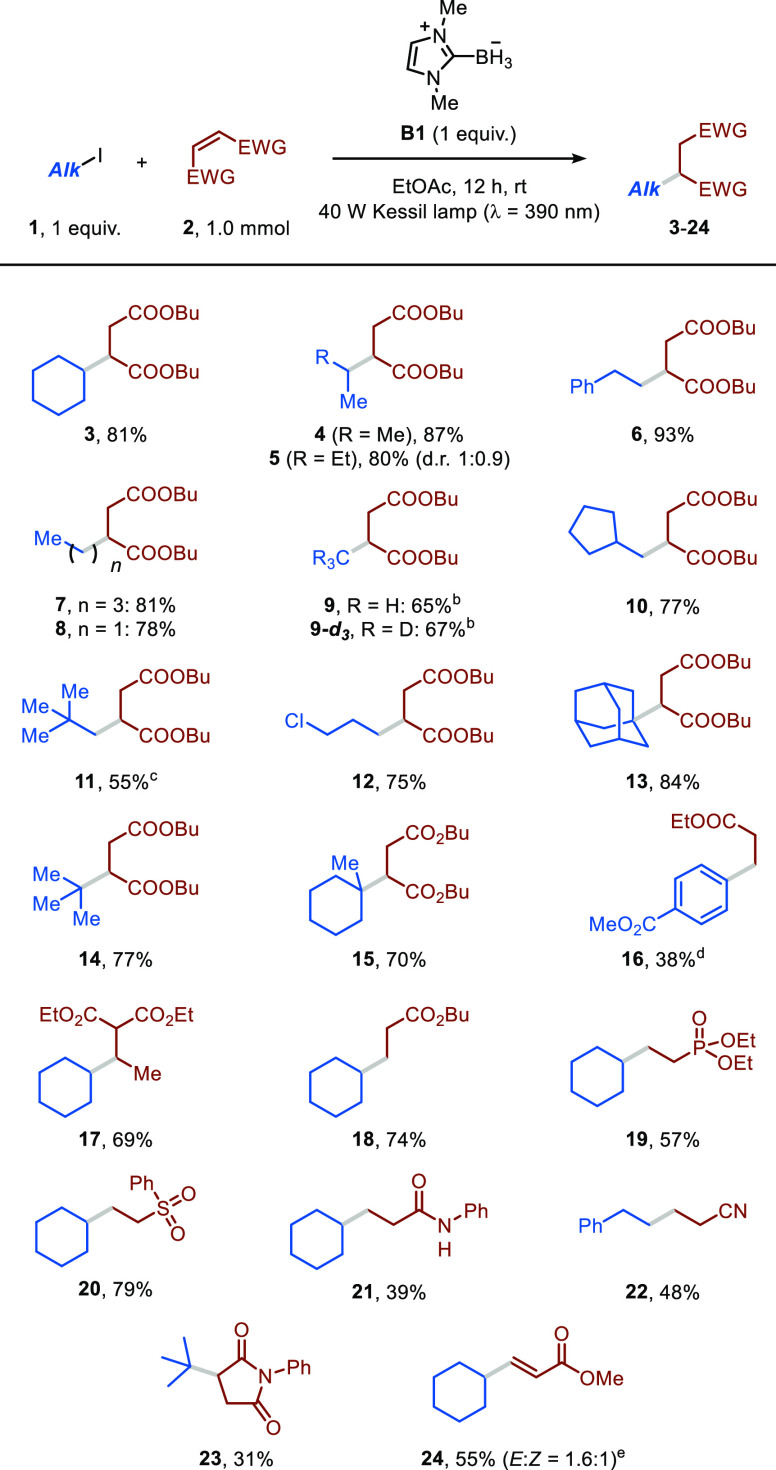
Substrate Scope for LBR-Mediated XAT under UV-Light Irradiation **2a** (1.0 mmol), **1a** (1 equiv), **B1** (1 equiv) in EtOAc (5 mL); solution degassed through N_2_ bubbling (5 min) prior to irradiation (λ = 390 nm). See Section 9 in SI for further details. 10 equiv of iodomethane (**1g** or **1g-*****d***_***3***_) were used. 2 equiv of 1-iodo-2,2-dimethylpropane (**1i**) were used. Methyl 4-iodobenzoate was used as organohalide. Methyl propiolate was used as the radical trap.

Unfortunately, unbiased alkyl bromides could not be activated under our conditions. Finally, the reaction remained efficient when engaging other electron-poor olefins as radical acceptors, and the expected products were isolated in moderate to good yield (**17**–**23**, 31–74%), showcasing the tolerance of this manifold toward a wide variety of functional groups, including esters, phosphonates, sulfone, amides, nitriles, and imides. Remarkably, an electron-poor alkyne was also amenable to this transformation, and the corresponding alkenoate was isolated in 55% yield (**24**) in an ∼2:1 diastereomeric ratio.

Next, we set off to get more insights into the reaction mechanism. UV–vis analysis of all the components involved in the transformation (**1a**, **2a**, and **B1**) showed that only iodocyclohexane absorbs light above 350 nm (see Section 6.1 in the SI). We propose that this weak absorption is responsible for the homolysis of the C–I bond.^25^ The occurrence of an EDA complex was excluded, as the mixture of **1a** and **B1** showed only additive absorbance. Next, we measured a quantum yield of 1.4 for the process (see Section 6.2 in SI), which suggests the occurrence of a radical chain.^26^

We also carried out a closer computational inspection of the S_H_2-based elementary steps of the radical chain (i.e., XAT and HAT). We aimed to understand why **B1** is uniquely suited as a radical chain carrier for this intermolecular RRH (Scheme 3A). Thus, the reaction between iodocyclohexane and dimethyl maleate was considered in the presence of different ligated boranes (**B1**–**3**). We employed Density Functional Theory (DFT) at the ωB97xD/def2TZVP level to optimize the pertinent stationary points, and the solvent effect was evaluated via an implicit model by means of single-point calculations on the obtained structures (MeCN bulk; see Sections 7 and 8 in SI). We found that among the evaluated LBRs, the tertiary amine-ligated one (deriving from **B3**) exhibited the highest halogen affinity, proving the most effective halogen abstractor within the series (barrierless XAT, Δ*G*_XAT_ = −29.0 kcal·mol^–1^; Table S5 in the SI). In contrast, the pyridine-ligated boryl radical (from **B2**) displayed the lowest affinity (Δ*G*^‡^_XAT_ = +18.3 kcal·mol^–1^, Δ*G*_XAT_ = −6.4 kcal·mol^–1^), while the presence of the NHC ring imparted intermediate properties (Δ*G*^‡^_XAT_ = +11.6 kcal·mol^–1^, Δ*G*_XAT_ = −13.0 kcal·mol^–1^).^7c^ Conversely, when the parent ligated boranes were evaluated in the role of hydrogen atom donors to get the desired product, a different trend was observed. In fact, HAT from **B3** showed a significantly higher barrier (Δ*G*^‡^_HAT_ = +18.1 kcal·mol^–1^, Δ*G*_HAT_ = +6.4 kcal·mol^–1^) compared to **B2** and **B1**, for which Δ*G*^‡^_HAT_ = +14.0 (Δ*G*_HAT_ = −17.4 kcal·mol^–1^) and +12.4 kcal·mol^–1^ (Δ*G*_HAT_ = −11.9 kcal·mol^–1^), respectively. Noteworthy, the calculated energy changes for the three HAT steps are in line with the BDE values of the B–H bonds reported in the literature for the considered borane species.^27^ Altogether, these data suggest that **B1** is uniquely positioned among ligated boranes in sustaining the targeted RRH due to the more balanced properties in the XAT and HAT steps. Of note, computations revealed that the premature reduction of the cyclohexyl radical to cyclohexane by the ligated boranes is more kinetically disfavored than by silanes and tin hydride (see Table S5 in SI).

**Scheme 3:**
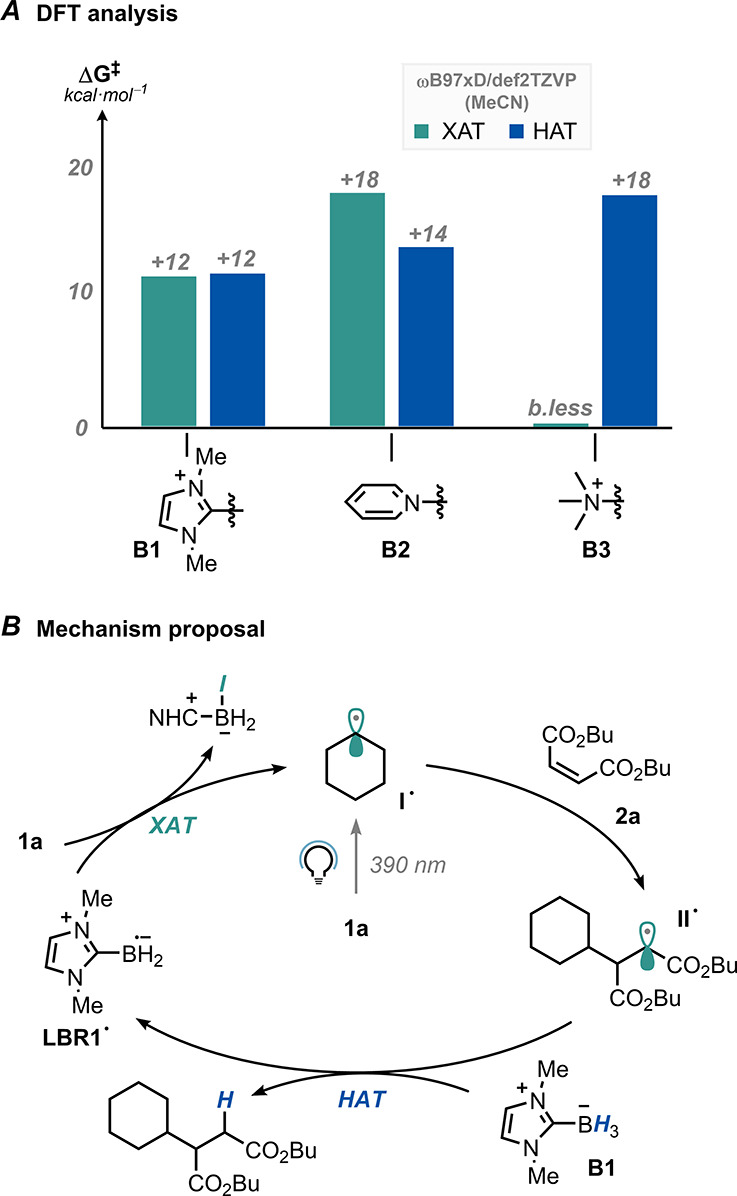
(A) DFT Analyses of the XAT and HAT Steps for Representative LBRs; (B) Proposed Mechanism

Given the above, a proposed mechanism is displayed in Scheme 3B. Thus, **1a** is photolyzed under UV-light irradiation to deliver the cyclohexyl radical (**I**^**•**^). The latter species is trapped by electron-poor olefin **2a** to deliver the electrophilic radical adduct **II**^**•**^. Noteworthily, cyclohexane is formed if **2a** is omitted: this happens via a sluggish, polarity-mismatched hydrogen atom abstraction from **B1** (Δ*G*^‡^_HAT_ = +17.3 kcal·mol^–1^).^28^**II**^**•**^ reacts with **B1** via a polarity-matched hydrogen atom abstraction to yield the expected hydroalkylated product **3**, together with a fresh NHC-ligated boryl radical (**LBR1**^•^). The latter species can activate a new molecule of **1a** via a XAT step,^7c^ thus kicking off a radical chain. Radical quenching and competition experiments are in agreement with this scenario (section 6 in the SI).

Finally, a comparison with other tactics for light-mediated, photocatalyst-free reductive radical hydroalkylation via XAT is in order. In one of these instances, the Dilman group reported the radical silyldifluoromethylation of electron-deficient alkenes capitalizing on the same LBR.^29^ However, the development of the reaction scope was frustrated by the need for a halogen-bonding interaction between the substrate and an NHC borane. As previously discussed, the Gaunt and Noël groups utilized tris(trimethylsilyl)silane (TTMS) as a radical chain carrier. It is worth noting, however, that TTMS requires refrigeration due to its relatively fragile nature, while **B1** is a bench-stable solid, enhancing operational simplicity of our protocol. Additionally, the removal of boron-containing byproducts during aqueous workup significantly streamlines the purification process of the product. In contrast, silicon-based byproducts necessitate column chromatography for removal. Moreover, our approach represents a more atom-economical approach to reductive hydroalkylation.

In summary, we have presented a method for the reductive hydroalkylation and hydroarylation of electron-poor olefins using NHC-ligated boranes without the need for a photocatalyst. This approach aligns with the recent trend of developing sustainable XAT methodologies, offering benefits, such as operational simplicity and cost-effectiveness. Compared to previously established methods, this approach (i) appears to be well-suited for generating highly reactive primary alkyl radicals, as well as the methyl radical, (ii) eliminates the need for any reagents to be used in superstoichiometric amounts, and (iii) smoothly takes place at room temperature and in the absence of photocatalysts and/or initiators, thus increasing the attractivity of this manifold.

## Data Availability

The data underlying this study are available in the published article and its Supporting Information.

## Supporting Information Available

Experimental procedures, characterization data of synthesized compounds, copies of NMR spectra, computational details, IRC and PES profiles, optimized geometries (PDF)
