# Supplementary material for: Photoinduced Intermolecular Radical Hydroalkylation of Olefins via Ligated Boryl Radicals-Mediated Halogen Atom Transfer
Source: Org Lett. 2024 Jul 1;26(27):5839–43. doi: 10.1021/acs.orglett.4c02034 (PMC11250028; doi:10.1021/acs.orglett.4c02034)
Supplement: Supplementary file 1 — ol4c02034_si_001.pdf [file ol4c02034_si_001.pdf]

Supporting Information

Photoinduced Intermolecular Radical Hydroalkylation of Olefins via Ligated Boryl Radicals-mediated Halogen Atom Transfer

Ting Wan,[a,b] Łukasz W. Ciszewski,[a] Davide Ravelli[c] and Luca Capaldo[a,d,*]

[a] *Flow Chemistry Group, van't Hoff Institute for Molecular Sciences (HIMS), University of Amsterdam, 1098 XH Amsterdam, The Netherlands.*

[b] *The Research Center of Chiral Drugs, Innovation Research Institute of Traditional Chinese Medicine, Shanghai University of Traditional Chinese Medicine, Shanghai 201203, China.*

[c] *PhotoGreen Lab, Department of Chemistry, University of Pavia, 27100 Pavia, Italy.*

[d] *SynCat Lab, Department of Chemistry, Life Sciences and Environmental Sustainability, University of Parma, 43124 Parma, Italy.*

* *Email: luca.capaldo@unipr.it*

Contents

1. General information **S4**

2. Reactor design **S5**

UFO reactor *S5*

3. Charts of starting materials **S7**

Organic halides S7

Electron-poor olefins S7

4. Synthesis of starting materials **8**

Synthesis of **B1** S8

Synthesis of organic halides S8

5. Optimization of reaction conditions **S9**

5.1 Screening of ligated boranes S9

5.2 Screening of solvents S10

5.3 Optimization of substrates ratio S10

5.4 Control experiments S11

6. Mechanistic investigation **S12**

6.1 UV-Vis spectroscopic analysis S12

6.2 Quantum yield measurements S12

6.3 Competition experiments S13

6.4 Radical trapping experiments S14

7. DFT analysis **S17**

8. Computational Details **S22**

8.1. IRC Plots S30

8.2. Relaxed PES Scan S42

8.3. Optimized Structures S45

9. General procedures for preparative experiments S84

10. Characterization data S84

11. References S92

12. NMR spectra S94

12.1 NMR spectra of starting materials S94

12.2 NMR spectra of products S97

1. General information

Reagents and consumables. All reagents and solvents were bought from Sigma Aldrich, TCI, Flurochem, VWR International and Biosolv and used as received. Disposable syringes were purchased from Laboratory Glass Specialist. Product isolation was performed manually, using silica (P60, SILICYCLE), or automatically, by a Biotage® Isolation Four, with Biotage® SNAP KP-Sil 20 or 50 g flash chromatography cartridges. TLC analysis was performed using Silica on aluminum foils TLC plates (F254, SILICYCLE) with visualization under ultraviolet light (254 nm and 365 nm) or appropriate TLC staining (potassium permanganate or cerium ammonium molybdate).

NMR spectroscopy. ^{1}H (400 MHz or 300 MHz), ^{13}C (101 MHz or 75 MHz) and ^{31}P (121 MHz) spectra were recorded unless stated otherwise on ambient temperature using a Bruker AV400 or a Bruker AV300. ^{1}H NMR spectra are reported in parts per million (ppm) downfield relative to $CDCl_3$ (7.26 ppm) or CD_2Cl_2 (5.32 ppm) and all ^{13}C NMR spectra are reported in ppm relative to $CDCl_3$ (77.2 ppm) or CD_2Cl_2 (53.8 ppm) unless stated otherwise. The multiplicities of signals are designated by the following abbreviations: s (singlet), d (doublet), t (triplet), q (quartet), m (multiplet), dd (doublet of doublets), dt (doublet of triplets), td (triplet of doublets), tt (triplets of triplets), ddd (doublet of doublet of doublets), qd (quartet of doublet). Coupling constants (J) are reported in hertz (Hz). NMR data was processed using the MestReNova 14 software package. Known products were characterized by comparing to the corresponding ^{1}H NMR, ^{13}C NMR and ^{31}P NMR with those available in the literature.

Melting point. Melting points were measured using a Büchi Melting Point M-565 apparatus.

Mass spectrometry. High resolution mass spectra (HRMS) were collected on an AccuTOF LC, JMS-T100LP Mass spectrometer (JEOL, Japen).

UV-Vis spectroscopy. UV-Vis spectra were recorded with a double beam spectrophotometer Shimadzu UV2700 equipped with a deuterium lamp (190-350 nm), a halogen lamp (330-900 nm) and a photomultiplier (Hamamatsu R928). Measurements were performed in a quartz cuvette (optical path: 1 cm). All spectra were recorded in CH_3CN (solvent cutoff: 190 nm) in quartz cuvettes (optical path: 1 cm) with a bandwidth of 5 nm and a data pitch of 1 nm.

2. Reactor design

UFO reactor

For all batch experiments a homemade, a 3D-printed reactor was adopted and used as described elsewhere.[1] The reactor was designed to fit reaction vials and to be equipped with a Kessil lamp PR160L series (λ_{em} = 390 nm). The reactor was designed in Adobe Inventor 2021 with 4 different parts. The lid (100 mm × 12 mm) is designed to host up to 8 reactions vials and holds the Kessil lamp in the center (**Figure S1**A); a fan (SUNON DCLüfter 24 V; 50x50x15 Vapo RoHS) is mounted on the bottom of the reactor for cooling. The box is designed with holes to allow the air flow to escape the reactor and keep the temperature stable around 30−33 °C (**Figure S1**B), as measured by an external thermometer. A reflector is situated underneath the lamp and reflects the photons inside the box to have homogeneous light distribution (**Figure S1**C). Finally, the stirring plate adapter (**Figure S1**D) was added to fix the system on a stirring plate and provide homogeneous stirring (**Figure S1**E). It also spaces the reflector from the plate to ensure a continuous air flow from the top to the bottom of the system. All the inside surfaces were covered with reflective tape. An overview of the assembled reactor is shown in **Figure S1**.

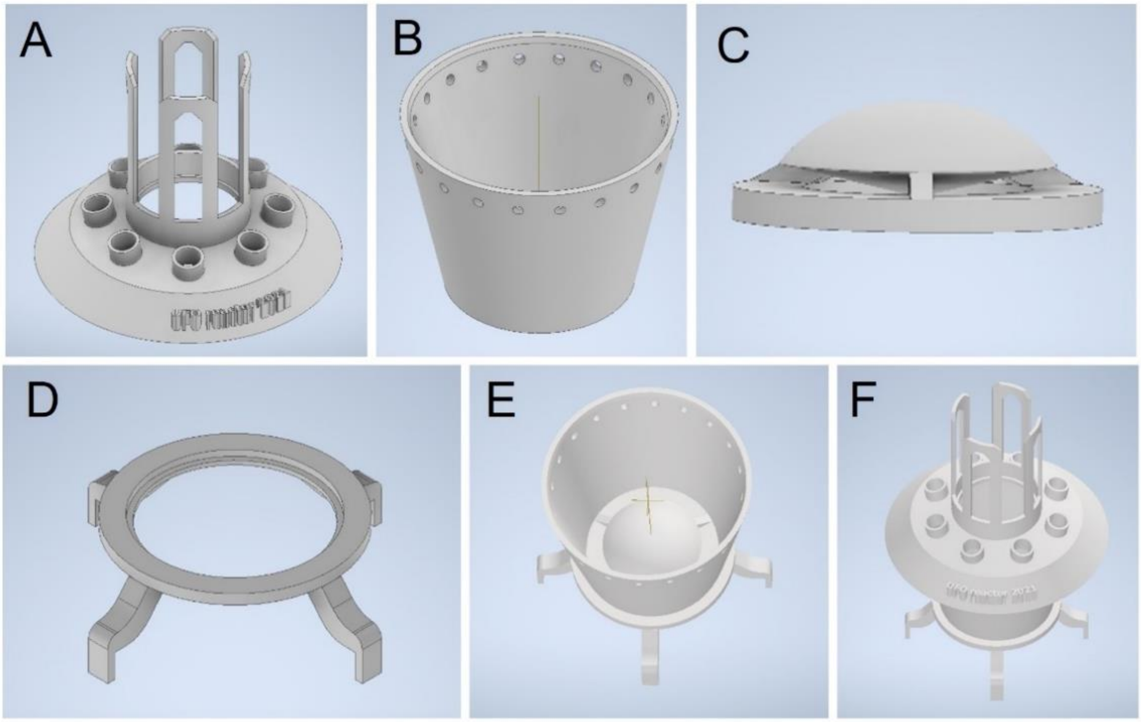

Figure S1: Overview of the 3D-printed reactor: A) lid designed to host up to 8 reactions vials and hold the Kessil lamp in the center; B) body of the reactor; C) light reflector: it is coated with reflective tape; D) adapter for stirring plate; E) inside of the reactor; G) overall reactor. Reprinted with permission from

Wan, T.; Capaldo, L.; Ravelli, D.; Vitullo, W.; de Zwart, F. J.; de Bruin, B.; Noël, T. Photoinduced Halogen-Atom Transfer by N-Heterocyclic Carbene-Ligated Boryl Radicals for C(sp^3)-C(sp^3) Bond Formation. *J. Am. Chem. Soc.* **2023**, *145*, 991−999. DOI: 10.1021/jacs.2c10444. Copyright © 2022 The Authors.

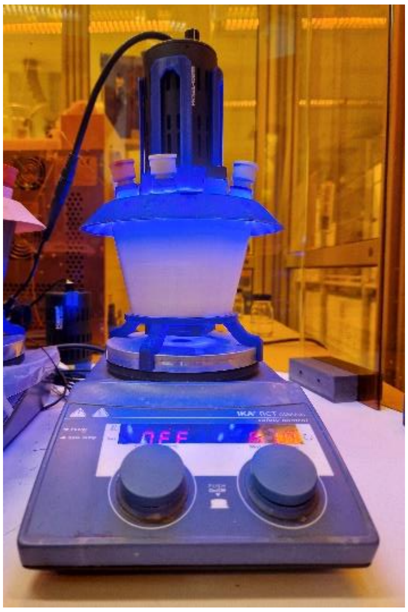

Figure S2: Picture of the assembled reactor equipped with a Kessil lamp (λ_{em} = 390 nm). Reprinted with permission from Wan, T.; Capaldo, L.; Ravelli, D.; Vitullo, W.; de Zwart, F. J.; de Bruin, B.; Noël, T. Photoinduced Halogen-Atom Transfer by N-Heterocyclic Carbene-Ligated Boryl Radicals for C(sp^3)-C(sp^3) Bond Formation. *J. Am. Chem. Soc.* **2023**, *145*, 991−999. DOI: 10.1021/jacs.2c10444. Copyright © 2022 The Authors.

3. Charts of starting materials

Organic halides

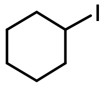
1a

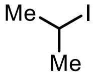
1b

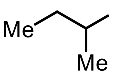
1c

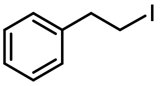
1d

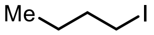
1e

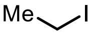
1f

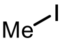
1g

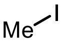
1g-*d*$_3$

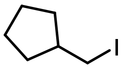
1h

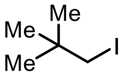
1i

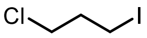
1j

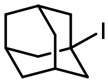
1k

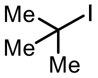
1l

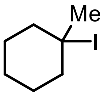
1m*

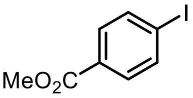
1n

* Homemade

Electron-poor olefins

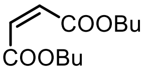
2a

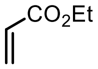
2b

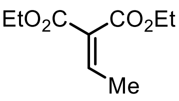
2c

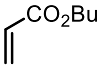
2d

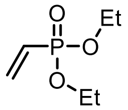
2e

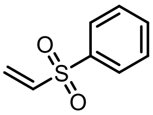
2f

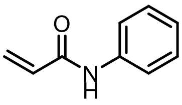
2g

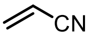
2h

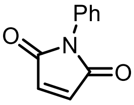
2i

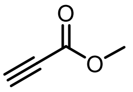
2j

4. Synthesis of starting materials

Synthesis of **B1**

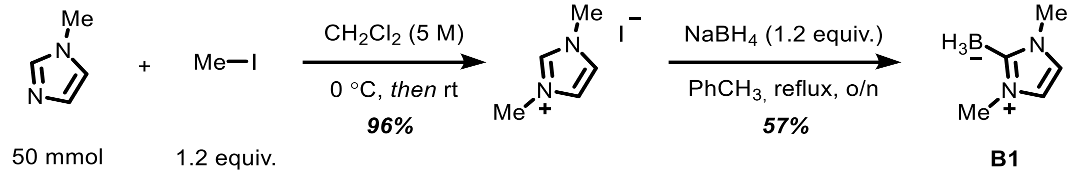

B1 was prepared by following a procedure reported in the literature.[2] At 0 °C, methyl iodide (1.2 equiv) was added dropwise to a CH_2Cl_2 solution of 1-methylimidazole (50 mmol, 5.0 M) over 30 minutes. The ice bath was removed and the reaction mixture was allowed to stir for 1 h at room temperature, after which the mixture was concentrated and dried under vacuum to give 1,3-dimethyl-1H-imidazol-3-ium iodide (10.8 g, 96%). Without purification, 1,3-dimethyl-1H-imidazol-3-ium iodide (10.8 g, 48 mmol) was suspended in toluene (1 mL/mmol) and sodium borohydride (1.2 equiv) was added in one portion. The mixture was refluxed overnight. The hot reaction solvent was decanted from the insoluble mixture, and the remaining residue was extracted with hot toluene (2×1 reaction volume), and concentrated under reduced pressure. Purification by flash chromatography on silica gel (Cyclohexane:Ethyl Acetate 20:80) to give **B1** as a white solid (3 g, 57%; 54% over two steps). Spectroscopic data are in accordance with the literature.[2]

^{1}H NMR (300 MHz, $CDCl_3$) δ 6.81 (s, 2H), 3.75 (s, 6H), 1.49 – 0.53 (m, 3H).

^{13}C NMR (75 MHz, $CDCl_3$) δ 120.0, 36.1. The signal of the α-B-carbon was not observed.

^{11}B NMR (96 MHz, $CDCl_3$) δ -37.5 (q, J = 86.3 Hz).

Synthesis of organic halides

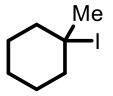

1-Iodo-1-methylcyclohexane (1m). Prepared by following a procedure reported in the literature.[3] Compound **1m** is unstable and has to be stored in the dark at −22 °C. ^{1}H NMR (300 MHz, $CDCl_3$) δ 2.22 – 2.06 (m, 5H), 1.74 – 1.61 (m, 5H), 1.33 – 1.13 (m, 1H), 1.09 – 0.91 (m, 2H). ^{13}C NMR (75 MHz, $CDCl_3$) δ 59.6, 46.0, 39.2, 25.4, 25.2.

5. Optimization of reaction conditions

The optimization of the reaction conditions was carried out by studying the radical addition of iodocyclohexane (**1a**) onto dibutyl maleate (**2a**) to give dibutyl 2-cyclohexylsuccinate (**3**) on a 0.2 mmol scale (see Table S1-S4).

In a 7 mL vial equipped with a screw cap **2a** (0.2 mmol), **1a** (*n* equiv.) and ligated borane (*n* equiv.) were dissolved in 1.0 mL of the chosen solvent. The mixture was bubbled with N_2 (1 min) and irradiated with a 40 W Kessil lamp (λ = 390 nm, full intensity) in the UFO reactor (**Figure S1**). Consumption of **2a** was found to be complete (by TLC) after 12 hour of continuous irradiation with this setup. After irradiation, CH_2Br_2 was added as external standard: 0.2 mL of the reaction crude were withdrawn, diluted with 0.3 mL of CD_3CN and analyzed via ^{1}H-NMR.

5.1 Screening of ligated boranes

Table S1: Screening of ligated boranes

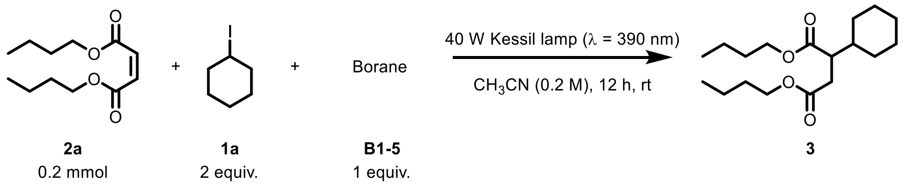

Entry	Ligated boranes	Yield of **3** (%)[a]
1	**B1**	75
2	**B2**	n.d.[b]
3	**B3**	n.d.
4	**B4**	n.d.
5	**B5**	n.d.

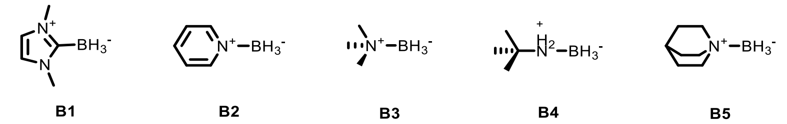

[a] Yields determined by ^{1}H-NMR, CH_2Br_2 as external standard. [b] When **B2** was used, complete reduction of **2a** to dibutyl succinate was observed; in all the other cases a consumption <20% of **2a** was consistently observed.

5.2 Screening of solvents

Table S2: Screening of solvents.

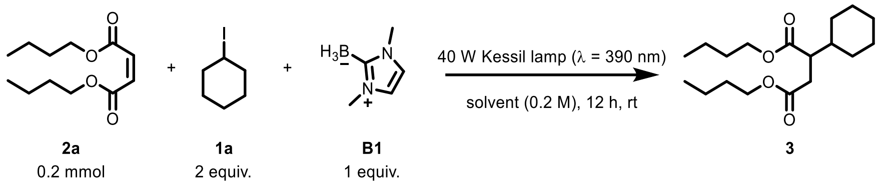

Entry	Solvent	Yield (%)[a]
1	Hexane	28
2	Toluene	91
3	DCE	86
4	*t*BuOH	80
5	EtOAc	92
6	CH_3OH	75
7	CH_3CN	75
8	CH_3CN/H_2O (9:1)	81
9	Me_2CO	Messy crude

[a] Yields determined by ^{1}H-NMR, CH_2Br_2 as external standard.

5.3 Optimization of substrates ratio

Table S3: Screening of substrates ratio.

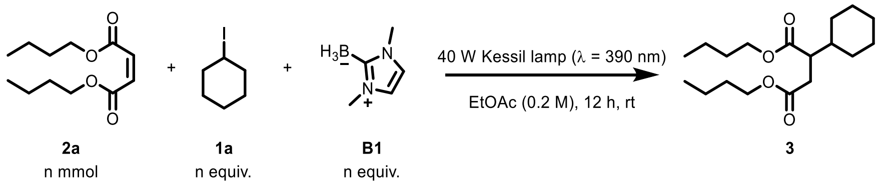

Entry	Substrates ratio (**2a**:**1a**:**B1**)	Yield (%)[a]
1	1:2:1	92
2	1:1:1	89
3	1:1:1.2	88

[a] Yields determined by ^{1}H-NMR, CH_2Br_2 as external standard.

5.4 Control experiments

Table S4: Control experiments.

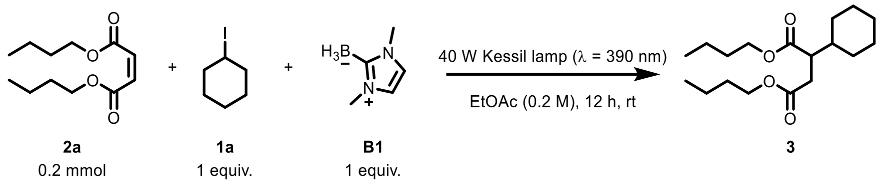

Entry	Changes	Yield (%)[a]
1	Without **1a**	n.a.[b]
2	Without **2a**	n.a.[c]
3	Without **B1**	n.d.
4	Dark or 456 nm	n.d.
5	Heating at 60 °C in the dark	n.d.

[a] Yields determined by ^{1}H-NMR, CH_2Br_2 as external standard. [b] starting materials were recovered untouched. [c] formation of cyclohexane was observed.

6. Mechanistic investigation

6.1 UV-Vis spectroscopic analysis

Prompted by Dilman's report on the radical silyldifluoromethylation of electron-deficient alkenes,[4] where a halogen bonding responsible for weakening the C–I bond was claimed, we decided to investigate ground state interactions between the reactants in our mixture. In particular, we started by recording an absorption spectrum of single components (**1a**, dimethyl maleate and **B1**) and that of a mixture of **1a** and **B1** to seek for the formation of an EDA complex (**Figure S3**).

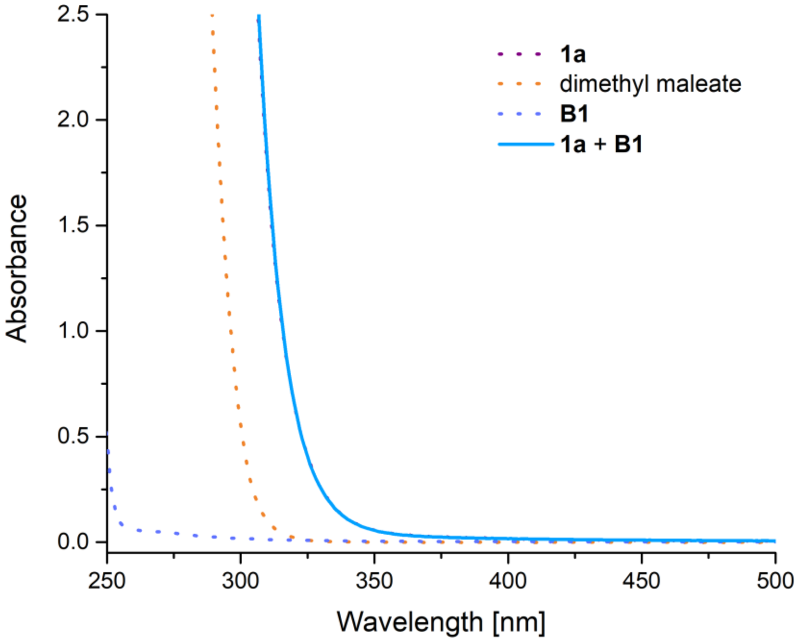

Figure S3. Absorption spectra of borane **B1**, iodocyclohexane (**1a**), dimethyl maleate and a mixture of **1a** and **B1**. All spectra were recorded 0.2 M in EtOAc (solvent cutoff: 190 nm) in quartz cuvettes (optical path: 1 cm) with a bandwidth of 5 nm and a data pitch of 1 nm. Scan rate: medium.

In our experiments we could not find any evidence for the formation of an EDA complex. We propose that the weak absorption of UV-light by alkyl iodides, known to generate C-radicals upon irradiation, initiates the reaction.[5]

6.2 Quantum yield measurements

As described elsewhere,[6] we calculated a photon flux of $1.61 \cdot 10^{-6}$ E s^{-1} for the 40 W Kessil lamp PR160L (390 nm, full intensity) using the reactor shown in **Figure S1**.

Our model reaction was run under identical experimental conditions: a N_2-bubbled EtOAc solution (0.2 M) of **1a** (1 equiv.), **2a** (0.6 mmol) and **B1** (1 equiv.) was irradiated using the reactor shown in **Figure S1**. The yield was monitored in time and the value after 1 h was adopted for computing the quantum yield.

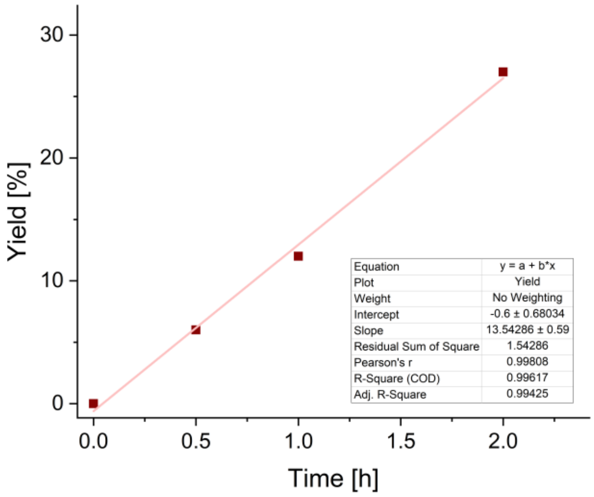

Figure S4. Initial kinetic profile for the formation of compound **3**.

After 3600 s of irradiation, $7.2 \cdot 10^{-5}$ mol of product **3** (corresponding to 12% ^{1}H-NMR yield) were produced (**Figure S4**). We estimate a lower value for the quantum yield to be 1.4, which is in agreement with a radical chain mechanism.

6.3 Competition experiments

To get further insights into the reaction mechanism, we performed a competition experiment where we reacted a 1:1:1 mixture of a primary (iodobutane, **1e**), secondary (iodocyclohexane, **1a**) and tertiary (1 iodoadamantane, **1k**) organohalides (2 equiv.) with **2a** (0.5 mmol). These stoichiometric ratio was adapted from our previous report for the sake of comparability.[7] The yields of the corresponding products were calculated by GC-MS by means of calibration curves built with authentic samples and results are reported in **Figure S5**.

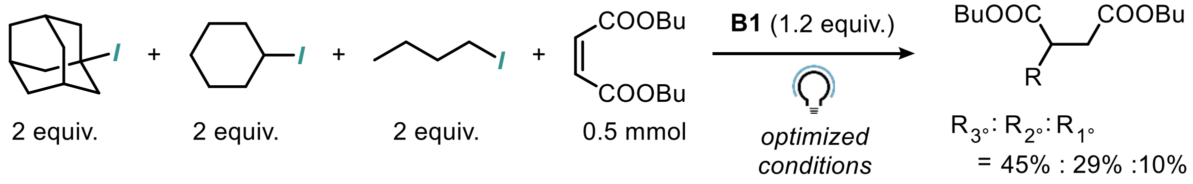

Figure S5. Competition experiments with different alkyl iodides.

Product distribution correlates with the stability of the C-centered radicals generated via XAT. Given the results above, we conclude that the same ligated borane was generated during the course of the reaction.

6.4 Radical trapping experiments

To evaluate the effect of the free radical TEMPO (2,2,6,6-tetramethylpiperidine *N*-oxyl) on the reaction outcome, we performed a set of experiments by subjecting cyclohexyl iodide (**1a**) and dimethyl maleate to our optimized conditions in the presence of ligated borane **B1**. The crude reaction mixtures related to the experiments indicated in **Scheme S1** have been analyzed via GC-MS analysis (**Figure S6**), and the corresponding traces were compared with those of selected reference compounds (see **Figure S7**), while substrates cyclohexyl iodide **1a** and dimethyl maleate have been recognized through comparison with the database.

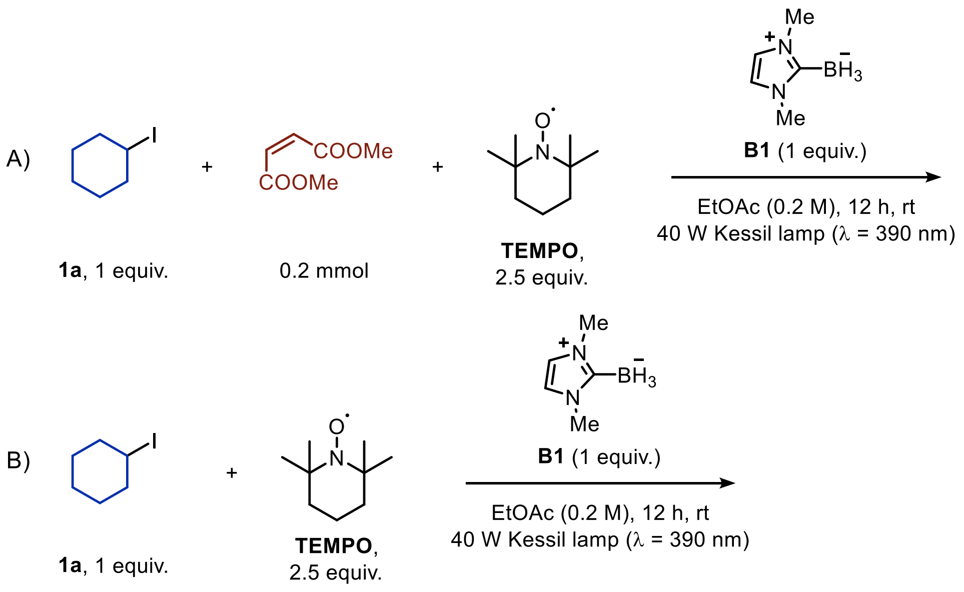

Scheme S1. Experiments performed to evaluate the effect of the TEMPO additive on the model reaction between cyclohexyl iodide **1a** and dimethyl maleate in the presence of ligated borane **B1**.

Inspection of the GC charts in **Figure S6** and comparison with the database revealed that the peak at 6.6 MIN is related to dimethyl maleate, while that at 7.7 min to cyclohexyl iodide (**1a**). Furthermore, by comparison with **Figure S7**, it is safe to assume that the peaks at 9.2 and 13.4 min are related to TEMPO and ligated borane **B1**, respectively. Furthermore, no additional significant peak is present in **Figure S6**A,B, with the only exception of the small peak at 16.0 min. On one hand, this demonstrates that the reaction from equation A in **Scheme S1** is completely inhibited in the presence of TEMPO. On the other hand, analysis of the MS

spectrum associated with the above mentioned 16.0 min peak, revealed that it is the adduct between TEMPO and the cyclohexyl radical, in accordance with previous data available in the literature.[8]

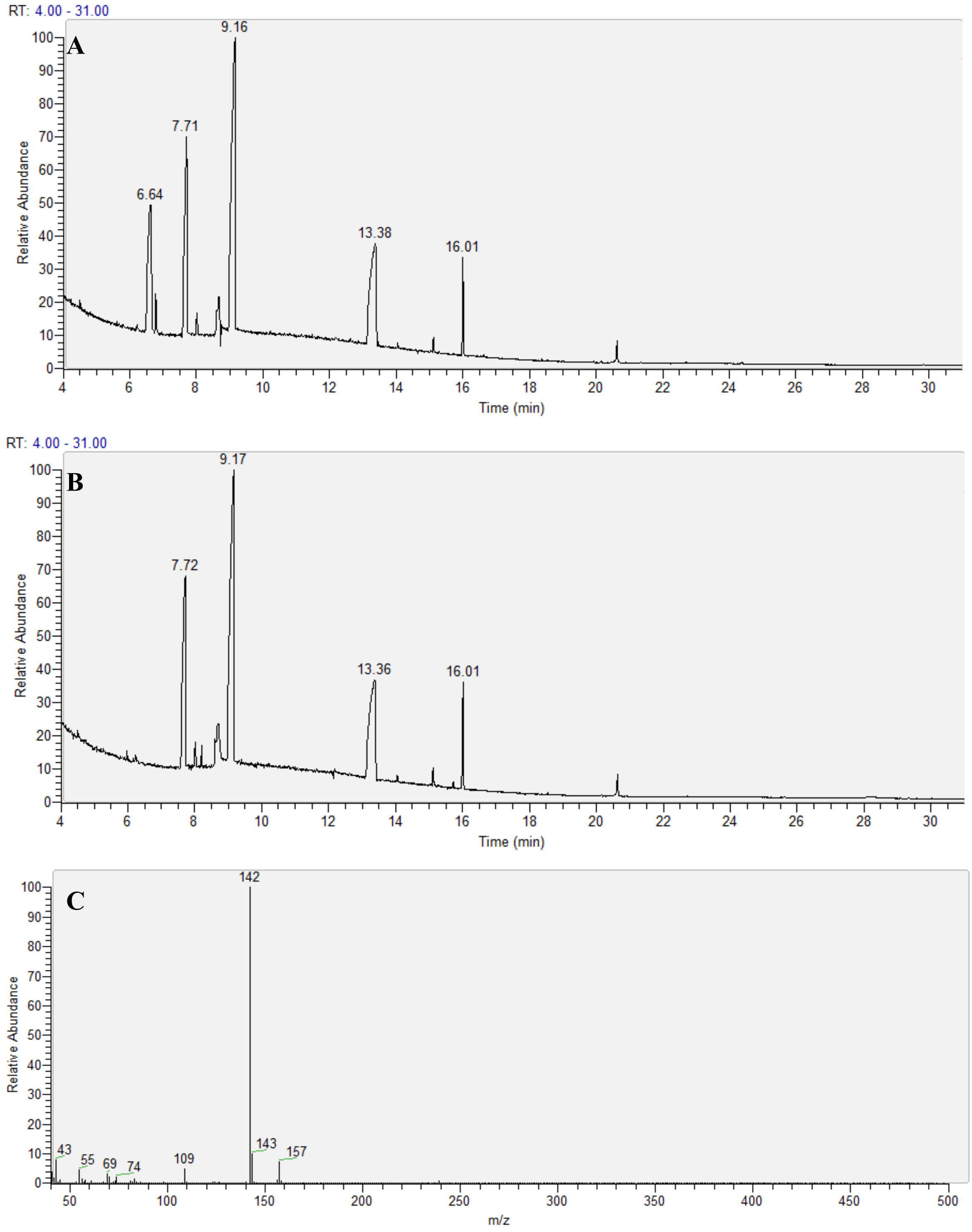

Figure S6. GC-MS traces of the crude reaction mixtures corresponding to the experiments gathered in **Scheme S1**: A) reaction between **1a** and dimethyl maleate in the presence of the TEMPO additive (2.5 equiv.); and B) reaction of **1a** alone with the TEMPO additive (2.5 equiv.). Panel C shows the mass spectrum of the peak at 16.01 min in **Figure S6**A,B.

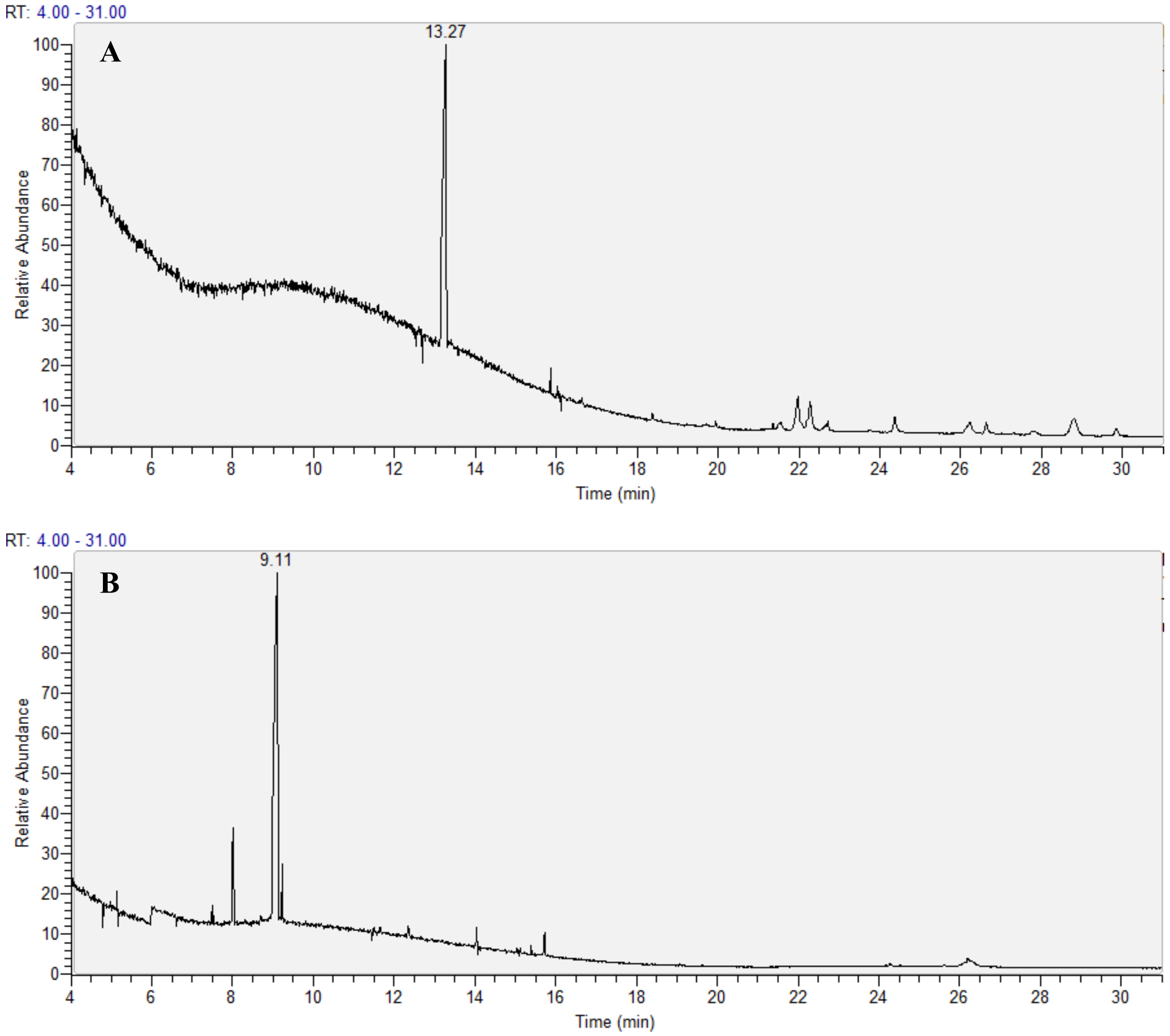

Figure S7. GC-MS chromatograms of selected reference compounds: A) ligated borane **B1**; and B) TEMPO additive.

7. DFT analysis

Strategy. Our computational work has been intended to model the reference reaction between iodocyclohexane (**1a**) and dimethyl maleate (**DMM**) to deliver dimethyl 2-cyclohexylsuccinate (**Prod**) in the presence of different radical chain carriers (**CC-H**), namely ligated boranes NHC-borane (**B1**), pyridine-borane (**B2**) and trimethylamine-borane (**B3**), as well as trimethylstannane (**Sn$_H$**) and tris(trimethylsilyl)silane (**Si$_H$**). The operating mechanistic scenario is depicted in **Scheme S2**, wherein three key steps can be recognized:

- The initial XAT step operated by the radical originating from the employed chain carrier (**CC$^•$**), described by **TS1**;
- The radical addition of cyclohexyl radical (**I$^•$**) to **DMM** to deliver radical adduct **II$^•$**, described by **TS2** (note: this is the only step independent from the chain carrier adopted);
- The final HAT step from the employed chain carrier (**CC-H**) to radical adduct **II$^•$**, described by **TS3**;

At the same time, we also modeled two possible competitive pathways:

- The addition of the radical originating from the chain carrier (**CC$^•$**) to **DMM**, proceeding via **TS4**;
- The premature reduction of cyclohexyl radical **I$^•$** by the employed chain carrier, described by **TS5**.

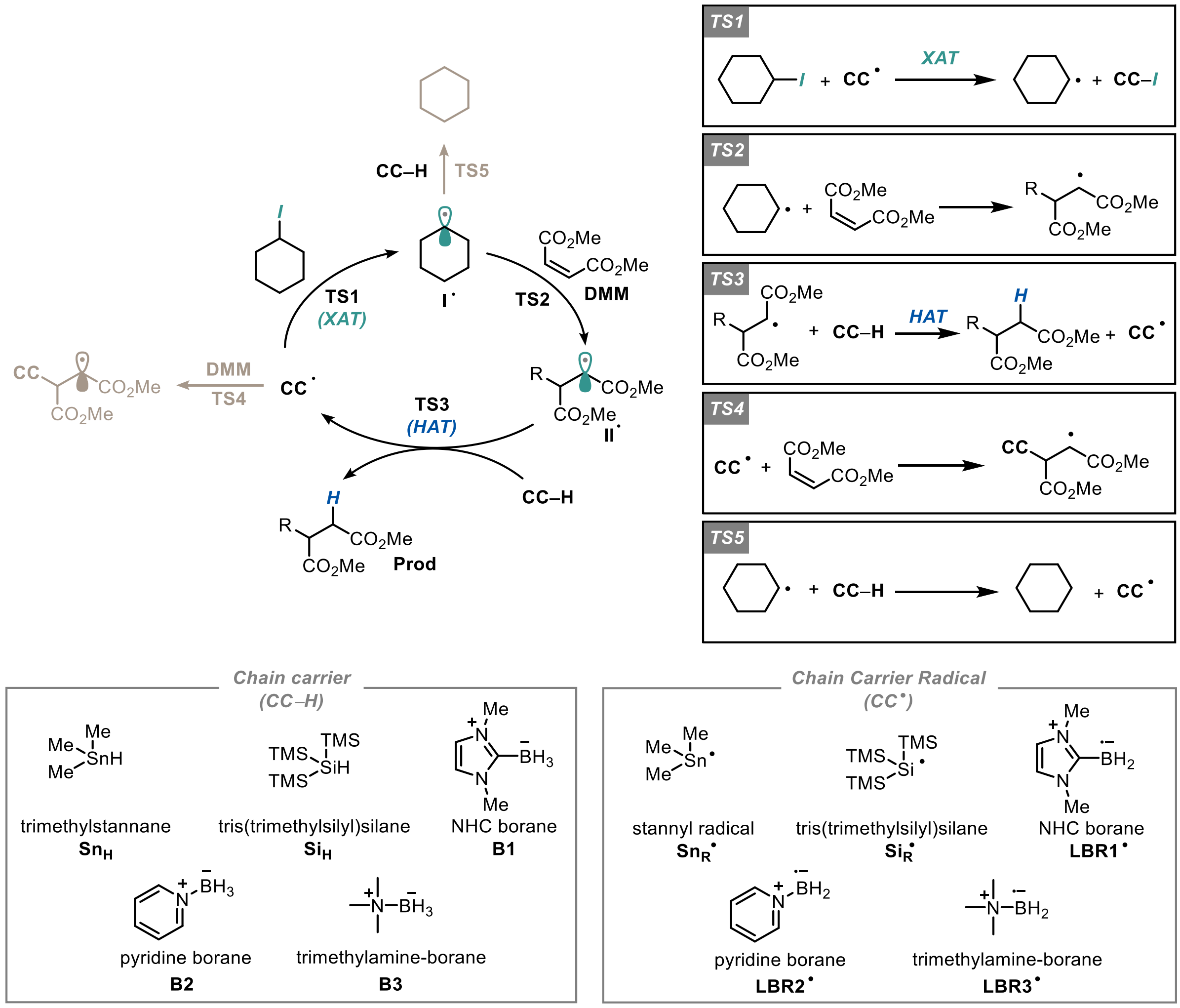

Scheme S2. Mechanistic scenario considered to implement our computational analysis.

Such description has been already reported in a previous work by us for the chain carrier **B1**,[7] while the complete reaction pathway is reported below for chain carriers **B2** (**Scheme S3**) and **B3** (**Scheme S4**). On the other hand, **Table S5** gathers the complete set of parameters determined for **TS1-5**, including $\Delta G^{\ddagger}$ and ΔG, in the case of the different chain carriers considered.

Table S5. Kinetic ($\Delta G^{\ddagger}$) and thermodynamic (ΔG) parameters obtained for **TS1-5** in the case of the different chain carriers (**CC-H**) considered, as determined from ωB97xD/def2TZVP calculations in bulk acetonitrile.[a]

**Chain Carrier (CC-H)**	**TS1 (XAT)** 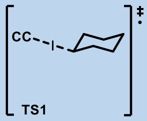		**TS2** 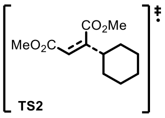		**TS3 (HAT)** 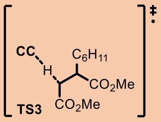		**TS4** 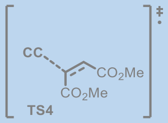		**TS5** 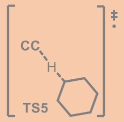	
	**ΔG‡**	**ΔG**	**ΔG‡**	**ΔG**	**ΔG‡**	**ΔG**	**ΔG‡**	**ΔG**	**ΔG‡**	**ΔG**
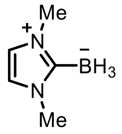 NHC borane **B1**	**11.60**	**-13.01**			**12.43**	**-11.91**	**12.40**	**-9.07**	**17.33**	**-14.57**
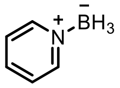 pyridine borane **B2**	18.26	-6.45			14.14	-17.35	13.83	-6.39	18.48	-20.02
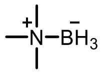 trimethylamine-borane **B3**	b.less	-29.00	**15.50**	**-17.40**	18.06	6.41	b.less	-25.99	23.68	3.74
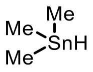 trimethylstannane **Sn_H**	**b.less**	**-20.14**			10.17	-15.64	10.93	-8.97	11.10	-18.31
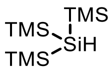 tris(trimethylsilyl)silane **Si_H**	**6.08**	**-18.16**			12.87	-10.29	11.95	-9.09	12.07	-12.95

[a] Values expressed in kcal·mol^{-1} have been reported. Columns have been indicated with different backgrounds to highlight the direct competition between **TS1** *vs* **TS4** and **TS3** *vs* **TS5**, respectively; data reported with a bold font have already been reported in a previous work by us;[7] b.less: barrierless.

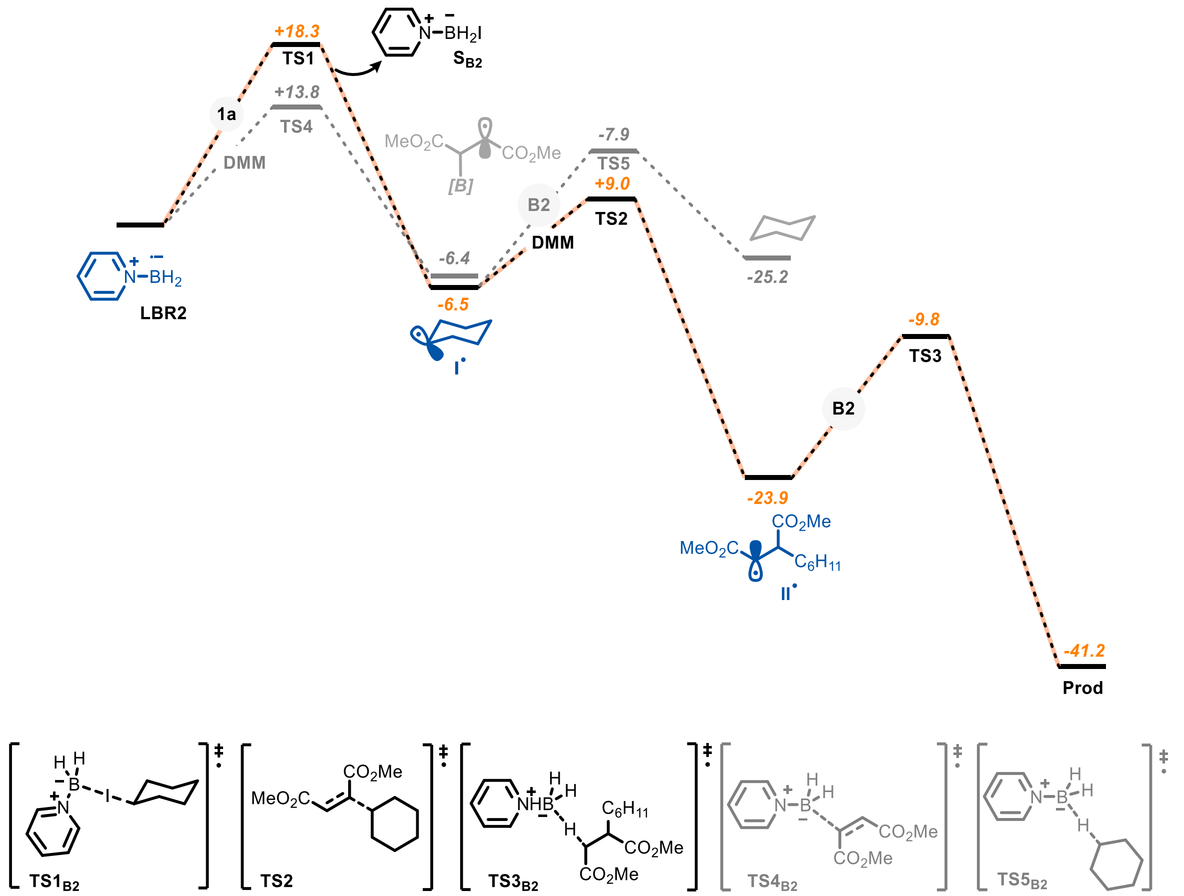

Scheme S3. Reaction profile for the reference reaction between **1a** and **DMM** in the presence of the chain carrier **B2**, as determined from ωB97xD/def2TZVP calculations in bulk acetonitrile.

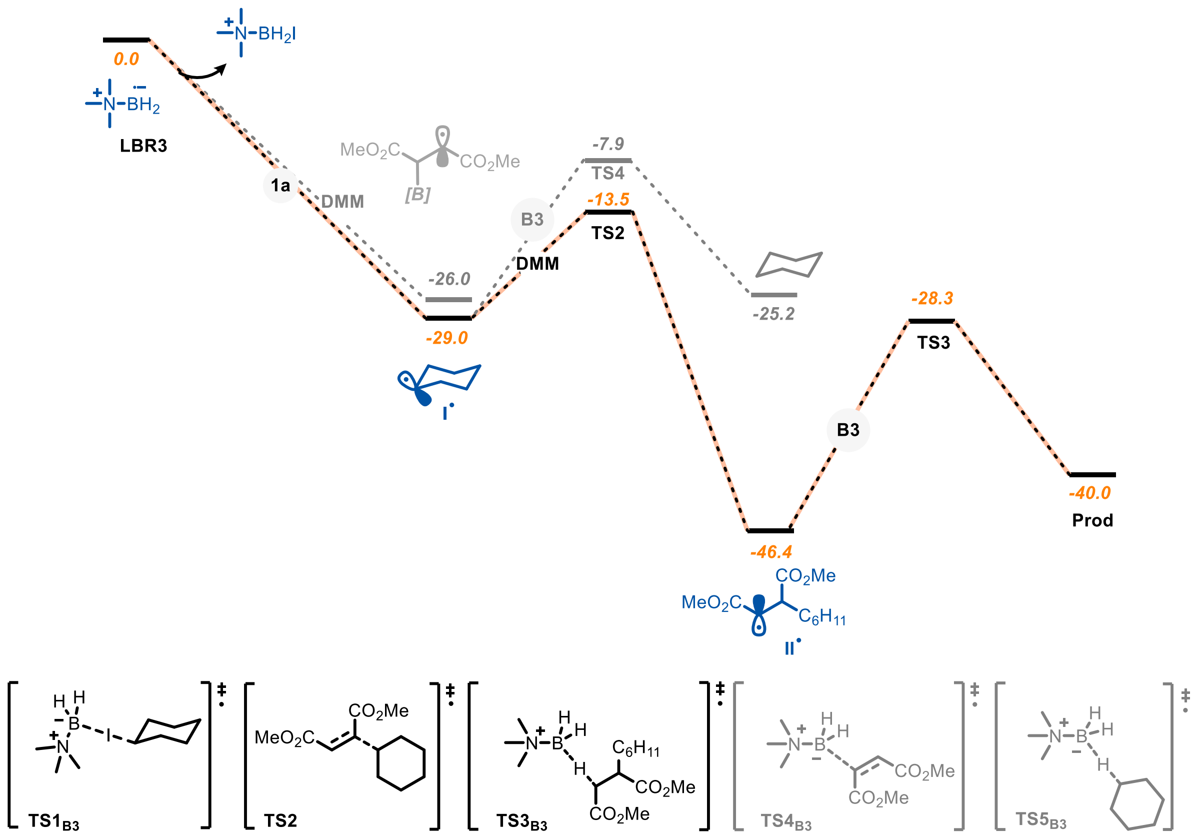

Scheme S4. Reaction profile for the reference reaction between **1a** and **DMM** in the presence of the chain carrier **B3**, as determined from ωB97xD/def2TZVP calculations in bulk acetonitrile.

8. Computational Details

All the calculations were carried out using the Gaussian 16 program package, revision C.01,[9] installed on Galileo100 at CINECA facility (Italy) and on the EOS cluster at the University of Pavia. Based on previous work,[7] in our investigation the level of theory chosen for the optimization of the reported stationary points was DFT (Density Functional Theory) by using the ωB97xD functional and the def2TZVP basis set in the gas phase. When appropriate, an unrestricted formalism (U prefix) has been adopted via the UωB97xD/def2TZVP keyword. No symmetry constraint was applied to the structures investigated and a thorough conformers search has been performed to locate the absolute minimum for each species. The structures and data used for this work correspond to those of the absolute minimum. Frequency calculations were performed in the gas phase to check that minima and transition states (TS) had 0 or 1 imaginary frequencies, respectively.

Solvent effect was included by single-point calculations at the same level of theory (ωB97xD/def2TZVP) adopting the standard implicit solvent model implemented in Gaussian 16 via the keyword SCRF=(SOLVENT=ACETONITRILE) on the optimized geometries obtained in vacuo.

The DFT Gibbs free energies reported in the main text have been calculated by means of Eq. S1 reported below:

$$G_{DFT} = E_{0(DFT,MeCN)} + \Delta G_{CORR(vacuo)} \qquad (S1)$$

Where:

- $E_{0(DFT,MeCN)}$ is the total electronic energy calculated at the SCRF-ωB97xD/def2TZVP level (acetonitrile bulk);
- $\Delta G_{CORR(vacuo)}$ is the unscaled thermal correction to Gibbs Free Energy as from the output of the frequency calculation in vacuo, also including the zero-point vibrational energy (ZPVE).

The two terms from Eq. S1 have been reported in bold for all the stationary points reported below.

As for TSs, Intrinsic Reaction Coordinate (IRC) calculations were performed in both directions (40 steps each) at the same level of theory adopted for optimizations (ωB97xD/def2TZVP in the gas phase) in order to investigate the process in detail and to confirm the nature of the TS itself. The "LQA" option for the IRC keyword has been consistently specified, in order to adopt the local quadratic approximation for the predictor step.

When structures not corresponding to stationary points have been considered (e.g. when stretching a bond or along IRC), energies have been expressed simply by considering the first term of Eq. S1, that is $E_{0(DFT,MeCN)}$, since frequency calculations (needed for determining $\Delta G_{CORR(vacuo)}$) were carried out exclusively on stationary points.

Optimized geometry listed in cartesian format (coordinates are given in Å), minimum energies and thermochemical data (in Hartree; the default options were adopted in the latter case, *viz.* temperature: 298.150 K and pressure: 1.00000 atm) are reported below.

The conversion factor adopted between Hartree and kcal mol^{-1} is: 1 Hartree = 627.509 kcal mol^{-1}.

It is important to stress that some of the structures reported here have already been reported by us in a previous work.[7] For the sake of completeness, such structures have been reported here as well, clearly indicating that the very same structure can be likewise found elsewhere.

Modeling of the reference reaction

SPECIES	G_{DFT} [Hartree]	SPECIES	G_{DFT} [Hartree]
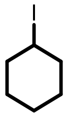 **1a**	-532.956177	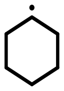 **I•**	-235.098148
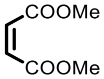 **DMM**	-534.287989	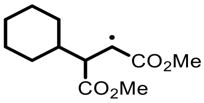 **II•**	-769.413862
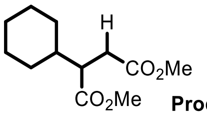 **Prod**	-770.062835	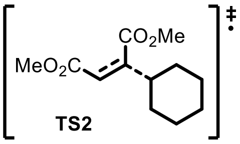 **TS2**	-769.361443
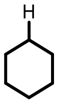 **1-Red**	-235.751370		

Table S6. Gibbs free energies (shown in Hartree) of the species optimized to model the reference reaction between **1a** and **DMM** to deliver **Prod** adopted in our computational study, as determined from ωB97xD/def2TZVP calculations in bulk acetonitrile. All the species gathered in this table have been already reported in a previous work by us.[7]

Use of ligated borane B1

SPECIES	GDFT [Hartree]	SPECIES	GDFT [Hartree]
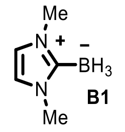	-331.398913	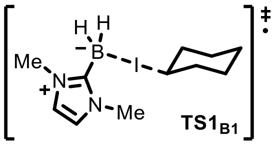	-863.706597
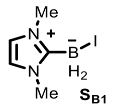	-628.647677	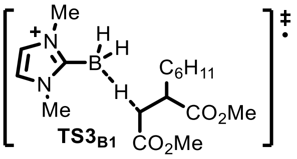	-1100.792971
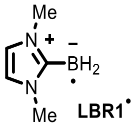	-330.768912	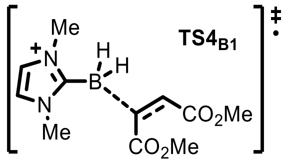	-865.037139
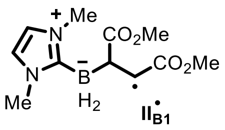	-865.071356	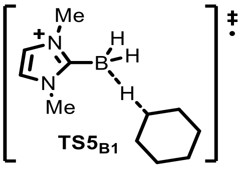	-566.469443

Table S7. Gibbs free energies (shown in Hartree) of the species optimized to model the behaviour of ligated borane **B1**, as determined from ωB97xD/def2TZVP calculations in bulk acetonitrile. All the species gathered in this table have been already reported in a previous work by us.[7]

Use of ligated borane B2

SPECIES	G_{DFT} [Hartree]	SPECIES	G_{DFT} [Hartree]
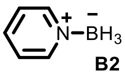 B2	-274.869135	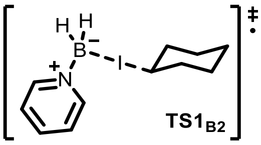 $TS1_{B2}$	-807.174884
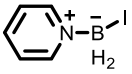 S_{B2}	-572.116124	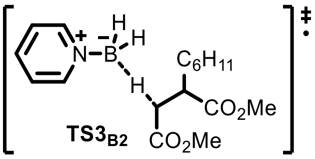 $TS3_{B2}$	-1044.260469
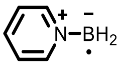 LBR2•	-274.247812	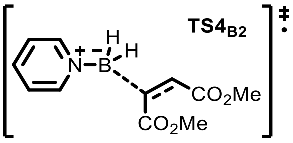 $TS4_{B2}$	-808.513766
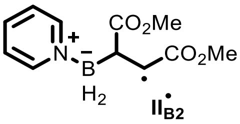 $II^{•}_{B2}$	-808.545979	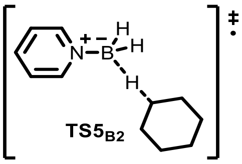 $TS5_{B2}$	-509.937835

Table S8. Gibbs free energies (shown in Hartree) of the species optimized to model the behaviour of ligated borane **B2**, as determined from ωB97xD/def2TZVP calculations in bulk acetonitrile.

Use of ligated borane B3

SPECIES	G_{DFT} [Hartree]	SPECIES	G_{DFT} [Hartree]
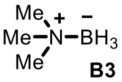 **B3**	-201.037674	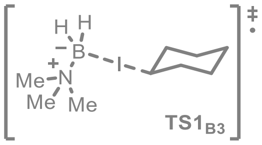 **TS1$_{B3}$**	Structure not located (see section 8.2)
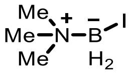 **S$_{B3}$**	-498.282734	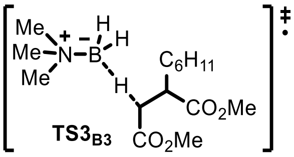 **TS3$_{B3}$**	-970.422753
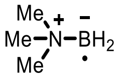 **LBR3$^•$**	-200.378484	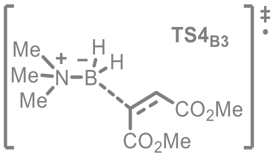 **TS4$_{B3}$**	Structure not located (see section 8.2)
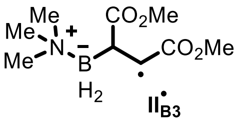 **II$^•_{B3}$**	-734.707886	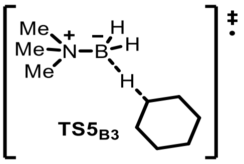 **TS5$_{B3}$**	-436.098081

Table S9. Gibbs free energies (shown in Hartree) of the species optimized to model the behaviour of ligated borane **B3**, as determined from ωB97xD/def2TZVP calculations in bulk acetonitrile.

Use of the Sn-based chain carrier $Me_3Sn^{\bullet}$

SPECIES	G_{DFT} [Hartree]	SPECIES	G_{DFT} [Hartree]
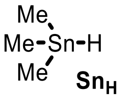	-334.633996	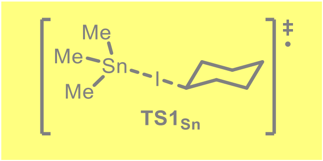	Structure not located (see section 8.2)
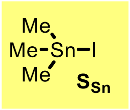	-631.900065	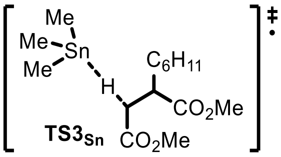	-1104.031654
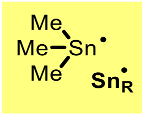	-334.009945	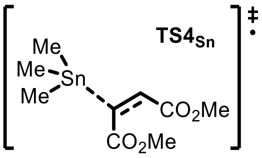	-868.280510 [a]
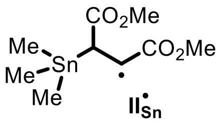	-868.312224	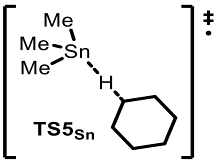	-569.714462

[a] Due to the marked tendency of $Me_3Sn^{\bullet}$ to form Sn–O bonds, this TS structure has been optimized by reducing the maximum size for an optimization step via the keyword "OPT=(MAXSTEP=5)".

Table S10. Gibbs free energies (shown in Hartree) of the species optimized to model the behaviour of $Me_3Sn^{\bullet}$ (**$Sn_R^{\bullet}$**) in the role of chain carrier, as determined from ωB97xD/def2TZVP calculations in bulk acetonitrile. Structures/situations highlighted with a yellow background have been already reported in a previous work by us.[7]

Use of the Si-based chain carrier $(Me_3Si)_3Si^{\bullet}$

SPECIES	G_{DFT} [Hartree]	SPECIES	G_{DFT} [Hartree]
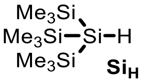 **Si_H**	-1517.702094	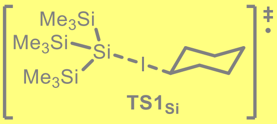 **$TS1_{Si}$**	-2050.015998
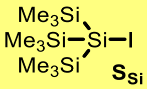 **S_{Si}**	-1814.956484	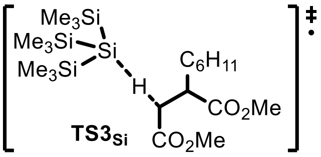 **$TS3_{Si}$**	-2287.095443
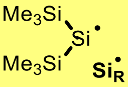 **$Si_R^{\bullet}$**	-1517.069513	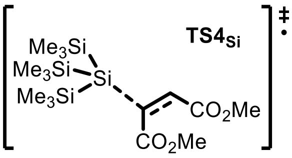 **$TS4_{Si}$**	-2051.338454
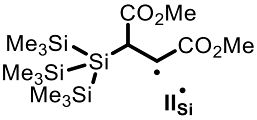 **$II_{Si}^{\bullet}$**	-2051.371987	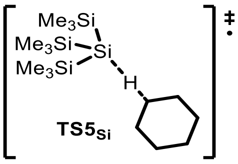 **$TS5_{Si}$**	-1752.781012

Table S11. Gibbs free energies (shown in Hartree) of the species optimized to model the behaviour of $(Me_3Si)_3Si^{\bullet}$ (**$Si_R^{\bullet}$**) in the role of chain carrier, as determined from ωB97xD/def2TZVP calculations in bulk acetonitrile. Structures highlighted with a yellow background have been already reported in a previous work by us.[7]

8.1. IRC Plots

The IRC analysis of the TS structures already published by us can be found in the corresponding literature reference and will not be duplicated here.[7]

TS1$_{B2}$

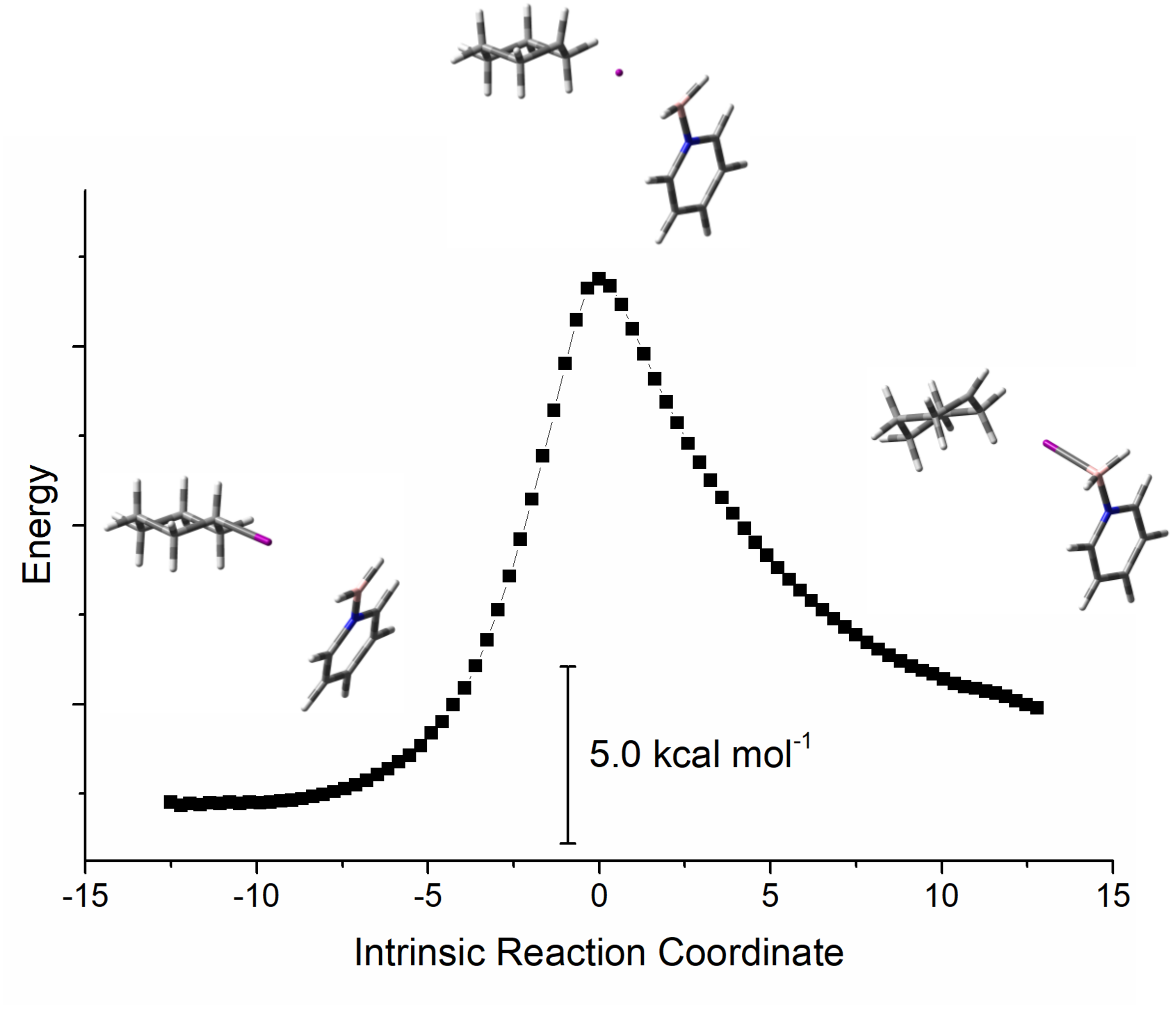

TS3$_{B2}$

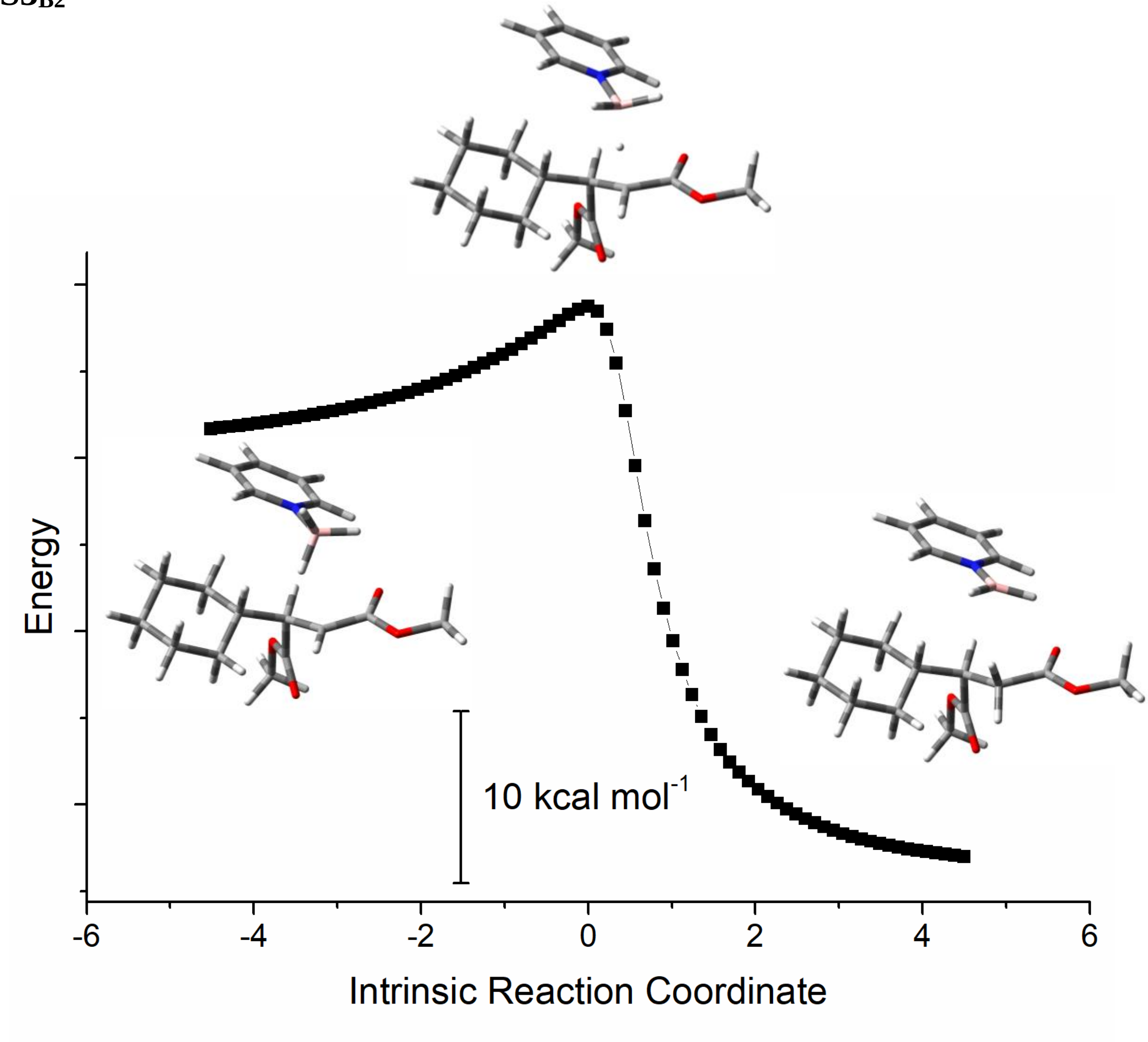

Energy
10 kcal mol-1
-6
-4
-2
0
2
4
6
Intrinsic Reaction Coordinate

TS4$_{B2}$

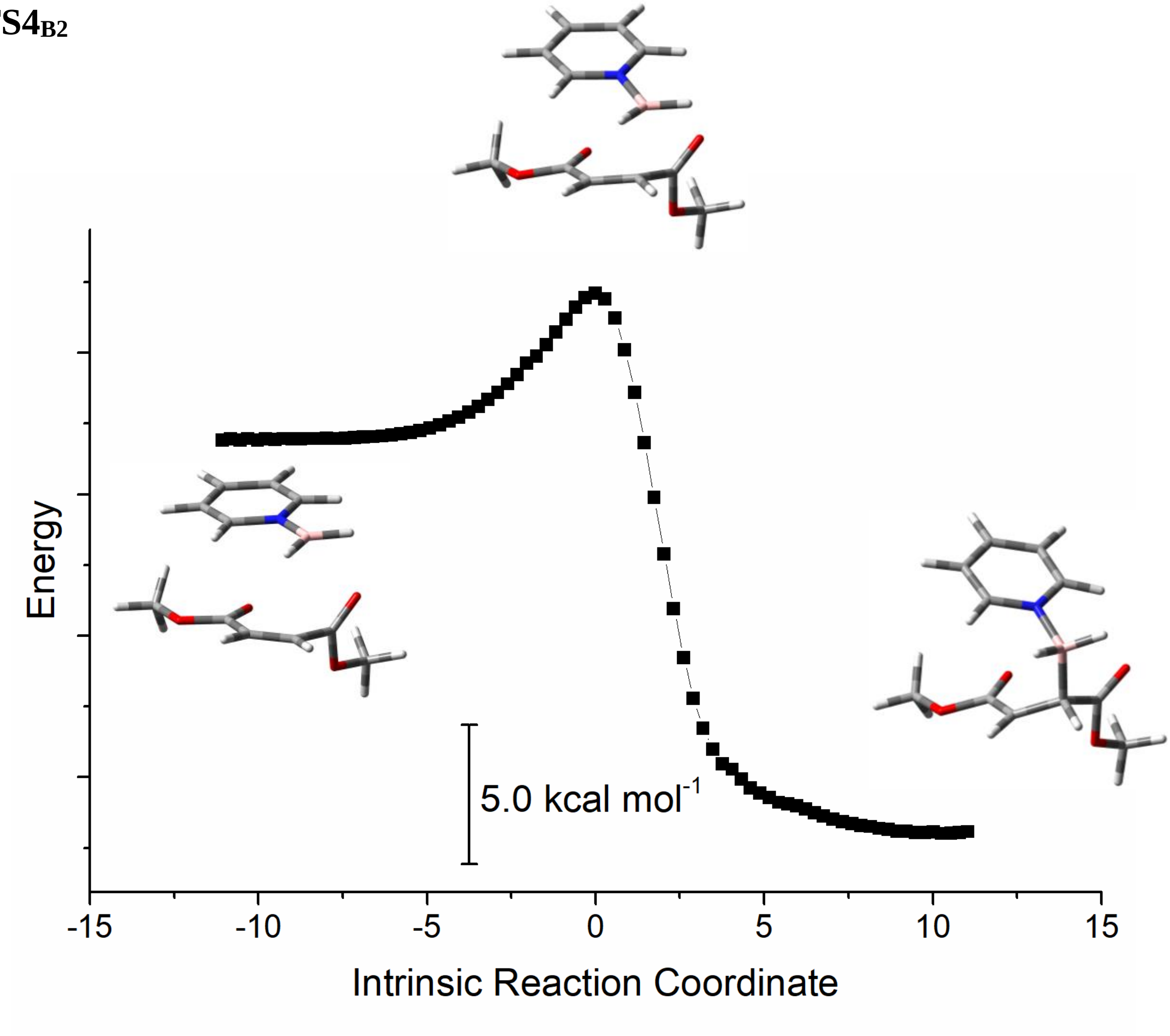

Energy
5.0 kcal mol-1
-15
-10
-5
0
5
10
15
Intrinsic Reaction Coordinate

TS5$_{B2}$

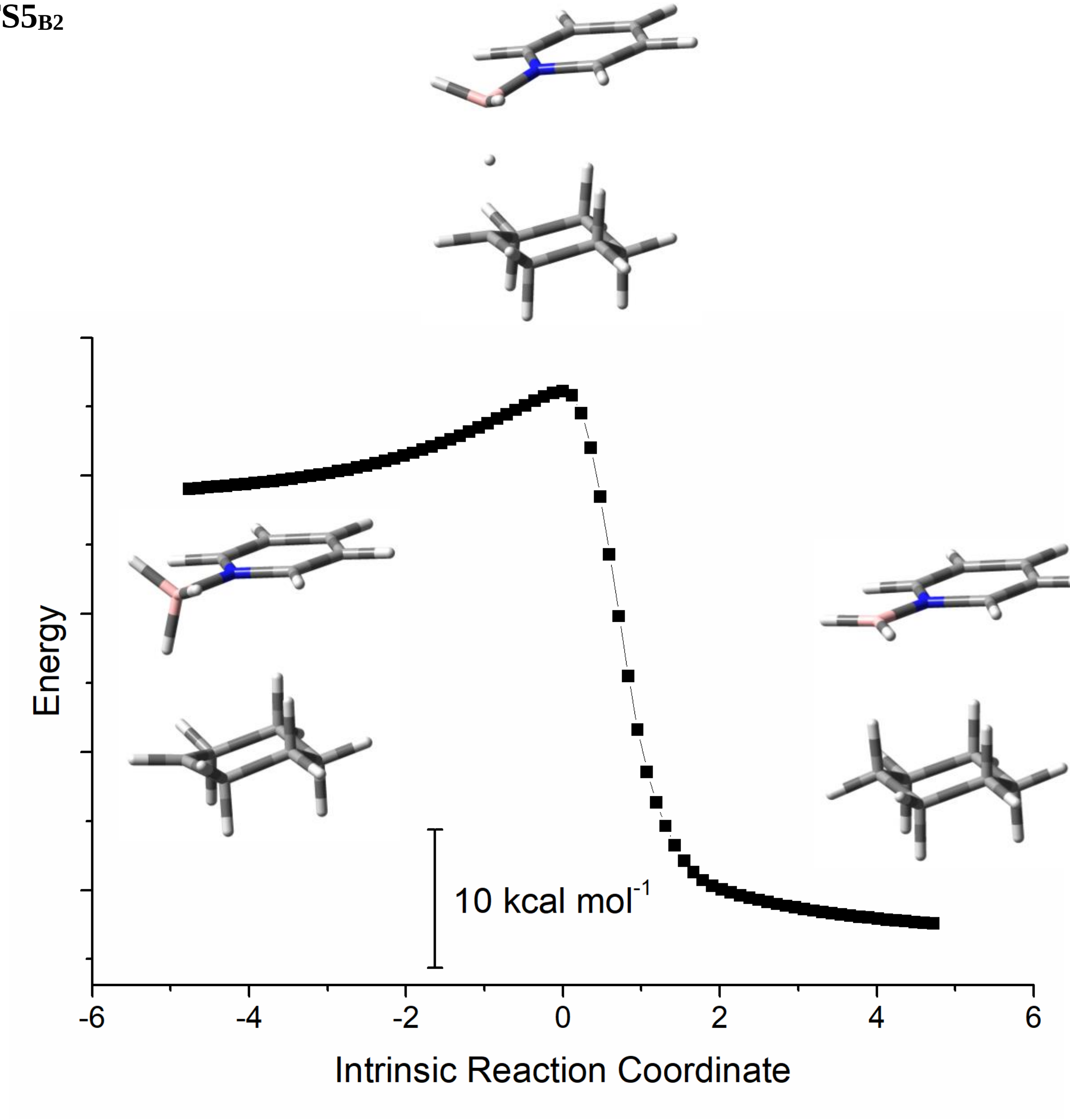

Figure S8. IRC plots of the transition states **TS1,3,4,5** describing the behaviour of ligated borane **B2** and adopted to trace the reaction profile reported in **Scheme S3**, as from calculations at the ωB97xD/def2TZVP level of theory in the gas phase (total electronic energy values have been reported). The three structures reported in each graph refer, respectively, to those of the first point (left), the transition state (center) and the last point (right) along the reaction coordinate.

TS3$_{B3}$

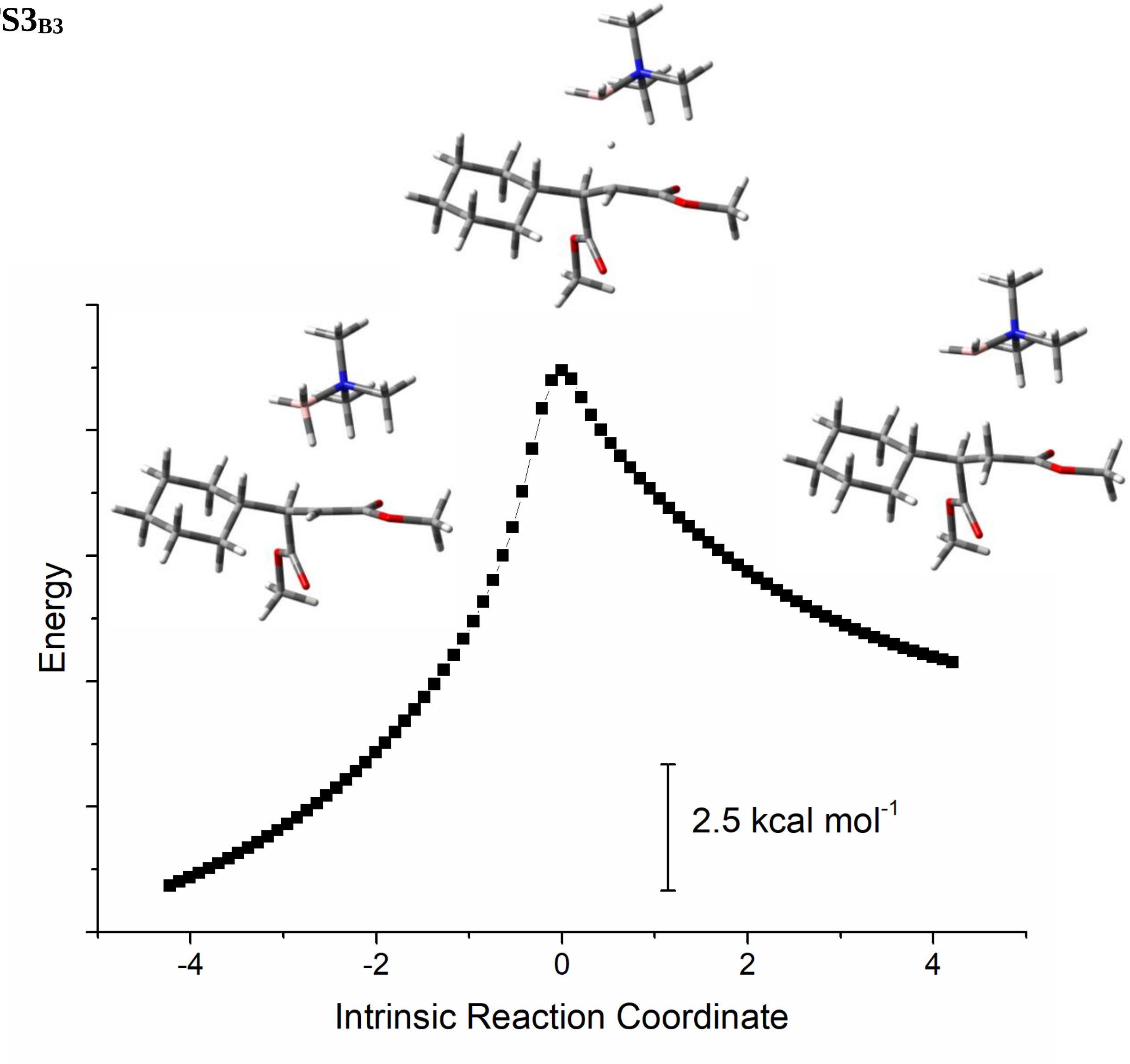

TS5$_{B3}$

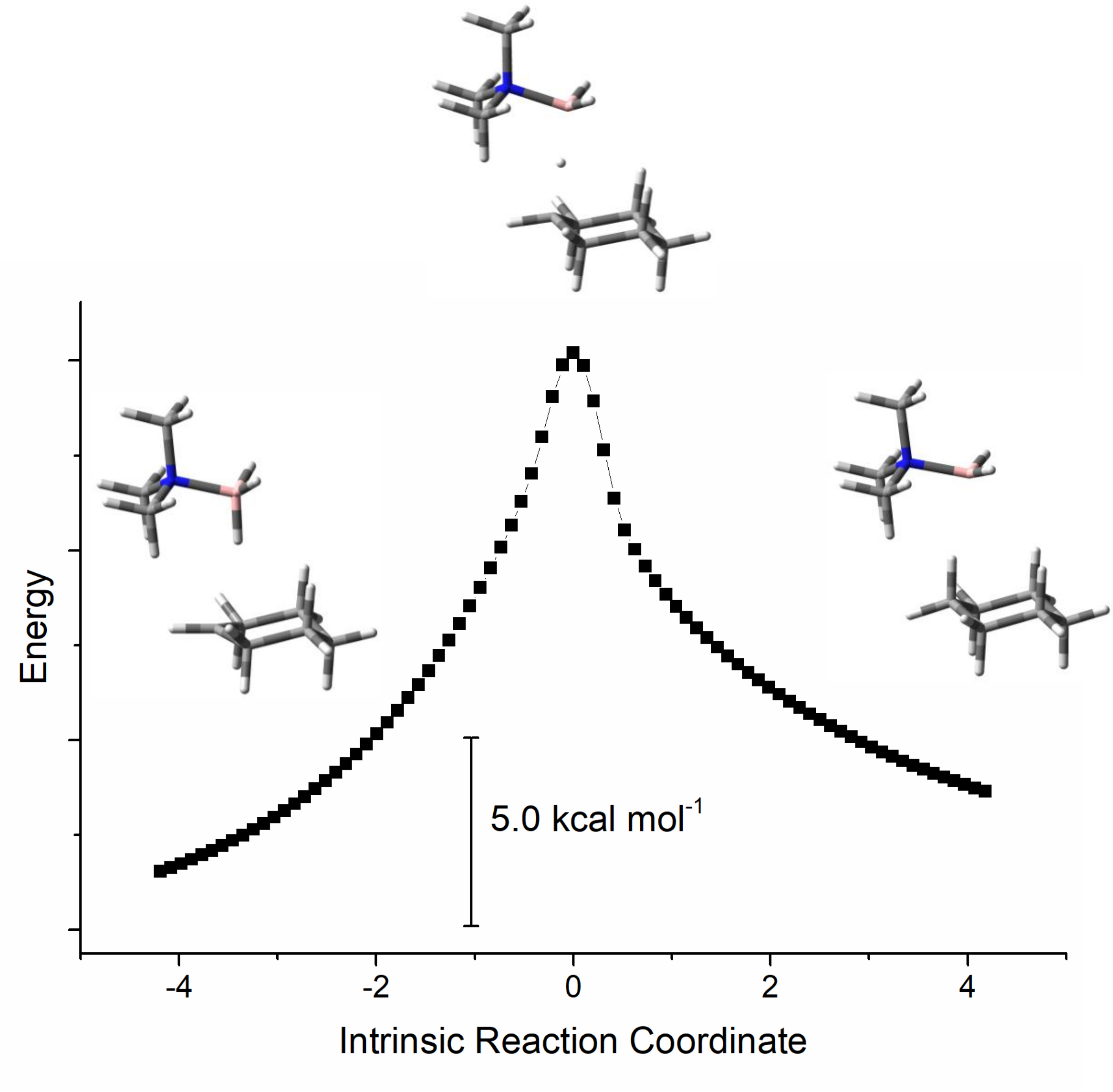

Figure S9. IRC plots of the transition states **TS3,5** describing the behaviour of ligated borane **B3** and adopted to trace the reaction profile reported in **Scheme S4**, as from calculations at the ωB97xD/def2TZVP level of theory in the gas phase (total electronic energy values have been reported). The three structures reported in each graph refer, respectively, to those of the first point (left), the transition state (center) and the last point (right) along the reaction coordinate.

TS3$_{Sn}$

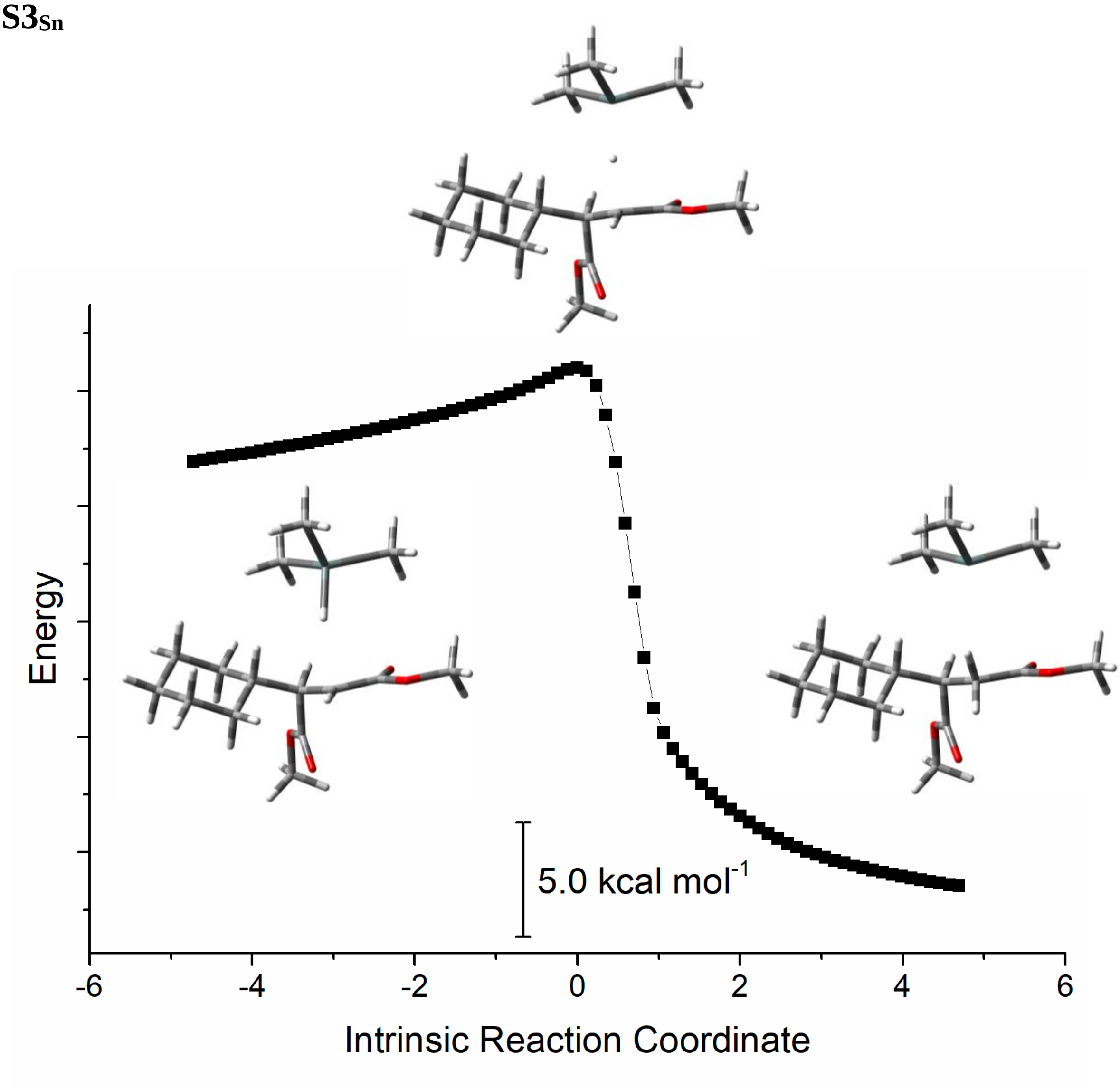

Energy
5.0 kcal mol-1
-6
-4
-2
0
2
4
6
Intrinsic Reaction Coordinate

TS4$_{Sn}$

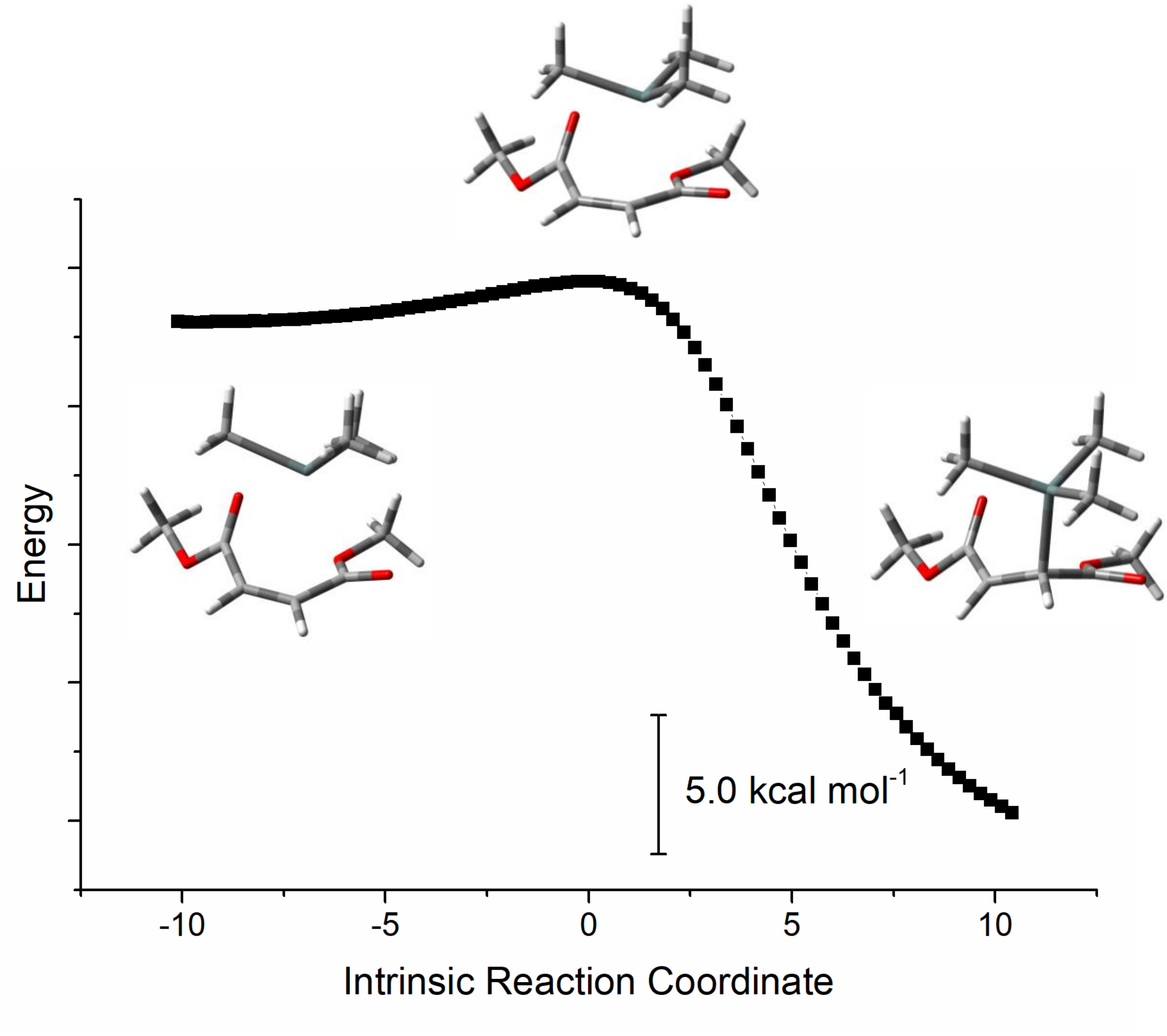

Energy
5.0 kcal mol-1
-10
-5
0
5
10
Intrinsic Reaction Coordinate

$TS5_{Sn}$

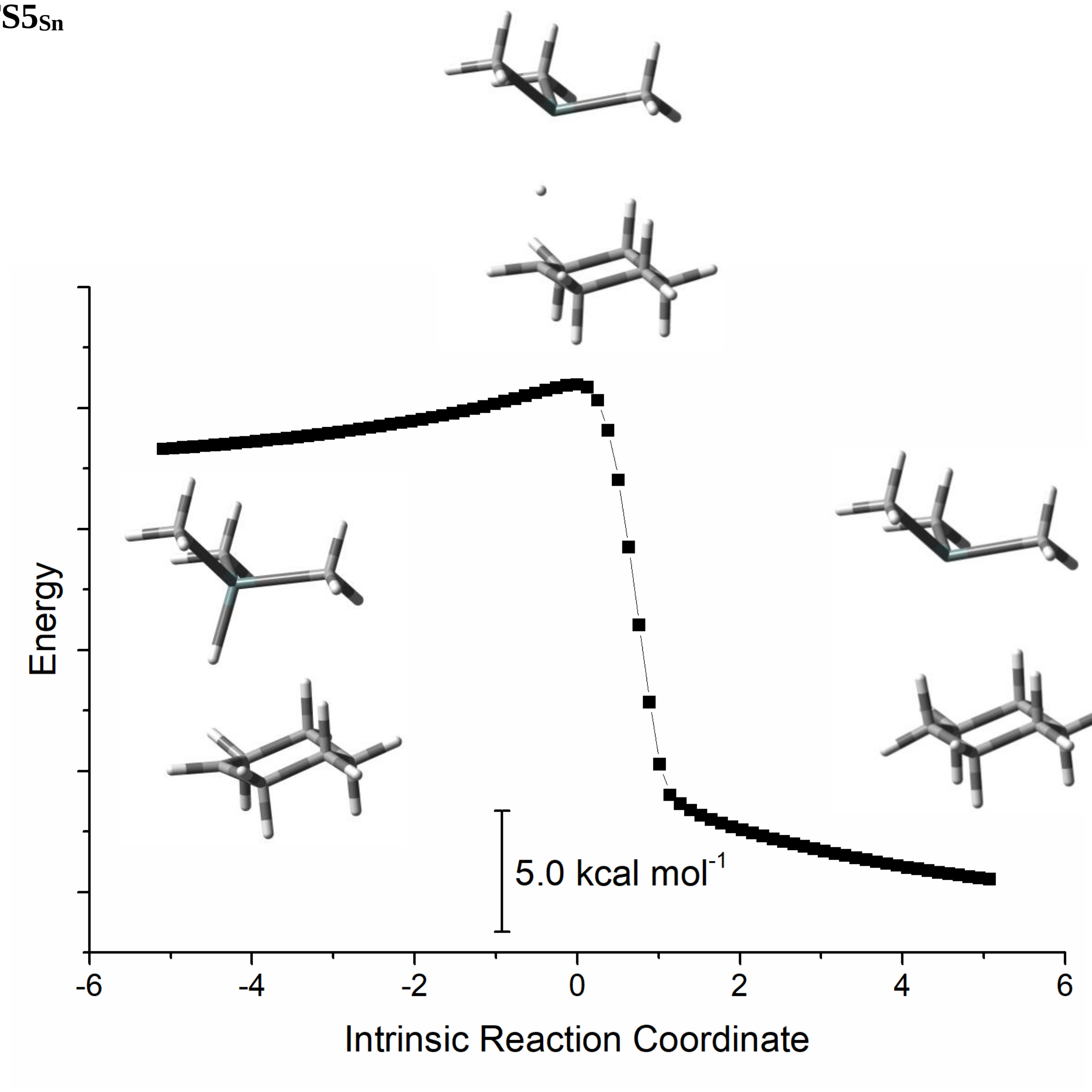

Figure S10. IRC plots of the transition states **TS3,4,5** describing the behaviour of trimethylstannane (Me_3Sn-H; **Sn_H**), as from calculations at the ωB97xD/def2TZVP level of theory in the gas phase (total electronic energy values have been reported). The three structures reported in each graph refer, respectively, to those of the first point (left), the transition state (center) and the last point (right) along the reaction coordinate.

TS3$_{Si}$

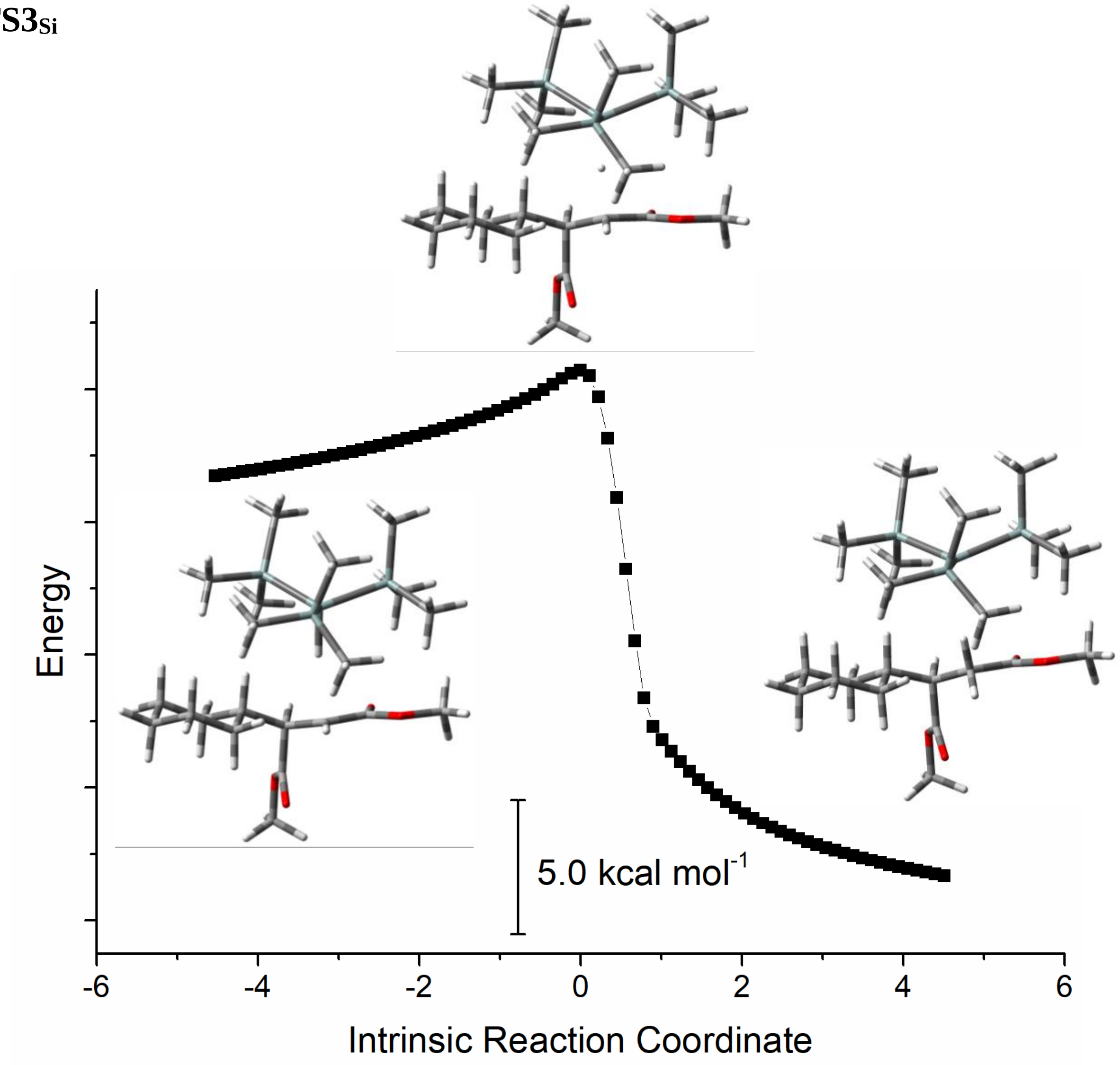

Energy
5.0 kcal mol-1
-6
-4
-2
0
2
4
6
Intrinsic Reaction Coordinate

TS4$_{Si}$

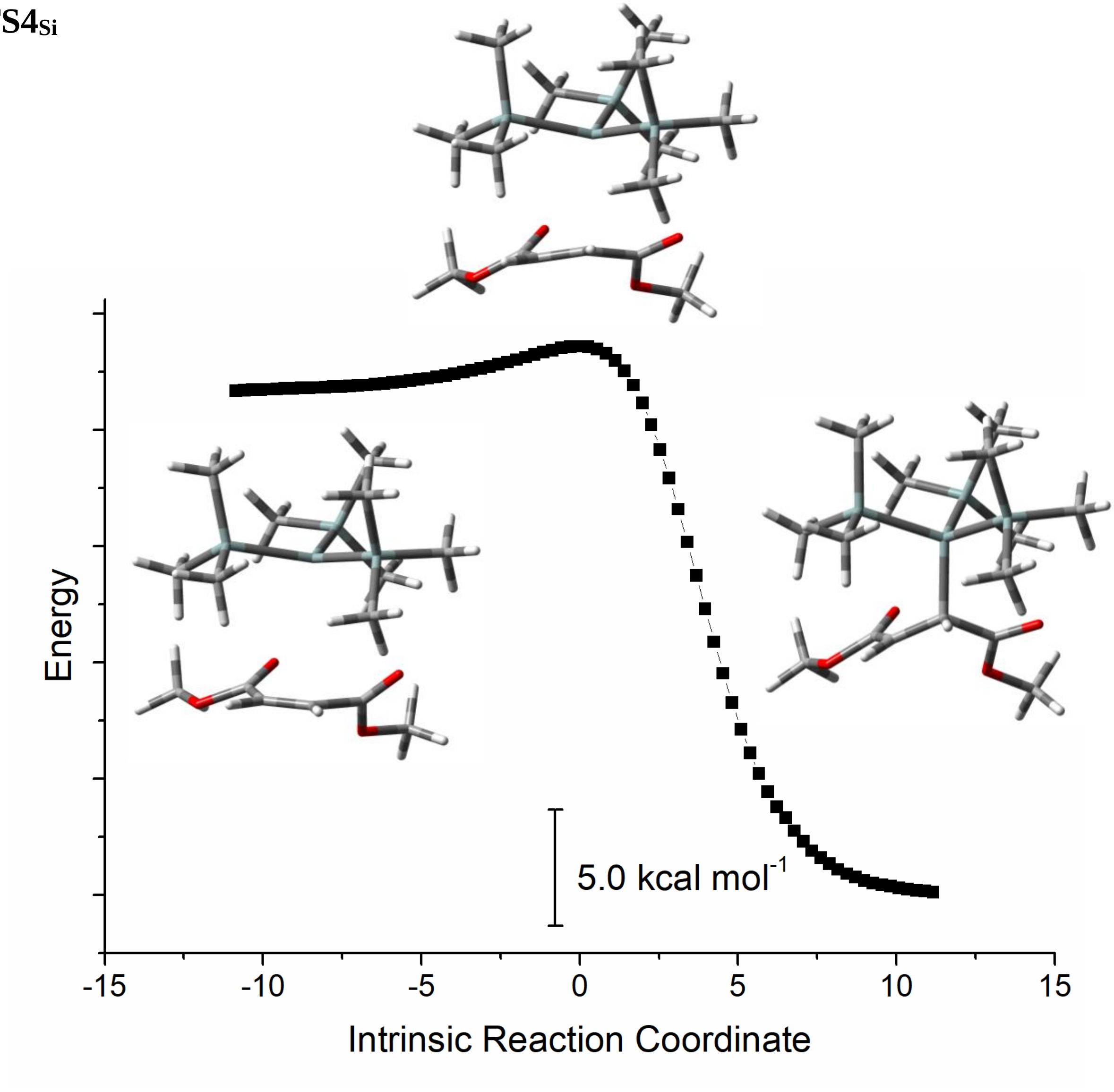

Energy
5.0 kcal mol-1
-15
-10
-5
0
5
10
15
Intrinsic Reaction Coordinate

TS5$_{Si}$

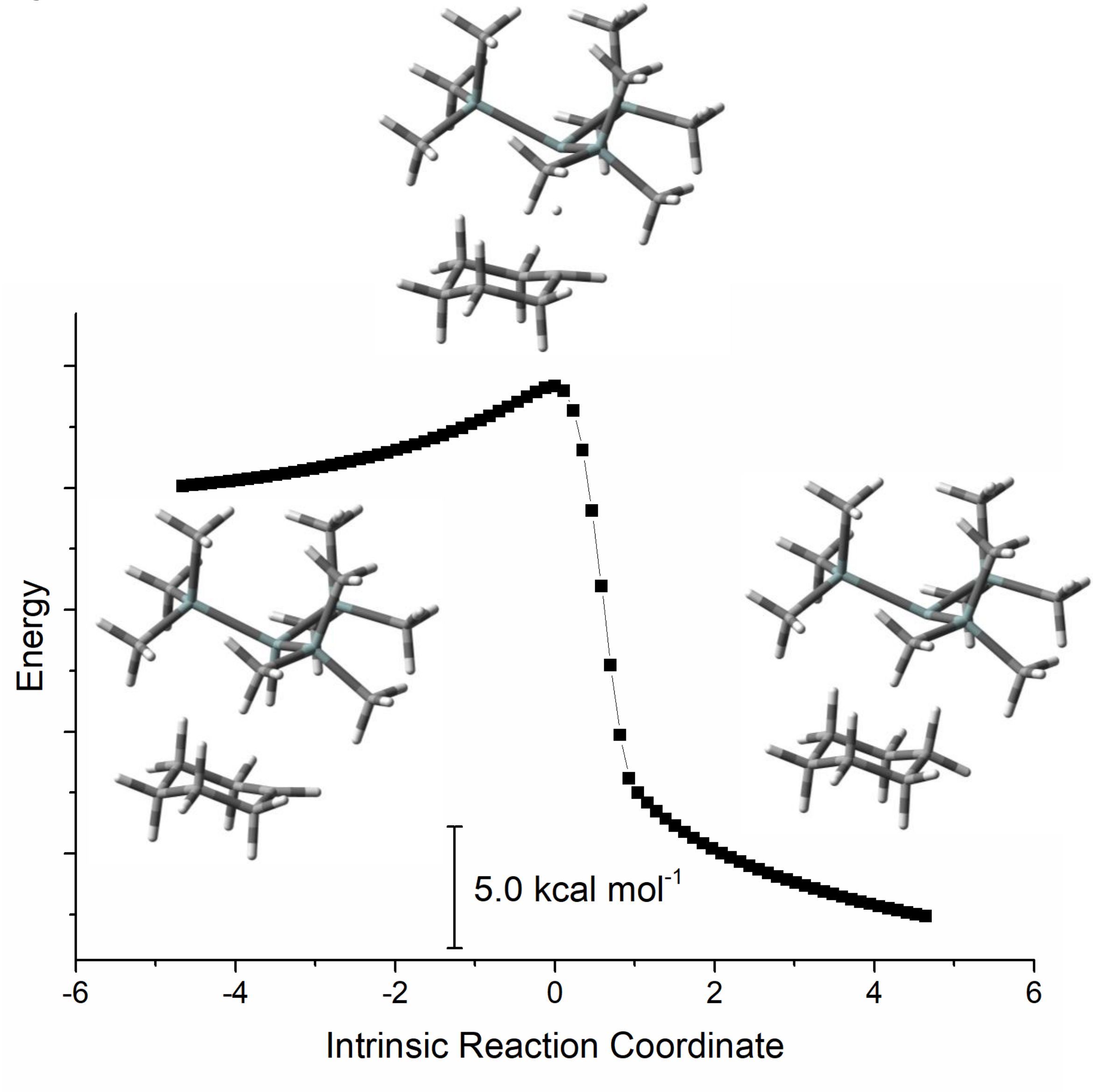

Figure S11 IRC plots of the transition states **TS3,4,5** describing the behaviour of tris(trimethylsilyl)silane ((TMS)$_3$Si-H; **Si$_H$**), as from calculations at the ωB97xD/def2TZVP level of theory in the gas phase (total electronic energy values have been reported). The three structures reported in each graph refer, respectively, to those of the first point (left), the transition state (center) and the last point (right) along the reaction coordinate.

8.2. Relaxed PES Scan

In a few instances, all the attempts to locate a transition state along the reaction coordinate failed, specifically when describing the XAT step from cyclohexyl iodide (**1a**) by **LBR3•** and $Me_3Sn^{\bullet}$ (**Sn$_R$•**) as the halogen abstractor failed. The latter situation has been already fully analyzed and described by us in a previous work.[7] In addition, no TS was located for the radical addition of **LBR3•** onto **DMM** to afford radical adduct $\mathbf{II^{\bullet}_{B3}}$. In all such instances, we performed a relaxed potential energy surface (PES) scan to actually confirm the nature of the considered reaction step, via the adoption of the "OPT = MODREDUNDANT" approach.

- XAT step by **LBR3•** from **1a**

As for the XAT step by **LBR3•**, we elongated the C–I bond in the model substrate **1a** in the presence of named abstractor. Thus, we froze the C–I bond at the equilibrium length found in **1a** alone (2.16 Å) and then elongated it with a 0.1 Å step size until bond length > 3.5 Å (14 steps). As apparent from **Figure S12**, the total electronic energy values follow a decreasing profile upon C–I elongation (black symbols, left axis). In the same graphic, the B–I bond length (not involved in any constraints) is also plotted, showing that this value decreases finally reaching a plateau (red symbols, right axis), which corresponds to the same B–I bond length found in the Me_3N-BH_2–I species alone (2.26Å).

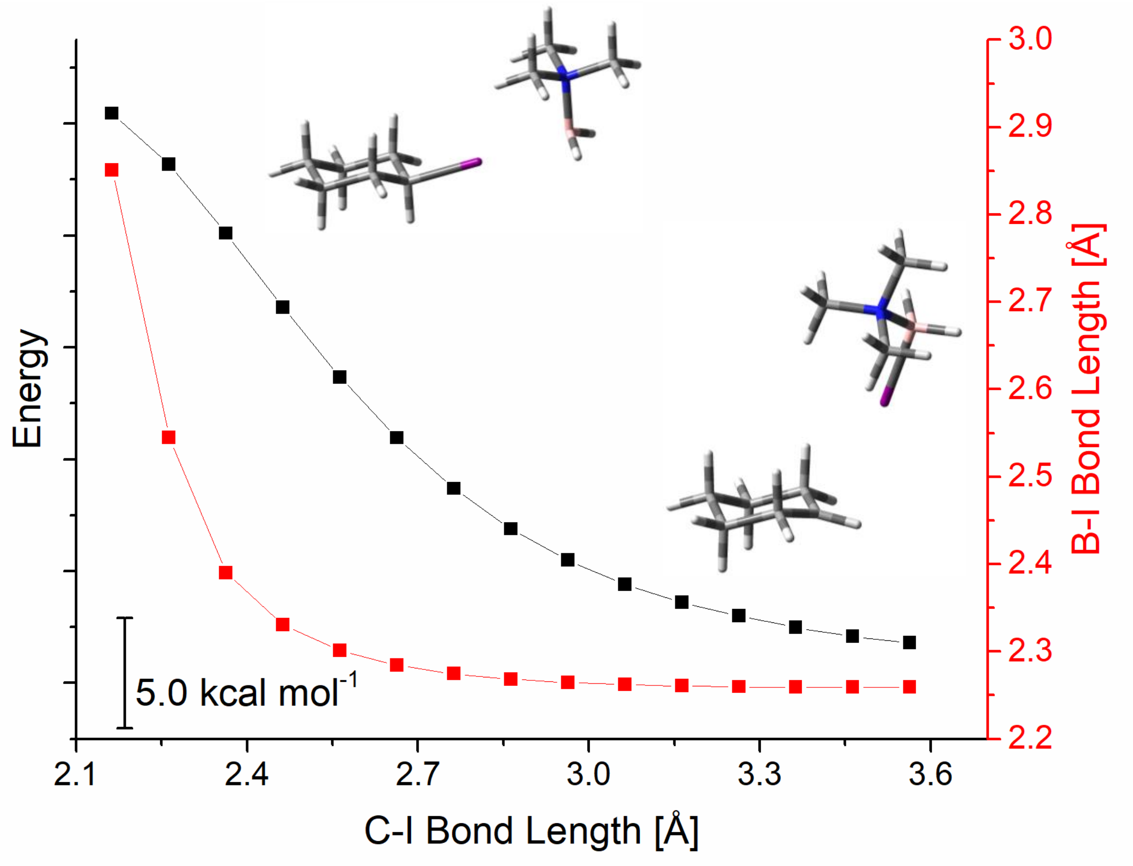

Figure S12. Relaxed PES scan describing the XAT step in cyclohexyl iodide (**1a**) promoted by the abstractor **LBR3•** (see **Table S5** and **Table S9**), as from calculations at the ωB97xD/def2TZVP level of theory in the gas phase (total electronic energy values have been reported). The two structures reported in the graph refer, respectively, to those of the first (left) and last (right) points of the scan.

- Radical addition step of **LBR3•** onto **DMM**

As for the radical addition step of **LBR3•** onto **DMM**, we started from the structure of radical adduct $\mathbf{II}^{\bullet}_{\mathbf{B3}}$ and elongated the C–B bond from the equilibrium length (1.66 Å) with a 0.1 Å step size until bond length > 3.0 Å (14 steps). As apparent from **Figure S13**, the total electronic energy values follow an increasing profile upon C–B bond elongation.

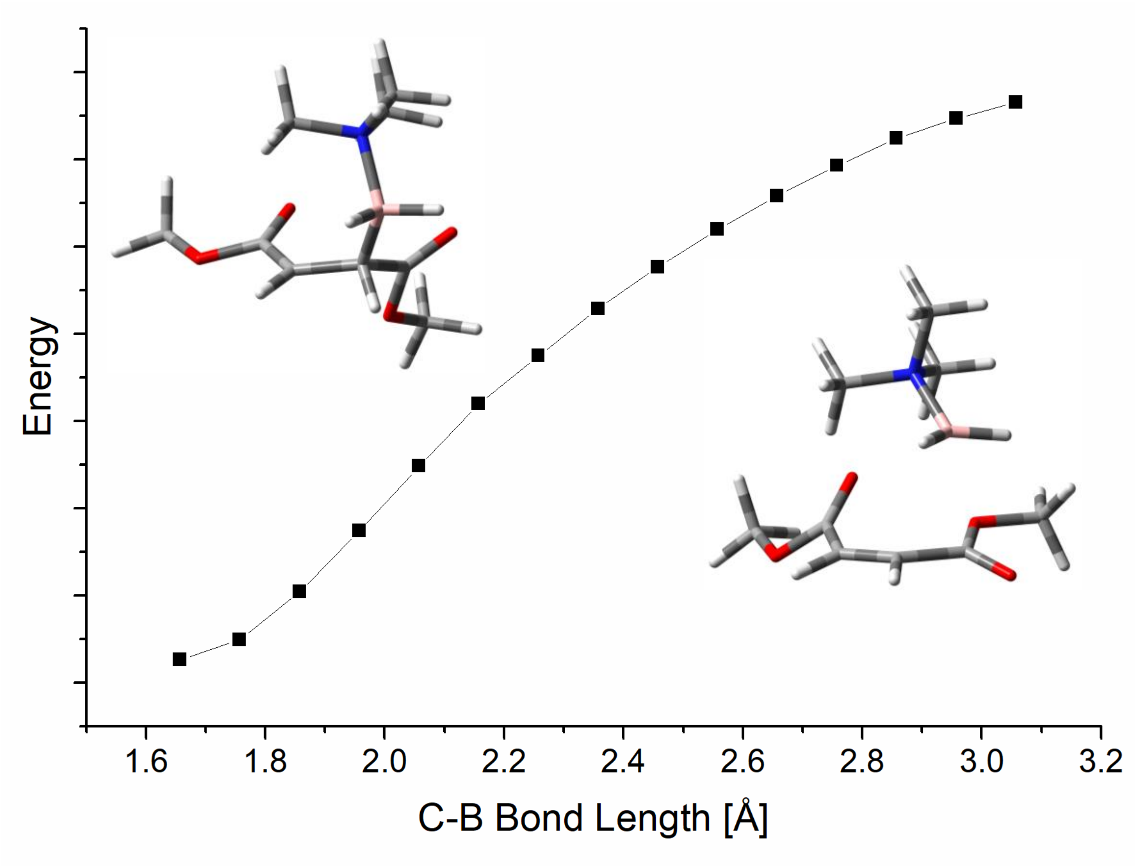

Figure S13. Relaxed PES scan describing the radical addition step of **LBR3•** onto **DMM** (see **Table S5** and **Table S9**), as from calculations at the ωB97xD/def2TZVP level of theory in the gas phase (total electronic energy values have been reported). The two structures reported in the graph refer, respectively, to those of the first (left) and last (right) points of the scan.

8.3. Optimized Structures

All the structures reported below and indicated with a * symbol have already been reported by us in a previous work.[7]

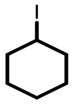

1a *

C	2.59021200	-1.25759000	0.18929600
C	1.09405300	-1.26191200	-0.13397300
C	0.43757500	0.00000000	0.40512400
C	1.09405300	1.26191200	-0.13397400
C	2.59021100	1.25759100	0.18929800
C	3.27269100	0.00000000	-0.33980000
H	0.95464900	-1.30377800	-1.21858300
H	0.61626500	-2.14827200	0.28534500
H	2.72534600	-1.31395200	1.27489200
H	3.05450500	-2.15206200	-0.23105100
H	0.95465200	1.30377400	-1.21858500
H	0.61626200	2.14827300	0.28533900
H	3.05450400	2.15206400	-0.23104600
H	2.72534300	1.31395000	1.27489400
H	3.22926500	0.00000100	-1.43435400
H	4.32988700	-0.00000100	-0.06638500
H	0.46873400	0.00000100	1.49503800
I	-1.68306000	0.00000000	-0.02398700

E(UwB97XD, vacuo)	-533.081999
Zero-point correction=	0.161571
Thermal correction to Energy=	0.168598
Thermal correction to Enthalpy=	0.169542
Thermal correction to Gibbs Free Energy=	**0.128715**
E(UwB97XD, MeCN)	**-533.084892**

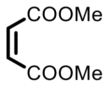

DMM *

C	0.66278500	-1.55002100	-0.30836200
C	-0.65369300	-1.41160300	-0.36207400
H	1.11638600	-2.50056500	-0.56478400
H	-1.28276300	-2.23309300	-0.67778000
C	1.62452100	-0.50736700	0.16763500
O	2.14315300	-0.55055400	1.24765300
O	1.88150900	0.39836800	-0.77005700
C	-1.33894600	-0.15341600	0.01739200
O	-0.80661100	0.82041200	0.48041900
O	-2.65268300	-0.24264200	-0.21663700
C	-3.41973100	0.91071300	0.11902800

H	-3.32328400	1.13775100	1.18062200
H	-3.08685000	1.77251500	-0.45928300
H	-4.44887400	0.66298200	-0.12567800
C	2.73714400	1.46877600	-0.37185000
H	2.27556000	2.02735600	0.44193400
H	3.70490600	1.08800900	-0.04629500
H	2.84949800	2.09788000	-1.25036900

E(UwB97XD, vacuo)	-534.379468
Zero-point correction=	0.139721
Thermal correction to Energy=	0.150563
Thermal correction to Enthalpy=	0.151507
Thermal correction to Gibbs Free Energy=	**0.101849**
E(UwB97XD, MeCN)	**-534.389838**

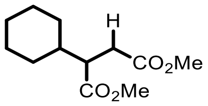

Prod *

C	-1.20126400	-1.07618700	0.00229900
C	-0.16232300	-0.06758100	-0.46796500
H	-1.11649300	-1.25267700	1.07546400
C	-2.61446700	-0.62694100	-0.26685000
O	-2.92985700	0.41528800	-0.77506300
O	-3.49626300	-1.54726000	0.13767300
C	-0.27310400	1.19638400	0.36199900
O	-0.52167200	1.22348900	1.53914600
O	-0.03526600	2.29200600	-0.36222000
C	-0.11337800	3.52835800	0.33982400
H	-1.11058900	3.66521500	0.75732500
H	0.61724600	3.55959300	1.14848100
H	0.09976400	4.29992000	-0.39510700
C	-4.86972300	-1.21707200	-0.05006300
H	-5.12442100	-0.31226600	0.50156600
H	-5.08652200	-1.06033700	-1.10673700
H	-5.43261400	-2.06484400	0.33112900
C	1.26808200	-0.65459000	-0.43615200
C	2.29367300	0.28090700	-1.08076700
C	1.74281400	-1.08115200	0.95542700
H	1.22147900	-1.55853600	-1.05791500
C	3.67590100	-0.36171700	-1.14560300
H	2.35787900	1.20611500	-0.49921300
H	1.95787500	0.56841300	-2.08019500
C	3.12935200	-1.71863300	0.89936900
H	1.76805100	-0.20674400	1.61319600
H	1.03695300	-1.78301100	1.40380800
C	4.14811000	-0.79559500	0.23859300
H	4.39000600	0.33470600	-1.59062000
H	3.63806800	-1.23646900	-1.80460400
H	3.45517700	-1.98440000	1.90736800
H	3.07234800	-2.65440800	0.33166900
H	5.12023600	-1.28926100	0.17169800

H	4.28741800	0.09264300	0.86481400
H	-0.37963000	0.21116600	-1.50005600
H	-1.04980900	-2.04008900	-0.48903400

E(UwB97XD, vacuo)	-770.322039
Zero-point correction=	0.315646
Thermal correction to Energy=	0.332740
Thermal correction to Enthalpy=	0.333684
Thermal correction to Gibbs Free Energy=	**0.268986**
E(UwB97XD, MeCN)	**-770.331821**

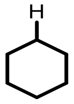

1-Red *

C	1.37647000	-0.47626300	-0.22954200
C	1.10069200	0.95382600	0.22958400
C	-0.27572200	1.43007600	-0.22963100
C	-1.37645100	0.47628300	0.22958800
C	-1.10071600	-0.95381100	-0.22954800
C	0.27572100	-1.43008500	0.22953700
H	1.14664800	0.99375300	1.32390400
H	1.87722300	1.62693700	-0.14130800
H	1.43414000	-0.49615900	-1.32385400
H	2.34759100	-0.81230000	0.14147000
H	-1.43398300	0.49621500	1.32390800
H	-2.34759400	0.81234000	-0.14133200
H	-1.87723800	-1.62690100	0.14141800
H	-1.14680200	-0.99374100	-1.32386000
H	0.28729600	-1.48994200	1.32383900
H	0.47029400	-2.43911700	-0.14146000
H	-0.47029500	2.43911000	0.14127000
H	-0.28724400	1.48964900	-1.32392600

E(UwB97XD, vacuo)	-235.893931
Zero-point correction=	0.171535
Thermal correction to Energy=	0.177179
Thermal correction to Enthalpy=	0.178123
Thermal correction to Gibbs Free Energy=	**0.142822**
E(UwB97XD, MeCN)	**-235.894192**

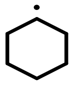

I• *

C	-1.25912300	0.70835400	-0.24284900
C	-1.28175500	-0.77199900	0.15670500
C	-0.00000100	-1.45638700	-0.16566900
C	1.28175400	-0.77200000	0.15670500
C	1.25912400	0.70835300	-0.24285000

C	0.00000100	1.40149400	0.27025000
H	-1.46240700	-0.82685200	1.24414500
H	-2.12427500	-1.28343700	-0.31342900
H	-1.28871600	0.78497000	-1.33440800
H	-2.15270900	1.20945200	0.13619000
H	1.46240500	-0.82685200	1.24414600
H	2.12427400	-1.28344000	-0.31342700
H	2.15271000	1.20945000	0.13618700
H	1.28871500	0.78496800	-1.33440800
H	0.00000100	1.38781000	1.36655100
H	0.00000100	2.45171700	-0.03055900
H	-0.00000100	-2.51467200	-0.39473900

E(UwB97XD, vacuo)	-235.224466
Zero-point correction=	0.156606
Thermal correction to Energy=	0.162638
Thermal correction to Enthalpy=	0.163582
Thermal correction to Gibbs Free Energy=	**0.126870**
E(UwB97XD, MeCN)	**-235.225018**

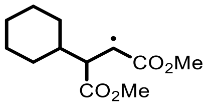

II• *

C	1.19957000	-1.02406400	0.24052000
C	0.14300700	-0.02961300	0.56026900
H	0.94904800	-2.04888200	0.00328500
C	2.59379900	-0.64162800	0.28016000
O	2.99114600	0.46340500	0.57962700
O	3.40919400	-1.65883100	-0.04788900
C	0.28873000	1.20445600	-0.32435200
O	0.49548600	1.17822600	-1.50694600
O	0.11638500	2.32809800	0.37399300
C	0.23639000	3.53897100	-0.36705200
H	1.23003300	3.61434900	-0.80775000
H	-0.50978100	3.58027800	-1.16078900
H	0.07566700	4.34058200	0.34867600
C	4.79874100	-1.35523100	-0.04527300
H	5.01879300	-0.55719500	-0.75485800
H	5.12349900	-1.04238500	0.94760800
H	5.30350000	-2.27227900	-0.33752800
C	-1.27696700	-0.63215700	0.48095500
C	-2.33730400	0.31349700	1.04763300
C	-1.67653300	-1.09604200	-0.92158500
H	-1.25086100	-1.51818900	1.12871800
C	-3.71647600	-0.33961500	1.05957800
H	-2.37558200	1.22222600	0.43749800
H	-2.05640800	0.62948900	2.05551700
C	-3.05216900	-1.75768200	-0.91103800
H	-1.69052000	-0.23561500	-1.59645300
H	-0.93142100	-1.78523100	-1.32556200
C	-4.11311100	-0.82711300	-0.33084300

H	-4.45858200	0.36452600	1.44201000
H	-3.70583000	-1.19019700	1.75032300
H	-3.32582900	-2.05949600	-1.92423300
H	-3.00974600	-2.67393300	-0.31115800
H	-5.08136800	-1.33123400	-0.29361300
H	-4.23238400	0.03701000	-0.99371700
H	0.30401800	0.31631200	1.58791900

E(UwB97XD, vacuo)	-769.658463
Zero-point correction=	0.301940
Thermal correction to Energy=	0.318989
Thermal correction to Enthalpy=	0.319933
Thermal correction to Gibbs Free Energy=	**0.254921**
E(UwB97XD, MeCN)	**-769.668783**

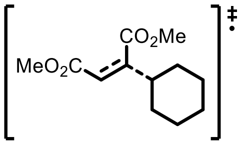

TS2 *

C	1.28137600	-0.48119900	1.31515800
C	0.49768400	0.61414800	1.21185300
H	1.26029400	-1.08828300	2.20966500
C	2.11612900	-0.94707100	0.20787500
O	2.22143000	-0.41156200	-0.86977900
O	2.77195200	-2.07401900	0.53341900
C	0.63323000	1.60638200	0.08661200
O	-0.13788700	1.77747700	-0.81547600
O	1.72400900	2.34904200	0.28188200
C	2.01818600	3.28902700	-0.74876500
H	2.17466500	2.76697100	-1.69241400
H	1.20439200	4.00504000	-0.86319300
H	2.92843900	3.79333700	-0.43600300
C	3.61643300	-2.60761900	-0.47907900
H	3.04016100	-2.85377100	-1.37132100
H	4.39355200	-1.89227800	-0.74939800
H	4.05902300	-3.50481500	-0.05420100
C	-1.66458200	-0.34962300	0.83853500
C	-2.68936200	0.73510500	0.79763600
C	-1.50633300	-1.14701300	-0.41167400
H	-1.59051500	-0.90301900	1.77153900
C	-4.05048000	0.13168900	0.39735300
H	-2.40244800	1.47468900	0.04632200
H	-2.77086000	1.24549900	1.75990200
C	-2.86782500	-1.74290700	-0.81417700
H	-1.15437800	-0.49262300	-1.21361100
H	-0.76647800	-1.93910000	-0.28139500
C	-3.93480600	-0.65426700	-0.90661900
H	-4.79009200	0.92991900	0.30006400
H	-4.40177700	-0.53395100	1.19292700
H	-2.77129400	-2.26630100	-1.76818200
H	-3.17358400	-2.48744500	-0.07125600

H	-4.90037800	-1.09621500	-1.16362400
H	-3.67455100	0.03421600	-1.71751700
H	0.00190600	1.00073000	2.09306800

E(UwB97XD, vacuo)	-769.602704
Zero-point correction=	0.298499
Thermal correction to Energy=	0.315794
Thermal correction to Enthalpy=	0.316738
Thermal correction to Gibbs Free Energy=	**0.250769**
E(UwB97XD, MeCN)	**-769.612212**

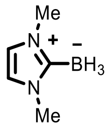

B1 *

C	0.77146100	-1.51291600	0.00045100
C	-0.57300700	-1.58835500	-0.00069400
C	-0.02002600	0.58658100	0.00016700
N	1.09372500	-0.17191800	0.00108600
H	1.52093100	-2.28389900	0.00055100
H	-1.23024800	-2.43936000	-0.00143300
N	-1.04024700	-0.29470000	-0.00124600
B	-0.20596900	2.16894000	-0.00018800
H	-0.87338000	2.44226200	0.98584500
H	0.86229500	2.74239200	0.01366300
H	-0.84723000	2.44391000	-1.00337800
C	2.45286900	0.32989200	-0.00006500
H	2.41369700	1.41469500	0.00879700
H	2.98040700	-0.02503200	0.88524200
H	2.97402200	-0.01029400	-0.89492900
C	-2.43232600	0.10375900	0.00087500
H	-3.05355400	-0.78845400	-0.03763100
H	-2.65203800	0.67404700	0.90217100
H	-2.63323000	0.73759700	-0.86124400

E(UwB97XD, vacuo)	-331.510016
Zero-point correction=	0.160452
Thermal correction to Energy=	0.169544
Thermal correction to Enthalpy=	0.170489
Thermal correction to Gibbs Free Energy=	**0.125942**
E(UwB97XD, MeCN)	**-331.524855**

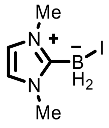

S_{B1} *

C	2.88802900	-0.67473800	-0.67385100
C	2.88795500	0.67473200	-0.67395900

C	1.14896400	0.00000200	0.55017400
N	1.81553600	-1.07089300	0.08802800
H	3.55138800	-1.37776000	-1.14430700
H	3.55125900	1.37775000	-1.14449900
N	1.81554400	1.07089300	0.08803200
B	-0.18660100	0.00000600	1.40843800
H	-0.30841600	-1.01183400	2.04384100
H	-0.30841800	1.01185200	2.04383100
C	1.39349200	-2.44343100	0.30183700
H	1.43794700	-2.68904100	1.36065100
H	0.36953200	-2.56497400	-0.04904500
H	2.05563200	-3.10035600	-0.25711000
C	1.39351600	2.44343200	0.30186100
H	2.05561600	3.10035300	-0.25713800
H	0.36953400	2.56496300	-0.04896000
H	1.43803300	2.68905800	1.36066900
I	-1.82959200	0.00000000	-0.20790400

E(UwB97XD, vacuo)	-628.747854
Zero-point correction=	0.154203
Thermal correction to Energy=	0.164246
Thermal correction to Enthalpy=	0.165190
Thermal correction to Gibbs Free Energy=	**0.116429**
E(UwB97XD, MeCN)	**-628.764106**

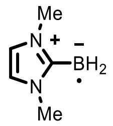

LBR1•*

C	0.67454600	-1.50605400	-0.00002000
C	-0.67456500	-1.50603300	0.00007100
C	0.00002400	0.65105800	-0.00009200
N	1.08697100	-0.19663200	-0.00014000
H	1.36908300	-2.32721100	0.00003800
H	-1.36911100	-2.32718500	0.00002400
N	-1.08696700	-0.19660900	-0.00004900
B	0.00000400	2.14939900	0.00001800
H	1.04312000	2.73736000	-0.00002400
H	-1.04317200	2.73726300	0.00007200
C	2.45297000	0.25824700	0.00008800
H	2.65096700	0.86672200	-0.88357900
H	2.65098300	0.86595700	0.88428500
H	3.11395600	-0.60634300	-0.00030400
C	-2.45296800	0.25824900	0.00004200
H	-3.11393500	-0.60635500	-0.00045300
H	-2.65109500	0.86591000	0.88425000
H	-2.65088400	0.86676700	-0.88361200

E(UwB97XD, vacuo)	-330.877078
Zero-point correction=	0.150417
Thermal correction to Energy=	0.158964
Thermal correction to Enthalpy=	0.159908

Thermal correction to Gibbs Free Energy=	**0.117135**
E(UwB97XD, MeCN)	**-330.886047**

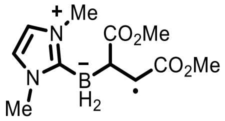

II$^{\bullet}_{B1}$ *

C	1.37510700	-0.48369800	1.31152600
C	0.72528800	0.82451900	1.43619500
H	1.66959400	-1.02363000	2.20130400
C	1.51961000	-1.15557300	0.05051700
O	1.11659200	-0.74522000	-1.02152000
O	2.14773200	-2.35140700	0.17458800
C	1.09015600	1.77648900	0.31898200
O	0.34735700	2.51356800	-0.27578300
O	2.41301900	1.77022200	0.08727500
C	2.85813300	2.58646500	-0.98656900
H	2.39035600	2.27142300	-1.91977000
H	2.61911400	3.63469900	-0.80469400
H	3.93544300	2.44829400	-1.03828600
C	2.34230500	-3.06467700	-1.03547400
H	1.38773300	-3.29900100	-1.51100700
H	2.94235800	-2.48474100	-1.73776300
H	2.86117500	-3.98066100	-0.76316900
H	1.09287100	1.29819000	2.35229700
B	-0.91680500	0.67108800	1.75323400
H	-1.04735600	-0.06636300	2.71368200
H	-1.36377500	1.76694100	2.00847900
C	-1.75660700	0.04988500	0.52919300
C	-3.10938900	-0.13715400	-1.24094300
C	-2.74135100	-1.38208700	-0.88319400
H	-3.74292900	0.20767200	-2.03798600
H	-2.98297300	-2.33999400	-1.30721100
C	-1.34203300	-2.37779800	0.91021200
H	-0.56105600	-2.83921400	0.30714400
H	-0.91778000	-2.02772000	1.84608300
H	-2.12168900	-3.11017200	1.11808600
C	-2.71410300	2.16510600	-0.36423500
H	-1.76007600	2.66478700	-0.22962900
H	-3.14690600	2.44309600	-1.32314300
H	-3.39728800	2.43763400	0.43916100
N	-1.92089200	-1.24965700	0.21073900
N	-2.50098000	0.72639400	-0.36409300

E(UwB97XD, vacuo)	-865.299658
Zero-point correction=	0.294393
Thermal correction to Energy=	0.313797
Thermal correction to Enthalpy=	0.314742
Thermal correction to Gibbs Free Energy=	**0.244927**
E(UwB97XD, MeCN)	**-865.316283**

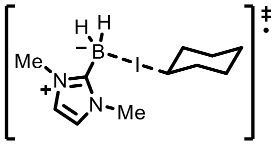

$TS1_{B1}$ *

C	-4.56579800	1.27622200	0.61255100
C	-3.11717100	1.26618800	0.10359400
C	-2.42840500	-0.00872800	0.51693700
C	-3.14666500	-1.25351400	0.06389000
C	-4.59532200	-1.24573700	0.57269800
C	-5.32042000	0.03082300	0.15385200
H	-3.12481300	1.33704500	-0.98948200
H	-2.57638400	2.13827600	0.47569800
H	-4.56278200	1.31377300	1.70745500
H	-5.07444300	2.17981200	0.26668000
H	-3.15545800	-1.28969400	-1.03086900
H	-2.62654400	-2.14929500	0.40803200
H	-5.12480200	-2.12577900	0.19854400
H	-4.59372000	-1.31795400	1.66586300
H	-5.41060000	0.04916900	-0.93806300
H	-6.33741800	0.03645200	0.55349600
H	-2.16782500	-0.02838500	1.57472200
I	-0.17737000	-0.02109800	-0.44297500
C	4.04250700	0.71827700	1.40889300
C	4.05214000	-0.63049400	1.43849900
C	2.98680500	-0.00442800	-0.42983000
N	3.39719100	1.09030200	0.25512700
H	4.44168900	1.43692500	2.10171800
H	4.46139400	-1.31235400	2.16195900
N	3.41263200	-1.06202200	0.30235300
B	2.12772400	-0.03835100	-1.71399600
H	2.05549700	0.98085500	-2.33538900
H	2.06647500	-1.08535700	-2.28856700
C	3.08467400	2.44769900	-0.13674800
H	3.48747100	2.65477600	-1.12663500
H	2.00279500	2.58149300	-0.16147100
H	3.52400200	3.13028000	0.58721000
C	3.11991200	-2.43967700	-0.02955200
H	3.57330200	-3.08373200	0.72057700
H	2.04025100	-2.59169300	-0.04315300
H	3.52158600	-2.68240100	-1.01175400

E(UwB97XD, vacuo)	-863.955455
Zero-point correction=	0.312062
Thermal correction to Energy=	0.329128
Thermal correction to Enthalpy=	0.330072
Thermal correction to Gibbs Free Energy=	**0.262280**

E(UwB97XD, MeCN) **-863.968877**

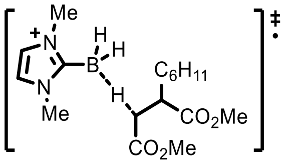

$TS3_{B1}$ *

C	-0.03316700	0.21614700	-1.00436300
C	-1.12292900	0.33746600	0.02049600
H	-0.29463000	-0.10023500	-2.00535800
C	1.09811700	1.10419000	-0.89529900
O	1.37892000	1.77612000	0.08016600
O	1.90092300	1.05294200	-1.98429700
C	-1.74470300	1.72182100	-0.01707000
O	-1.90053300	2.39098000	-1.00283000
O	-2.13499400	2.11899300	1.20296600
C	-2.75312000	3.39899300	1.26534500
H	-2.06750900	4.17146600	0.91709200
H	-3.65334100	3.42363200	0.65064300
H	-3.00400100	3.55743100	2.31113600
C	3.05282600	1.87853900	-1.93313400
H	2.77448100	2.92962400	-1.84837700
H	3.68378400	1.61656000	-1.08188400
H	3.58473400	1.70755600	-2.86671200
C	-2.18251300	-0.77700900	-0.13668500
C	-3.01542800	-0.97532900	1.13020500
C	-3.09778700	-0.59882200	-1.34971100
H	-1.60260000	-1.69446400	-0.28645400
C	-3.97072400	-2.15687400	0.98666200
H	-3.58812900	-0.06626300	1.34060400
H	-2.35034200	-1.13082700	1.98388600
C	-4.04701400	-1.78450700	-1.50153300
H	-3.68515700	0.31824900	-1.23305800
H	-2.50983400	-0.46836000	-2.26049300
C	-4.87105400	-2.00141700	-0.23565000
H	-4.57404900	-2.26280800	1.89141600
H	-3.38635300	-3.07832600	0.88797800
H	-4.70458100	-1.62820300	-2.35979300
H	-3.46407800	-2.68820000	-1.71128400
H	-5.51500900	-2.87710400	-0.34639300
H	-5.53231400	-1.14006500	-0.08747100
H	-0.67705400	0.22302600	1.01043600
C	4.08391200	-0.06390900	1.59491200
C	4.63892800	-0.52488700	0.45662200
C	2.57317000	-1.37908800	0.59727000
N	2.82258400	-0.60175000	1.67139800
H	4.47195300	0.59648600	2.34903900
H	5.61083700	-0.35611900	0.02905100
N	3.70015300	-1.32912800	-0.14492500
B	1.22982300	-2.08510100	0.19392800
H	1.38211500	-3.00225300	-0.57434800
H	0.48377000	-2.28847600	1.12071900
H	0.64870700	-1.09715000	-0.50931100
C	1.84204400	-0.22999400	2.67162400
H	1.31638500	0.66702200	2.34155700
H	1.13447700	-1.04489800	2.79471200

H	2.35117900	-0.04016700	3.61483600
C	3.84330800	-1.95016300	-1.44600200
H	4.86700300	-1.81057000	-1.78663700
H	3.61933700	-3.01234200	-1.37939800
H	3.15420800	-1.48578800	-2.15126700

E(UwB97XD, vacuo)	-1101.177270
Zero-point correction=	0.462319
Thermal correction to Energy=	0.488819
Thermal correction to Enthalpy=	0.489764
Thermal correction to Gibbs Free Energy=	**0.402464**
E(UwB97XD, MeCN)	**-1101.195435**

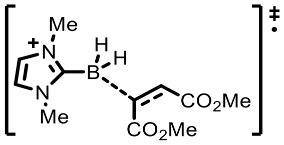

TS4$_{B1}$ *

C	-0.47712800	1.12388900	1.35575200
C	-1.18030600	-0.01888800	1.51648600
H	0.03927500	1.56799200	2.19507900
C	-0.25390400	1.71743600	0.04827700
O	-0.65957100	1.29457400	-1.01139100
O	0.51916800	2.82783500	0.13243900
C	-2.11478900	-0.55312500	0.47442900
O	-2.04000000	-1.61458900	-0.08411700
O	-3.13247700	0.29345900	0.29682800
C	-4.05434400	-0.05943800	-0.72812300
H	-3.54261000	-0.10222300	-1.68975800
H	-4.51474700	-1.02575200	-0.52168200
H	-4.80520000	0.72637000	-0.73280400
C	0.78796500	3.47495300	-1.10200800
H	1.28128000	2.79537800	-1.79841400
H	-0.13449000	3.83119500	-1.56200100
H	1.43713600	4.31468700	-0.86495800
H	-1.32395400	-0.43318800	2.50553300
B	0.67038000	-1.88056600	1.76481200
H	0.98310700	-1.43759400	2.83127400
H	-0.00220800	-2.86277700	1.69443100
C	1.37826500	-1.37923500	0.51299000
C	1.95231200	-1.09285000	-1.63638300
C	2.53483600	-0.10221000	-0.93115400
H	1.96613900	-1.29833600	-2.69157500
H	3.15688300	0.71467500	-1.25001400
C	2.71903900	0.51042100	1.46807300
H	3.78265800	0.29824200	1.59498800
H	2.57657500	1.56974100	1.25612600
H	2.19702600	0.25631200	2.38427200
C	0.53276000	-3.07800400	-1.09563100
H	-0.49495900	-2.99455000	-0.75203800
H	0.55096200	-3.19342400	-2.17728300
H	1.00913900	-3.94161300	-0.63058800

N	2.17802500	-0.27896300	0.38402700
N	1.25638800	-1.87107100	-0.75273700

E(UwB97XD, vacuo)	-865.264533
Zero-point correction=	0.291706
Thermal correction to Energy=	0.311457
Thermal correction to Enthalpy=	0.312402
Thermal correction to Gibbs Free Energy=	**0.242138**
E(UwB97XD, MeCN)	**-865.279277**

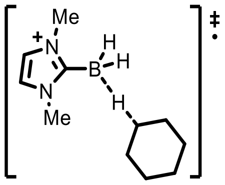

TS5$_{B1}$ *

C	1.80436000	-1.25201000	0.90563200
C	2.41159100	-1.28090100	-0.49748800
C	2.19820800	0.00203400	-1.24313800
C	2.40786200	1.28457100	-0.49575600
C	1.80081600	1.25209200	0.90735600
C	2.22525400	0.00011400	1.66824200
H	3.49033300	-1.48358100	-0.40242900
H	1.99870300	-2.11509200	-1.07339300
H	0.71261700	-1.25359600	0.82882800
H	2.08799600	-2.15219300	1.45647000
H	3.48600400	1.49032600	-0.40052700
H	1.99244200	2.11826400	-1.07057600
H	2.08202200	2.15230500	1.45939400
H	0.70907100	1.25078300	0.83063000
H	3.31381500	0.00156700	1.79790700
H	1.78738400	-0.00120100	2.66958600
H	2.53952900	0.00331400	-2.27383100
C	-2.39424300	0.67186300	1.19642100
C	-2.39297400	-0.67500000	1.19623600
C	-1.46541500	-0.00042500	-0.73628500
N	-1.83030700	1.07227900	0.00488100
H	-2.74811500	1.37313700	1.93049000
H	-2.74555500	-1.37713800	1.93010100
N	-1.82824600	-1.07401500	0.00461800
B	-0.64186700	0.00017400	-2.05847000
H	-0.76484700	-1.02312500	-2.69190400
H	-0.76527100	1.02353600	-2.69168800
H	0.60152600	0.00020500	-1.59009700
C	-1.60673500	2.44146200	-0.40346500
H	-0.54026300	2.62198500	-0.53880700
H	-2.11077700	2.63678200	-1.34844400
H	-1.99462000	3.10460500	0.36693500
C	-1.60240100	-2.44265000	-0.40433700
H	-2.11054200	-2.64016400	-1.34668500
H	-0.53604200	-2.61961600	-0.54495700
H	-1.98414700	-3.10671700	0.36834100

E(UwB97XD, vacuo)	-566.730491
Zero-point correction=	0.316489
Thermal correction to Energy=	0.331911
Thermal correction to Enthalpy=	0.332856
Thermal correction to Gibbs Free Energy=	**0.272515**
E(UwB97XD, MeCN)	**-566.741958**

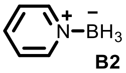

B2

B2

B	-2.50233200	0.00001300	0.02092400
H	-2.85647700	1.01485900	-0.54016600
H	-2.85643300	-1.01372100	-0.54221300
N	-0.89053800	0.00006000	-0.02188400
C	-0.21485300	1.15169100	-0.01529600
C	-0.21493600	-1.15163400	-0.01529500
C	1.16413200	1.19140400	0.00391300
H	-0.82415800	2.04426300	-0.02749800
C	1.16403100	-1.19146200	0.00391100
H	-0.82431800	-2.04415600	-0.02750300
C	1.86944700	-0.00005200	0.01349700
H	1.66955800	2.14663500	0.00751400
H	1.66938300	-2.14673200	0.00751000
H	2.95153800	-0.00009600	0.02446500
H	-2.79059400	-0.00121900	1.20207200

E(UwB97XD, vacuo)	-274.947601
Zero-point correction=	0.122380
Thermal correction to Energy=	0.128732
Thermal correction to Enthalpy=	0.129676
Thermal correction to Gibbs Free Energy=	**0.091638**
E(UwB97XD, MeCN)	**-274.960773**

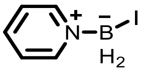

S$_{B2}$

B	0.26859100	0.00199500	1.42485400
H	0.36680700	1.02223500	2.04707400
H	0.36687100	-1.01654200	2.04985300
N	-1.13836500	0.00093300	0.68978700
C	-1.72627500	1.15672000	0.36304400
C	-1.72545900	-1.15582700	0.36498500
C	-2.93259900	1.19290100	-0.30155500
H	-1.18778000	2.04894700	0.64857700
C	-2.93174900	-1.19398700	-0.29954600
H	-1.18632100	-2.04719200	0.65200100
C	-3.54798700	-0.00104300	-0.63924600
H	-3.37599600	2.14667400	-0.54850200

H	-3.37447000	-2.14848800	-0.54488900
H	-4.49571400	-0.00181700	-1.16150500
I	1.82446500	-0.00024300	-0.22682000

E(UwB97XD, vacuo) -572.181440
Zero-point correction= 0.115579
Thermal correction to Energy= 0.122907
Thermal correction to Enthalpy= 0.123852
Thermal correction to Gibbs Free Energy= **0.080799**

E(UwB97XD, MeCN) **-572.196923**

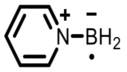

LBR2•

B	2.42363000	0.00000000	-0.00070100
H	2.98966400	1.05026200	-0.00109600
H	2.98966200	-1.05026300	-0.00161200
N	1.00598400	0.00000000	0.00033400
C	0.26849800	1.19026500	0.00056300
C	0.26849800	-1.19026500	0.00058900
C	-1.08912300	1.19874100	-0.00005800
H	0.86214600	2.09164500	0.00103800
C	-1.08912400	-1.19874000	-0.00003300
H	0.86214500	-2.09164600	0.00108200
C	-1.82097100	0.00000000	-0.00053300
H	-1.59487200	2.15516000	-0.00000300
H	-1.59487200	-2.15516000	0.00003400
H	-2.90058400	0.00000000	-0.00144100

E(UwB97XD, vacuo) -274.326905
Zero-point correction= 0.111526
Thermal correction to Energy= 0.117345
Thermal correction to Enthalpy= 0.118289
Thermal correction to Gibbs Free Energy= **0.081672**

E(UwB97XD, MeCN) **-274.329484**

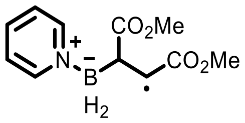

$II^{\bullet}_{B2}$

C	-0.84564700	0.90173700	1.39278700
C	-1.17908500	-0.52697500	1.40350500
H	-0.83500000	1.45778100	2.32032000
C	-0.36466700	1.56759300	0.21206500
O	-0.22610900	1.05088400	-0.88131700
O	-0.05395100	2.86344700	0.44383800
C	-1.91002000	-0.99262800	0.16061600
O	-1.67743700	-1.98415300	-0.48056200
O	-2.93562300	-0.17830700	-0.13119500

C	-3.65381300	-0.48837600	-1.31769600
H	-2.98914600	-0.45296500	-2.18128700
H	-4.10169500	-1.48035500	-1.25345700
H	-4.42713800	0.27095400	-1.40411500
C	0.42696700	3.58447700	-0.67913500
H	1.34322000	3.13551300	-1.06657900
H	-0.31521800	3.60125200	-1.47821300
H	0.62210600	4.59385900	-0.32506300
H	-1.85913000	-0.71852900	2.24020500
B	0.15769900	-1.43051500	1.81686100
H	0.59160000	-1.05291700	2.88366300
H	-0.10814900	-2.60682300	1.80150400
C	1.32524500	-1.85653800	-0.40882900
C	2.24139100	-0.23806500	0.96146300
C	2.26443500	-1.60564500	-1.38708200
H	0.53258800	-2.58077200	-0.52456800
C	3.20665200	0.06629100	0.02742200
H	2.17660400	0.26539100	1.91542200
C	3.21849300	-0.62811600	-1.17091200
H	2.23236700	-2.16983400	-2.30781900
H	3.93380100	0.83591700	0.24321000
H	3.96275400	-0.40906400	-1.92547300
N	1.31674300	-1.17918800	0.74380100

E(UwB97XD, vacuo)	-808.739436
Zero-point correction=	0.255407
Thermal correction to Energy=	0.272404
Thermal correction to Enthalpy=	0.273348
Thermal correction to Gibbs Free Energy=	**0.208809**
E(UwB97XD, MeCN)	**-808.754788**

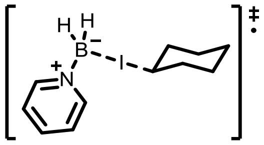

TS1$_{B2}$

C	3.83856900	1.62175500	-0.99823900
C	2.41178400	1.05143700	-0.96883800
C	2.41693100	-0.30664600	-0.32463500
C	3.00220600	-0.32451600	1.05987900
C	4.42951400	0.24458600	1.03079800
C	4.45657300	1.63335900	0.39762700
H	1.77281100	1.72683300	-0.38949200
H	1.99800200	1.00332600	-1.97731400
H	4.45744500	1.00949600	-1.66278200
H	3.82452000	2.63028300	-1.41926200
H	2.38826200	0.29444100	1.72303100
H	3.00112200	-1.33493700	1.47119900
H	4.83392600	0.27849200	2.04553000
H	5.07400800	-0.42825200	0.45512000
H	3.89455500	2.32756500	1.03254100
H	5.48198100	2.00796200	0.35304000

I	0.05669200	-1.08107700	-0.16845400
B	-2.38323900	-1.74491400	0.02670900
H	-2.39970000	-2.33488900	1.06492800
H	-2.57241700	-2.30300800	-1.01205400
N	-3.03076200	-0.38180900	0.10139600
C	-3.19154100	0.24362800	1.29193400
C	-3.38206700	0.28011700	-1.02683900
C	-3.69294600	1.51777500	1.38195900
H	-2.88833900	-0.32734700	2.15676800
C	-3.88809700	1.55513600	-0.99395800
H	-3.22527500	-0.26265600	-1.94700200
C	-4.05179300	2.19875900	0.22591200
H	-3.80009200	1.97107800	2.35727100
H	-4.15155000	2.03853300	-1.92405900
H	-4.44832200	3.20348800	0.27427500
H	2.79111600	-1.09844900	-0.97060800

E(UwB97XD, vacuo)	-807.388986
Zero-point correction=	0.273067
Thermal correction to Energy=	0.287466
Thermal correction to Enthalpy=	0.288410
Thermal correction to Gibbs Free Energy=	**0.226642**
E(UwB97XD, MeCN)	**-807.401526**

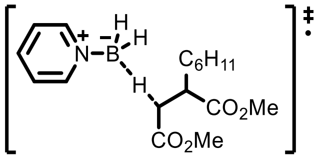

TS3$_{B2}$

C	-1.39050200	-0.50390100	-0.93853500
C	-0.29948300	-0.74983100	0.06489900
H	-1.38251900	-1.09628600	-1.84438500
C	-2.70651200	-0.16415200	-0.43040600
O	-2.93301200	0.30906400	0.66883300
O	-3.67000900	-0.35165100	-1.34705900
C	-0.52269500	-2.08217600	0.76310000
O	-1.03226400	-3.05156900	0.27002600
O	-0.05779800	-2.06193600	2.02073400
C	-0.19967600	-3.27617100	2.75061700
H	-1.25104100	-3.55000900	2.83638900
H	0.33660700	-4.08741900	2.25766300
H	0.22342900	-3.08328600	3.73307200
C	-4.97410700	0.06202300	-0.96851500
H	-5.31115800	-0.47756700	-0.08291700
H	-4.99478200	1.13218500	-0.75839900
H	-5.61551300	-0.16636000	-1.81611600
C	1.11398200	-0.66971700	-0.55849300
C	2.20557000	-0.43751100	0.48632500
C	1.47648200	-1.86037300	-1.44717000
H	1.09029700	0.21342800	-1.20599100
C	3.56241000	-0.21815700	-0.17555600
H	2.26262900	-1.29944500	1.15762500

H	1.94322800	0.42188100	1.10942400
C	2.83207300	-1.64853400	-2.11670800
H	1.51087400	-2.77254700	-0.84171200
H	0.70565100	-2.02266800	-2.20266700
C	3.92911100	-1.38477800	-1.08886600
H	4.33400200	-0.07325800	0.58454100
H	3.52726300	0.70373000	-0.76885300
H	3.08712500	-2.51755000	-2.72697800
H	2.76432400	-0.79439500	-2.79981400
H	4.88039600	-1.19079900	-1.58977700
H	4.07180500	-2.28342800	-0.47848700
H	-0.36625100	0.01310500	0.84178600
B	-0.78482600	2.17331700	-1.77660900
H	-1.89203200	2.64414400	-1.82370800
H	-0.07251400	2.28274300	-2.74143800
H	-1.00321300	0.86378100	-1.52415500
C	-0.69881700	2.68127500	0.68078200
C	1.26740700	2.93733000	-0.51798400
C	-0.06068600	3.00829000	1.85534400
H	-1.74248300	2.40016900	0.64679300
C	1.96177100	3.27096300	0.62275800
H	1.73490100	2.89014900	-1.49053400
C	1.29214100	3.31003100	1.83519300
H	-0.62758100	3.01249000	2.77520100
H	3.01726600	3.49206400	0.55286700
H	1.81532500	3.56525000	2.74693700
N	-0.04400600	2.64491300	-0.49490100

E(UwB97XD, vacuo)	-1044.610024
Zero-point correction=	0.423744
Thermal correction to Energy=	0.447606
Thermal correction to Enthalpy=	0.448550
Thermal correction to Gibbs Free Energy=	**0.367347**
E(UwB97XD, MeCN)	**-1044.627816**

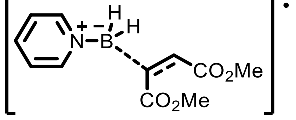

TS4$_{B2}$

C	-0.46729100	0.87553900	1.50223700
C	-1.29133900	-0.20221300	1.43331700
H	-0.09179400	1.21495000	2.45781000
C	0.01581300	1.56625800	0.31357200
O	-0.22417900	1.25848400	-0.83306300
O	0.78775000	2.62617000	0.63582600
C	-2.05108500	-0.55949100	0.18781500
O	-1.93286900	-1.56969000	-0.45226400
O	-2.95831000	0.38078900	-0.08538000
C	-3.68311700	0.20154100	-1.29775800
H	-2.99605100	0.20119000	-2.14397400
H	-4.23833300	-0.73641000	-1.28337800

H	-4.36422100	1.04601500	-1.36172100
C	1.31753600	3.34818700	-0.46534500
H	1.93239800	2.70028400	-1.09207200
H	0.51779100	3.76617300	-1.07766100
H	1.92052200	4.14557300	-0.03771200
H	-1.71331800	-0.60549800	2.34599400
B	0.18202700	-2.01407700	1.78184500
H	0.49984700	-1.66876700	2.87973100
H	-0.58578600	-2.89971500	1.58128500
C	0.97205200	-2.18271100	-0.55793800
C	2.04749500	-0.68913500	0.90096100
C	1.71847100	-1.73085500	-1.60480600
H	0.20840900	-2.93597300	-0.66285000
C	2.81947000	-0.20875400	-0.12819600
H	2.14723800	-0.33819800	1.91606400
C	2.65747100	-0.71372600	-1.40792400
H	1.55818600	-2.16166000	-2.58266600
H	3.53928400	0.57004700	0.08262700
H	3.24800300	-0.33857800	-2.23191300
N	1.11367300	-1.66898700	0.70352100

E(UwB97XD, vacuo)	-808.708640
Zero-point correction=	0.252861
Thermal correction to Energy=	0.269871
Thermal correction to Enthalpy=	0.270815
Thermal correction to Gibbs Free Energy=	**0.206956**
E(UwB97XD, MeCN)	**-808.720722**

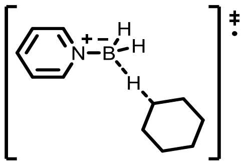

TS5$_{B2}$

C	-2.15686000	-0.74824700	1.18581200
C	-2.87113500	0.50671300	0.68490200
C	-2.19337400	1.09539300	-0.51246100
C	-1.79928400	0.15315700	-1.60860400
C	-1.12452300	-1.11503800	-1.08272100
C	-1.92444900	-1.74548300	0.05406800
H	-3.90799200	0.23801600	0.42566900
H	-2.93574400	1.25060700	1.48220500
H	-1.19165100	-0.45490500	1.61061800
H	-2.73182400	-1.21257000	1.99034200
H	-2.70074600	-0.14405000	-2.16914400
H	-1.15124200	0.65962700	-2.32876700
H	-0.99227500	-1.83124800	-1.89728100
H	-0.12402700	-0.86830500	-0.71658300
H	-2.89309200	-2.08623100	-0.33015900
H	-1.40733900	-2.63194700	0.43000500
H	-2.54310400	2.07275500	-0.82894700
B	0.32398200	2.14021400	0.67290700
H	0.13094300	2.26049200	1.85989100

H	0.60522600	3.14330700	0.06089500
H	-0.75108300	1.63445700	0.13310000
C	2.01725700	0.97294400	-0.77400800
C	1.59271500	0.05807400	1.30842300
C	2.83651000	-0.07898800	-1.10642400
H	1.82175700	1.79437900	-1.44778300
C	2.39865800	-1.02341400	1.03372900
H	1.07898100	0.17471800	2.25119900
C	3.03536400	-1.10757400	-0.19476400
H	3.31418100	-0.08748900	-2.07610900
H	2.52413500	-1.78936100	1.78614400
H	3.67287700	-1.94737300	-0.43466200
N	1.38526300	1.04554600	0.41530200

E(UwB97XD, vacuo)	-510.166234
Zero-point correction=	0.278217
Thermal correction to Energy=	0.290851
Thermal correction to Enthalpy=	0.291796
Thermal correction to Gibbs Free Energy=	**0.237614**
E(UwB97XD, MeCN)	**-510.175449**

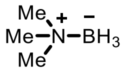

B3

N	0.00447200	0.00000000	-0.00000500
C	-0.48731300	1.36354000	-0.27123100
H	-0.10670600	2.03218100	0.49736800
H	-0.10650700	1.68723400	-1.23709700
H	-1.58019500	1.38133600	-0.27488300
C	-0.48823000	-0.44658800	1.31623600
H	-0.10839500	-1.44679300	1.51119900
H	-0.10732500	0.22780400	2.07964700
H	-1.58111900	-0.45158600	1.33295700
C	-0.48828600	-0.91632500	-1.04509200
H	-0.10778600	-0.58530500	-2.00858700
H	-0.10809100	-1.91488900	-0.84259100
H	-1.58117500	-0.92767300	-1.05833100
B	1.64491800	-0.00070500	0.00009800
H	1.97093500	-1.14829200	0.22696700
H	1.97169300	0.37644200	-1.10701900
H	1.97174700	0.76929900	0.88043100

E(UwB97XD, vacuo)	-201.154012
Zero-point correction=	0.155930
Thermal correction to Energy=	0.162685
Thermal correction to Enthalpy=	0.163630
Thermal correction to Gibbs Free Energy=	**0.126993**
E(UwB97XD, MeCN)	**-201.164667**

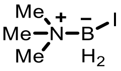

S_{B3}

N	-1.78532300	0.00082100	0.00001200
C	-3.02029000	0.81945000	-0.00036700
H	-3.02707100	1.45007500	0.88543900
H	-3.02653300	1.45005900	-0.88618900
H	-3.89538900	0.16733200	-0.00062900
C	-1.78126300	-0.85342900	1.20881900
H	-0.88097100	-1.46270700	1.20942300
H	-1.77687900	-0.21399100	2.08865000
H	-2.66745000	-1.49052000	1.21209100
C	-1.78050700	-0.85341400	-1.20880100
H	-1.77555200	-0.21396700	-2.08862200
H	-0.88022200	-1.46270500	-1.20883600
H	-2.66669700	-1.49049700	-1.21264500
B	-0.54930000	1.06110500	0.00037800
H	-0.64127000	1.69238300	-1.01737500
H	-0.64127600	1.69171300	1.01854500
I	1.44557400	-0.00196400	0.00000500

E(UwB97XD, vacuo)	-498.385706
Zero-point correction=	0.148573
Thermal correction to Energy=	0.156563
Thermal correction to Enthalpy=	0.157507
Thermal correction to Gibbs Free Energy=	**0.115191**
E(UwB97XD, MeCN)	**-498.397925**

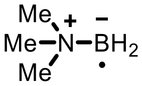

LBR3•

N	0.00000000	0.02145400	-0.05770800
C	-1.20484900	-0.83237300	-0.10967200
H	-2.08600800	-0.19593600	-0.07914500
H	-1.20074000	-1.37945500	-1.04960200
H	-1.21351400	-1.52546000	0.73542700
C	0.00000800	0.78581700	1.21166700
H	0.88502400	1.41708700	1.23707300
H	-0.88497300	1.41713700	1.23705300
H	-0.00001900	0.10644100	2.06981600
C	1.20483000	-0.83240000	-0.10967000
H	1.20069100	-1.37951300	-1.04958100
H	2.08600200	-0.19598000	-0.07918100
H	1.21349600	-1.52546200	0.73545000
B	0.00001300	1.03519000	-1.30826900
H	1.05711400	1.60436000	-1.38297900
H	-1.05707100	1.60439000	-1.38297900

E(UwB97XD, vacuo)	-200.480650
Zero-point correction=	0.144249
Thermal correction to Energy=	0.151071

Thermal correction to Enthalpy=	0.152015
Thermal correction to Gibbs Free Energy=	**0.114688**
E(UwB97XD, MeCN)	**-200.493172**

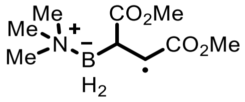

$\mathbf{II^{\bullet}_{B3}}$

C	0.64986100	-0.52221900	1.27064600
C	-0.14032400	0.71314900	1.17105700
H	0.66537100	-1.06317400	2.20739700
C	1.34328600	-1.11464000	0.15867900
O	1.28671200	-0.73080800	-0.99609000
O	2.06499500	-2.19487100	0.52482100
C	0.44281500	1.74220800	0.22085000
O	-0.15793400	2.40858200	-0.57803100
O	1.76124800	1.89360400	0.43348700
C	2.42716600	2.79896800	-0.43573600
H	2.33318700	2.46813700	-1.47046100
H	2.01199900	3.80295600	-0.34423900
H	3.47061100	2.79175300	-0.13017800
C	2.78338100	-2.83621000	-0.51888100
H	2.10497600	-3.20486400	-1.28956900
H	3.49338000	-2.14871400	-0.97980700
H	3.30947700	-3.66509600	-0.05197000
H	-0.08112300	1.19710700	2.15530100
B	-1.78362400	0.50172000	1.12868500
H	-2.13034600	-0.12034500	2.11331700
H	-2.30915300	1.58708700	1.06184600
N	-2.40698200	-0.35979100	-0.12529700
C	-3.87450400	-0.18789200	-0.05023900
H	-4.11502700	0.86109900	-0.19966400
H	-4.35723700	-0.79490300	-0.81916600
H	-4.21747400	-0.49388000	0.93587700
C	-2.10751500	-1.80188900	-0.00749400
H	-2.60791400	-2.35077500	-0.80817400
H	-1.03641600	-1.95818600	-0.08655000
H	-2.45318100	-2.15785600	0.96021200
C	-1.93916300	0.12669400	-1.44461500
H	-2.10796100	1.19777600	-1.50303700
H	-0.87292200	-0.06420200	-1.54115300
H	-2.48343300	-0.39504000	-2.23542700

E(UwB97XD, vacuo)	-734.937376
Zero-point correction=	0.288764
Thermal correction to Energy=	0.306398
Thermal correction to Enthalpy=	0.307342
Thermal correction to Gibbs Free Energy=	**0.242789**
E(UwB97XD, MeCN)	**-734.950675**

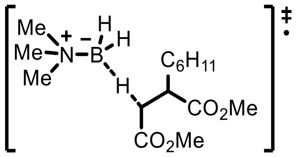

TS3$_{B3}$

C	-0.52248500	0.51118300	0.84392700
C	0.58819400	0.34378700	-0.16806000
H	-0.20542200	0.55414200	1.88075400
C	-1.54611400	1.49022200	0.51599700
O	-1.87325800	1.81558200	-0.61129300
O	-2.20258700	1.94123000	1.60513300
C	1.23618300	1.67446600	-0.49851600
O	1.28163000	2.63010500	0.22817300
O	1.79812000	1.65894100	-1.71641400
C	2.46523900	2.85478700	-2.10290700
H	1.76880000	3.69303900	-2.11910700
H	3.27694300	3.08449300	-1.41199300
H	2.85730700	2.66792300	-3.09944300
C	-3.23672900	2.88077100	1.35722300
H	-2.84284100	3.76719300	0.85951100
H	-4.01964200	2.45040000	0.72966500
H	-3.64021500	3.14370200	2.33212800
C	1.63272400	-0.68944400	0.32059800
C	2.37085800	-1.37618700	-0.82822400
C	2.63118100	-0.12032200	1.33081600
H	1.05194600	-1.46413200	0.83235300
C	3.31223000	-2.46142000	-0.31273200
H	2.93949600	-0.63283100	-1.39520100
H	1.64255900	-1.80957800	-1.51950300
C	3.56421800	-1.20758400	1.85607400
H	3.23150000	0.66054400	0.84999900
H	2.10658000	0.36424500	2.15700900
C	4.29595500	-1.90756300	0.71434400
H	3.85121000	-2.91752600	-1.14659200
H	2.71889300	-3.25699400	0.15180600
H	4.28123900	-0.77721500	2.55891900
H	2.97861000	-1.94550100	2.41555300
H	4.92937700	-2.70843300	1.10309200
H	4.96180500	-1.18907800	0.22286900
H	0.16269400	-0.03495200	-1.10015200
B	-1.68803600	-2.07884700	0.52916000
H	-2.04605200	-2.49642400	1.60061700
H	-0.82503800	-2.67576700	-0.06436900
H	-1.15277600	-0.67844500	0.76524900
N	-2.96215200	-1.90784500	-0.43375300
C	-2.57108100	-1.26716800	-1.71252400
H	-3.43063800	-1.23626500	-2.38531600
H	-2.22393700	-0.25346000	-1.51942500
H	-1.77080200	-1.85134600	-2.16150000
C	-3.51615300	-3.25170600	-0.71506100
H	-3.78971900	-3.72455400	0.22539000
H	-4.39398400	-3.16734200	-1.36049800
H	-2.75180500	-3.85025000	-1.20512100
C	-4.00003400	-1.08644700	0.22784400

H	-4.27424400	-1.55869800	1.16818100
H	-3.59792600	-0.09731500	0.42754300
H	-4.87503400	-1.00155600	-0.41953700

E(UwB97XD, vacuo)	-970.806098
Zero-point correction=	0.456708
Thermal correction to Energy=	0.481413
Thermal correction to Enthalpy=	0.482357
Thermal correction to Gibbs Free Energy=	**0.400200**
E(UwB97XD, MeCN)	**-970.822953**

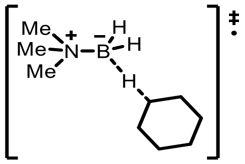

TS5$_{B3}$

C	-2.45767700	1.25839100	0.56384900
C	-1.74935500	1.27278800	-0.79068600
C	-0.96876700	0.00022100	-1.01573400
C	-1.74906800	-1.27256700	-0.79094900
C	-2.45739600	-1.25861300	0.56358800
C	-3.30494900	-0.00022200	0.73160500
H	-2.50104100	1.40660000	-1.58346500
H	-1.08136200	2.13839500	-0.84988600
H	-1.70272600	1.28828900	1.35561400
H	-3.07723600	2.15189300	0.67497000
H	-2.50072700	-1.40637500	-1.58375600
H	-1.08090800	-2.13803200	-0.85033600
H	-3.07676000	-2.15227500	0.67451600
H	-1.70244200	-1.28850600	1.35535000
H	-4.10645300	-0.00023500	-0.01738900
H	-3.79023300	-0.00037900	1.71085200
H	-0.41686200	0.00038300	-1.95965500
B	1.04742500	0.00049000	1.02761900
H	1.01194800	-1.03052700	1.65911600
H	1.01227500	1.03191300	1.65847000
H	0.01143400	0.00032000	-0.00192000
N	2.40721500	0.00000800	0.14482500
C	3.58603300	0.00001100	1.03612600
H	3.54598400	-0.88439700	1.66758100
H	4.50860200	-0.00057600	0.44828400
H	3.54660200	0.88503300	1.66676100
C	2.43967800	-1.20625600	-0.70773800
H	1.56604700	-1.20196100	-1.35482400
H	3.35193500	-1.21981300	-1.30888600
H	2.40147700	-2.08540700	-0.06882500
C	2.44012100	1.20583700	-0.70833700
H	1.56677700	1.20126300	-1.35581100
H	2.40167600	2.08531200	-0.06988600
H	3.35264200	1.21904000	-1.30909300

E(UwB97XD, vacuo)	-436.359687

Zero-point correction=	0.311290
Thermal correction to Energy=	0.324810
Thermal correction to Enthalpy=	0.325754
Thermal correction to Gibbs Free Energy=	**0.270101**
E(UwB97XD, MeCN)	**-436.368182**

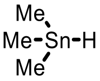

Sn_H

C	-0.97194600	-1.77599700	0.47301700
H	-2.00243800	-1.81687900	0.12010200
H	-0.45035700	-2.66607200	0.12110600
H	-0.97892700	-1.78747300	1.56335200
C	2.02407100	0.04646800	0.47317400
H	2.57519300	-0.82482800	0.11928900
H	2.53355800	0.94390100	0.12219200
H	2.03759400	0.04500400	1.56350600
C	-1.05245700	1.72950200	0.47302800
H	-0.57139100	2.64260200	0.12237400
H	-2.08330300	1.72380500	0.11883400
H	-1.06131400	1.73987300	1.56336000
Sn	0.00006100	0.00000400	-0.23942400
H	0.00029600	0.00004400	-1.95825400

E(UwB97XD, vacuo)	-334.713673
Zero-point correction=	0.115138
Thermal correction to Energy=	0.123830
Thermal correction to Enthalpy=	0.124774
Thermal correction to Gibbs Free Energy=	**0.081231**
E(UwB97XD, MeCN)	**-334.715227**

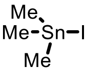

S_{Sn} *

Sn	-0.97610100	-0.00000100	-0.00002500
C	-1.57463000	-0.07660900	2.05072800
H	-2.66295100	-0.07915300	2.12306300
H	-1.18535200	-0.98072500	2.51667500
H	-1.18508800	0.79008500	2.58285200
C	-1.57430600	1.81441800	-0.95904200
H	-1.18864500	1.83977400	-1.97722300
H	-2.66259500	1.88087600	-0.98888600
H	-1.18087900	2.66976400	-0.41162900
C	-1.57438300	-1.73775300	-1.09175900
H	-2.66268500	-1.80102500	-1.12751200
H	-1.18769800	-1.68772400	-2.10864500
H	-1.18208900	-2.63167600	-0.60897100
I	1.74043300	-0.00000900	0.00003700

E(UwB97XD, vacuo)	-631.965891
Zero-point correction=	0.109211
Thermal correction to Energy=	0.119547
Thermal correction to Enthalpy=	0.120491
Thermal correction to Gibbs Free Energy=	**0.070734**
E(UwB97XD, MeCN)	**-631.970799**

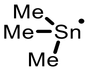

$Sn_R^•$ *

Sn	0.00000200	0.00002000	-0.29626800
C	-1.70175600	1.08249500	0.49917000
H	-1.65131200	1.05005500	1.59003500
H	-1.68466300	2.12278300	0.17666100
H	-2.63670200	0.62637000	0.17619700
C	-0.08664500	-2.01502300	0.49912700
H	0.77677900	-2.59609000	0.17763300
H	-0.08564700	-1.95507300	1.58999300
H	-0.99522500	-2.52099700	0.17515200
C	1.78838900	0.93243300	0.49917400
H	1.73488200	0.90535900	1.59004000
H	2.68064300	0.39709900	0.17701300
H	1.86121500	1.97005600	0.17586100

E(UwB97XD, vacuo)	-334.081207
Zero-point correction=	0.107025
Thermal correction to Energy=	0.115545
Thermal correction to Enthalpy=	0.116490
Thermal correction to Gibbs Free Energy=	**0.072357**
E(UwB97XD, MeCN)	**-334.082302**

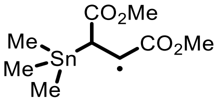

$II_{Sn}^•$

C	-1.31604700	-0.19562400	-1.37941400
C	-0.08059100	0.55153700	-1.20168700
H	-1.62098900	-0.51911900	-2.36588700
C	-2.11395400	-0.64241900	-0.26123200
O	-1.76189000	-0.61042800	0.90173700
O	-3.29898800	-1.14536100	-0.65015200
C	-0.12044300	1.91082500	-0.57194000
O	0.83834000	2.64242100	-0.51318300
O	-1.31428600	2.23139000	-0.07071600
C	-1.38276700	3.46733800	0.63224400
H	-0.69036100	3.46599700	1.47408900
H	-1.13937000	4.30041300	-0.02682100
H	-2.40721400	3.54782400	0.98558300
C	-4.12878800	-1.63936600	0.39249700

H	-3.63683300	-2.45417700	0.92507000
H	-4.36684300	-0.84859100	1.10444200
H	-5.03359600	-1.99700200	-0.09214900
H	0.47141900	0.65454900	-2.13474000
Sn	1.36682800	-0.56630300	0.10972500
C	0.82385300	-2.63562500	0.05612900
H	-0.06124300	-2.79689500	0.66900900
H	0.61059100	-2.94557800	-0.96710100
H	1.64111200	-3.24730700	0.43946300
C	3.24504700	-0.23238500	-0.86899900
H	4.06684300	-0.62956200	-0.27218100
H	3.26092800	-0.71461200	-1.84668400
H	3.38868400	0.83995000	-0.99929300
C	1.32633700	0.30613400	2.05563200
H	0.31887500	0.22185800	2.45853800
H	2.02651300	-0.20532100	2.71660100
H	1.60881100	1.35605300	1.98495400

E(UwB97XD, vacuo)	-868.499031
Zero-point correction=	0.248091
Thermal correction to Energy=	0.268527
Thermal correction to Enthalpy=	0.269471
Thermal correction to Gibbs Free Energy=	**0.196317**
E(UwB97XD, MeCN)	**-868.508541**

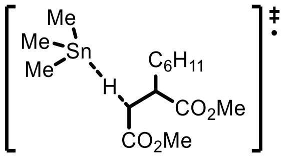

TS3$_{Sn}$

C	0.16777700	1.15393800	0.86871700
C	1.19048700	0.64057300	-0.09829800
H	0.32278000	1.01546600	1.93028600
C	-0.63434700	2.29910400	0.46860100
O	-0.70457200	2.73095000	-0.66159100
O	-1.36563300	2.78587700	1.48603800
C	2.32207700	1.64931800	-0.22912900
O	2.70285300	2.37641300	0.64838800
O	2.86849900	1.61996800	-1.44868700
C	3.94998600	2.52176700	-1.66480000
H	3.62202400	3.55068200	-1.51927100
H	4.77012500	2.31088300	-0.97822100
H	4.26470100	2.36515900	-2.69306800
C	-2.23766200	3.85921000	1.15624200
H	-1.67491900	4.71057200	0.77298200
H	-2.96320500	3.54925100	0.40296000
H	-2.74315200	4.12591500	2.08089400
C	1.71426200	-0.76331800	0.28266700
C	2.40107300	-1.47248800	-0.88505900
C	2.61682000	-0.78013900	1.51761600
H	0.81842600	-1.34725800	0.52760800
C	2.78009800	-2.90286500	-0.51302800

H	3.30066800	-0.91982700	-1.17148100
H	1.74355900	-1.46907500	-1.75796400
C	2.99920900	-2.20836700	1.89796300
H	3.52596300	-0.20582100	1.31051200
H	2.12953100	-0.28502400	2.35996200
C	3.66075600	-2.93860900	0.73278800
H	3.28721500	-3.38577800	-1.35116600
H	1.86674400	-3.47966900	-0.32501900
H	3.66307300	-2.19774200	2.76504900
H	2.09771400	-2.75367900	2.19975300
H	3.88481700	-3.97107700	1.01022400
H	4.61903700	-2.45778600	0.50694500
H	0.72597700	0.57085200	-1.08462900
H	-1.03511700	0.02263900	0.66046700
Sn	-2.29869400	-0.94238300	-0.21769300
C	-1.29447400	-1.88236000	-1.86909500
H	-0.83047200	-1.12792100	-2.50449200
H	-0.51900000	-2.55754700	-1.50621900
H	-2.00206600	-2.45549300	-2.46933900
C	-3.75375500	0.47543600	-0.90820500
H	-4.32426000	0.86974900	-0.06724300
H	-3.24419300	1.30049800	-1.40594000
H	-4.44420100	0.00806800	-1.61101600
C	-3.19467100	-2.43221500	1.04550600
H	-2.44012600	-3.13202600	1.40407000
H	-3.67511900	-1.96568000	1.90497700
H	-3.94682000	-2.98875400	0.48493900

E(UwB97XD, vacuo)	-1104.374394
Zero-point correction=	0.416724
Thermal correction to Energy=	0.443731
Thermal correction to Enthalpy=	0.444675
Thermal correction to Gibbs Free Energy=	**0.354341**
E(UwB97XD, MeCN)	**-1104.385995**

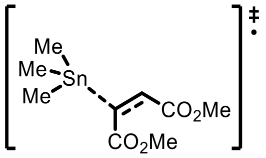

TS4$_{Sn}$

C	-1.60370200	-0.46911500	-1.44618900
C	-0.79541000	0.59051600	-1.59377000
H	-1.67228700	-1.18413400	-2.25770600
C	-2.33816900	-0.85895200	-0.22075000
O	-1.89125200	-0.84491000	0.89612000
O	-3.55546300	-1.32184600	-0.52567700
C	-0.71710700	1.81576700	-0.74721800
O	0.13835300	2.65305300	-0.88186300
O	-1.72395100	1.92092400	0.11530600
C	-1.68291900	3.04446000	0.98942500
H	-0.77550600	3.01996300	1.59266200
H	-1.71163100	3.97414900	0.42160700

H	-2.56086200	2.95541000	1.62306300
C	-4.33136900	-1.79300700	0.57297300
H	-3.82503900	-2.61837900	1.07359800
H	-4.50036500	-0.99256200	1.29304600
H	-5.27388700	-2.12653500	0.14760000
H	-0.24128500	0.69950400	-2.51729700
Sn	1.74490700	-0.37777300	0.00903500
C	1.28912100	-2.47724400	0.25083800
H	1.98198200	-2.91479900	0.97271700
H	0.27222700	-2.57872500	0.62759100
H	1.38327900	-3.01406000	-0.69264000
C	3.85861300	-0.14450100	-0.40986300
H	4.42711100	-0.51491500	0.44629800
H	4.14348000	-0.71168300	-1.29507000
H	4.09990100	0.90589100	-0.56665300
C	1.27061700	0.66304400	1.83812400
H	0.21579600	0.51222500	2.06541300
H	1.87462100	0.26695500	2.65685900
H	1.47758500	1.72675000	1.72467300

E(UwB97XD, vacuo)	-868.464783
Zero-point correction=	0.247208
Thermal correction to Energy=	0.267722
Thermal correction to Enthalpy=	0.268666
Thermal correction to Gibbs Free Energy=	**0.193368**
E(UwB97XD, MeCN)	**-868.473878**

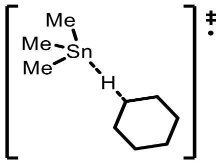

TS5$_{Sn}$

C	-2.62894000	1.28228600	0.50515500
C	-2.51239400	1.24292900	-1.01918300
C	-1.87060700	-0.01836600	-1.50831100
C	-2.31734400	-1.31128500	-0.89794200
C	-2.44266600	-1.22436600	0.62391800
C	-3.24891100	-0.00106700	1.05035800
H	-3.52296700	1.32510100	-1.45035800
H	-1.95952700	2.11382100	-1.38168700
H	-1.63181700	1.40696900	0.93886400
H	-3.21698100	2.15004800	0.81157700
H	-3.29843900	-1.58922800	-1.31428600
H	-1.63257000	-2.11701200	-1.17735100
H	-2.89773300	-2.13772000	1.01293900
H	-1.44243000	-1.16087700	1.06600600
H	-4.27549700	-0.09586800	0.67755400
H	-3.31371000	0.04555000	2.13984700
H	-1.61527200	-0.04957200	-2.56369900
H	-0.25933300	0.11060900	-0.91898400
C	1.84101400	-2.04434000	0.25712000
H	1.04055000	-2.57190500	0.77690200

H	2.01133500	-2.53426200	-0.70176900
H	2.75125800	-2.12351500	0.85312000
C	2.93201500	1.02275900	-1.03670000
H	3.10075800	0.58406000	-2.02015700
H	2.71142800	2.08270800	-1.16336900
H	3.84816200	0.92668300	-0.45229500
C	1.07060300	0.91212700	1.92362500
H	0.82534700	1.97127900	1.83981700
H	0.26844900	0.41511700	2.47061600
H	1.99250800	0.81499000	2.49865900
Sn	1.31159700	0.02537900	-0.02660400

E(UwB97XD, vacuo)	-569.938087
Zero-point correction=	0.271328
Thermal correction to Energy=	0.287057
Thermal correction to Enthalpy=	0.288001
Thermal correction to Gibbs Free Energy=	**0.224891**
E(UwB97XD, MeCN)	**-569.939353**

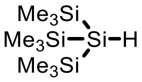

Si$_H$

C	1.51258900	1.70768100	1.77082500
H	1.39938100	0.79959700	2.36692900
H	2.35760700	2.27144600	2.17438400
H	0.61292800	2.31168700	1.90843700
C	3.41487500	0.37552200	-0.21982000
H	3.59919400	0.09073100	-1.25782500
H	4.25247700	0.99365500	0.11377900
H	3.41256100	-0.53662200	0.38106400
C	1.89213300	2.91176700	-1.01892300
H	2.07016800	2.71792000	-2.07858000
H	0.96502000	3.48261900	-0.93543700
H	2.70845300	3.53726300	-0.64819600
Si	-0.00009400	0.00042100	-0.81195200
Si	1.79015100	1.30699800	-0.04676500
Si	-2.02736600	0.89645300	-0.04702100
Si	0.23723600	-2.20315300	-0.04685600
C	-3.46690300	0.18274900	-1.02119900
H	-3.49701700	-0.90566300	-0.93856000
H	-4.41735500	0.57599800	-0.65097900
H	-3.38724600	0.43470900	-2.08059000
C	-2.23685400	0.45334000	1.76981300
H	-3.14700600	0.90418900	2.17367000
H	-2.31186200	-0.62783100	1.90600000
H	-1.39349200	0.80715300	2.36670800
C	-2.03372300	2.76948600	-0.21725400
H	-1.24441700	3.22310000	0.38633800
H	-1.87707500	3.07339400	-1.25437400
H	-2.98888700	3.18459500	0.11489400
C	0.72196500	-2.16340800	1.77106200
H	-0.00616000	-1.60785500	2.36599800

H	0.78309800	-3.17698000	2.17559900
H	1.69667900	-1.69014000	1.90931900
C	-1.38102800	-3.14578000	-0.22035200
H	-1.72094000	-3.16158800	-1.25800500
H	-1.26310400	-4.18067600	0.11136900
H	-2.16961800	-2.68956300	0.38222600
C	1.57710400	-3.09238400	-1.01877600
H	1.31971200	-3.15124300	-2.07822700
H	2.53399900	-2.57287100	-0.93638700
H	1.71287800	-4.11141400	-0.64700200
H	0.00010300	0.00047200	-2.30847200

E(UwB97XD, vacuo)	-1517.991469
Zero-point correction=	0.345469
Thermal correction to Energy=	0.371220
Thermal correction to Enthalpy=	0.372165
Thermal correction to Gibbs Free Energy=	**0.291515**
E(UwB97XD, MeCN)	**-1517.993609**

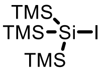

S_{Si} *

Si	-0.15255600	0.00055600	-0.00019400
Si	-0.86148700	-1.77291000	1.36289200
Si	-0.84791000	2.07435100	0.84940900
Si	-0.83888600	-0.29401000	-2.22465500
C	-0.62890800	-3.39934500	0.45501100
H	0.41230800	-3.53226600	0.15372300
H	-0.90613200	-4.24211200	1.09333100
H	-1.24754300	-3.44199500	-0.44411500
C	0.10775700	-1.79343500	2.96690900
H	-0.23442600	-2.60867300	3.60961600
H	1.17326600	-1.93460200	2.77644300
H	-0.01201300	-0.85659700	3.51444200
C	-2.68806000	-1.50873100	1.72884100
H	-3.08866000	-2.34769200	2.30359300
H	-2.84458300	-0.59989800	2.31417300
H	-3.27355200	-1.42171000	0.81099800
C	0.13548400	-1.67712800	-3.03126500
H	-0.19504800	-1.82655000	-4.06238800
H	1.20210300	-1.44570300	-3.04601000
H	0.00572500	-2.61843100	-2.49370800
C	-2.66652000	-0.73980700	-2.19576200
H	-3.05800800	-0.81988900	-3.21310100
H	-2.82838600	-1.69986500	-1.70052400
H	-3.25599500	0.01334700	-1.66842600
C	-0.59200200	1.30255300	-3.18053900
H	0.44921500	1.62838200	-3.13280300
H	-0.85592700	1.16822900	-4.23260600
H	-1.21464900	2.10476500	-2.77855900
C	0.12961100	3.46496400	0.05948400
H	0.01127700	3.46551200	-1.02576900

H	-0.20789600	4.43305600	0.43849300
H	1.19431500	3.36620500	0.27881500
C	-0.61435900	2.10642300	2.71167800
H	0.42653100	1.91045600	2.97746000
H	-0.88922400	3.08252300	3.11939500
H	-1.23440600	1.35201700	3.20093200
C	-2.67343100	2.26929000	0.43762100
H	-3.26488600	1.43833600	0.82806400
H	-3.06670000	3.19278100	0.87029700
H	-2.83068500	2.31463400	-0.64235900
I	2.34694900	-0.00631700	0.01159200

E(UwB97XD, vacuo) -1815.233921
Zero-point correction= 0.338909
Thermal correction to Energy= 0.366336
Thermal correction to Enthalpy= 0.367281
Thermal correction to Gibbs Free Energy= **0.281117**

E(UwB97XD, MeCN) **-1815.237601**

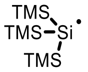

$\mathbf{Si_R^{\bullet}}$ *

Si	0.00022700	0.00109600	-0.69018000
Si	1.99994300	1.02841100	-0.05921200
Si	-1.89148600	1.21585900	-0.05880100
Si	-0.10833700	-2.24395500	-0.05866800
C	3.43448100	-0.16918700	-0.26581900
H	3.52668300	-0.50361500	-1.30110900
H	4.37608400	0.30646300	0.02061800
H	3.30294200	-1.05382000	0.36138700
C	2.31983800	2.57117500	-1.08209500
H	3.23494400	3.06897300	-0.75049500
H	2.43165600	2.32709800	-2.14024200
H	1.49634500	3.28219600	-0.98823800
C	1.88220200	1.52221300	1.75513100
H	2.81618000	1.98106800	2.09120500
H	1.07759200	2.24338900	1.91522900
H	1.68394700	0.65482000	2.38810600
C	1.06796500	-3.29225400	-1.08155300
H	1.04384800	-4.33307800	-0.74788600
H	0.79901800	-3.26942400	-2.13931800
H	2.09503900	-2.93249900	-0.99003500
C	0.38062600	-2.38559200	1.75498500
H	0.31430700	-3.42359900	2.09277500
H	1.40684800	-2.04621200	1.91303300
H	-0.27219300	-1.78058400	2.38784300
C	-1.86265500	-2.88895300	-0.26394800
H	-2.19710800	-2.80604700	-1.30000700
H	-1.92229400	-3.94087300	0.02735800
H	-2.56354900	-2.32921200	0.35955600
C	-3.38424500	0.72078200	-1.08623400
H	-3.58521100	-0.34868500	-0.99625000

H	-4.27508100	1.26061300	-0.75438400
H	-3.22754600	0.94343500	-2.14338600
C	-1.57495000	3.05821700	-0.26066200
H	-1.33173700	3.30828500	-1.29526900
H	-2.45857300	3.63383700	0.02716400
H	-0.74361500	3.38620900	0.36747500
C	-2.26381700	0.86139000	1.75376500
H	-1.41483500	1.12164500	2.38940900
H	-3.12904600	1.43972500	2.08964000
H	-2.48618700	-0.19662200	1.91045700

E(UwB97XD, vacuo)	-1517.349336
Zero-point correction=	0.337358
Thermal correction to Energy=	0.362914
Thermal correction to Enthalpy=	0.363858
Thermal correction to Gibbs Free Energy=	**0.281916**
E(UwB97XD, MeCN)	**-1517.351429**

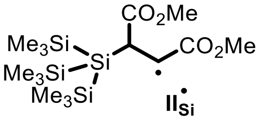

II•Si

C	-1.80137200	-0.11748400	-1.57870700
C	-0.61302900	0.70949100	-1.34132000
H	-1.89524200	-0.65285200	-2.51420300
C	-2.81719700	-0.36904800	-0.58408200
O	-2.78604500	0.00243400	0.56976200
O	-3.82312100	-1.11271900	-1.08359900
C	-0.81799500	2.15882100	-0.97547200
O	0.08965200	2.94295400	-0.85437600
O	-2.09563900	2.49886400	-0.83285700
C	-2.34232600	3.84283200	-0.42884100
H	-1.86441900	4.04280400	0.53008400
H	-1.96187200	4.54367900	-1.17152700
H	-3.42176800	3.92829100	-0.33988100
C	-4.83518200	-1.47634900	-0.15496600
H	-4.41559900	-2.07640800	0.65379400
H	-5.30640600	-0.59064800	0.27181500
H	-5.56058000	-2.05775200	-0.71802900
H	-0.01128300	0.73303100	-2.25246100
C	1.82899900	0.10226400	3.09946800
H	2.52339500	0.88732400	2.79834500
H	1.68169100	0.18146300	4.17992300
H	2.30523600	-0.86135200	2.90135200
C	-0.93387000	-1.09350600	2.95301100
H	-1.04910600	-0.91372100	4.02560500
H	-1.92106600	-1.08332600	2.49281300
H	-0.49992000	-2.08838300	2.83103400
C	-0.57940600	1.92819000	2.47638200
H	0.04480200	2.70851700	2.03618700
H	-1.56561400	1.96477500	2.00946800
H	-0.69571800	2.13912700	3.54242400

Si	0.64886800	-0.12099100	-0.06726300
Si	0.17061300	0.23098700	2.21183300
Si	0.66940800	-2.43767600	-0.47812800
Si	2.76202900	0.76267600	-0.65169000
C	1.80867500	-3.23660000	0.78827700
H	1.86400200	-4.31443800	0.61407800
H	2.82203200	-2.83634500	0.74275600
H	1.43368700	-3.08437100	1.80271100
C	1.31094100	-2.79213300	-2.21138300
H	1.33035400	-3.86729600	-2.40718900
H	0.67545600	-2.32796400	-2.96943100
H	2.32418200	-2.40771700	-2.34623700
C	-1.02947800	-3.22248100	-0.28641600
H	-0.93336900	-4.31165400	-0.27599800
H	-1.50161200	-2.91635500	0.64938100
H	-1.70084300	-2.95576900	-1.10421700
C	4.10680600	-0.47748900	-0.20473500
H	3.99992300	-1.41512300	-0.75352100
H	5.08832700	-0.06026900	-0.44513700
H	4.09654200	-0.70679000	0.86317100
C	3.16957600	2.38677600	0.19203600
H	3.26251100	2.27442100	1.27320000
H	4.12488800	2.76356100	-0.18425400
H	2.39842600	3.12847100	-0.01326000
C	2.77580200	1.02723300	-2.51408200
H	2.08404200	1.82602500	-2.78990000
H	3.77378500	1.32041900	-2.84981300
H	2.49384000	0.12130700	-3.05608300

E(UwB97XD, vacuo)	-2051.776146
Zero-point correction=	0.479539
Thermal correction to Energy=	0.516185
Thermal correction to Enthalpy=	0.517130
Thermal correction to Gibbs Free Energy=	**0.412098**
E(UwB97XD, MeCN)	**-2051.784085**

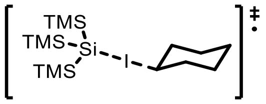

$TS1_{Si}$ *

Si	-1.58622300	0.00121400	-0.04212800
Si	-2.13863700	2.24761300	-0.36413900
Si	-1.91645800	-1.33138100	-1.93194300
Si	-2.44919900	-0.91885500	1.92348900
C	-1.43419000	-0.36819500	-3.47135500
H	-2.06233100	0.51669300	-3.59623900
H	-0.39511800	-0.03723700	-3.41321000
H	-1.54393000	-0.98657500	-4.36600200
C	-3.73762000	-1.79856900	-2.03567500
H	-3.93356400	-2.39756000	-2.92918800
H	-4.04133000	-2.38557000	-1.16589800
H	-4.37279000	-0.91113100	-2.07796800

C	-0.88316000	-2.89361300	-1.81894400
H	-1.04283500	-3.52543100	-2.69666200
H	0.17968700	-2.65139800	-1.75953700
H	-1.14202500	-3.47441100	-0.93141800
C	-0.93523600	3.04981700	-1.55988900
H	-0.94680600	2.54624700	-2.52854800
H	-1.19386200	4.09984300	-1.72006300
H	0.08446000	3.00313000	-1.17270200
C	-3.87954700	2.34836500	-1.07470200
H	-4.18149500	3.38991500	-1.21449900
H	-3.93571900	1.85025600	-2.04530400
H	-4.60582200	1.87211000	-0.41252500
C	-2.08586200	3.15054200	1.28242000
H	-1.10067400	3.05889800	1.74454500
H	-2.30061600	4.21394600	1.14876800
H	-2.82287300	2.74463700	1.97893700
C	-1.62678700	-0.14573700	3.42242500
H	-1.78528600	0.93423500	3.44613100
H	-2.03232700	-0.56861000	4.34536000
H	-0.54981400	-0.32383500	3.40820200
C	-2.15327100	-2.77414000	1.92878200
H	-2.67333600	-3.25907500	1.09960100
H	-1.08863000	-2.99839000	1.83493700
H	-2.51386400	-3.22165200	2.85857700
C	-4.30116200	-0.58461200	1.99049600
H	-4.74277700	-1.03189800	2.88519700
H	-4.50546600	0.48816000	2.01694100
H	-4.81065200	-1.00026100	1.11849900
C	5.68814100	-1.26390400	0.04795200
C	4.15853800	-1.25957300	-0.06266800
C	3.59639900	-0.00371600	0.56619200
C	4.16592800	1.26261400	-0.03433000
C	5.69557900	1.25522600	0.07610300
C	6.29160100	0.00079000	-0.55641200
H	3.87512800	-1.29386500	-1.11979200
H	3.73989600	-2.14921300	0.41058400
H	5.97284500	-1.33263400	1.10336800
H	6.08839800	-2.15295500	-0.44471600
H	3.88244400	1.32268800	-1.09023300
H	3.75296100	2.14381400	0.45931800
H	6.10114800	2.15264900	-0.39663800
H	5.98071000	1.29863500	1.13275200
H	6.09439400	0.01339800	-1.63402700
H	7.37710000	-0.00374000	-0.43532400
H	3.70071200	-0.01621700	1.65145500
I	1.30953200	0.00453500	0.34171800

E(UwB97XD, vacuo)	-2050.439538
Zero-point correction=	0.498400
Thermal correction to Energy=	0.532729
Thermal correction to Enthalpy=	0.533673
Thermal correction to Gibbs Free Energy=	**0.427393**
E(UwB97XD, MeCN)	**-2050.443391**

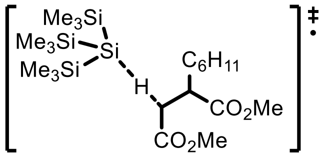

TS3$_{Si}$

C	-1.02126700	1.34097400	-0.62941600
C	-2.14556000	0.75696800	0.17399600
H	-0.99666700	1.14466600	-1.69318000
C	-0.53574000	2.65738200	-0.23057600
O	-0.75046200	3.18755600	0.83641400
O	0.23253400	3.21940500	-1.18237800
C	-3.42563500	1.51244400	-0.16000400
O	-3.80443500	1.76640000	-1.27319500
O	-4.09876300	1.85419800	0.94085900
C	-5.32278600	2.55095900	0.72865500
H	-5.14297000	3.48426500	0.19567700
H	-6.01684500	1.94069500	0.15011400
H	-5.72480700	2.74998400	1.71828000
C	0.75854000	4.50356100	-0.86904000
H	-0.04629600	5.20907000	-0.66324900
H	1.41512000	4.45226900	0.00026600
H	1.32137000	4.81517000	-1.74535500
C	-2.34263000	-0.76482700	-0.01292700
C	-3.49771800	-1.27696400	0.85171700
C	-2.51121900	-1.22248000	-1.46244600
H	-1.42193800	-1.22460000	0.36208700
C	-3.64953600	-2.79101900	0.76434500
H	-4.43224200	-0.81205400	0.51829000
H	-3.35354900	-0.96370900	1.88902700
C	-2.68351200	-2.73822100	-1.54943100
H	-3.37779500	-0.72454400	-1.90655700
H	-1.64277400	-0.92430200	-2.05546100
C	-3.83463500	-3.23542600	-0.68250800
H	-4.49394400	-3.11630400	1.37589000
H	-2.75690700	-3.27088600	1.18068000
H	-2.83217500	-3.03376000	-2.59038500
H	-1.76137200	-3.22696600	-1.21624600
H	-3.90882000	-4.32354700	-0.74127900
H	-4.77845600	-2.83183900	-1.06534000
H	-1.94177700	0.94170900	1.22969800
H	0.23727700	0.45556100	-0.21499400
C	2.59823700	2.34420800	1.99860100
H	3.31267900	3.14807900	2.19605900
H	1.62814000	2.79274500	1.77547400
H	2.48685000	1.76694100	2.91994000
C	4.81409700	0.41687600	1.14450000
H	5.17677600	-0.27971400	0.38599900
H	5.58662000	1.17543000	1.29813200
H	4.69702000	-0.13539400	2.07754100
C	3.58256700	2.25076500	-0.93306200
H	4.11845500	1.64241900	-1.66555900
H	2.67112000	2.61622800	-1.40611600
H	4.21437400	3.10858300	-0.68788000
Si	1.58769600	-0.38140700	0.10827200

Si	1.12427000	-1.75573300	1.95678500
Si	2.10672100	-1.38814500	-1.94457800
Si	3.20975100	1.24081800	0.60843500
C	0.44762200	-3.42511900	1.41768700
H	1.22265700	-4.01707600	0.92640900
H	0.09458300	-3.98984900	2.28467900
H	-0.38518100	-3.32394600	0.71983200
C	-0.12425100	-0.89596200	3.06750700
H	0.18413200	0.12646900	3.29542300
H	-1.11092200	-0.84861400	2.60459100
H	-0.22529500	-1.43786000	4.01162000
C	2.68891600	-2.05089800	2.95707700
H	3.04206000	-1.12489600	3.41525300
H	2.48897000	-2.76482500	3.76062100
H	3.49757500	-2.45209100	2.34310500
C	1.12688200	-2.95766200	-2.25878800
H	1.41539200	-3.39206100	-3.21972200
H	1.29289200	-3.70886400	-1.48525900
H	0.05708300	-2.74711800	-2.29794700
C	1.69522400	-0.15291000	-3.29948700
H	0.62354600	0.05669700	-3.32288200
H	2.21359300	0.79582300	-3.15095800
H	1.98012200	-0.54703800	-4.27856800
C	3.94321500	-1.79121900	-2.00582600
H	4.19656300	-2.27488600	-2.95285900
H	4.54932200	-0.88696000	-1.91987800
H	4.23245600	-2.46564000	-1.19685500

E(UwB97XD, vacuo)	-2287.654884
Zero-point correction=	0.648054
Thermal correction to Energy=	0.691470
Thermal correction to Enthalpy=	0.692415
Thermal correction to Gibbs Free Energy=	**0.571064**
E(UwB97XD, MeCN)	**-2287.666507**

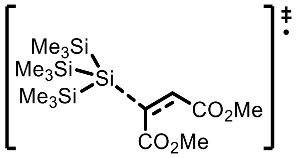

TS4$_{Si}$

C	2.03294100	0.32795700	-1.64725800
C	1.15946100	-0.68802200	-1.73335400
H	1.93706600	1.15102500	-2.34464100
C	3.00831000	0.54313700	-0.56447800
O	2.93314900	0.10224600	0.55395900
O	3.97086800	1.38881800	-0.95929500
C	1.23736500	-2.03996700	-1.10276900
O	0.28726300	-2.77390600	-0.99527600
O	2.47764600	-2.39339800	-0.78808100
C	2.63419900	-3.69250600	-0.22329300
H	2.05914200	-3.77844900	0.69788800
H	2.30234800	-4.45627400	-0.92656900

H	3.69616200	-3.79593400	-0.01931700
C	4.91708900	1.75495500	0.03948800
H	4.41869600	2.24989800	0.87350600
H	5.44268700	0.87590600	0.41226900
H	5.61142700	2.43559300	-0.44555800
H	0.40161500	-0.64875800	-2.50676600
C	-1.95609300	-0.48308000	3.35505400
H	-2.58088700	-1.30117300	2.99242100
H	-1.70722200	-0.68878400	4.40001700
H	-2.55060100	0.43255000	3.32465100
C	0.65817700	1.06850600	3.06347000
H	0.91713200	0.83552000	4.09988200
H	1.58504900	1.18296800	2.49897600
H	0.12382900	2.02111500	3.05860900
C	0.58394900	-1.91616900	2.39912600
H	0.05640800	-2.71989400	1.88192600
H	1.55355000	-1.76815000	1.91933600
H	0.75445300	-2.22254400	3.43454400
Si	-0.96524800	0.20090800	0.12777400
Si	-0.38343900	-0.31180300	2.33240100
Si	-0.96168000	2.46982400	-0.41025000
Si	-2.77097500	-0.95321000	-0.80293000
C	-2.29354400	3.35635400	0.58170800
H	-2.33221000	4.41631800	0.31539500
H	-3.27892800	2.92370400	0.39812100
H	-2.09311800	3.28635100	1.65318000
C	-1.33514800	2.66689100	-2.24469800
H	-1.34086800	3.72181200	-2.53085400
H	-0.58784700	2.15750800	-2.85760900
H	-2.31182900	2.24659200	-2.49483500
C	0.68898800	3.28999700	-0.03337300
H	0.61245700	4.36869000	-0.19541600
H	0.98627400	3.12435800	1.00336800
H	1.48914700	2.91710000	-0.67466100
C	-4.33042700	0.08495600	-0.59466000
H	-4.24149700	1.04267900	-1.11265800
H	-5.19715100	-0.43897800	-1.00725700
H	-4.53114500	0.29162000	0.45874300
C	-3.01073000	-2.61070700	0.04035000
H	-3.26283300	-2.49084600	1.09560600
H	-3.82291000	-3.16602400	-0.43648800
H	-2.09904000	-3.20507600	-0.03328000
C	-2.48403800	-1.22827300	-2.64012800
H	-1.66097700	-1.92956400	-2.78876500
H	-3.37759800	-1.65041200	-3.10760000
H	-2.24638100	-0.29478200	-3.15564500

E(UwB97XD, vacuo)	-2051.738286
Zero-point correction=	0.478219
Thermal correction to Energy=	0.515337
Thermal correction to Enthalpy=	0.516281
Thermal correction to Gibbs Free Energy=	**0.407580**
E(UwB97XD, MeCN)	**-2051.746034**

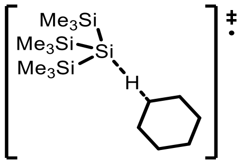

TS5$_{Si}$

C	3.40379100	-1.35581100	-0.39679600
C	2.88310500	-1.13753700	-1.81734000
C	2.20579300	0.19055100	-1.96762200
C	2.90734400	1.38793200	-1.40252100
C	3.42198600	1.13230700	0.01356800
C	4.22318300	-0.16448100	0.09249300
H	3.72982600	-1.19492000	-2.51915800
H	2.20040100	-1.94424200	-2.09818400
H	2.54913100	-1.49927600	0.27102200
H	3.99735300	-2.27139900	-0.35148800
H	3.75870700	1.65017900	-2.04975300
H	2.24198300	2.25574000	-1.41213200
H	4.02851700	1.97582100	0.35035100
H	2.56728900	1.06539300	0.69344900
H	5.12717200	-0.07034400	-0.52041400
H	4.55845300	-0.33559100	1.11853400
H	1.66644500	0.34898500	-2.89800200
H	0.91978100	0.04500600	-0.97550600
C	-2.90889600	2.28419200	0.70398100
H	-3.05372000	1.75390800	1.64745500
H	-3.24051800	3.31672900	0.84179100
H	-3.55990600	1.82042200	-0.04082900
C	-0.04510400	3.10544000	1.42776700
H	1.01222500	3.05847200	1.15656100
H	-0.32580800	4.15861700	1.51056700
H	-0.15634900	2.65085400	2.41485400
C	-0.95687500	3.11528200	-1.49569300
H	0.07597600	3.11600000	-1.84993900
H	-1.56985800	2.63482400	-2.26093500
H	-1.28289800	4.15484600	-1.40563500
Si	-0.41279500	0.00493700	-0.07849600
Si	-1.11049000	2.22264300	0.15269800
Si	-1.96872800	-1.23560900	-1.30545700
Si	-0.08676900	-0.98849400	2.01679000
C	-1.13324700	-2.77569100	-1.98986900
H	-0.71550200	-3.38761300	-1.18752700
H	-1.84163300	-3.39086500	-2.55089600
H	-0.31568200	-2.50507400	-2.66184700
C	-3.41538100	-1.75175800	-0.21788800
H	-4.16213900	-2.29915100	-0.79908500
H	-3.08580800	-2.39952700	0.59747200
H	-3.90476400	-0.88153500	0.22529300
C	-2.63148800	-0.21612000	-2.74151100
H	-3.17823500	0.65939700	-2.38400300
H	-1.82100800	0.13601500	-3.38340400
H	-3.31419900	-0.80927000	-3.35559200
C	-1.58930700	-0.62132100	3.09051900
H	-2.51495500	-0.94146000	2.60802300
H	-1.51212700	-1.13689400	4.05151600

H	-1.67120400	0.44924900	3.29237700
C	0.08552500	-2.85319000	1.83186900
H	0.92823000	-3.11327700	1.18751100
H	0.24802400	-3.32476000	2.80458300
H	-0.81505300	-3.28993600	1.39450400
C	1.43228800	-0.32742300	2.90825700
H	2.35663100	-0.60950200	2.40151400
H	1.41020500	0.76193400	2.98032700
H	1.47165500	-0.73065500	3.92385200

E(UwB97XD, vacuo)	-1753.217306
Zero-point correction=	0.502066
Thermal correction to Energy=	0.534554
Thermal correction to Enthalpy=	0.535498
Thermal correction to Gibbs Free Energy=	**0.438304**

E(UwB97XD, MeCN) **-1753.219316**

9. General procedures for preparative experiments

An ethyl acetate (0.2 M) solution containing the electron-poor olefin **2** (1 mmol), organic halide **1** (1 equiv.), **B1** (1 equiv.) was prepared in a 7 mL vial equipped with a screw cap and a stirring bar. The solution was bubbled with N_2 (5 min) and then irradiated by adopting the setup shown in **Figure S1** equipped with a 40 W Kessil lamp (λ = 390 nm, full intensity) for 12 h (see each case for details). After irradiation, the mixture was collected, solvent was removed under reduced pressure and the crude was purified via column chromatography on silica gel to provide the expected product.

10. Characterization data

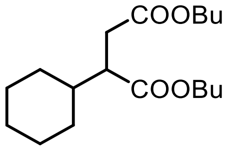

Dibutyl 2-cyclohexylsuccinate (3). Purified by flash column chromatography on silica gel (Cyclohexane:Ethyl Acetate 90:10) to afford the product as colorless oil (253 mg, 81%). ^{1}H NMR (300 MHz, $CDCl_3$) δ 4.15 – 4.0 (m, 4H), 2.77 – 2.60 (m, 2H), 2.49 – 2.35 (m, 1H), 1.79 – 1.52 (m, 10H), 1.45 – 1.29 (m, 4H), 1.28 – 0.98 (m, 5H), 0.92 (td, J_1 = 7 Hz, J_2 = 3 Hz, 6H). ^{13}C NMR (75 MHz, $CDCl_3$) δ 174.7, 172.8, 64.6, 64.4, 47.2, 40.2, 33.6, 30.8, 30.8, 30.7, 30.2, 26.5 (2C), 26.3, 19.3, 19.2, 13.8 (2C). Spectroscopic data are in accordance with the literature.[10]

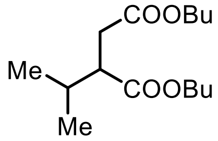

Dibutyl 2-isopropylsuccinate (4). Purified by flash column chromatography on silica gel (Cyclohexane:Ethyl Acetate 90:10) to afford the product as colorless oil (218 mg, 87%). ^{1}H NMR (300 MHz, $CDCl_3$) δ 4.15 – 3.96 (m, 4H), 2.77 – 2.58 (m, 2H), 2.44 – 2.30 (m, 1H), 2.04 – 1.88 (m, 1H), 1.66 – 1.49 (m, 4H), 1.44 – 1.25 (m, 4H), 0.90 – 0.79 (m, 12H). ^{13}C NMR (75 MHz, $CDCl_3$) δ 174.5, 172.6, 64.5, 64.4, 47.6, 33.2, 30.8, 30.7, 30.2, 20.1, 19.6, 19.2, 19.2, 13.8. HRMS (CI) m/z calcd for $C_{15}H_{29}O_4^+$: 273.2060 [M+H]$^+$; found: 273.2060.

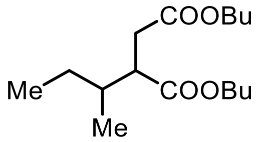

Dibutyl 2-(sec-butyl)succinate (5). Purified by flash column chromatography on silica gel (Cyclohexane:Ethyl Acetate 90:10) to afford the product as mixture of diastereomers as a colorless oil (229 mg, 80%, dr ~ 1:1). ^{1}H NMR (300 MHz, $CDCl_3$) δ 4.13 – 3.96 (m, 8H), 2.89 – 2.77 (m, 2H), 2.76 – 2.6 (m, 2H), 2.38 – 2.22 (m, 2H), 1.85 – 1.48 (m, 10H), 1.46 – 1.25 (m, 10H), 1.25 – 1.05 (m, 2H), 0.97 – 0.76 (m, 24H). ^{13}C NMR (75 MHz, $CDCl_3$) δ 174.7, 174.3, 172.7, 172.6, 64.5, 64.4, 64.3, 45.9, 45.7, 37.0, 36.5, 33.7, 31.8, 30.7, 30.7, 27.2, 26.8, 19.2, 19.2, 19.2, 16.5, 15.8, 13.7, 11.8, 11.8. HRMS (FI) m/z calcd for $C_{16}H_{30}O_4$: 286.2144; found: 286.2139.

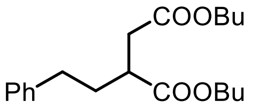

Dibutyl 2-phenethylsuccinate (6). Purified by flash column chromatography on silica gel (Cyclohexane:Ethyl Acetate 90:10) to afford the product as colorless oil (311 mg, 93%). ^{1}H NMR (300 MHz, $CDCl_3$) δ 7.33 – 7.24 (m, 2H), 7.23 – 7.12 (m, 3H), 4.19 – 4.02 (m, 4H), 2.94 – 2.83 (m, 1H), 2.76 (dd, J_1 = 16 Hz, J_2 = 9 Hz, 1H), 2.70 – 2.55 (m, 2H), 2.47 (dd, J_1 = 16 Hz, J_2 = 5 Hz, 1H), 2.07 – 1.90 (m, 1H), 1.90 – 1.75 (m, 1H), 1.71 – 1.52 (m, 4H), 1.49 – 1.24 (m, 4H), 0.93 (td, J_1 = 7 Hz, J_2 = 6 Hz, 6H). ^{13}C NMR (75 MHz, $CDCl_3$) δ 174.8, 172.0, 141.4, 128.6, 128.5, 126.2, 64.7, 64.7, 41.2, 36.3, 33.8, 33.4, 30.8, 30.7, 19.3, 19.2, 13.8, 13.8. HRMS (FI) m/z calcd for $C_{20}H_{30}O_4$: 334.2144; found: 334.2139.

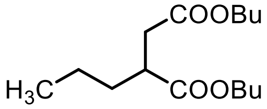

Dibutyl 2-butylsuccinate (7). Purified by flash column chromatography on silica gel (Cyclohexane:Ethyl Acetate 90:10) to afford the product as colorless oil (232 mg, 81%). ^{1}H NMR (300 MHz, $CDCl_3$) δ 4.16 – 4.00 (m, 4H), 2.88 – 2.76 (m, 1H), 2.69 (dd, J_1 = 16 Hz, J_2 = 9 Hz, 1H), 2.40 (dd, J_1 = 16 Hz, J_2 = 5 Hz, 1H), 1.72 – 1.45 (m, 6H), 1.45 – 1.20 (m, 8H), 0.99 – 0.82 (m, 9H). ^{13}C NMR (75 MHz, $CDCl_3$) δ 175.2, 172.2, 64.6, 64.5, 41.4, 36.3, 31.8, 30.8, 30.8, 29.2, 22.6, 19.3, 19.2, 14.0, 13.8 (2C). HRMS (CI) m/z calcd for $C_{16}H_{31}O_4^+$: 287.2217 $[M+H]^+$; found: 287.2226.

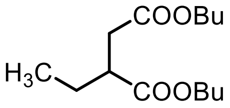

Dibutyl 2-ethylsuccinate (8). Purified by flash column chromatography on silica gel (Cyclohexane:Ethyl Acetate 90:10) to afford the product as colorless oil (201 mg, 78%). ^{1}H NMR (300 MHz, CDCl$_3$) δ 4.16 – 3.97 (m, 4H), 2.83 – 2.60 (m, 2H), 2.47 – 2.31 (m, 1H), 1.72 – 1.48 (m, 6H), 1.42 – 1.26 (m, 4H), 0.90 (td, J_1 = 7 Hz, J_2 = 2 Hz, 9H). ^{13}C NMR (75 MHz, CDCl$_3$) δ 174.9, 172.2, 64.5, 64.5, 42.8, 35.8, 30.8, 30.7, 25.1, 19.2, 19.2, 13.8 (2C), 11.4. HRMS (CI) m/z calcd for $C_{14}H_{27}O_4^+$: 259.1904 [M+H]$^+$; found: 259.1915.

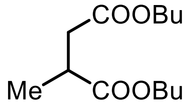

Dibutyl 2-methylsuccinate (9). **1g** (10 equiv.) was used. Purified by flash column chromatography on silica gel (Cyclohexane:Ethyl Acetate 90:10) to afford the product as colorless oil (159 mg, 65%). ^{1}H NMR (300 MHz, CDCl$_3$) δ 4.14 – 3.98 (m, 4H), 2.97 – 2.82 (m, 1H), 2.71 (dd, J_1 = 16 Hz, J_2 = 8 Hz, 1H), 2.37 (dd, J_1 = 16 Hz, J_2 = 6 Hz, 1H), 1.65 – 1.52 (m, 4H), 1.43 – 1.28 (m, 4H), 1.20 (d, J = 7 Hz, 3H), 0.91 (td, J_1 = 7 Hz, J_2 = 1 Hz, 6H). ^{13}C NMR (75 MHz, CDCl$_3$) δ 175.4, 172.0, 64.6, 64.6, 37.8, 36.0, 30.7 (2C), 19.2 (2C), 17.2, 13.8, 13.8. HRMS (FI) m/z calcd for $C_{13}H_{24}O_4$: 244.1675; found: 244.1670.

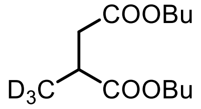

Dibutyl 2-methylsuccinate (d$_3$-9). **d$_3$-1g** (10 equiv.) was used. Purified by flash column chromatography on silica gel (Cyclohexane:Ethyl Acetate 90:10) to afford the product as colorless oil (166 mg, 67%). ^{1}H NMR (400 MHz, CDCl$_3$) δ 4.07 (td, J_1 = 6 Hz, J_2 = 4 Hz, 4H), 2.88 (t, J = 7 Hz, 1H), 2.72 (dd, J_1 = 16 Hz, J_2 = 8 Hz, 1H), 2.38 (dd, J_1 = 16 Hz, J_2 = 6 Hz, 1H), 1.64 – 1.54 (m, 4H), 1.42 – 1.30 (m, 4H), 0.92 (td, J_1 = 7 Hz, J_2 =2 Hz, 6H). ^{13}C NMR (101 MHz, CDCl$_3$) δ 175.5, 172.1, 64.7, 64.6, 37.8, 35.8, 30.8 (2C), 19.2 (2C), 16.9 – 15.8 (m), 13.8, 13.8.

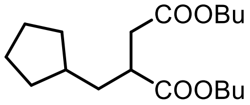

Dibutyl 2-(cyclopentylmethyl)succinate (10). Purified by flash column chromatography on silica gel (Cyclohexane:Ethyl Acetate 90:10) to afford the product as colorless oil (240 mg, 77%). ^{1}H NMR (300 MHz, CDCl$_3$) δ 4.16 – 3.98 (m, 4H), 2.83 – 2.76 (m, 1H), 2.67 (dd, J_1 = 16 Hz, J_2 = 9 Hz, 1H), 2.42 (dd, J_1 = 16 Hz, J_2 = 5 Hz, 1H), 1.88 – 1.22 (m, 17H), 1.17 – 0.99

(m, 2H), 0.91 (td, J_1 = 7 Hz, J_2= 2 Hz, 6H). ^{13}C NMR (75 MHz, $CDCl_3$) δ 175.5, 172.2, 64.6, 64.5, 41.0, 38.6, 38.0, 36.7, 32.8, 32.7, 30.7, 25.2, 25.1, 19.3, 19.2, 13.8. HRMS (FI) m/z calcd for $C_{18}H_{32}O_4$: 312.2301; found: 312.2292.

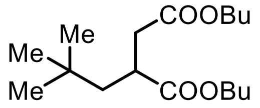

Dibutyl 2-neopentylsuccinate (11). 1i (2 equiv.) was used. Purified by flash column chromatography on silica gel (Cyclohexane:Ethyl Acetate 90:10) to afford the product as colorless oil (165 mg, 55%). ^{1}H NMR (300 MHz, $CDCl_3$) δ 4.12 – 3.97 (m, 4H), 2.90 – 2.75 (m, 1H), 2.64 (dd, J_1 = 16 Hz, J_2 = 8 Hz, 1H), 2.40 (dd, J_1 = 16 Hz, J_2 = 6 Hz, 1H), 1.75 (dd, J_1 = 14 Hz, J_2 = 8 Hz, 1H), 1.66 – 1.51 (m, 4H), 1.44 – 1.19 (m, 4H), 1.23 (dd, J_1 = 14 Hz, J_2 = 4 Hz, 1H), 0.99 – 0.84 (m, 15H). ^{13}C NMR (75 MHz, $CDCl_3$) δ 176.2, 171.8, 64.7 (2C), 45.8, 38.9, 38.4, 31.0, 30.7, 30.7, 29.5 (3C), 19.3, 19.2, 13.8 (2C). HRMS (FI) m/z calcd for $C_{17}H_{32}O_4$: 300.2301; found: 300.2305.

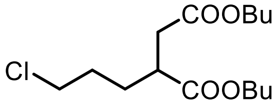

Dibutyl 2-(3-chloropropyl)succinate (12). Purified by flash column chromatography on silica gel (Cyclohexane:Ethyl Acetate 90:10) to afford the product as colorless oil (230 mg, 75%). ^{1}H NMR (300 MHz, $CDCl_3$) δ 4.17 – 4.01 (m, 4H), 3.61 – 3.47 (m, 2H), 2.92 – 2.80 (m, 1H), 2.73 (dd, J_1 = 16 Hz, J_2 = 9 Hz, 1H), 2.43 (dd, J_1 = 16 Hz, J_2 = 5 Hz, 1H), 1.91 – 1.67 (m, 4H), 1.67 – 1.53 (m, 4H), 1.47 – 1.30 (m, 4H), 0.93 (td, J_1 = 7 Hz, J_2 = 2 Hz, 6H). ^{13}C NMR (75 MHz, $CDCl_3$) δ 174.6, 171.9, 64.8, 64.8, 44.6, 40.8, 36.3, 30.8, 30.8, 30.1, 29.3, 19.3, 19.2, 13.8 (2C). HRMS (CI) m/z calcd for $C_{15}H_{28}ClO_4^+$: 307.1671 $[M+H]^+$; found: 307.1682.

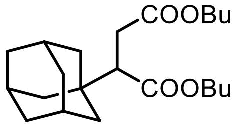

Dibutyl 2-((3r,5r,7r)-adamantan-1-yl)succinate (13). Purified by flash column chromatography on silica gel (Cyclohexane:Ethyl Acetate 90:10) to afford the product as colorless oil (306 mg, 84%). ^{1}H NMR (300 MHz, $CDCl_3$) δ 4.05 (dt, J_1 = 11 Hz, J_2 = 7 Hz, 4H), 2.73 (dd, J_1 = 17 Hz, J_2 = 12 Hz, 1H), 2.55 – 2.41 (m, 2H), 2.05 – 1.91 (m, 3H), 1.75 – 1.27 (m, 20H), 0.92 (td, J_1 = 7 Hz, J_2 = 5 Hz, 6H). ^{13}C NMR (75 MHz, $CDCl_3$) δ 173.9, 173.1, 64.6, 64.2, 52.6, 40.1 (3C), 36.9 (3C), 34.5, 31.4, 30.8, 30.8, 28.7 (3C), 19.4, 19.2, 13.8 (2C). HRMS (FD) m/z calcd for $C_{22}H_{36}O_4$: 364.2614; found: 364.2609.

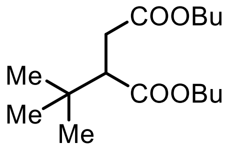

Dibutyl 2-(*tert*-butyl)succinate (14). Purified by flash column chromatography on silica gel (Cyclohexane:Ethyl Acetate 90:10) to afford the product as colorless oil (220 mg, 77%). ^{1}H NMR (300 MHz, $CDCl_3$) δ 4.14 – 3.98 (m, 4H), 2.77 (dd, J_1 = 16 Hz, J_2 = 12 Hz, 1H), 2.61 (dd, J_1 = 12 Hz, J_2 = 3 Hz, 1H), 2.45 (dd, J_1 = 16 Hz, J_2 = 3 Hz, 1H), 1.66 – 1.53 (m, 4H), 1.46 – 1.29 (m, 4H), 1.00 – 0.88 (m, 15H). ^{13}C NMR (75 MHz, $CDCl_3$) δ 174.4, 172.9, 64.7, 64.3, 51.5, 33.1, 32.7, 30.8, 30.8, 28.0 (3C), 19.4, 19.2, 13.8 (2C). HRMS (FI) m/z calcd for $C_{16}H_{30}O_4$: 286.2144; found: 286.2148.

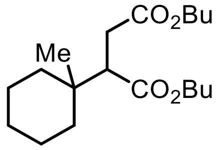

Dibutyl 2-(1-methylcyclohexyl)succinate (15). Purified by flash column chromatography on silica gel (Pentane:Ethyl Acetate 94:6) to afford the product as colorless oil (228 mg, 70%). ^{1}H NMR (300 MHz, $CDCl_3$) δ 4.11 – 3.99 (m, 4H), 2.83 – 2.64 (m, 2H), 2.51 – 2.36 (m, 1H), 1.74 – 1.17 (m, 18H), 0.93 – 0.84 (m, 9H). ^{13}C NMR (75 MHz, $CDCl_3$) δ 174.3, 173.0, 64.5, 64.2, 50.0, 36.4, 36.3, 35.2, 32.0, 30.7, 30.7, 26.1, 21.8, 21.8, 21.2, 19.3, 19.2, 13.8. HRMS (FI) m/z calcd for $C_{19}H_{34}O_4$: 326.2457; found: 326.2448.

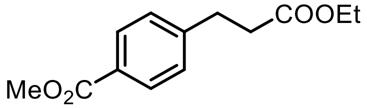

Dibutyl 2-(4-(methoxycarbonyl)phenyl)succinate (16). Purified by flash column chromatography on silica gel (Pentane:Ethyl Acetate 95:5 → 80:20) to afford the product as colorless oil (90 mg, 38%). ^{1}H NMR (300 MHz, $CDCl_3$) δ 8.02 – 7.91 (m, 2H), 7.33 – 7.23 (m, 2H), 4.13 (q, J = 7 Hz, 2H), 3.90 (s, 3H), 3.01 (t, J = 8 Hz, 2H), 2.64 (t, J = 8 Hz, 2H), 1.23 (t, J = 7 Hz, 3H). ^{13}C NMR (75 MHz, $CDCl_3$) δ 172.6, 167.1, 146.1, 129.9 (2C), 128.5 (2C), 128.4, 60.6, 52.1, 35.5, 31.0, 14.3. Spectroscopic data are in accordance with the literature.[11]

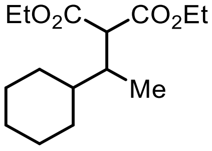

Diethyl 2-(1-cyclohexylethyl)malonate (17). Purified by flash column chromatography on silica gel (Pentane:Ethyl Acetate 94:6, second column: CH_2Cl_2) to afford the product as colorless oil (186 mg, 69%). ^{1}H NMR (300 MHz, $CDCl_3$) δ 4.25 – 4.10 (m, 4H), 3.37 (d, J = 9

Hz, 1H), 2.23 – 2.10 (m, 1H), 1.84 – 1.67 (m, 2H), 1.67 – 1.52 (m, 3H), 1.37 – 1.07 (m, 11H), 0.99 – 0.85 (m, 1H), 0.91 (d, J = 7 Hz, 3H). ^{13}C NMR (75 MHz, $CDCl_3$) δ 169.4, 169.2, 61.2, 61.2, 55.9, 40.4, 38.7, 31.6, 27.5, 26.8, 26.7, 26.6, 14.2, 13.0. HRMS (EI) m/z calcd for $C_{15}H_{26}O_4$: 270.1831; found: 270.1835. Spectroscopic data are in accordance with the literature.[12]

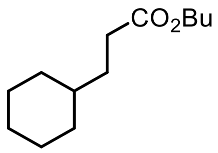

Methyl 3-cyclohexylbutanoate (18). Purified by flash column chromatography on silica gel (Pentane:Ethyl Acetate 94:6) to afford the product as colorless oil (156 mg, 74%). ^{1}H NMR (300 MHz, $CDCl_3$) δ 4.02 (t, J = 7 Hz, 2H), 2.33 – 2.19 (m, 2H), 1.72 – 1.43 (m, 9H), 1.41 – 1.27 (m, 2H), 1.26 – 1.01 (m, 4H), 0.95 – 0.75 (m, 5H). ^{13}C NMR (75 MHz, $CDCl_3$) δ 174.3, 64.1, 37.3, 33.0, 32.5, 32.0, 30.8, 26.6, 26.3, 19.2, 13.7. Spectroscopic data are in accordance with the literature.[13]

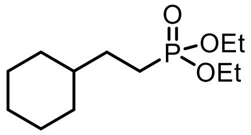

Diethyl (2-cyclohexylethyl)phosphonate (19). Purified by flash column chromatography on silica gel (Dichloromethane : Ethyl Acetate 80:20) to afford the product as colorless oil (142 mg, 57%). ^{1}H NMR (300 MHz, $CDCl_3$) δ 4.15 – 3.98 (m, 4H), 1.80 – 1.56 (m, 7H), 1.53 – 1.38 (m, 2H), 1.30 (t, J = 7 Hz, 6H), 1.26 – 1.01 (m, 4H), 0.99 – 0.76 (m, 2H). ^{13}C NMR (75 MHz, $CDCl_3$) δ 61.5, 61.4, 38.5, 38.3, 32.9, 29.7, 29.7, 26.6, 26.3, 24.2, 22.4, 16.6, 16.5. Spectroscopic data are in accordance with the literature.[14]

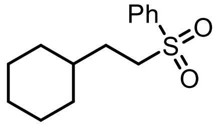

((2-Cyclohexylethyl)sulfonyl)benzene (20). Purified by flash column chromatography on silica gel (Heptane : Ethyl Acetate 90:10 → 75:25) to afford the product as light yellow oil (198 mg, 79%). ^{1}H NMR (300 MHz, $CDCl_3$) δ 7.99 – 7.84 (m, 2H), 7.73 – 7.63 (m, 1H), 7.63 – 7.53 (m, 2H), 3.19 – 3.04 (m, 2H), 1.79 – 1.51 (m, 7H), 1.37 – 1.01 (m, 4H), 0.98 – 0.78 (m, 2H). ^{13}C NMR (75 MHz, $CDCl_3$) δ 139.4, 133.7, 129.4, 128.2, 54.5, 36.8, 32.9, 29.7, 26.4, 26.1. Spectroscopic data are in accordance with the literature.[15]

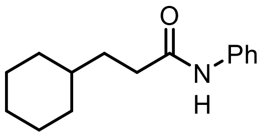

3-Cyclohexyl-*N*-phenylpropanamide (21). Purified by flash column chromatography on silica gel (Heptane:Ethyl Acetate 90:10 → 80:20) to afford the product as white solid (89 mg, 39%). ^{1}H NMR (300 MHz, CD_2Cl_2) δ 7.51 (d, *J* = 8 Hz, 2H), 7.46 – 7.35 (bs, 1H), 7.35 – 7.25 (m, 2H), 7.16 – 7.02 (m, 1H), 2.43 – 2.28 (m, 2H), 1.84 – 1.49 (m, 7H), 1.38 – 1.06 (m, 4H), 1.04 – 0.79 (m, 2H). ^{13}C NMR (75 MHz, CD_2Cl_2) δ 172.2, 138.9, 129.4, 124.4, 120.2, 37.9, 35.7, 33.7, 33.5, 27.1, 26.8. Spectroscopic data are in accordance with the literature.[16] M.p.: 92–94 °C (lit.[7] 91.9–95.4).

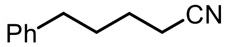

5-Phenylpentanenitrile (22). 2h (1.5 equiv.) was used. Purified by flash column chromatography on silica gel (100% Heptane → Heptane : Ethyl Acetate 90:10) to afford the product as colorless oil (77 mg, 48%). ^{1}H NMR (300 MHz, CD_2Cl_2) δ 7.34 – 7.25 (m, 2H), 7.23 – 7.16 (m, 3H), 2.66 (t, *J* = 7.3 Hz, 2H), 2.35 (t, *J* = 6.9 Hz, 2H), 1.86 – 1.59 (m, 4H). ^{13}C NMR (75 MHz, CD_2Cl_2) δ 142.2, 128.9, 128.9, 126.5, 120.3, 35.5, 30.9, 25.5, 17.5. Spectroscopic data are in accordance with the literature.[17]

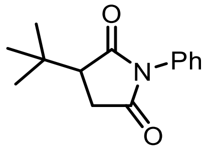

3-(*tert*-Butyl)-1-Phenylpyrrolidine-2,5-dione (23). Purified by flash column chromatography on silica gel (Heptane : Ethyl Acetate 90:10 → 50:50) to afford the product as colorless oil (71 mg, 31%). ^{1}H NMR (400 MHz, CD_2Cl_2) δ 7.55 – 7.48 (m, 2H), 7.48 – 7.41 (m, 1H), 7.30 – 7.25 (m, 2H), 2.91 (dd, J_1 = 18 Hz, J_2 = 9 Hz, 1H), 2.85 – 2.79 (m, 1H), 2.73 (dd, J_1 = 18 Hz, J_2 = 4 Hz, 1H), 1.15 (d, *J* = 1 Hz, 9H). ^{13}C NMR (101 MHz, CD_2Cl_2) δ 178.0, 176.0, 133.0, 129.6, 129.0, 127.4, 50.5, 34.2, 32.6, 27.5. Spectroscopic data are in accordance with the literature.[18]

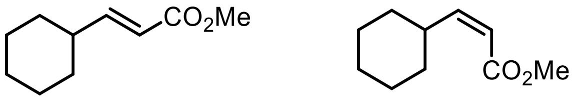

Major Minor

Methyl (*Z*)-3-cyclohexylacrylate and methyl (*E*)-3-cyclohexylacrylate (24). 2j (1.5 equiv.) was used. Purified by flash column chromatography on silica gel (Heptane : Ethyl Acetate 95:5) to afford the product as colorless oil (*Z*-isomer 34 mg, 21%, *E*-isomer 57 mg, 34%).

Major: ^{1}H NMR (300 MHz, $CDCl_3$) δ 6.91 (dd, J_1 = 16 Hz, J_2 = 7 Hz, 1H), 5.75 (dd, J_1 = 16 Hz, J_2 = 2 Hz, 1H), 3.71 (s, 3H), 2.19 – 2.05 (m, 1H), 1.86 – 1.54 (m, 5H), 1.44 – 1.02 (m, 5H). ^{13}C NMR (75 MHz, $CDCl_3$) δ 167.7, 154.7, 118.6, 51.5, 40.5, 31.8, 26.0, 25.8.

Minor: ^{1}H NMR (300 MHz, $CDCl_3$) δ 6.02 (dd, J_1 = 12 Hz, J_2 = 10 Hz, 1H), 5.65 (dd, J_1 = 12 Hz, J_2 = 1 Hz, 1H), 3.69 (s, 3H), 3.42 – 3.16 (m, 1H), 1.83 – 1.57 (m, 5H), 1.45 – 0.96 (m, 5H). ^{13}C NMR (75 MHz, $CDCl_3$) δ 166.9, 156.1, 117.3, 51.1, 37.5, 32.5, 26.0, 25.6.

Spectroscopic data are in accordance with the literature.[19]

11. References

(1) Masson, T. M.; Zondag, S. D. A.; Schuurmans, J. H. A.; Noël, T. Open-source 3D printed reactors for reproducible batch and continuous-flow photon-induced chemistry: design and characterization. *Reaction Chemistry & Engineering* **2024**. DOI: 10.1039/d4re00081a.

(2) Gardner, S.; Kawamoto, T.; Curran, D. P. Synthesis of 1,3-Dialkylimidazol-2-ylidene Boranes from 1,3-Dialkylimidazolium Iodides and Sodium Borohydride. *J. Org. Chem.* **2015**, *80* (19), 9794-9797. DOI: 10.1021/acs.joc.5b01682.

(3) Rezazadeh, S.; Devannah, V.; Watson, D. A. Nickel-Catalyzed C-Alkylation of Nitroalkanes with Unactivated Alkyl Iodides. *J. Am. Chem. Soc.* **2017**, *139* (24), 8110-8113. DOI: 10.1021/jacs.7b04312.

(4) Supranovich, V. I.; Levin, V. V.; Struchkova, M. I.; Korlyukov, A. A.; Dilman, A. D. Radical Silyldifluoromethylation of Electron-Deficient Alkenes. *Org. Lett.* **2017**, *19* (12), 3215-3218. DOI: 10.1021/acs.orglett.7b01334.

(5) (a) Cheng, Y.; Muck-Lichtenfeld, C.; Studer, A. Metal-Free Radical Borylation of Alkyl and Aryl Iodides. *Angew. Chem. Int. Ed.* **2018**, *57* (51), 16832-16836. DOI: 10.1002/anie.201810782. (b) McCauley, C. E.; Hamill, W. H.; Williams, R. R. Isomerization in the Photolysis of Alkyl Iodides. *J. Am. Chem. Soc.* **2002**, *76* (24), 6263-6266. DOI: 10.1021/ja01653a011. (c) Braslavsky, S. E.; Grotewold, J.; Lissi, E. A. Alkyl iodides as a source of alkyl radicals. Part I. Mechanism of the gas-phase photolysis of isopropyl iodide. *J. Chem. Soc. B* **1967**, 414-415. DOI: 10.1039/j29670000414.

(6) Luridiana, A.; Mazzarella, D.; Capaldo, L.; Rincon, J. A.; Garcia-Losada, P.; Mateos, C.; Frederick, M. O.; Nuno, M.; Jan Buma, W.; Noël, T. The Merger of Benzophenone HAT Photocatalysis and Silyl Radical-Induced XAT Enables Both Nickel-Catalyzed Cross-Electrophile Coupling and 1,2-Dicarbofunctionalization of Olefins. *ACS Catal.* **2022**, *12* (18), 11216-11225. DOI: 10.1021/acscatal.2c03805.

(7) Wan, T.; Capaldo, L.; Ravelli, D.; Vitullo, W.; de Zwart, F. J.; de Bruin, B.; Noël, T. Photoinduced Halogen-Atom Transfer by N-Heterocyclic Carbene-Ligated Boryl Radicals for C(sp^3)-C(sp^3) Bond Formation. *J. Am. Chem. Soc.* **2023**, *145* (2), 991-999. DOI: 10.1021/jacs.2c10444.

(8) Zhong, L.-J.; Chen, H.; Shang, X.; Xiong, B.-Q.; Tang, K.-W.; Liu, Y. Oxidant-Assisted Sulfonylation/Cyclization Cascade Synthesis of Alkylsulfonylated Oxindoles via the Insertion of SO2. *J. Org. Chem.* **2024**, *89* (8), 5409-5422. DOI: 10.1021/acs.joc.3c02860.

(9) Gaussian 16, Revision C.01, M. J. Frisch, G. W. Trucks, H. B. Schlegel, G. E. Scuseria, M. A. Robb, J. R. Cheeseman, G. Scalmani, V. Barone, G. A. Petersson, H. Nakatsuji, X. Li, M. Caricato, A. V. Marenich, J. Bloino, B. G. Janesko, R. Gomperts, B. Mennucci, H. P. Hratchian, J. V. Ortiz, A. F. Izmaylov, J. L. Sonnenberg, D. Williams-Young, F. Ding, F. Lipparini, F. Egidi, J. Goings, B. Peng, A. Petrone, T. Henderson, D. Ranasinghe, V. G. Zakrzewski, J. Gao, N. Rega, G. Zheng, W. Liang, M. Hada, M. Ehara, K. Toyota, R. Fukuda, J. Hasegawa, M. Ishida, T. Nakajima, Y. Honda, O. Kitao, H. Nakai, T. Vreven, K. Throssell, J. A. Montgomery, Jr., J. E. Peralta, F. Ogliaro, M. J. Bearpark, J. J. Heyd, E. N. Brothers, K. N. Kudin, V. N. Staroverov, T. A. Keith, R. Kobayashi, J. Normand, K. Raghavachari, A. P. Rendell, J. C. Burant, S. S. Iyengar, J. Tomasi, M. Cossi, J. M. Millam, M. Klene, C. Adamo, R. Cammi, J. W. Ochterski, R. L. Martin, K. Morokuma, O. Farkas, J. B. Foresman, and D. J. Fox, Gaussian, Inc., Wallingford CT, 2016.

(10) Rohe, S.; Morris, A. O.; McCallum, T.; Barriault, L. Hydrogen Atom Transfer Reactions via Photoredox Catalyzed Chlorine Atom Generation. *Angew. Chem. Int. Ed.* **2018**, *57* (48), 15664-15669. DOI: 10.1002/anie.201810187.

(11) Borlinghaus, N.; Schonfeld, B.; Heitz, S.; Klee, J.; Vukelic, S.; Braje, W. M.; Jolit, A. Enabling Metallophotoredox Catalysis in Parallel Solution-Phase Synthesis Using Disintegrating Reagent Tablets. *J. Org. Chem.* **2021**, *86* (23), 16535-16547. DOI: 10.1021/acs.joc.1c01867.
(12) Sumino, S.; Ryu, I. Hydroalkylation of Alkenes Using Alkyl Iodides and Hantzsch Ester under Palladium/Light System. *Org. Lett.* **2016**, *18* (1), 52-55. DOI: 10.1021/acs.orglett.5b03238.
(13) Jin, Y.; Zhang, Q.; Wang, L.; Wang, X.; Meng, C.; Duan, C. Convenient C(sp3)–H bond functionalisation of light alkanes and other compounds by iron photocatalysis. *Green Chem.* **2021**, *23* (18), 6984-6989. DOI: 10.1039/d1gc01563j.
(14) Geant, P.-Y.; Mohamed, B. S.; Périgaud, C.; Peyrottes, S.; Uttaro, J.-P.; Mathé, C. Probing the reactivity of H-phosphonate derivatives for the hydrophosphonylation of various alkenes and alkynes under free-radical conditions. *New J. Chem.* **2016**, *40* (6), 5318-5324. DOI: 10.1039/c6nj00123h.
(15) Xue, F.; Wang, F.; Liu, J.; Di, J.; Liao, Q.; Lu, H.; Zhu, M.; He, L.; He, H.; Zhang, D.; et al. A Desulfurative Strategy for the Generation of Alkyl Radicals Enabled by Visible-Light Photoredox Catalysis. *Angew. Chem. Int. Ed.* **2018**, *57* (22), 6667-6671. DOI: 10.1002/anie.201802710.
(16) Zhou, Z.; Kweon, J.; Jung, H.; Kim, D.; Seo, S.; Chang, S. Photoinduced Transition-Metal-Free Chan-Evans-Lam-Type Coupling: Dual Photoexcitation Mode with Halide Anion Effect. *J. Am. Chem. Soc.* **2022**, *144* (20), 9161-9171. DOI: 10.1021/jacs.2c03343.
(17) Truesdell, B. L.; Hamby, T. B.; Sevov, C. S. General C(sp(2))-C(sp(3)) Cross-Electrophile Coupling Reactions Enabled by Overcharge Protection of Homogeneous Electrocatalysts. *J. Am. Chem. Soc.* **2020**, *142* (12), 5884-5893. DOI: 10.1021/jacs.0c01475.
(18) Manley, D. W.; McBurney, R. T.; Miller, P.; Walton, J. C.; Mills, A.; O'Rourke, C. Titania-promoted carboxylic acid alkylations of alkenes and cascade addition-cyclizations. *J. Org. Chem.* **2014**, *79* (3), 1386-1398. DOI: 10.1021/jo4027929.
(19) Doohan, R. A.; Hannan, J. J.; Geraghty, N. W. The photomediated reaction of alkynes with cycloalkanes. *Org. Biomol. Chem.* **2006**, *4* (5), 942-952. DOI: 10.1039/b517631j.

12. NMR spectra

12.1 NMR spectra of starting materials

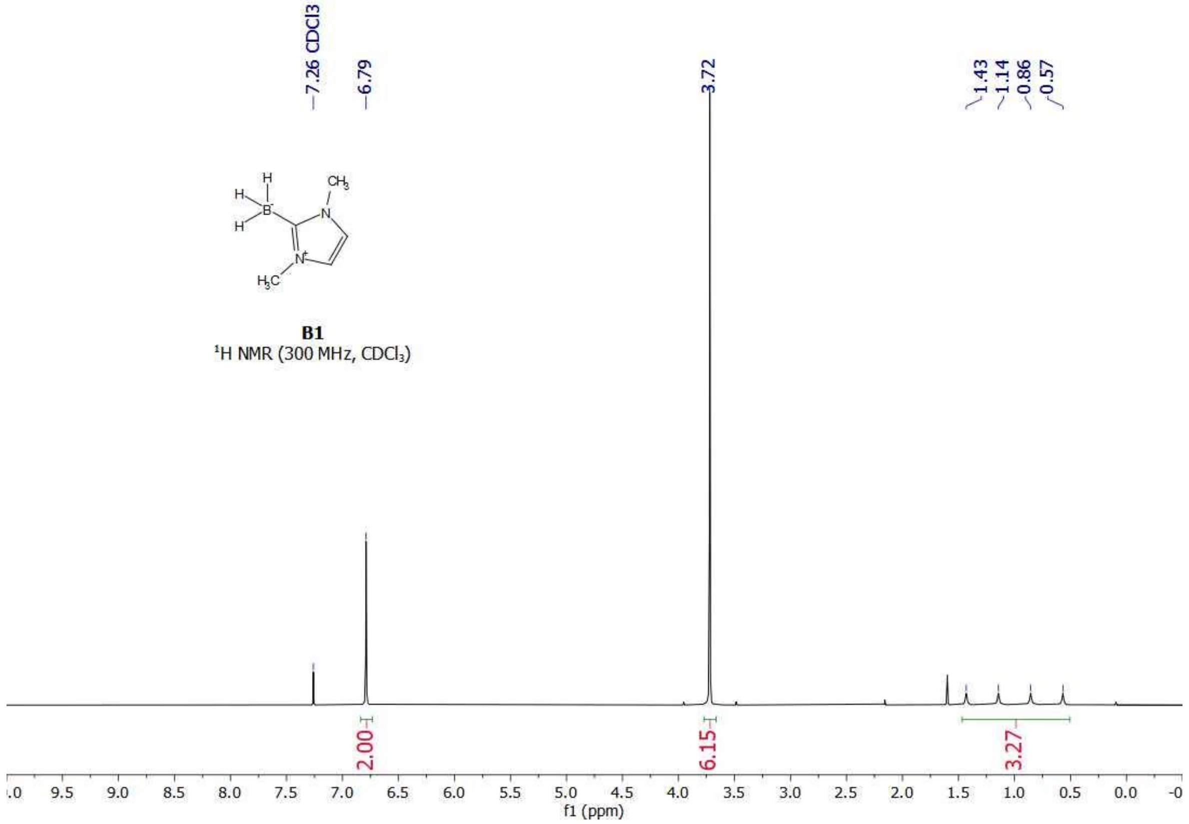

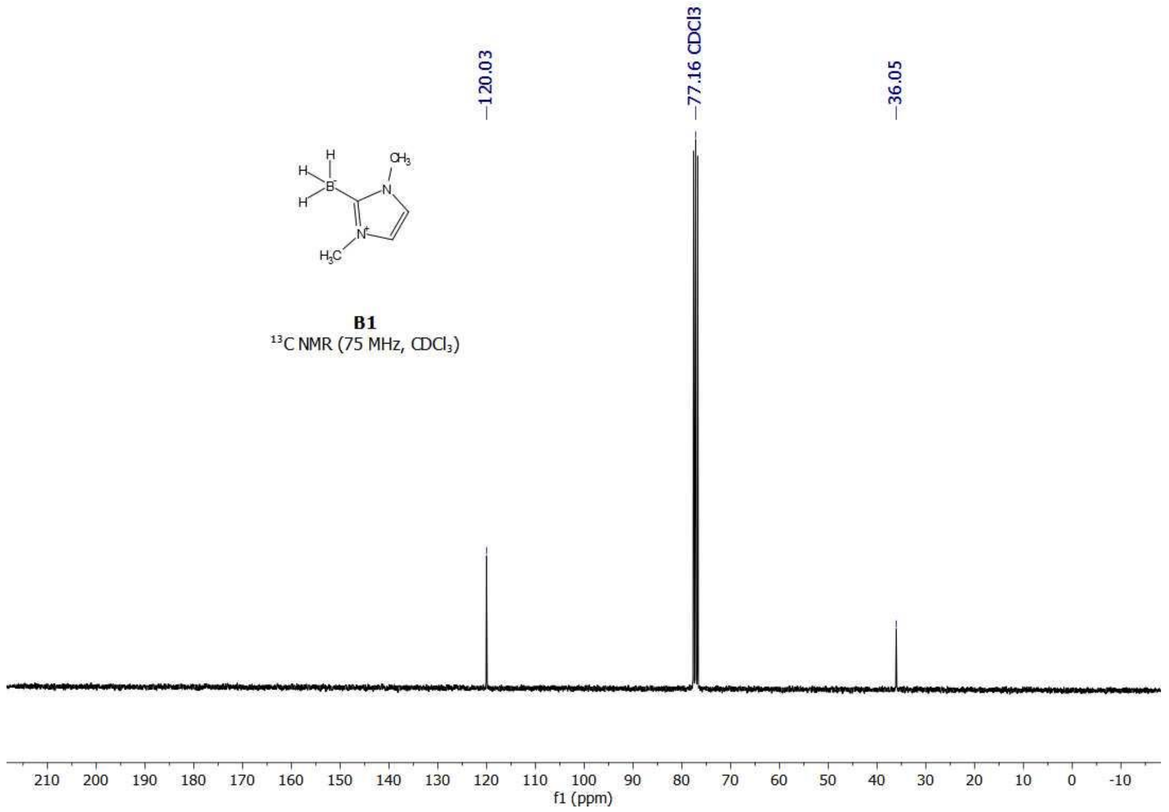

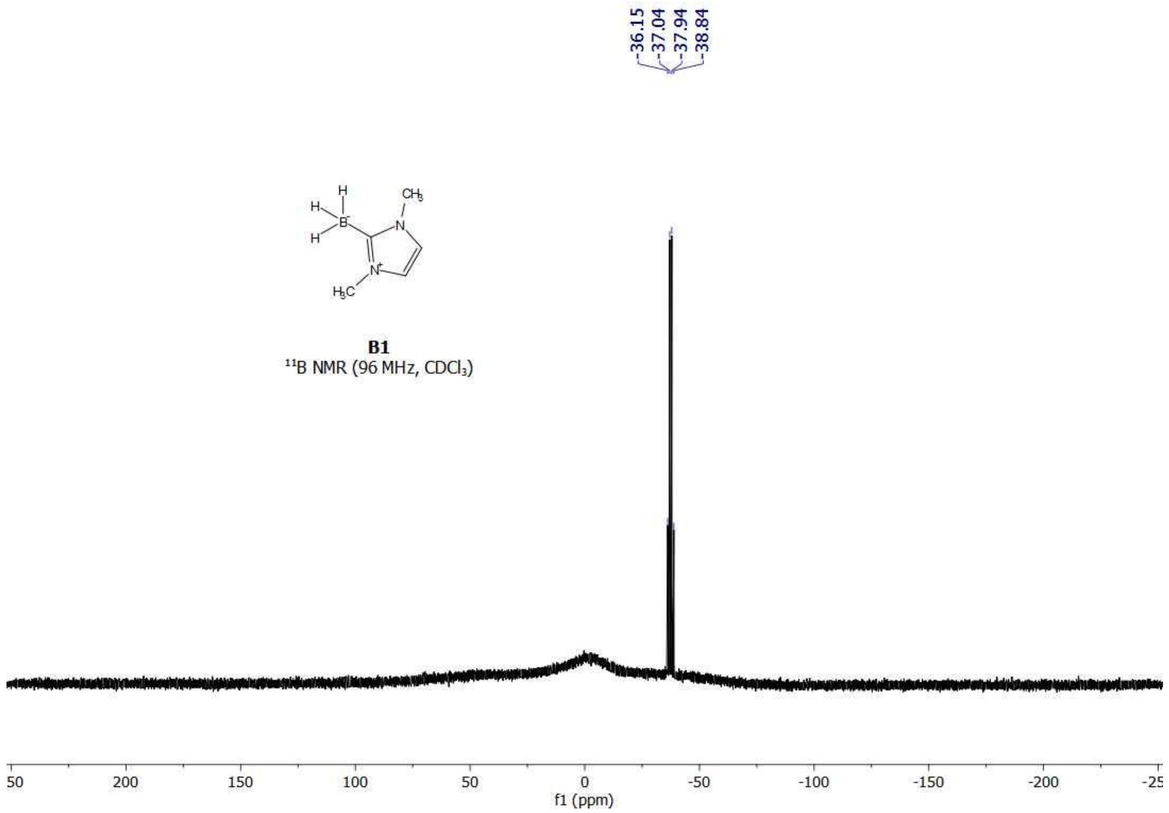
-36.15
-37.04
-37.94
-38.84
B1
^{11}B NMR (96 MHz, CDCl$_3$)
50
200
150
100
50
0
-50
-100
-150
-200
-25
f1 (ppm)

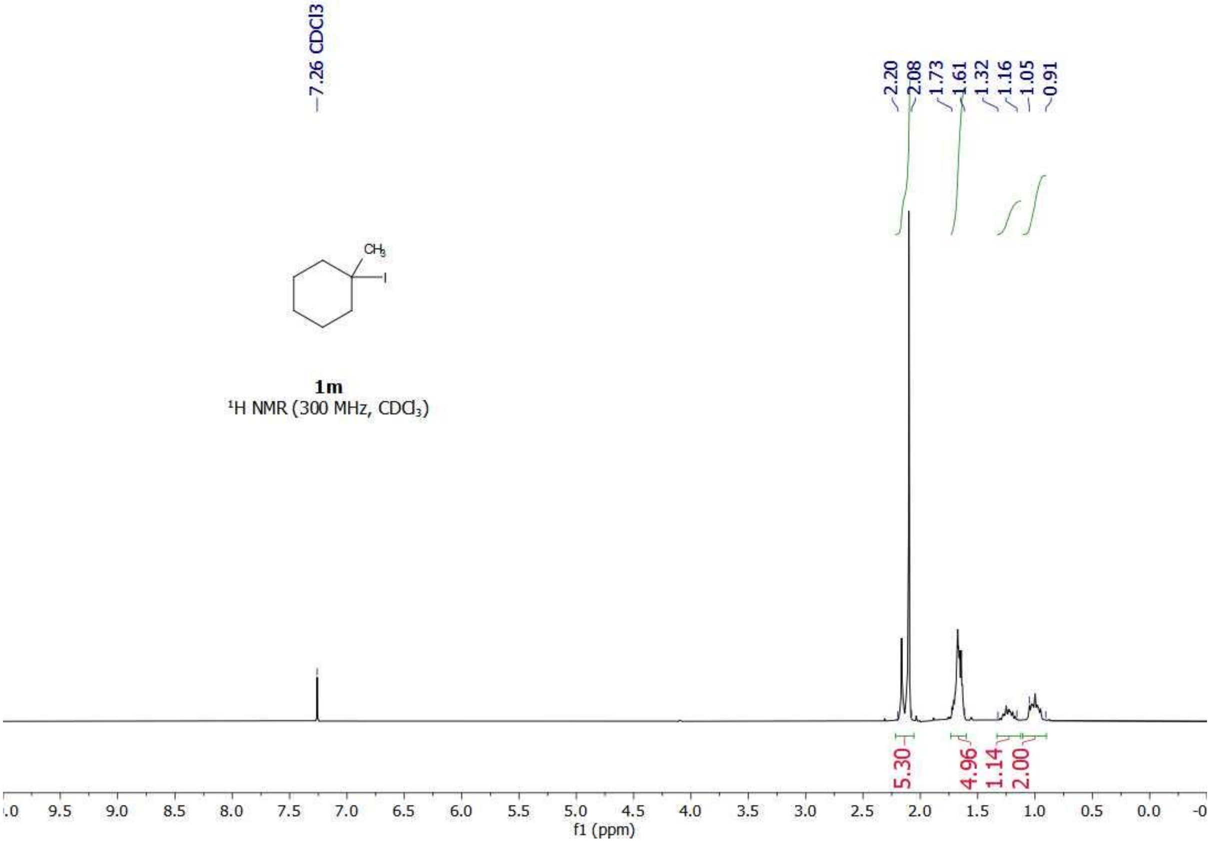
7.26 CDCl3
2.20
2.08
1.73
1.61
1.32
1.16
1.05
0.91
CH3
I
1m
1H NMR (300 MHz, CDCl3)
5.30
4.96
1.14
2.00
f1 (ppm)

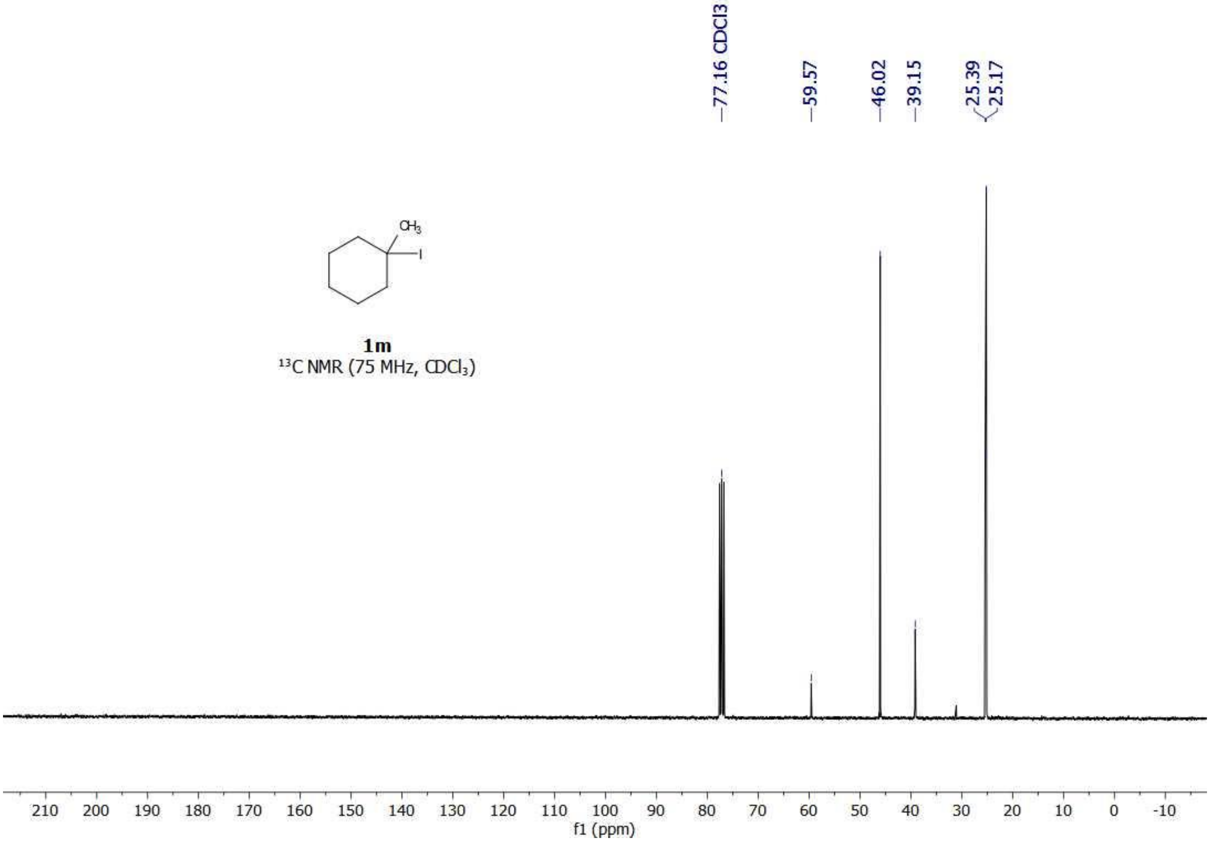
77.16 CDCl3
59.57
46.02
39.15
25.39
25.17
CH3
I
1m
13C NMR (75 MHz, CDCl3)
f1 (ppm)

12.2 NMR spectra of products

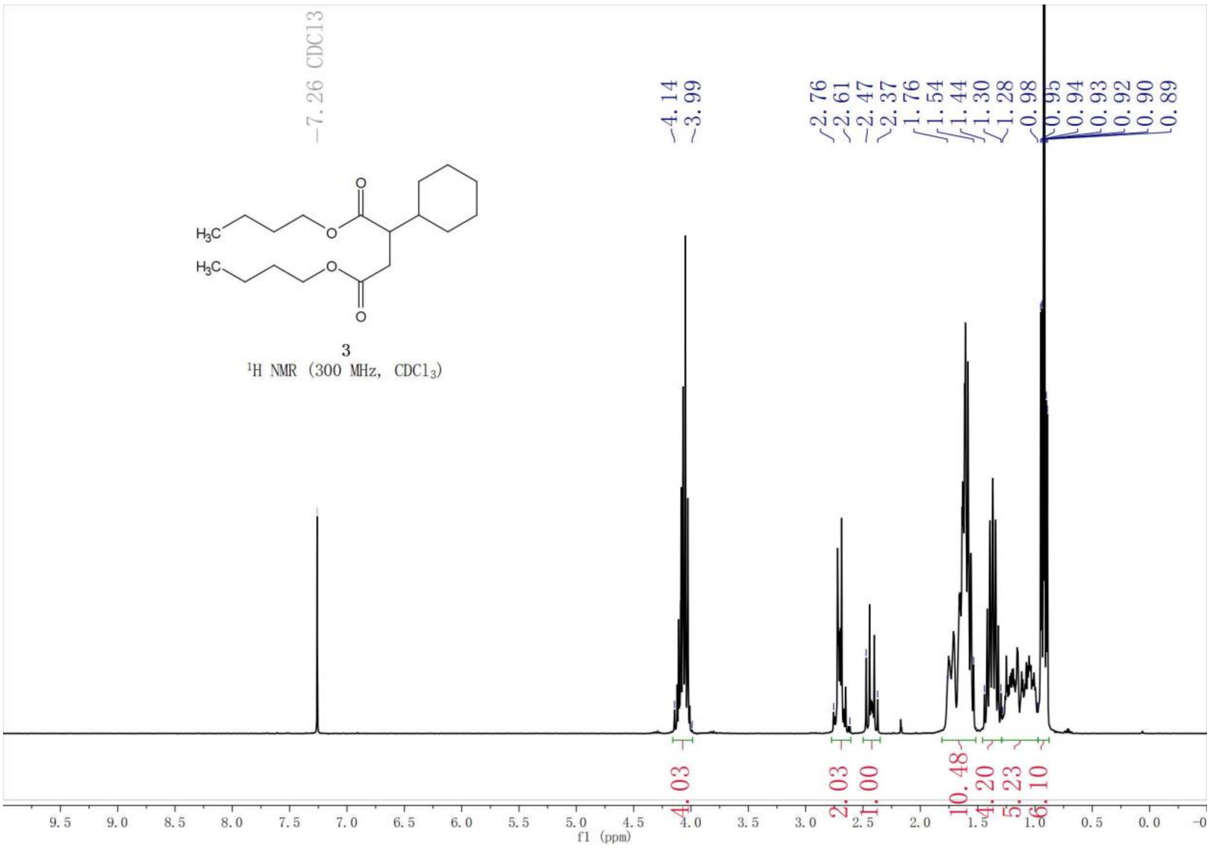

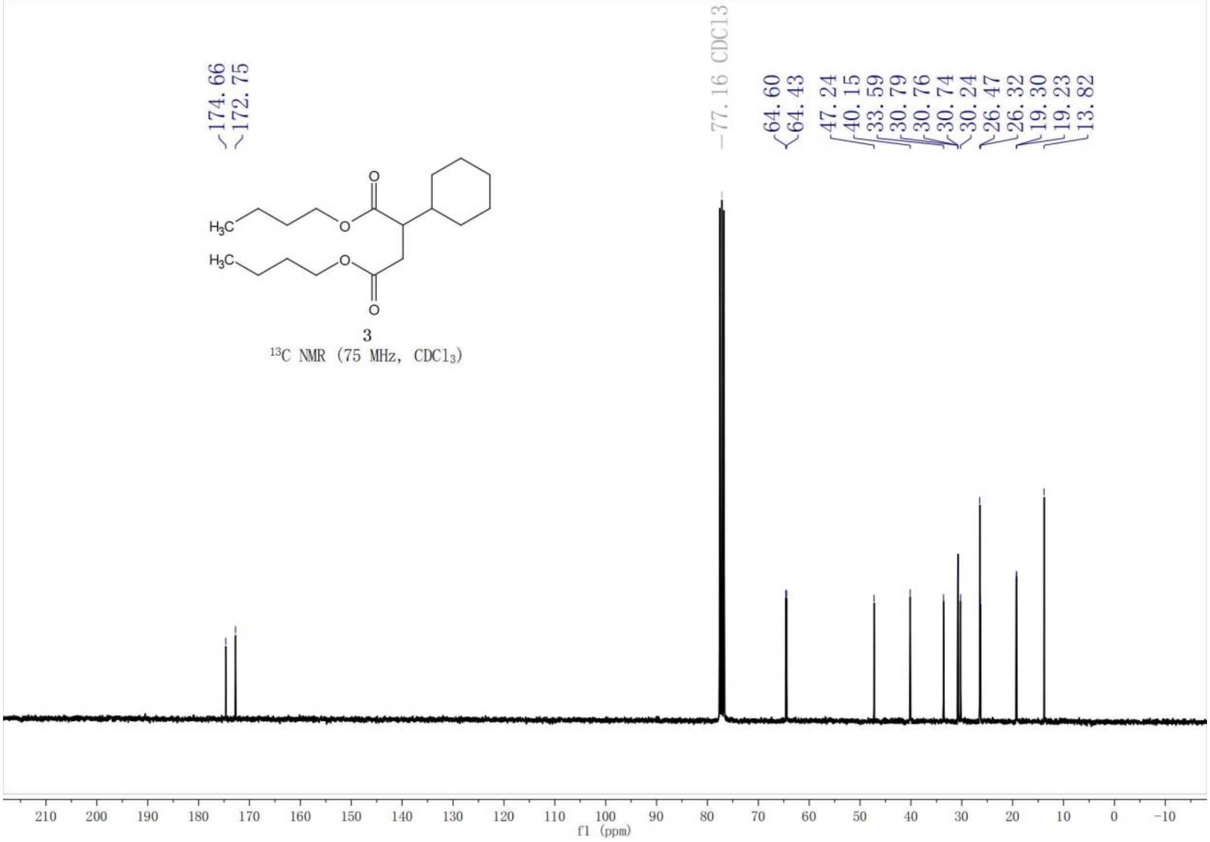

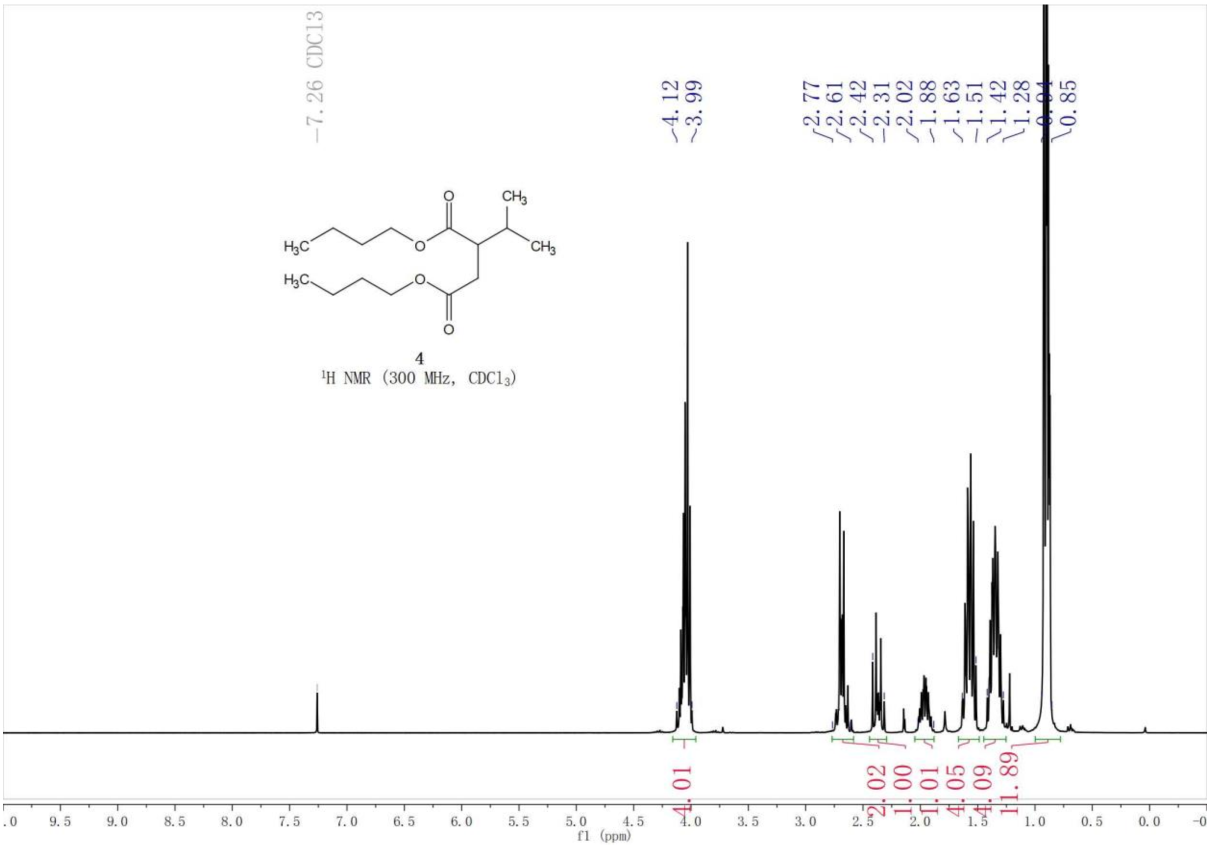
7.26 CDCl3
4.12
3.99
2.77
2.61
2.42
2.31
2.02
1.88
1.63
1.51
1.42
1.28
0.85
4
¹H NMR (300 MHz, CDCl3)
4.01
2.02
1.00
1.01
4.05
4.09
11.89
f1 (ppm)

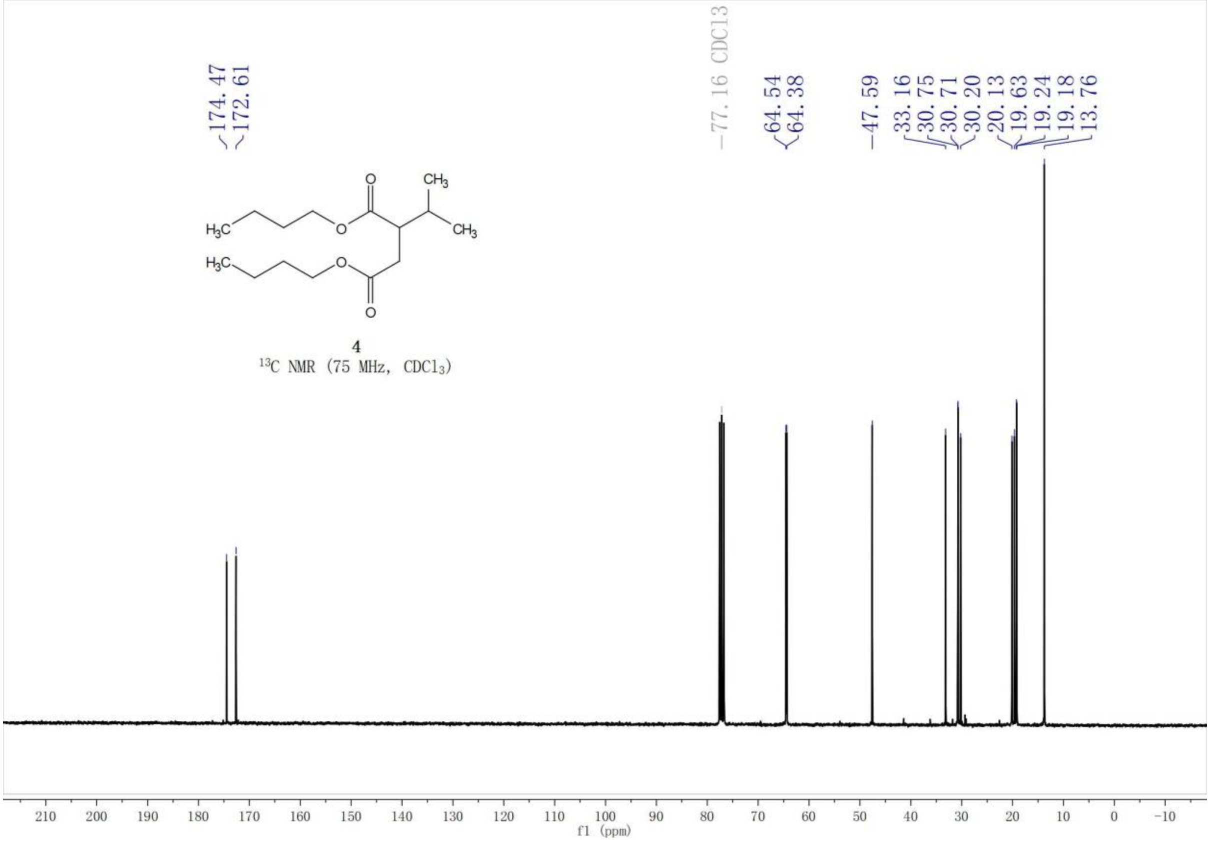
174.47
172.61
77.16 CDCl3
64.54
64.38
47.59
33.16
30.75
30.71
30.20
20.13
19.63
19.24
19.18
13.76
4
¹³C NMR (75 MHz, CDCl3)
f1 (ppm)

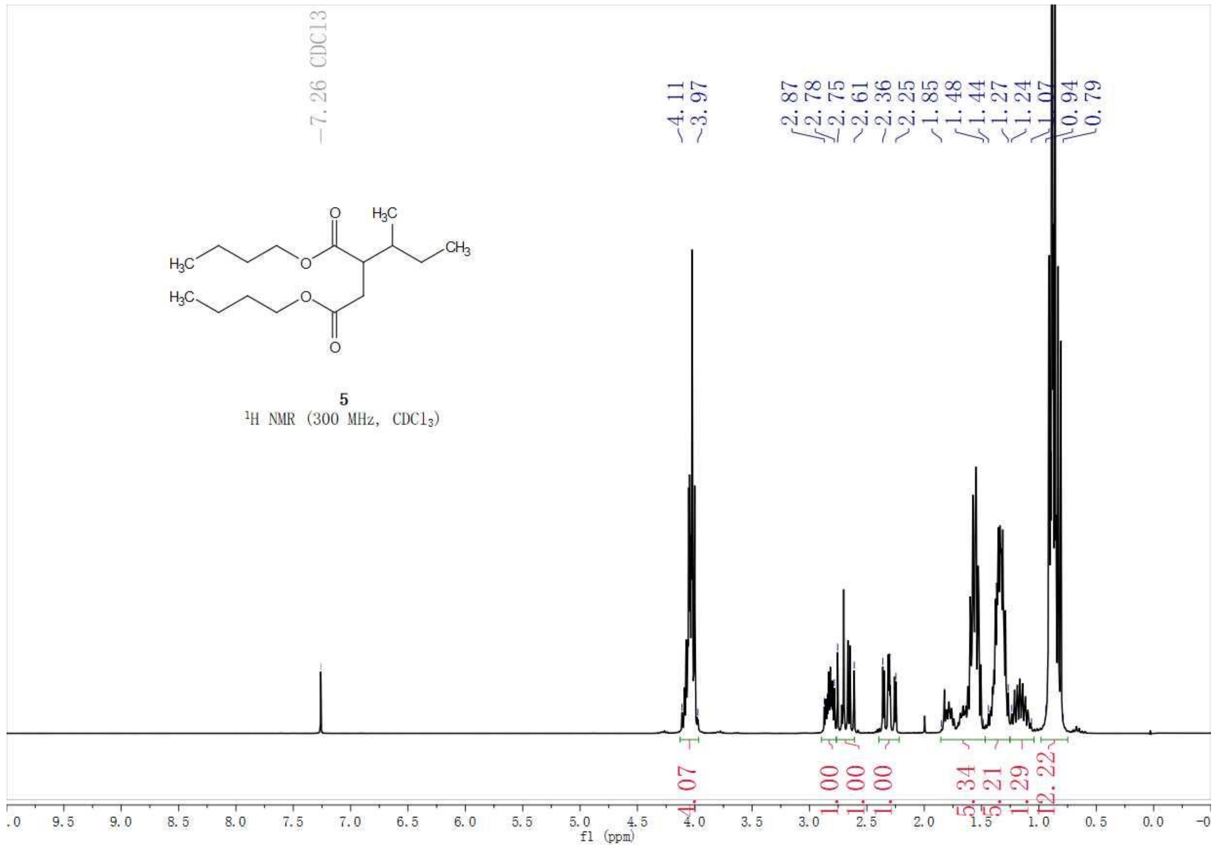
7.26 CDCl3
4.11
3.97
2.87
2.78
2.75
2.61
2.36
2.25
1.85
1.48
1.44
1.27
1.24
0.94
0.79
H3C
O
CH3
5
1H NMR (300 MHz, CDCl3)
4.07
1.00
1.00
1.00
5.34
5.21
1.29
12.22
9.5 9.0 8.5 8.0 7.5 7.0 6.5 6.0 5.5 5.0 4.5 4.0 3.5 3.0 2.5 2.0 1.5 1.0 0.5 0.0
f1 (ppm)

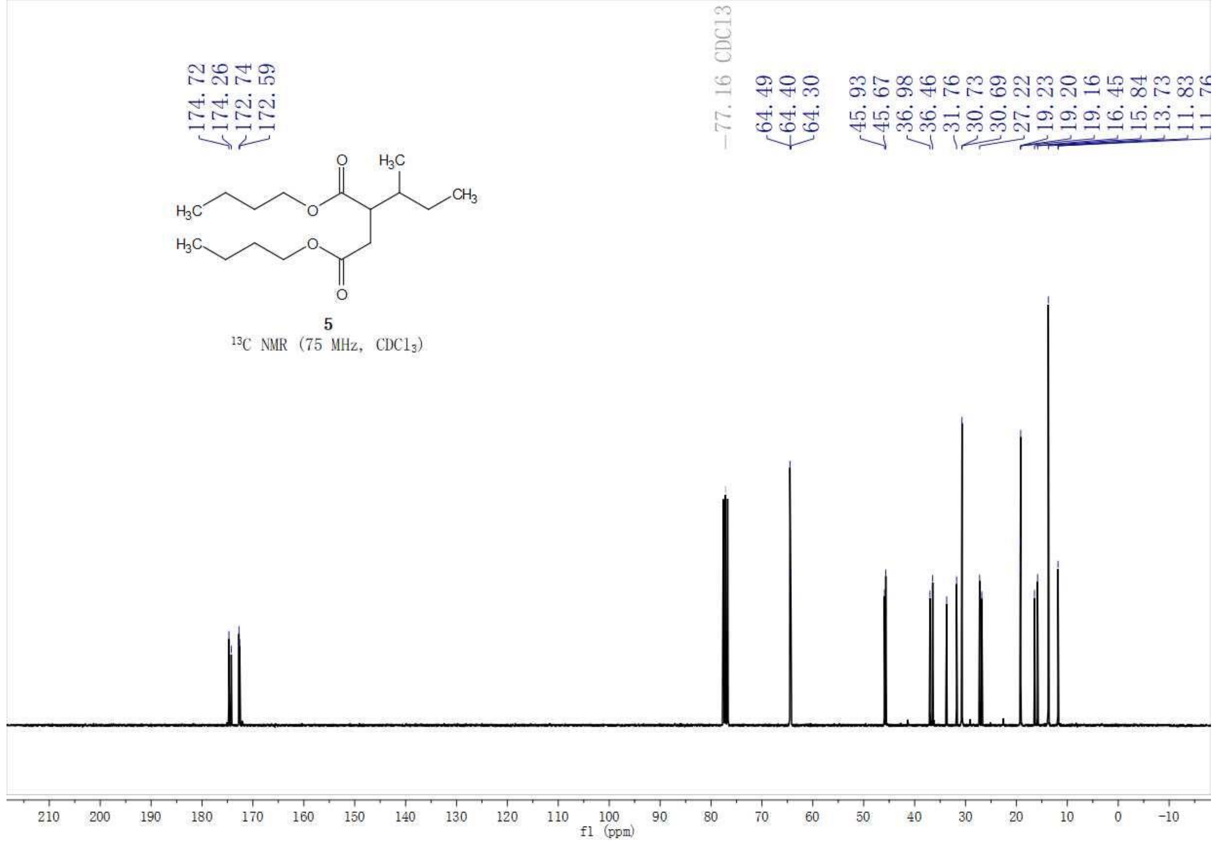
174.72
174.26
172.74
172.59
77.16 CDCl3
64.49
64.40
64.30
45.93
45.67
36.98
36.46
31.76
30.73
30.69
27.22
19.23
19.20
19.16
16.45
15.84
13.73
11.83
11.76
H3C
O
CH3
5
13C NMR (75 MHz, CDCl3)
210 200 190 180 170 160 150 140 130 120 110 100 90 80 70 60 50 40 30 20 10 0 -10
f1 (ppm)

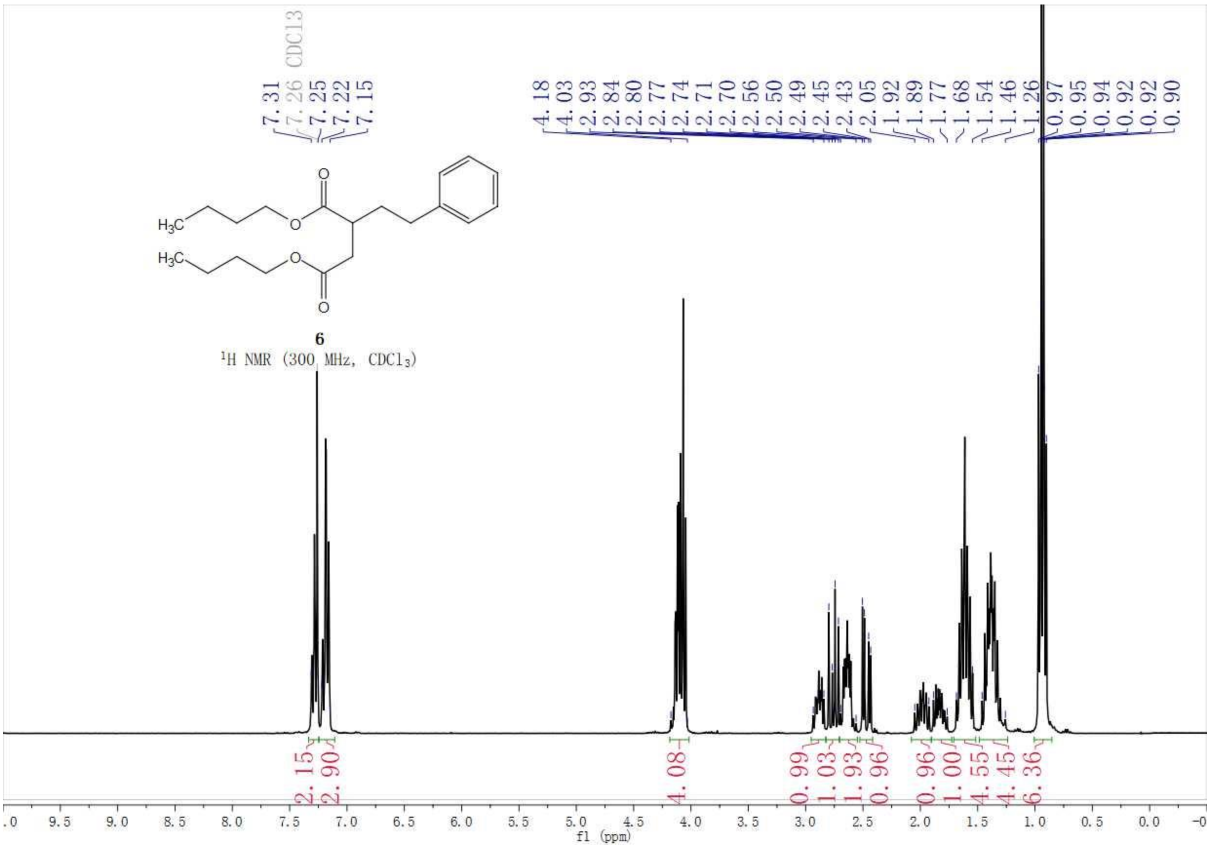
7.31
7.26 CDCl3
7.25
7.22
7.15
4.18
4.03
2.93
2.84
2.80
2.77
2.74
2.71
2.70
2.56
2.50
2.49
2.45
2.43
2.05
1.92
1.89
1.77
1.68
1.54
1.46
1.26
0.97
0.95
0.94
0.92
0.92
0.90
H3C
O
O
H3C
O
O
6
1H NMR (300 MHz, CDCl3)
2.15
2.90
4.08
0.99
1.03
1.93
0.96
0.96
1.00
4.55
4.45
6.36
f1 (ppm)

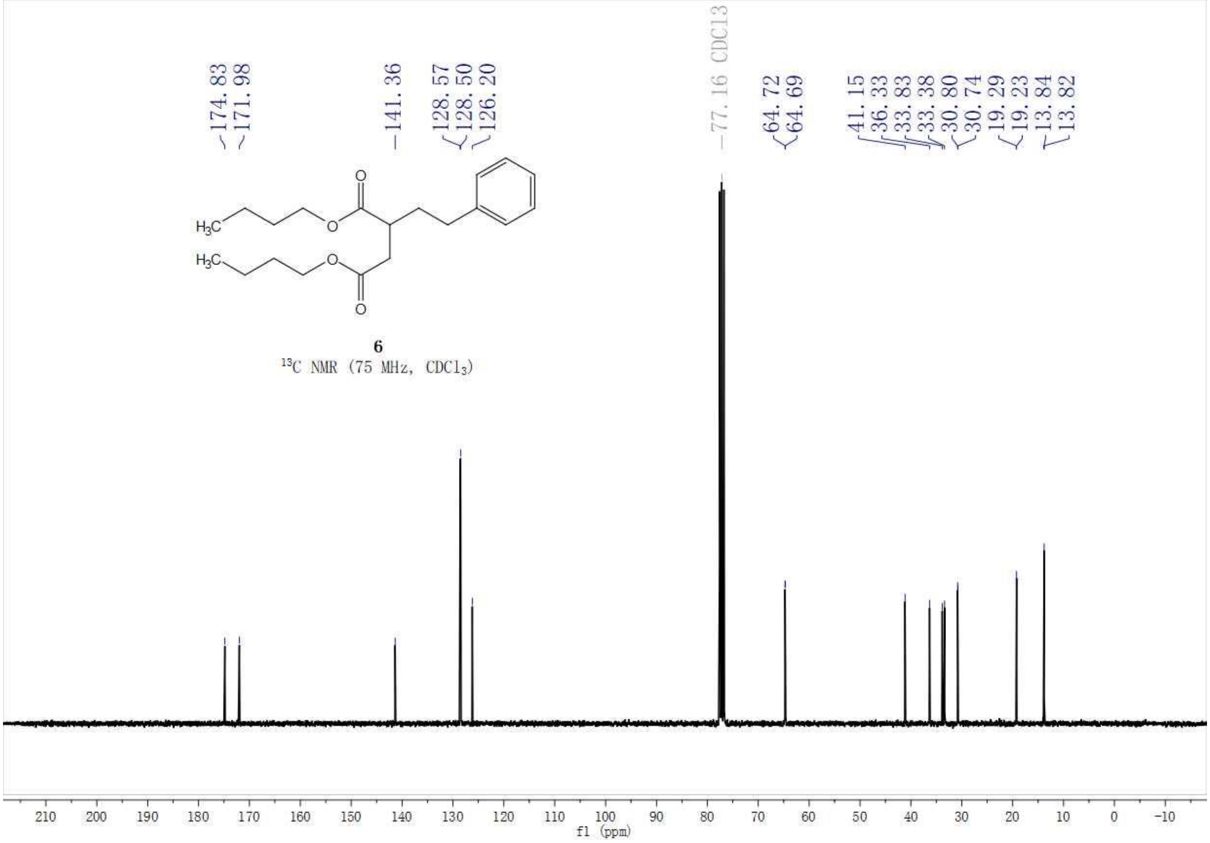
174.83
171.98
141.36
128.57
128.50
126.20
77.16 CDCl3
64.72
64.69
41.15
36.33
33.83
33.38
30.80
30.74
19.29
19.23
13.84
13.82
H3C
O
O
H3C
O
O
6
13C NMR (75 MHz, CDCl3)
f1 (ppm)

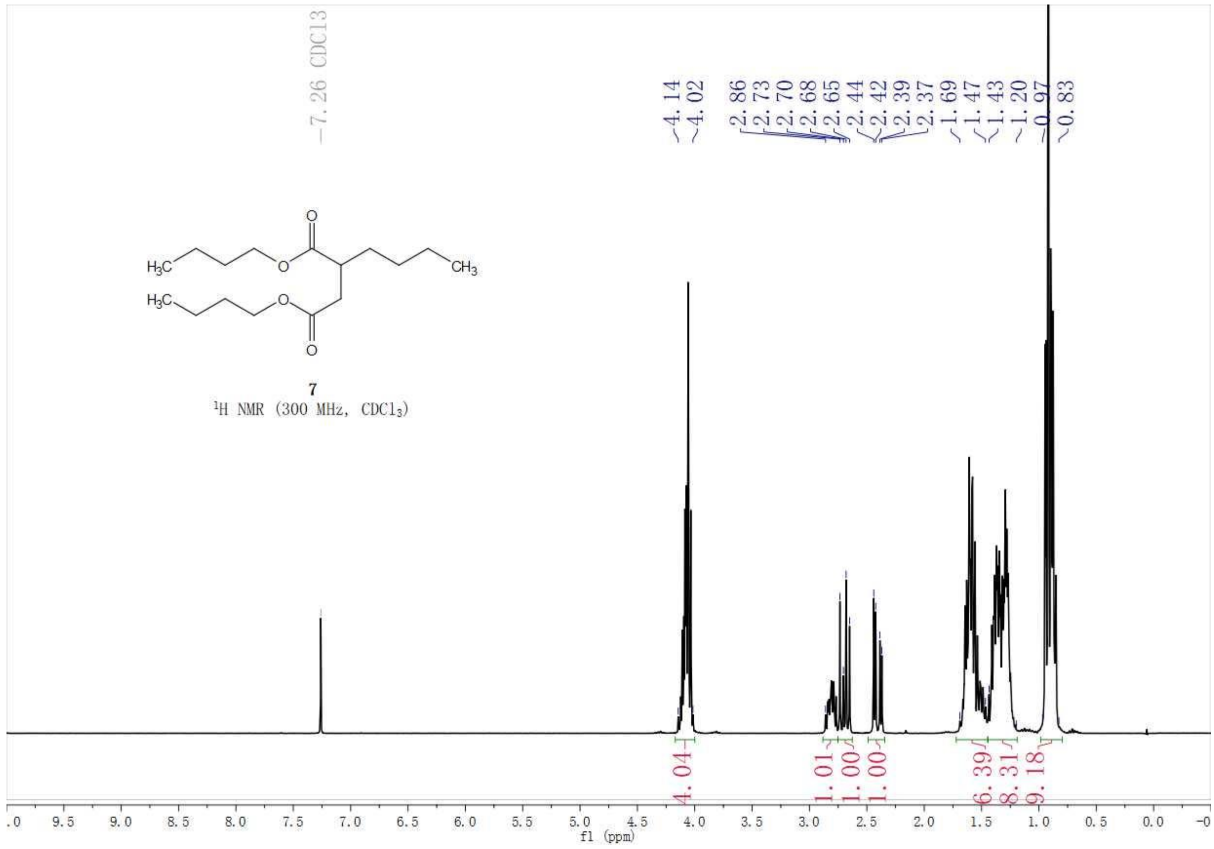

7
^{1}H NMR (300 MHz, $CDCl_3$)

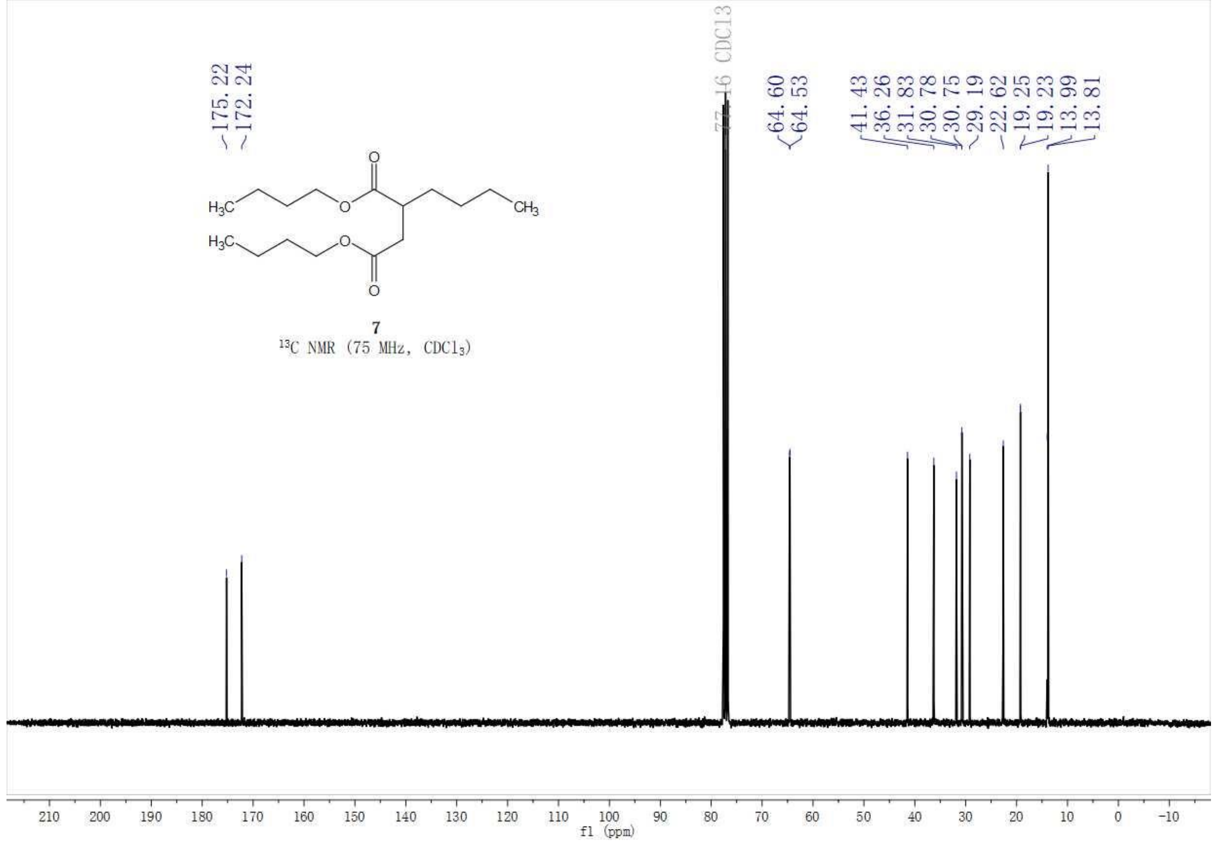

7
^{13}C NMR (75 MHz, $CDCl_3$)

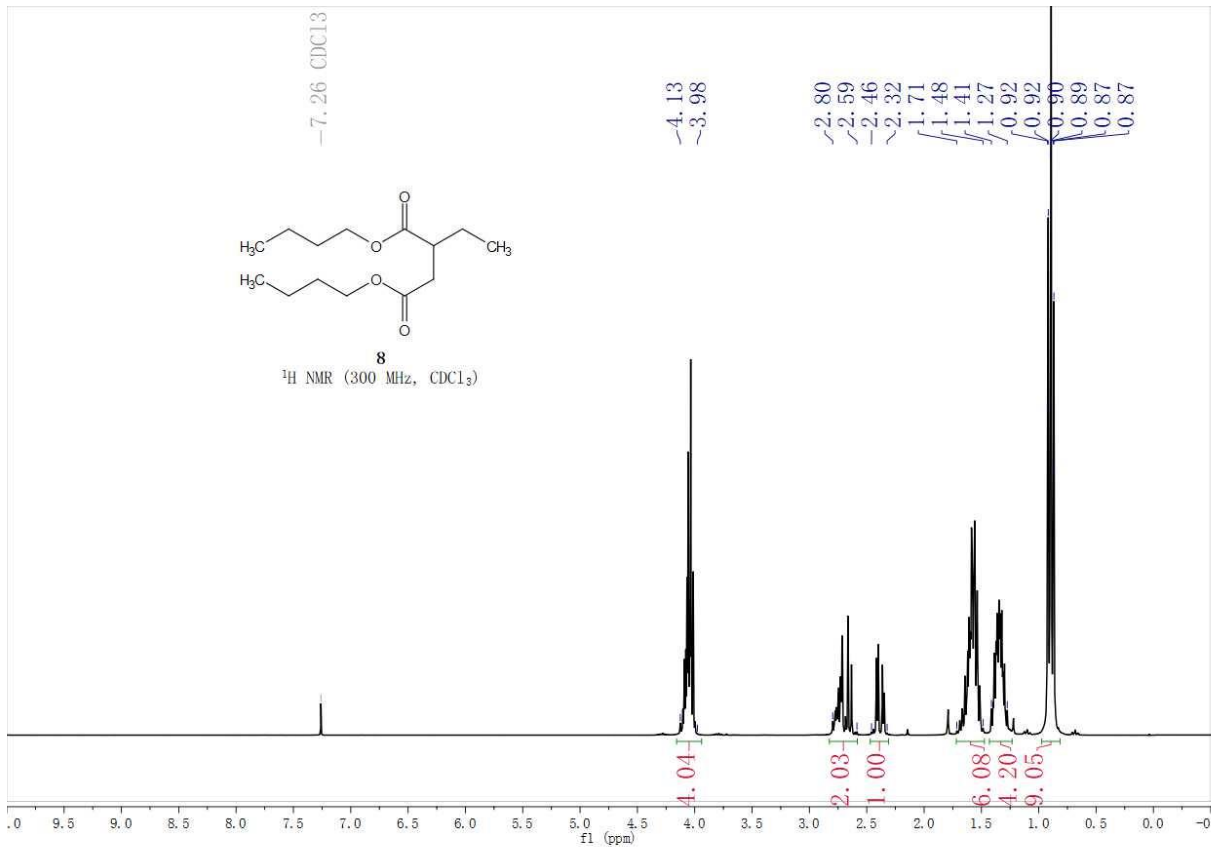
7.26 CDCl3
4.13
3.98
2.80
2.59
2.46
2.32
1.71
1.48
1.41
1.27
0.92
0.92
0.90
0.89
0.87
0.87
8
1H NMR (300 MHz, CDCl3)
4.04
2.03
1.00
6.08
4.20
9.05
f1 (ppm)

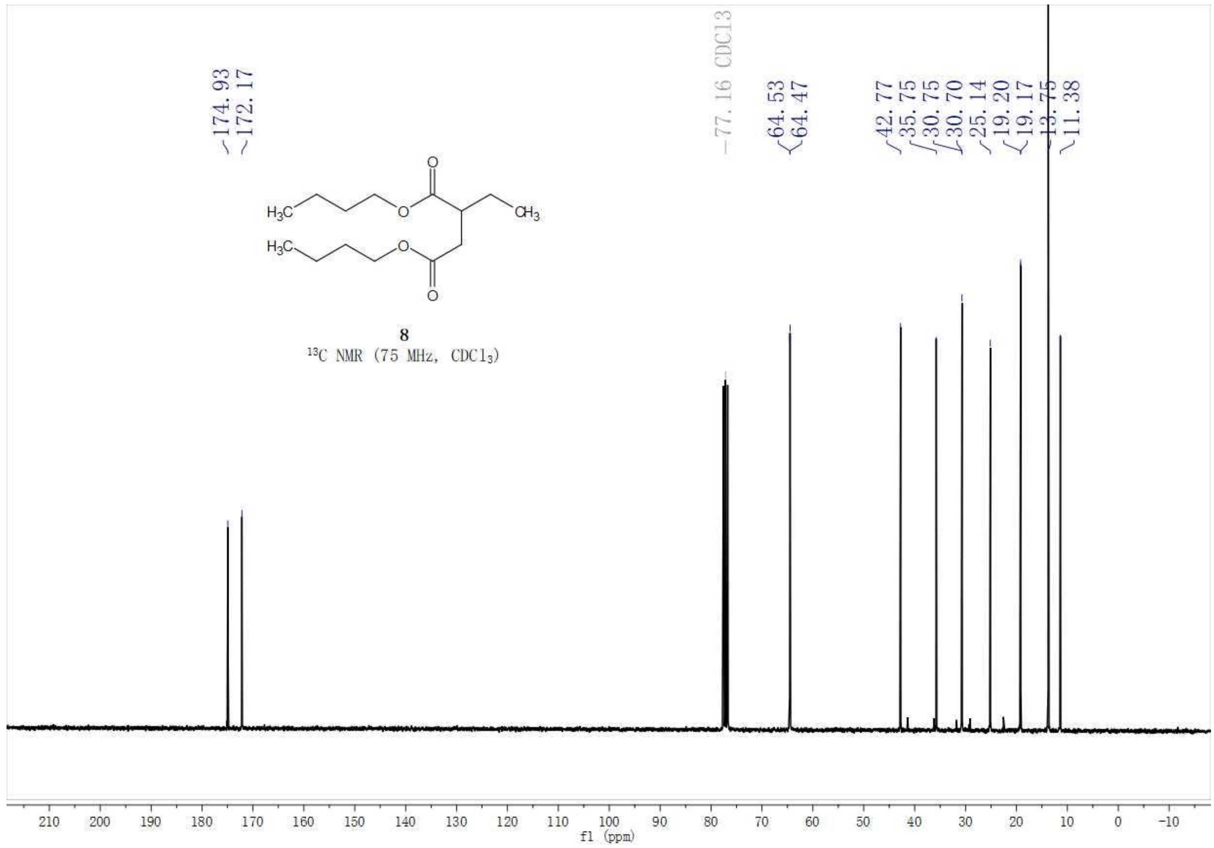
174.93
172.17
77.16 CDCl3
64.53
64.47
42.77
35.75
30.75
30.70
25.14
19.20
19.17
13.75
11.38
8
13C NMR (75 MHz, CDCl3)
f1 (ppm)

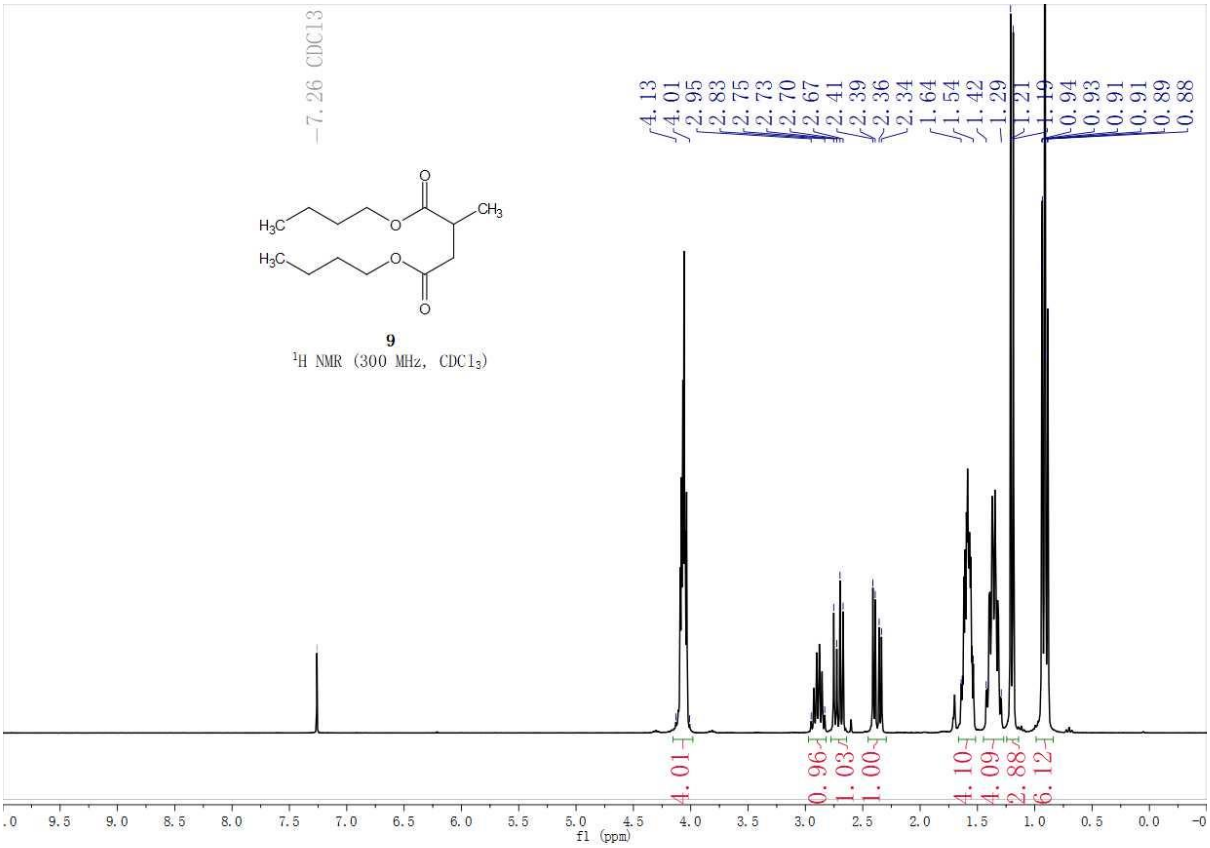

9

^{1}H NMR (300 MHz, $CDCl_3$)

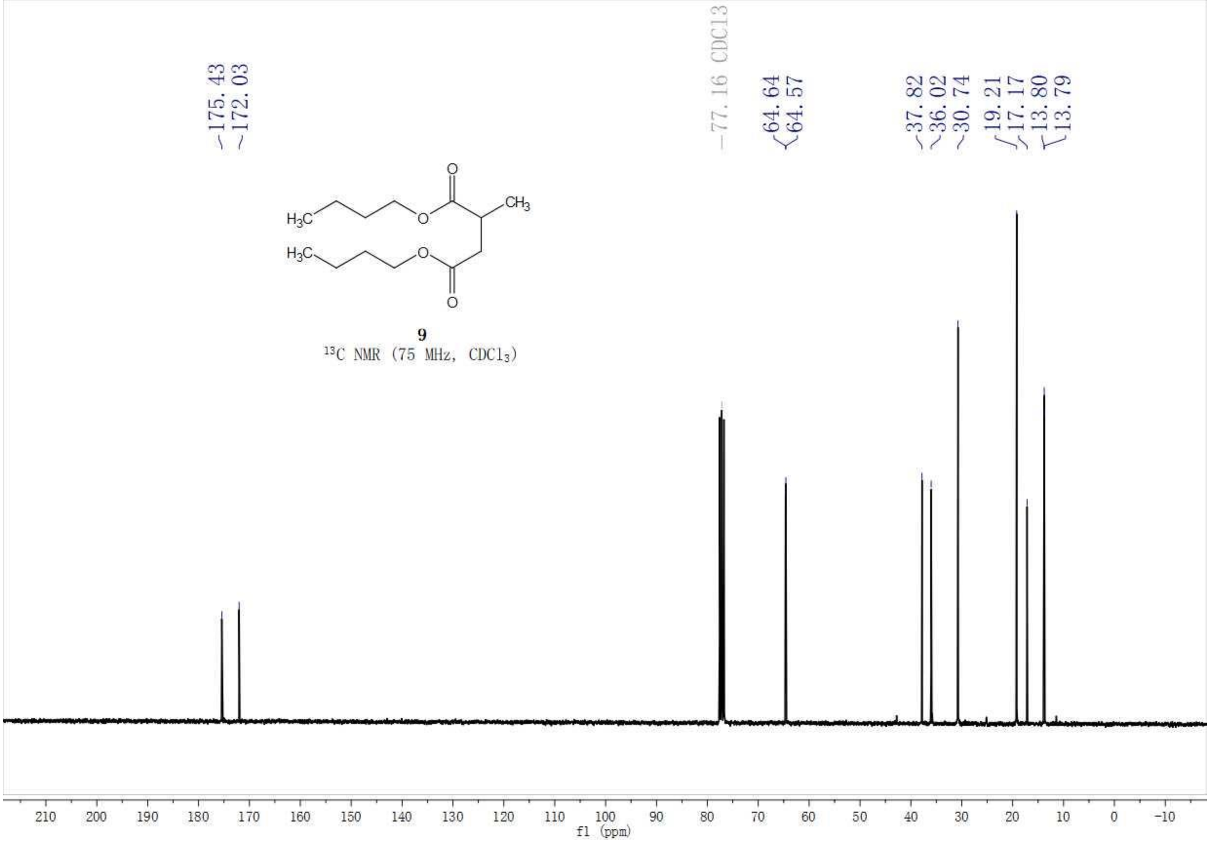

9

^{13}C NMR (75 MHz, $CDCl_3$)

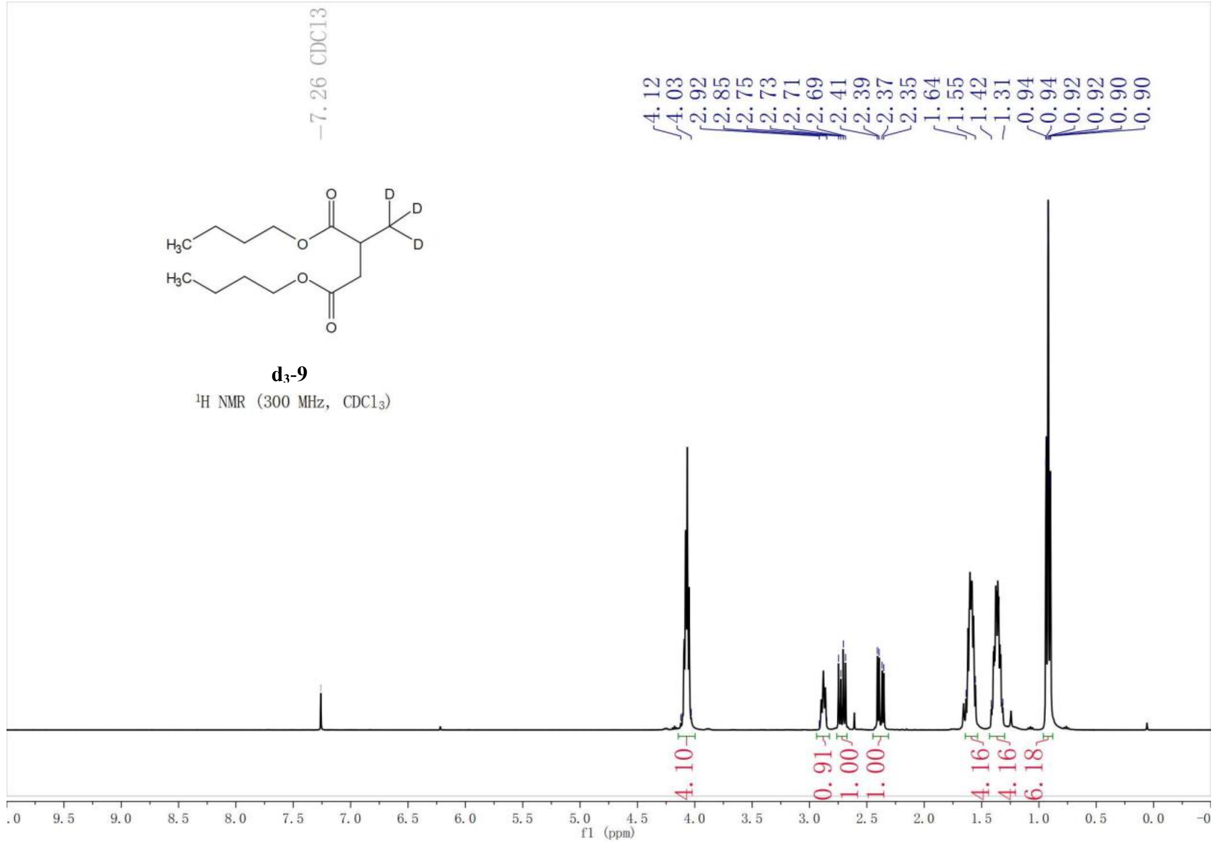

d$_3$-9

^{1}H NMR (300 MHz, $CDCl_3$)

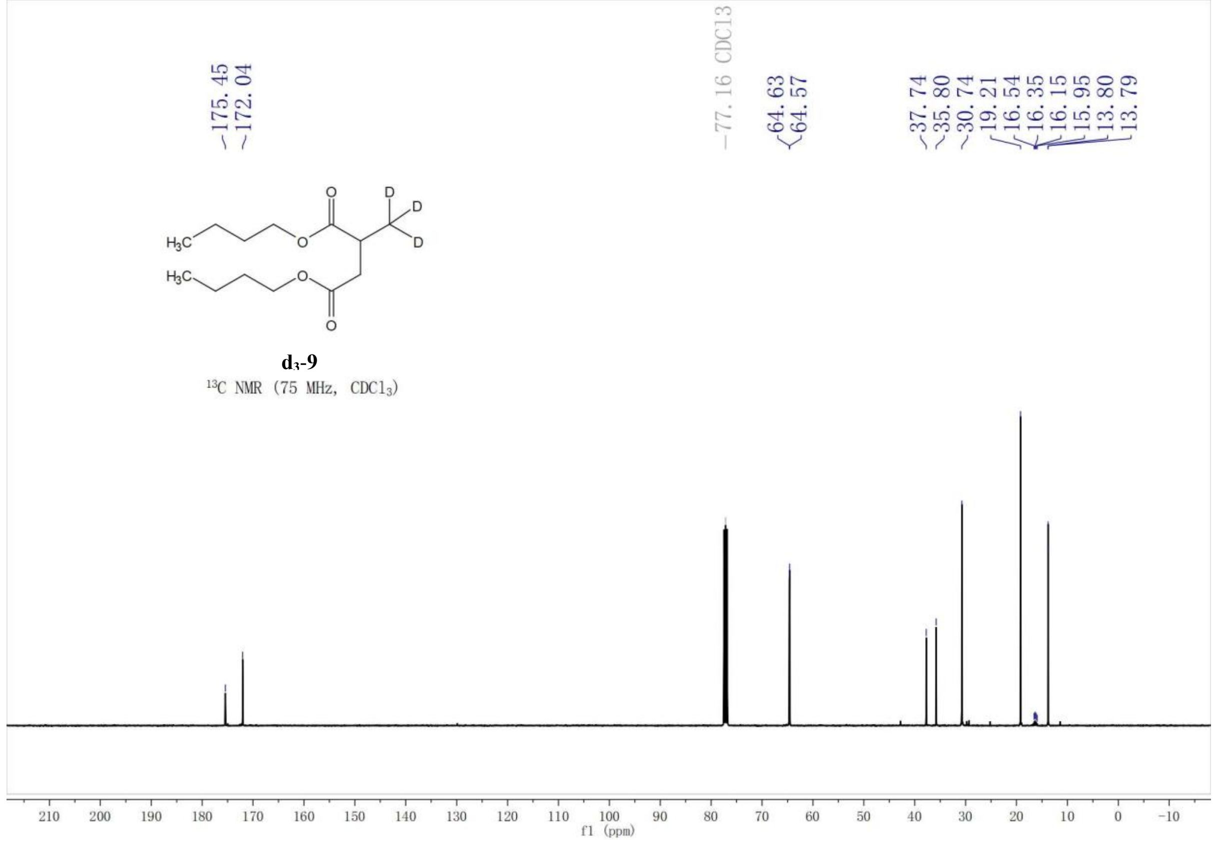

d$_3$-9

^{13}C NMR (75 MHz, $CDCl_3$)

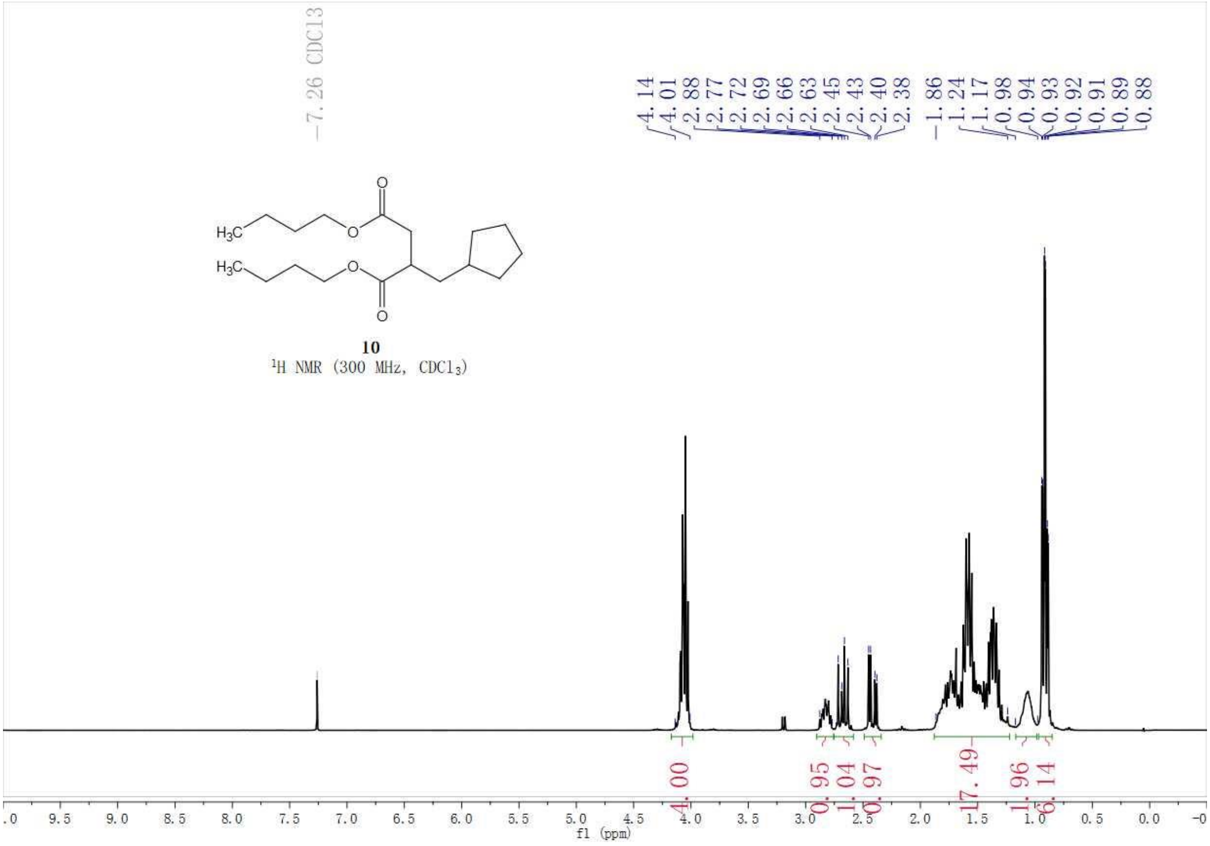

7.26 CDCl3
4.14
4.01
2.88
2.77
2.72
2.69
2.66
2.63
2.45
2.43
2.40
2.38
1.86
1.24
1.17
0.98
0.94
0.93
0.92
0.91
0.89
0.88
H3C
O
10
1H NMR (300 MHz, CDCl3)
4.00
0.95
1.04
0.97
17.49
1.96
6.14
f1 (ppm)

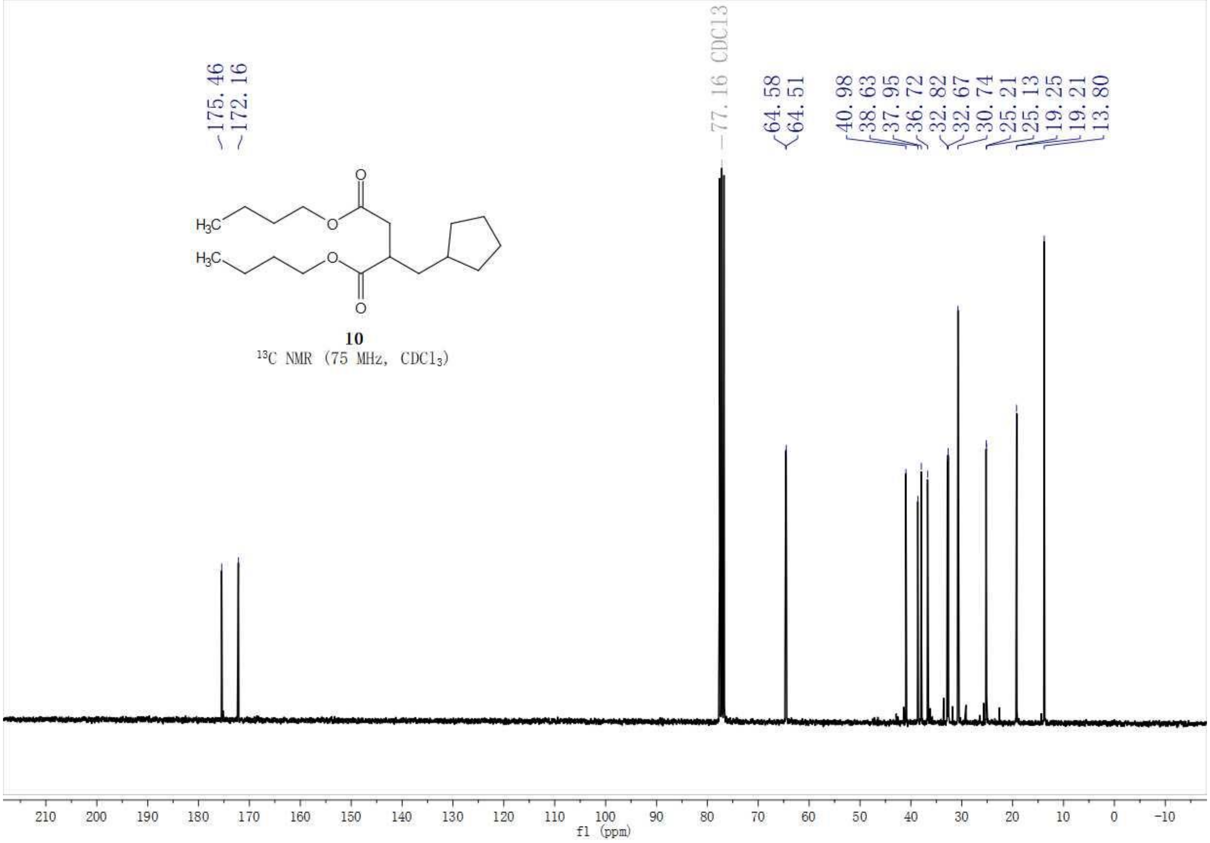

175.46
172.16
77.16 CDCl3
64.58
64.51
40.98
38.63
37.95
36.72
32.82
32.67
30.74
25.21
25.13
19.25
19.21
13.80
H3C
O
10
13C NMR (75 MHz, CDCl3)
f1 (ppm)

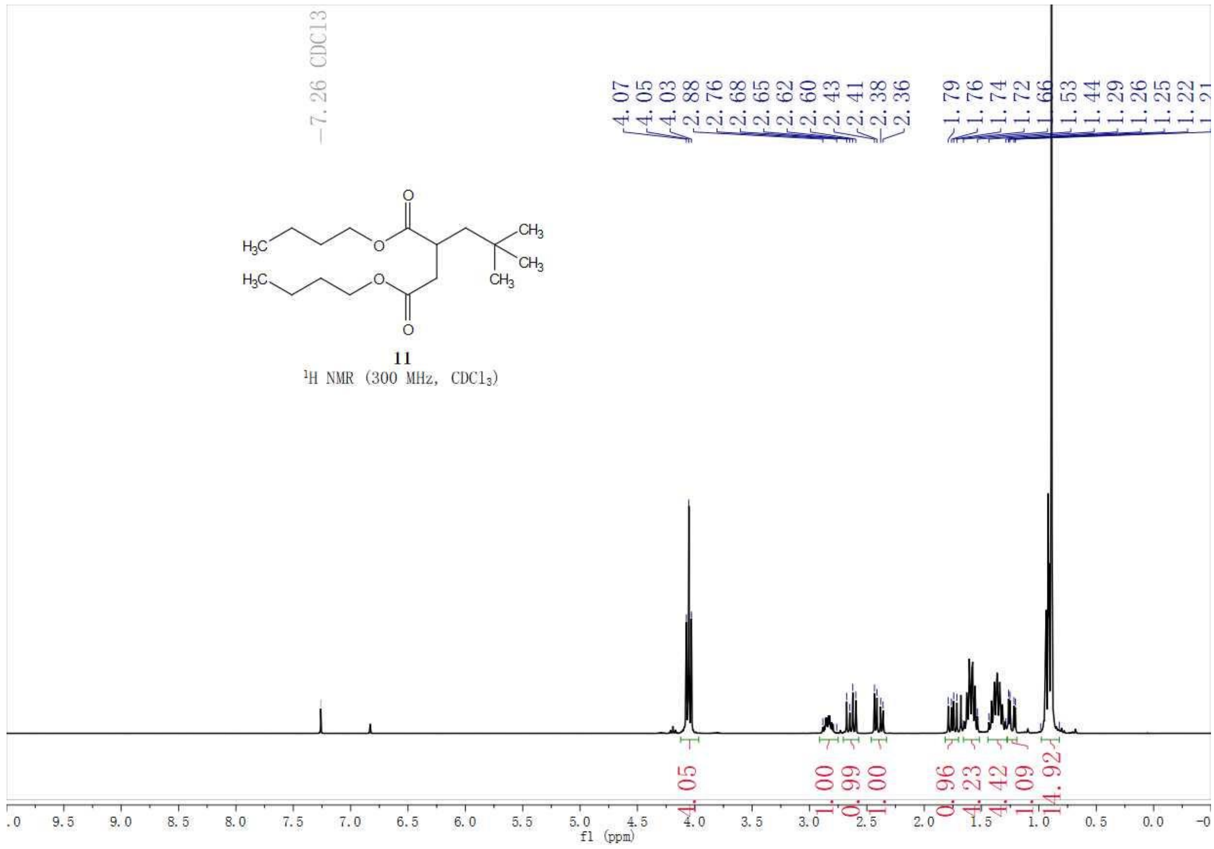

11

^{1}H NMR (300 MHz, $CDCl_3$)

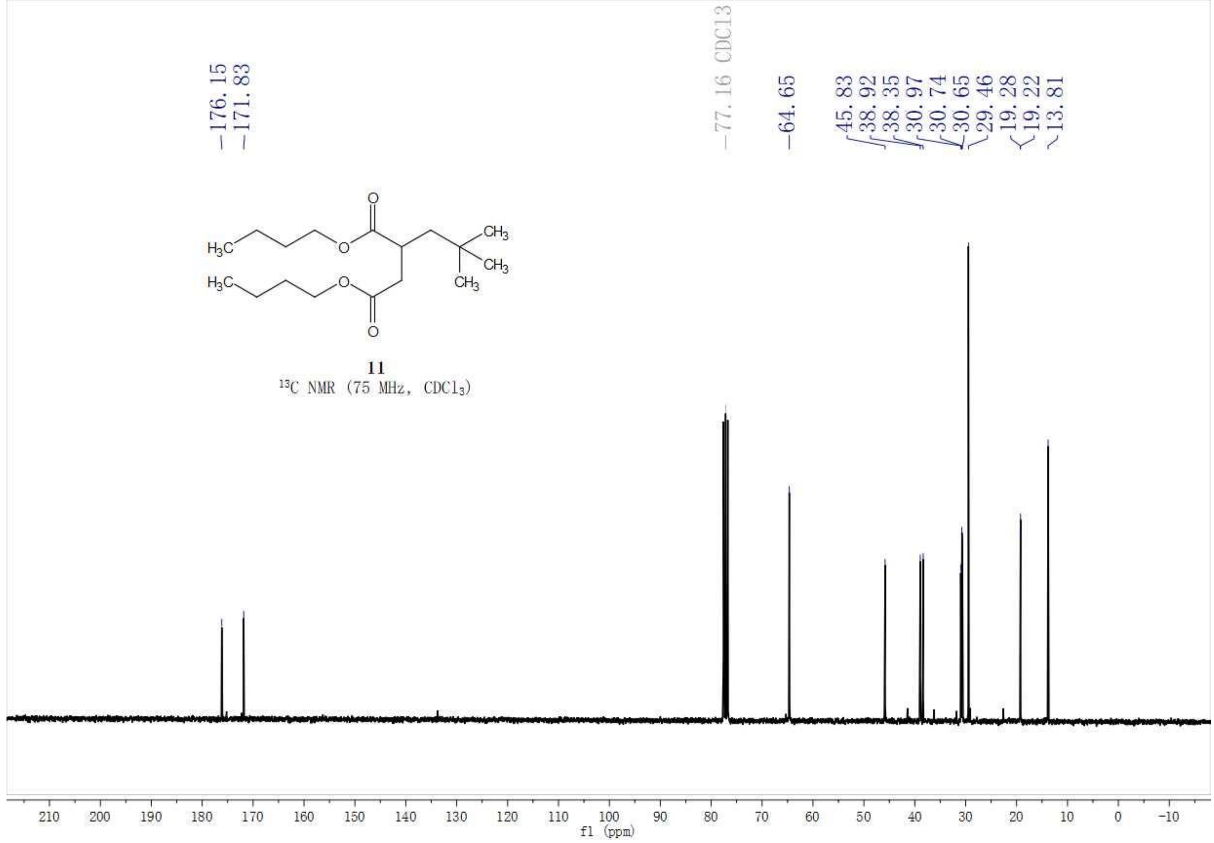

11

^{13}C NMR (75 MHz, $CDCl_3$)

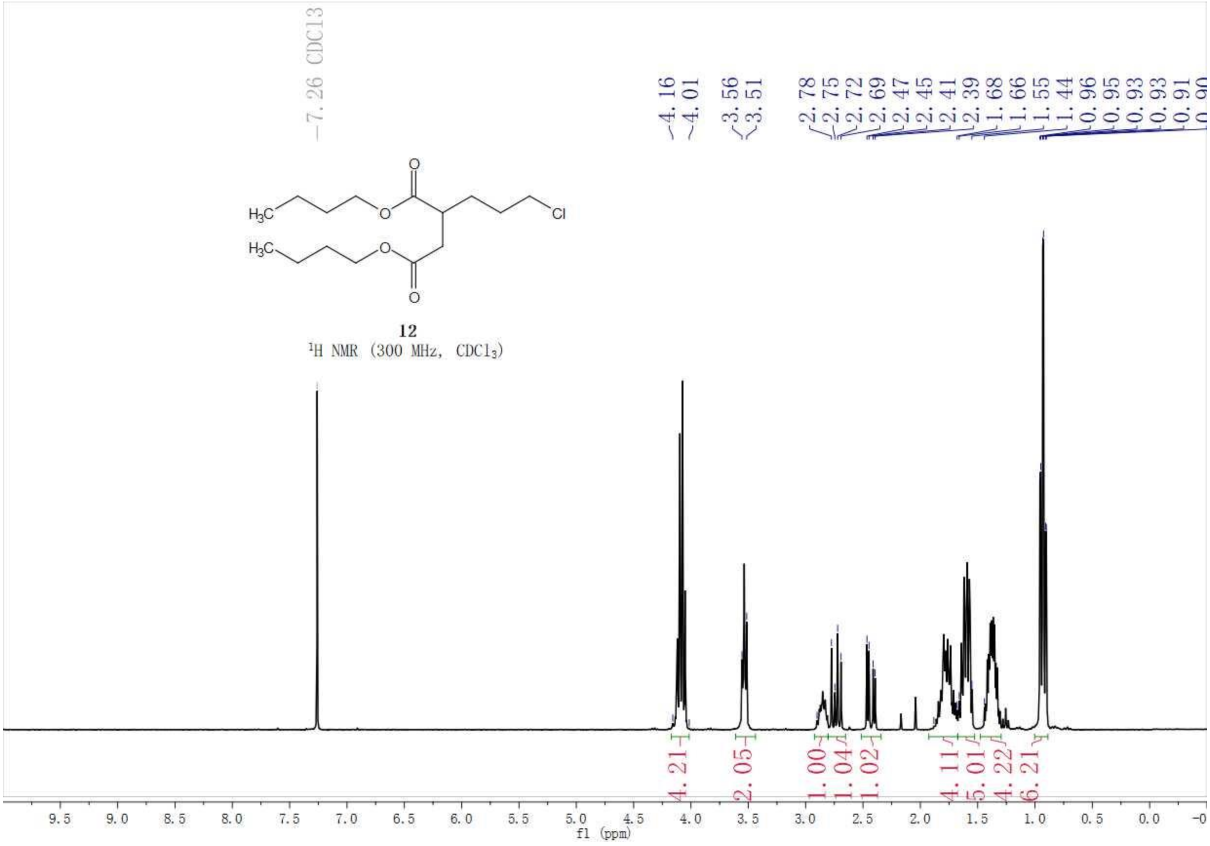

7.26 CDCl3
4.16
4.01
3.56
3.51
2.78
2.75
2.72
2.69
2.47
2.45
2.41
2.39
1.68
1.66
1.55
1.44
0.96
0.95
0.93
0.93
0.91
12
^{1}H NMR (300 MHz, CDCl$_3$)
4.21
2.05
1.00
1.04
1.02
4.11
5.01
4.22
6.21
9.5
9.0
8.5
8.0
7.5
7.0
6.5
6.0
5.5
5.0
4.5
4.0
3.5
3.0
2.5
2.0
1.5
1.0
0.5
0.0
f1 (ppm)

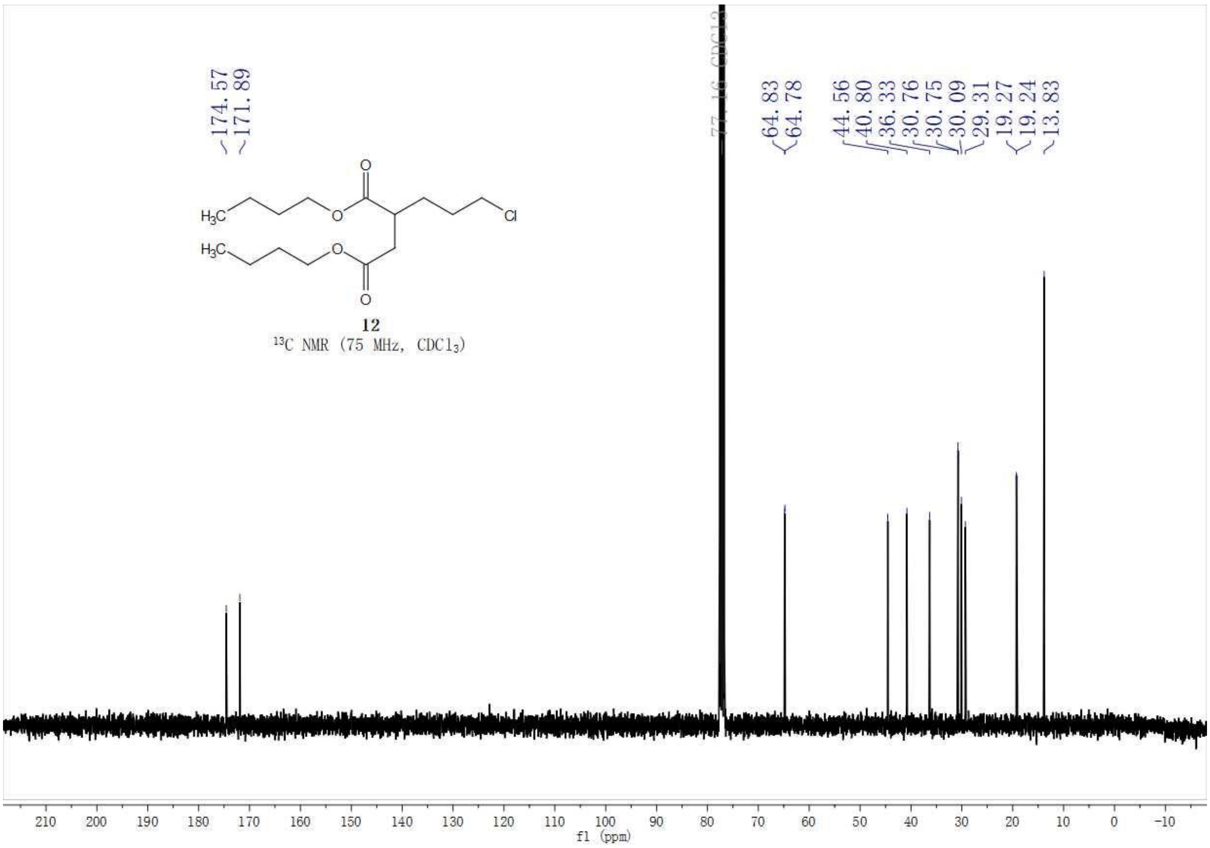

174.57
171.89
64.83
64.78
44.56
40.80
36.33
30.76
30.75
30.09
29.31
19.27
19.24
13.83
12
^{13}C NMR (75 MHz, CDCl$_3$)
210
200
190
180
170
160
150
140
130
120
110
100
90
80
70
60
50
40
30
20
10
0
-10
f1 (ppm)

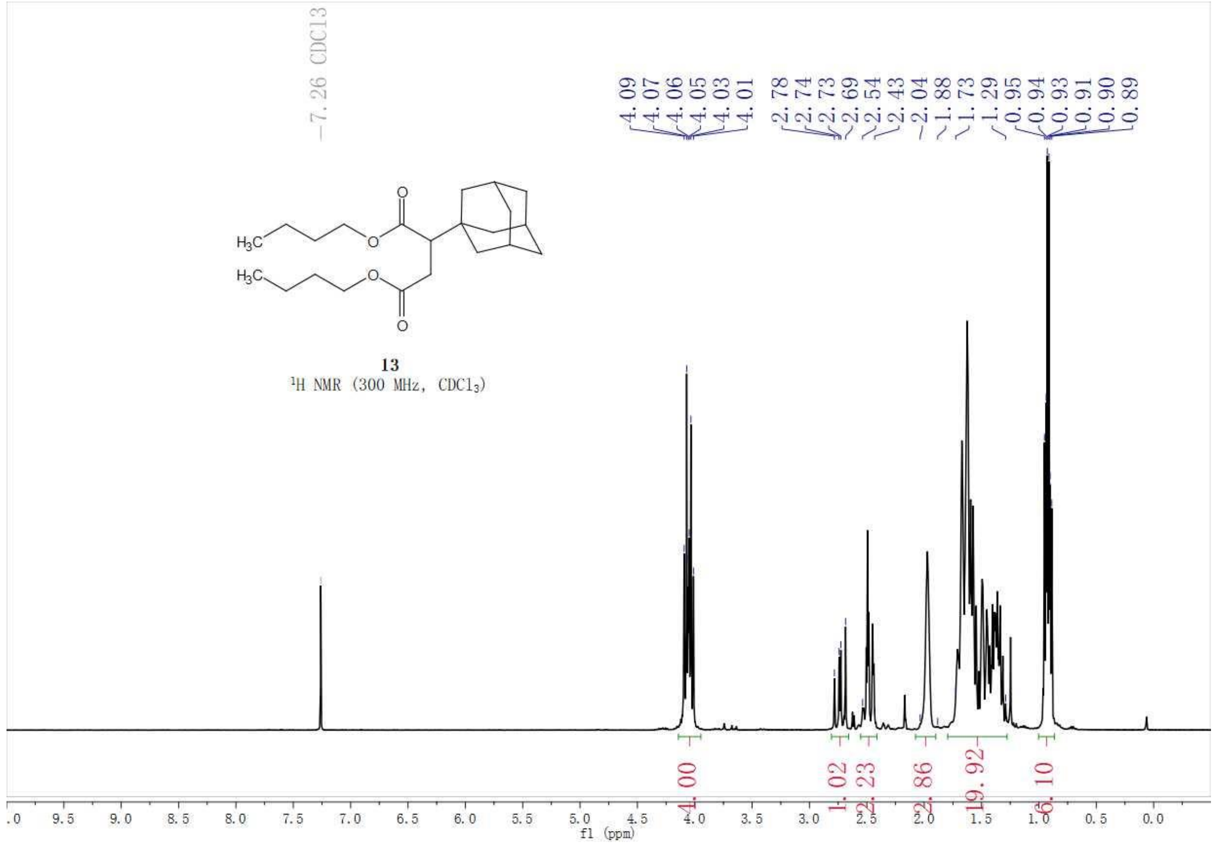
7.26 CDCl3
4.09
4.07
4.06
4.05
4.03
4.01
2.78
2.74
2.73
2.69
2.54
2.43
2.04
1.88
1.73
1.29
0.95
0.94
0.93
0.91
0.90
0.89
H3C
H3C
13
1H NMR (300 MHz, CDCl3)
4.00
1.02
2.23
2.86
19.92
6.10
f1 (ppm)

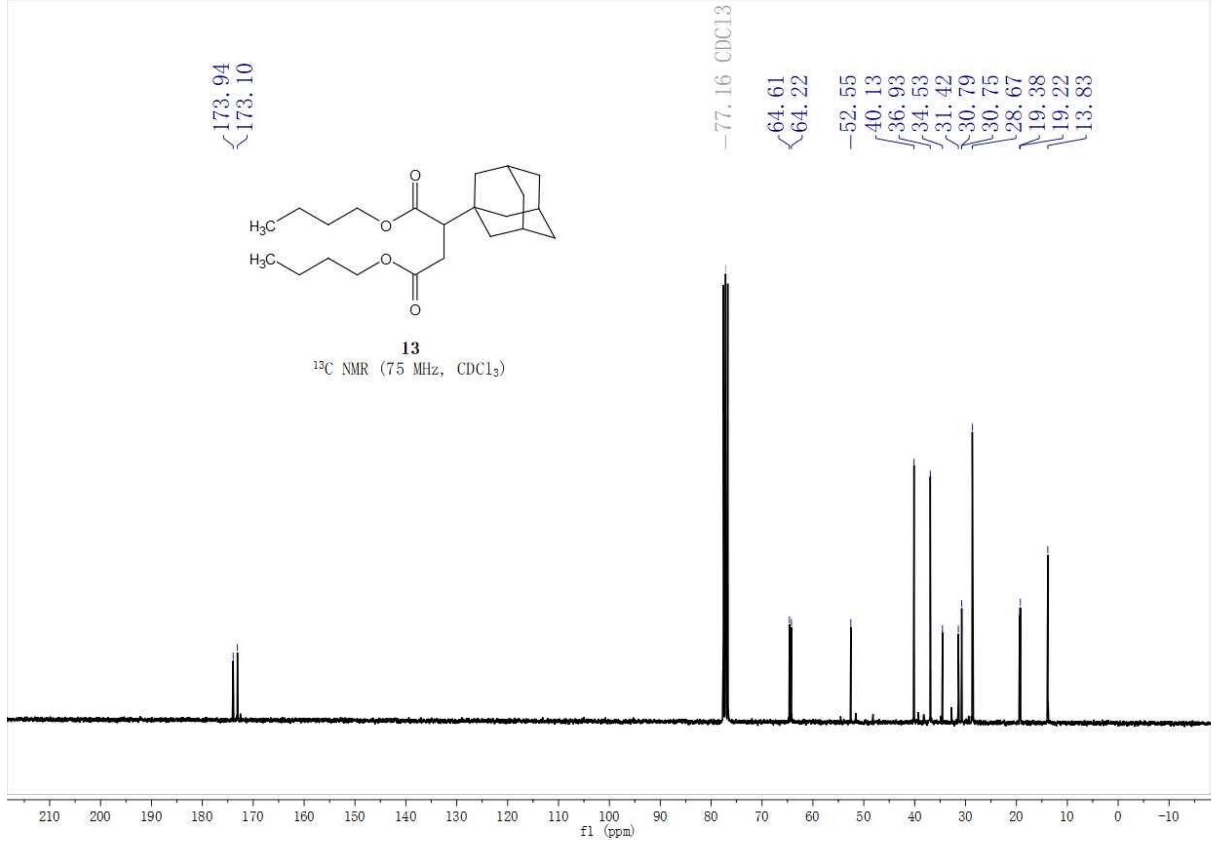
173.94
173.10
77.16 CDCl3
64.61
64.22
52.55
40.13
36.93
34.53
31.42
30.79
30.75
28.67
19.38
19.22
13.83
H3C
H3C
13
13C NMR (75 MHz, CDCl3)
f1 (ppm)

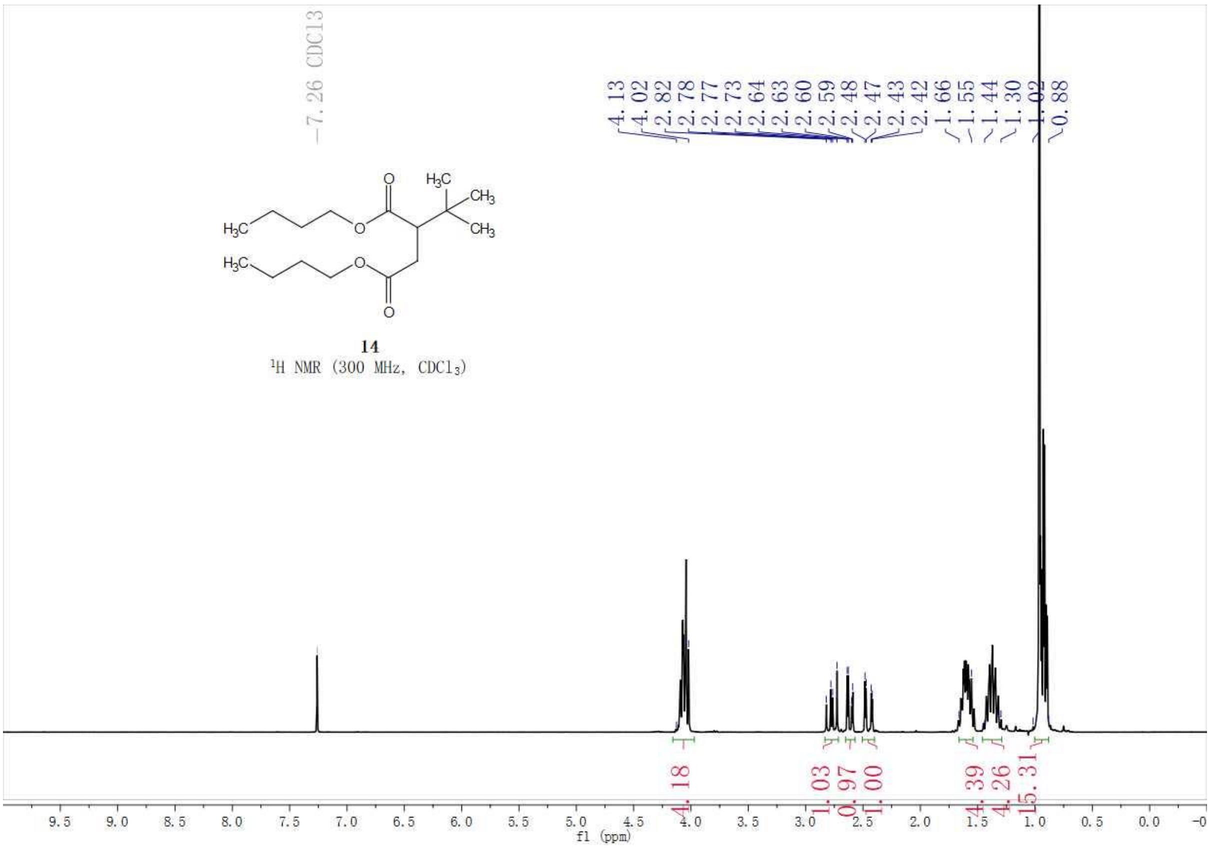

14
^{1}H NMR (300 MHz, $CDCl_3$)

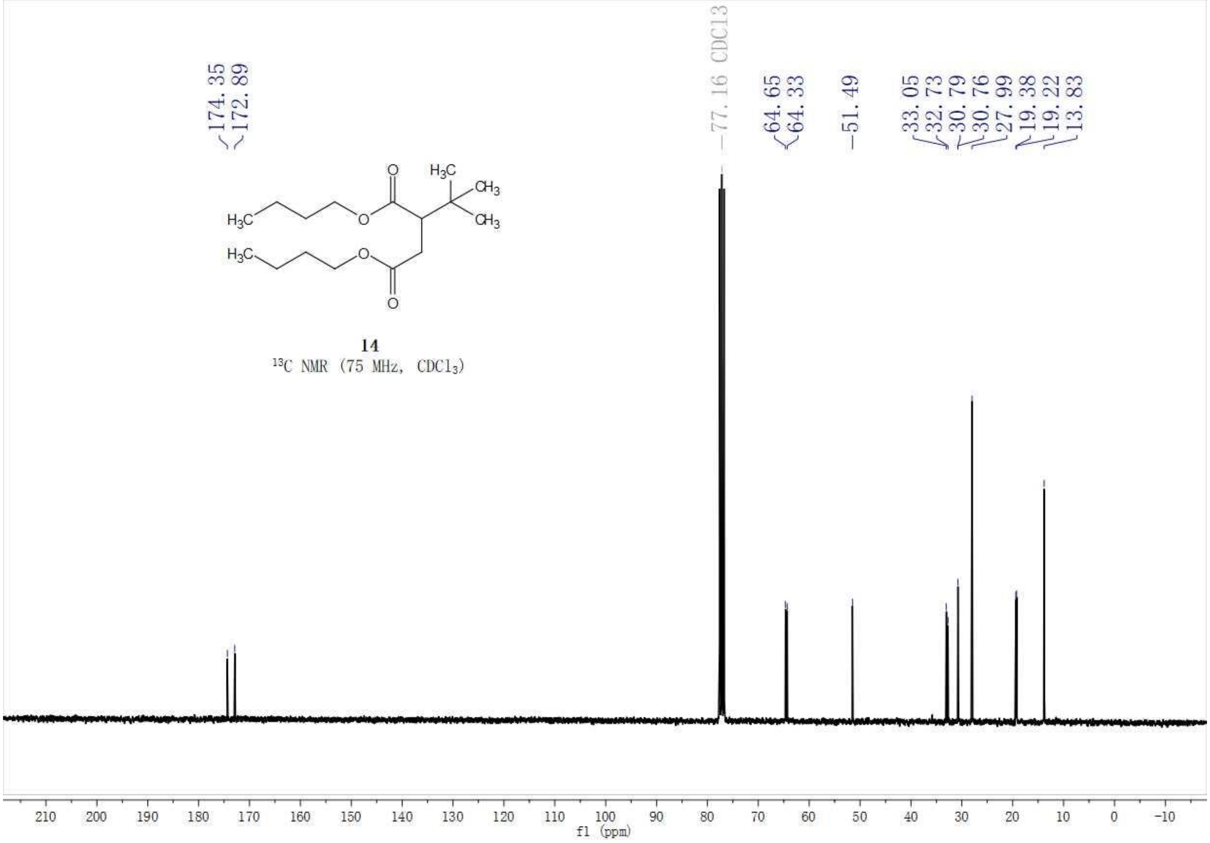

14
^{13}C NMR (75 MHz, $CDCl_3$)

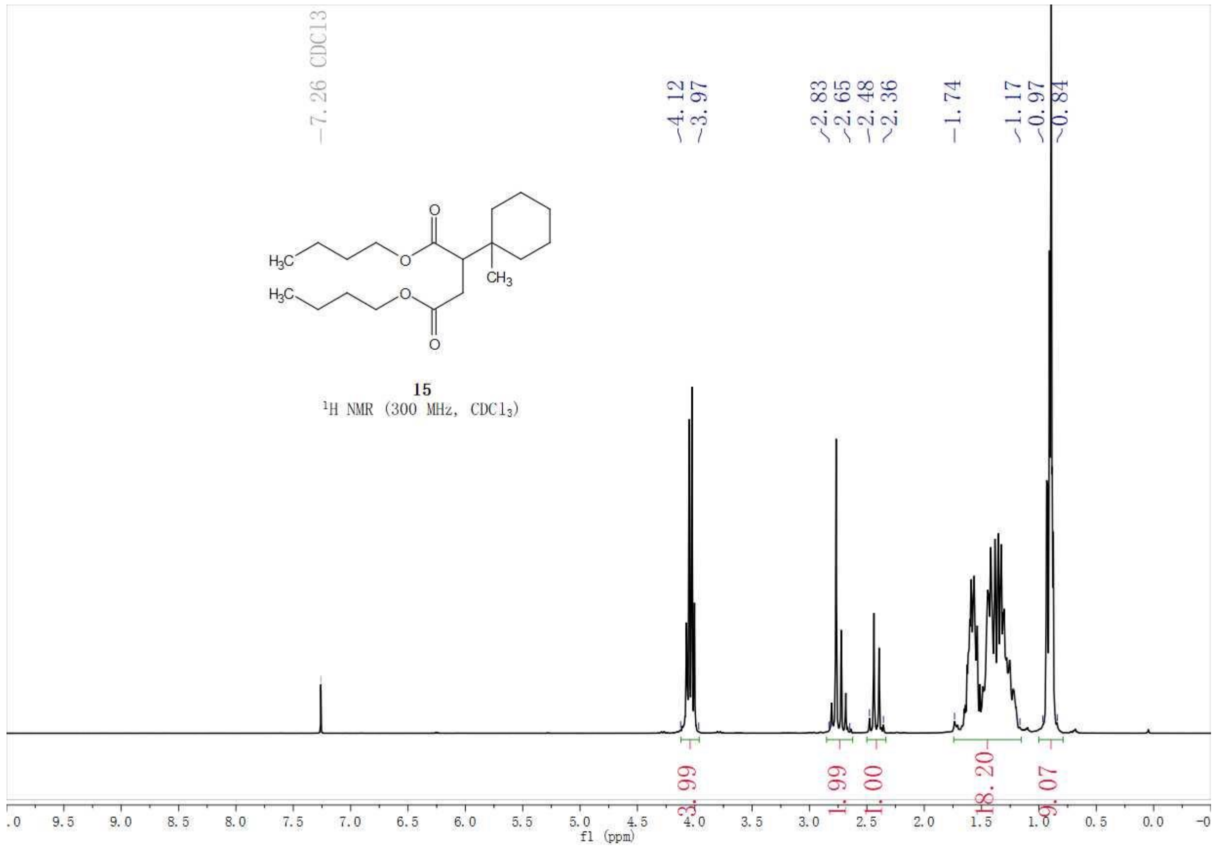

15
^{1}H NMR (300 MHz, $CDCl_3$)

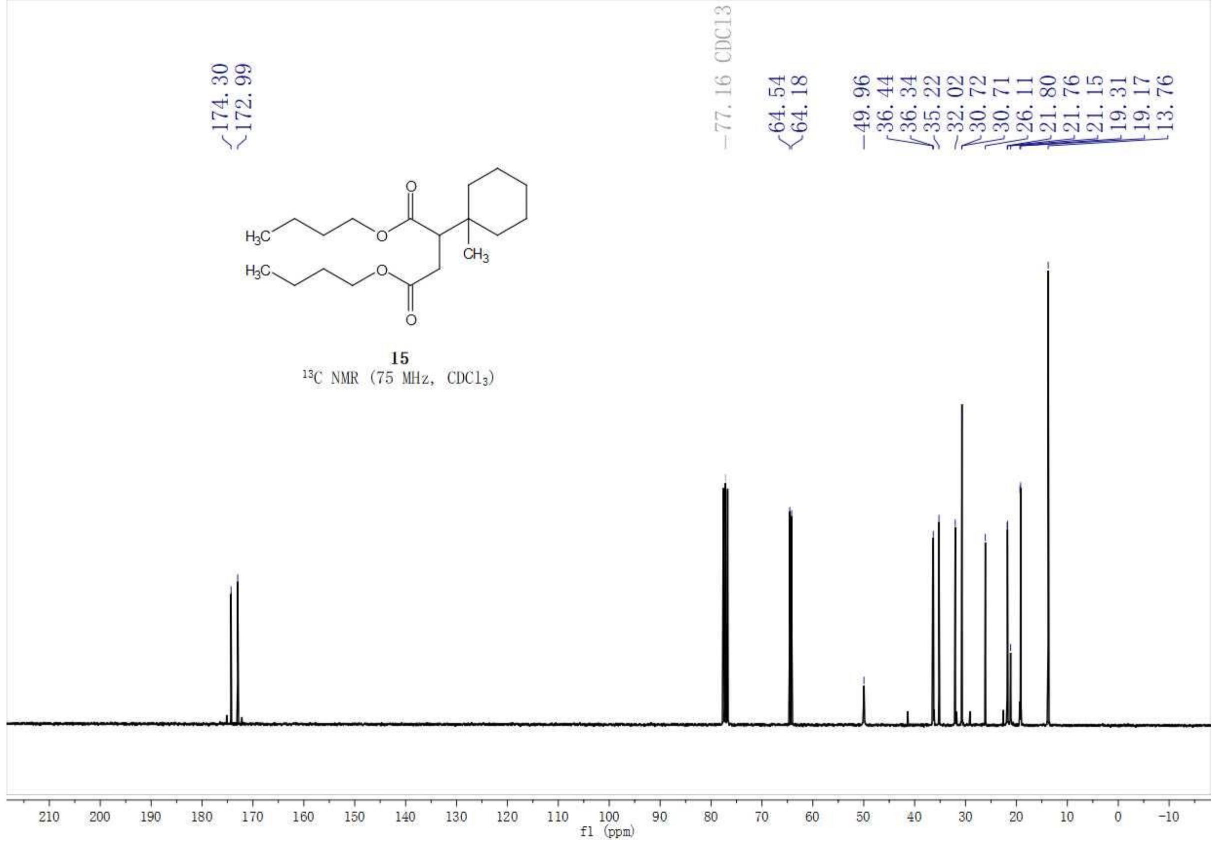

15
^{13}C NMR (75 MHz, $CDCl_3$)

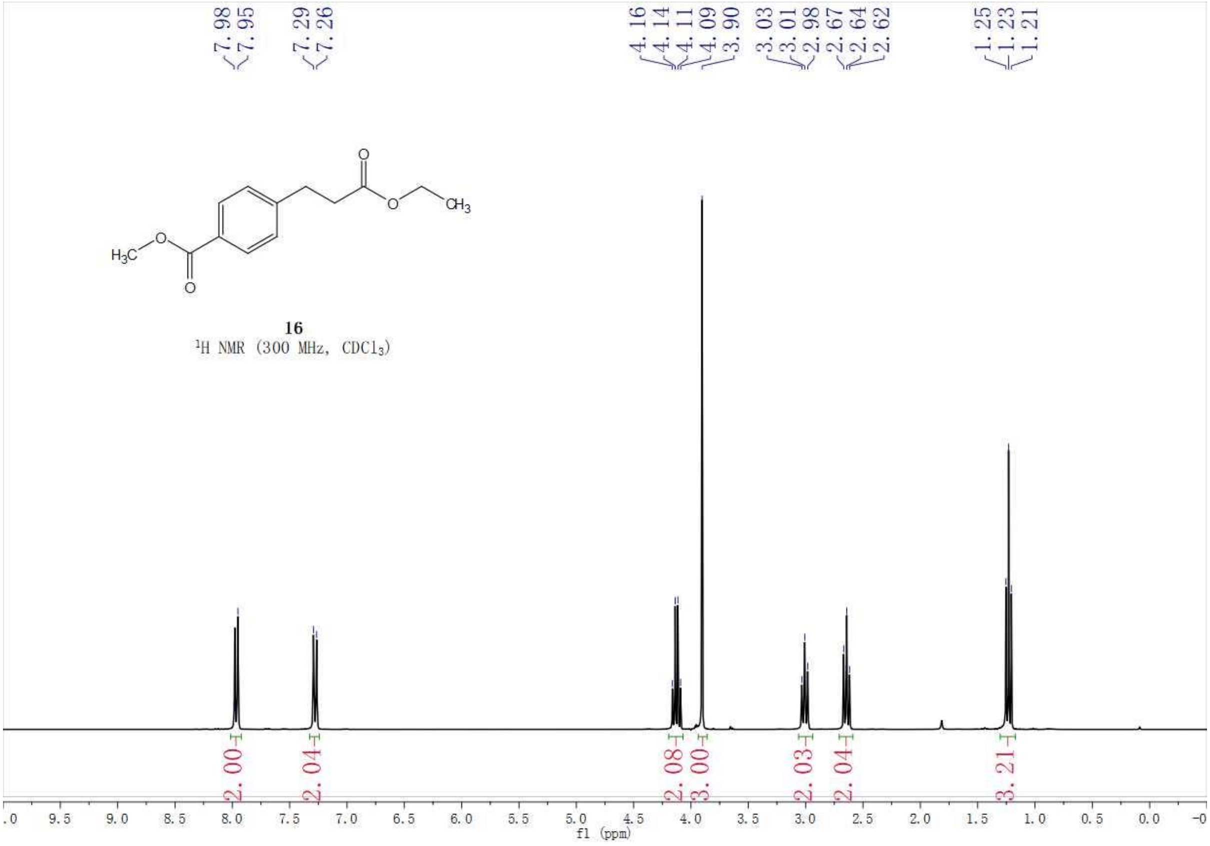
7.98
7.95
7.29
7.26
4.16
4.14
4.11
4.09
3.90
3.03
3.01
2.98
2.67
2.64
2.62
1.25
1.23
1.21
16
1H NMR (300 MHz, CDCl3)
2.00
2.04
2.08
3.00
2.03
2.04
3.21
f1 (ppm)

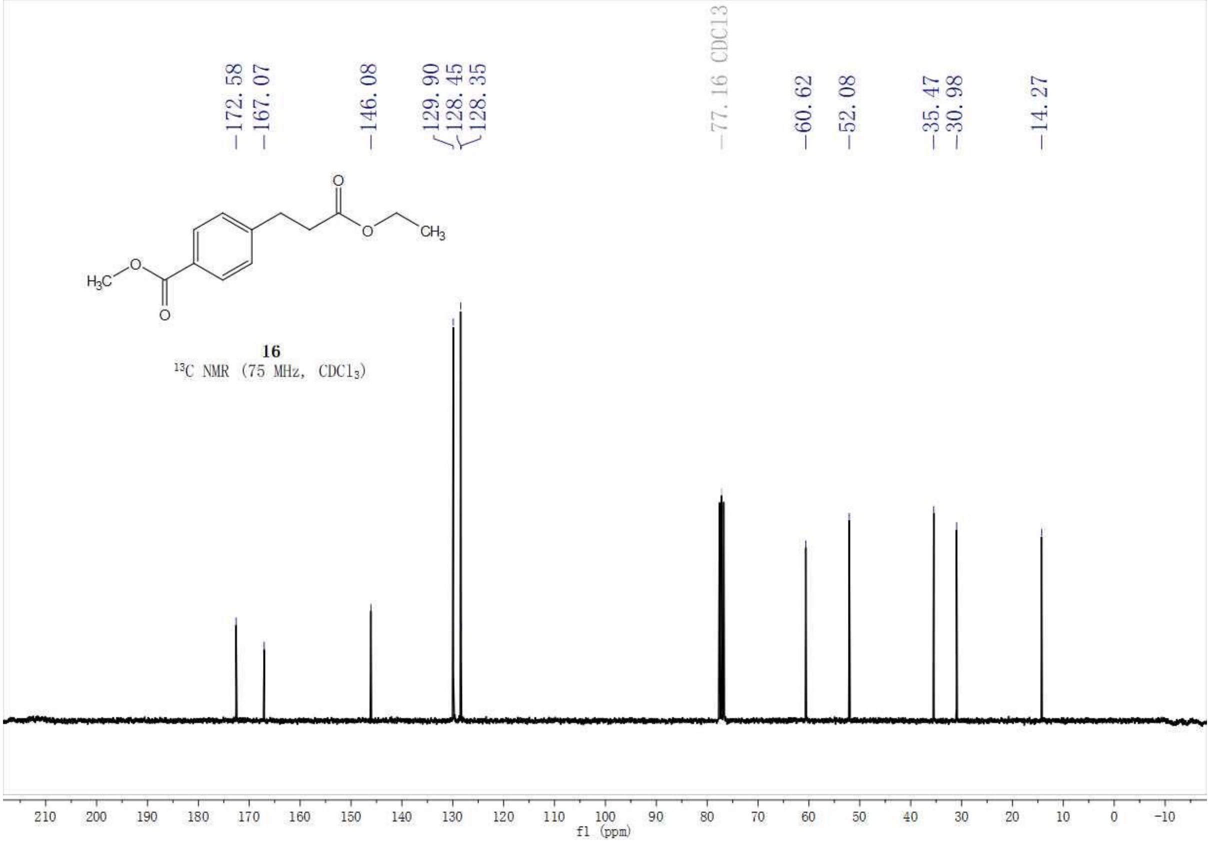
172.58
167.07
146.08
129.90
128.45
128.35
77.16 CDCl3
60.62
52.08
35.47
30.98
14.27
16
13C NMR (75 MHz, CDCl3)
f1 (ppm)

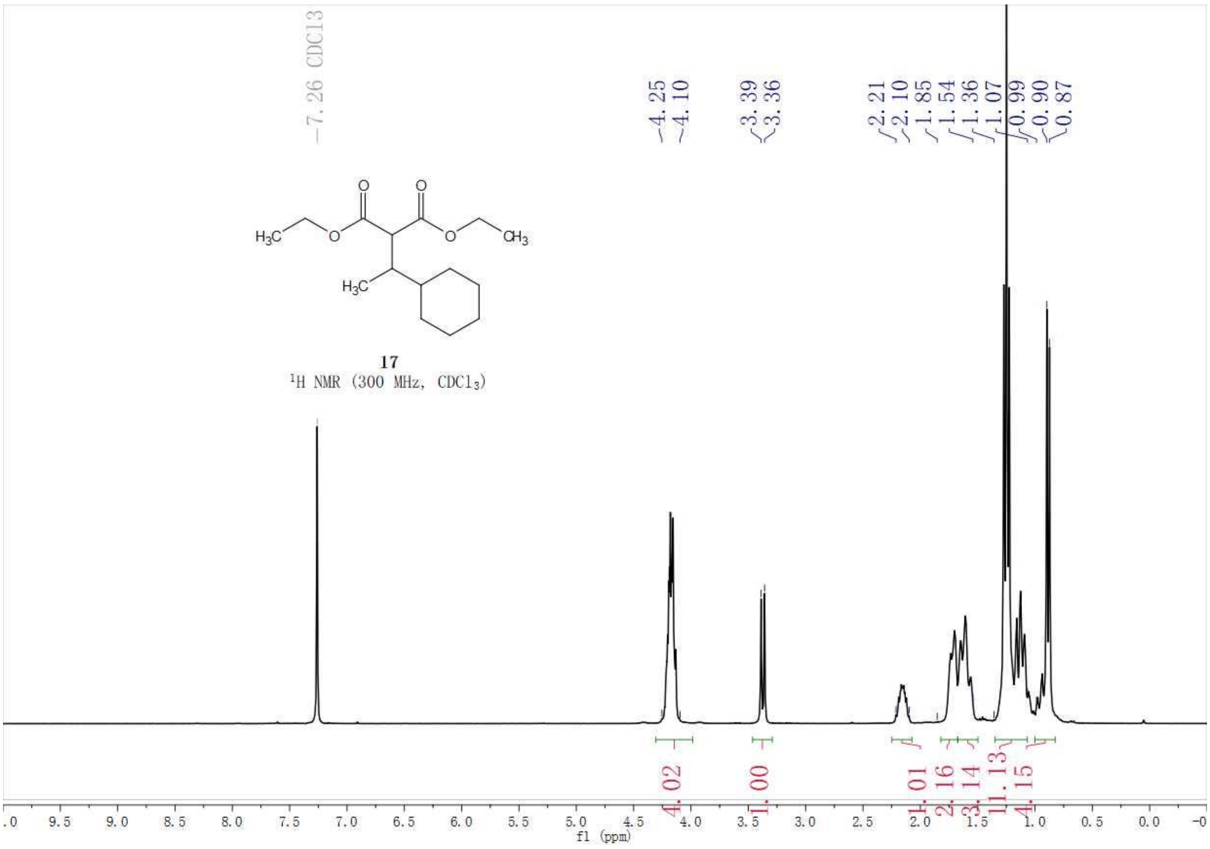
7.26 CDCl3
4.25
4.10
3.39
3.36
2.21
2.10
1.85
1.54
1.36
1.07
0.99
0.90
0.87
17
^{1}H NMR (300 MHz, CDCl$_3$)
4.02
1.00
1.01
2.16
3.14
11.13
4.15
fl (ppm)

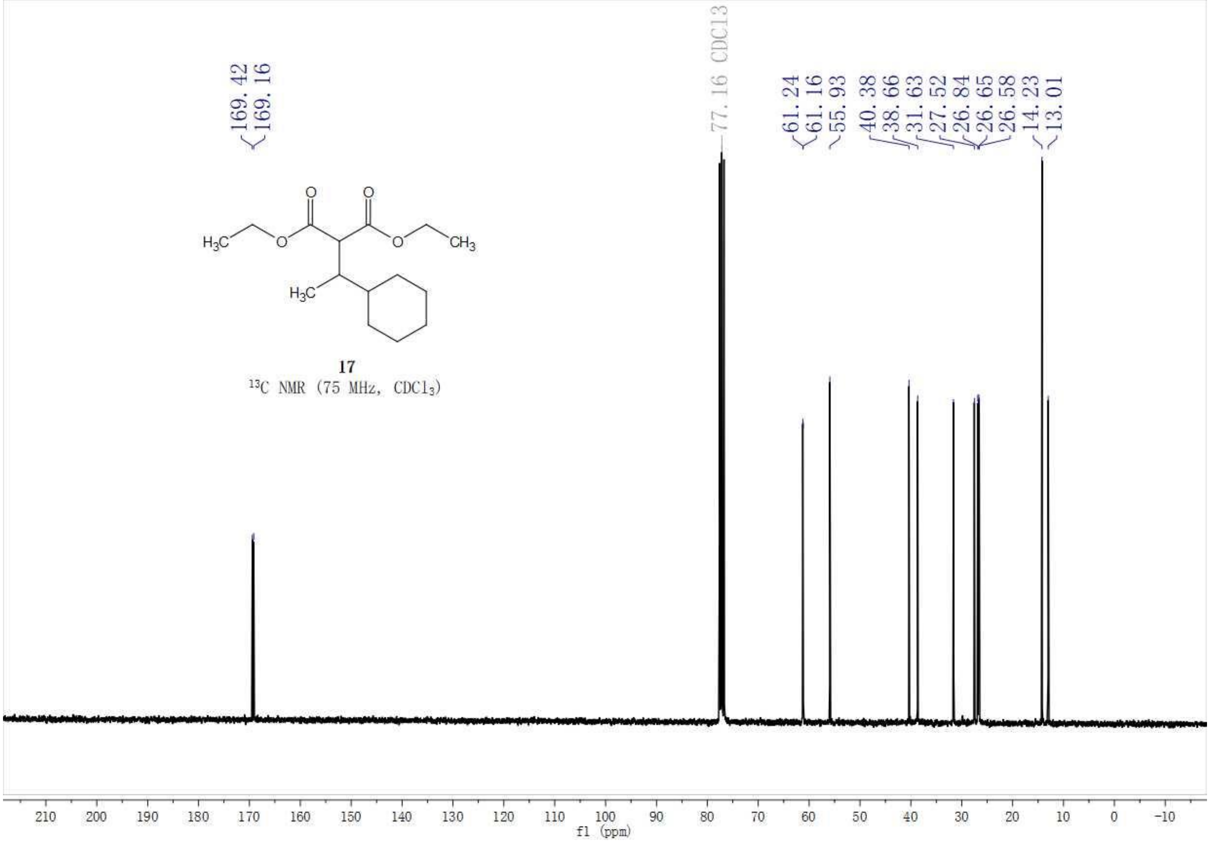
169.42
169.16
77.16 CDCl3
61.24
61.16
55.93
40.38
38.66
31.63
27.52
26.84
26.65
26.58
14.23
13.01
17
^{13}C NMR (75 MHz, CDCl$_3$)
fl (ppm)

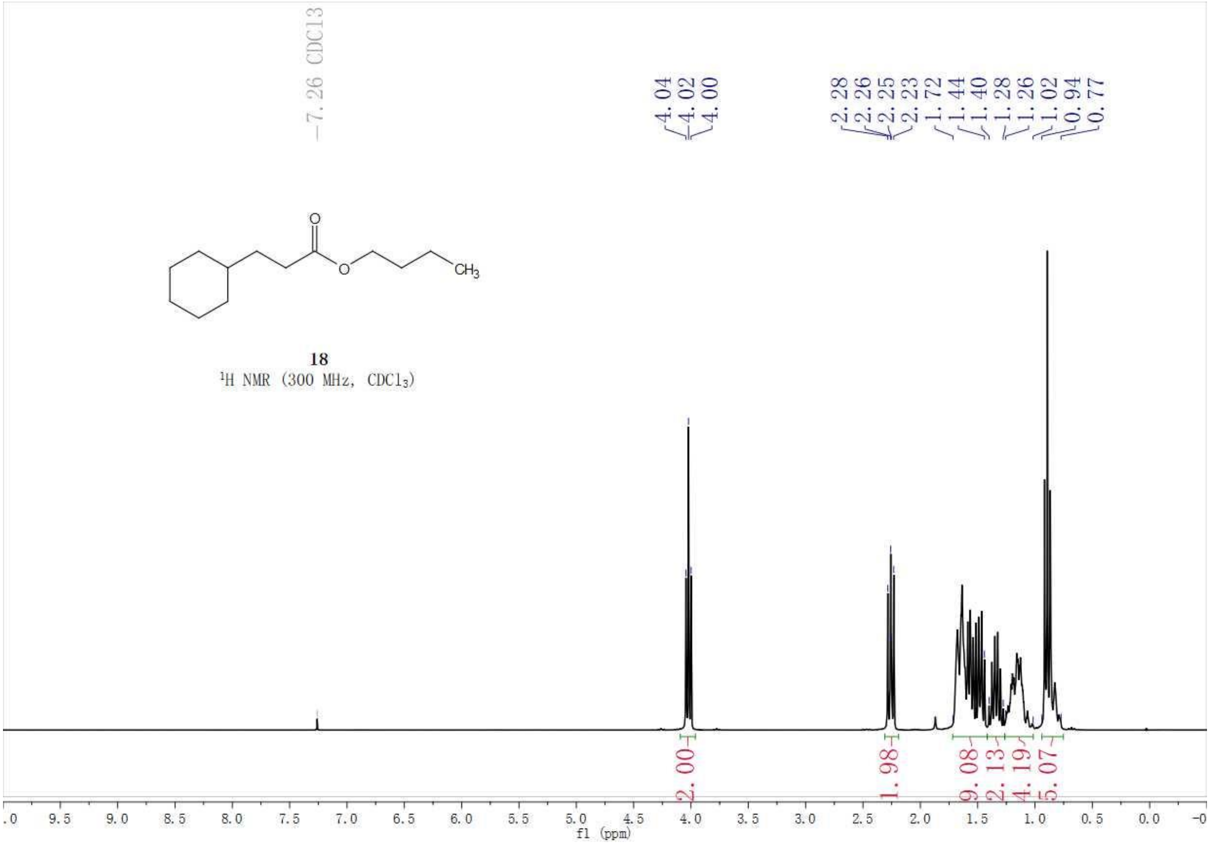

7.26 CDCl3
4.04
4.02
4.00
2.28
2.26
2.25
2.23
1.72
1.44
1.40
1.28
1.26
1.02
0.94
0.77
CH3
18
1H NMR (300 MHz, CDCl3)
2.00
1.98
9.08
2.13
4.19
5.07
f1 (ppm)

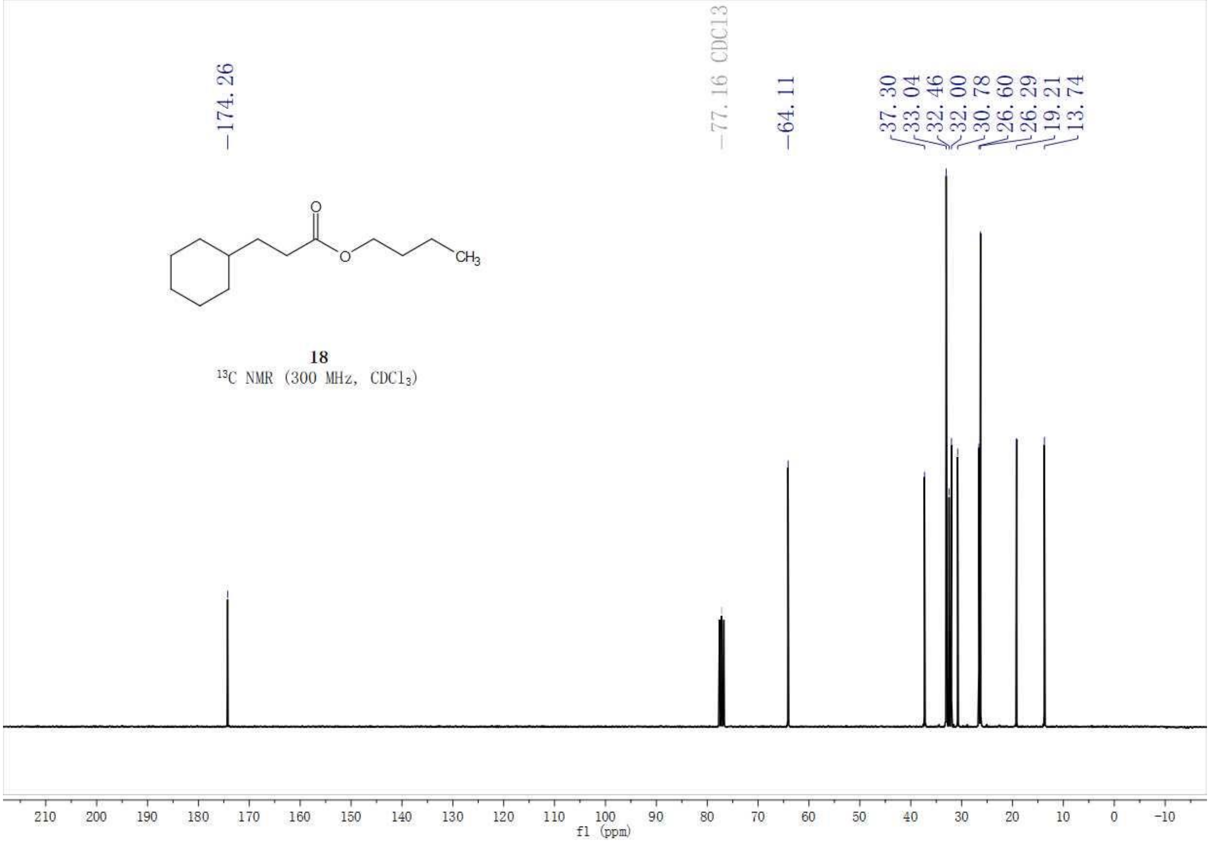

174.26
77.16 CDCl3
64.11
37.30
33.04
32.46
32.00
30.78
26.60
26.29
19.21
13.74
CH3
18
13C NMR (300 MHz, CDCl3)
f1 (ppm)

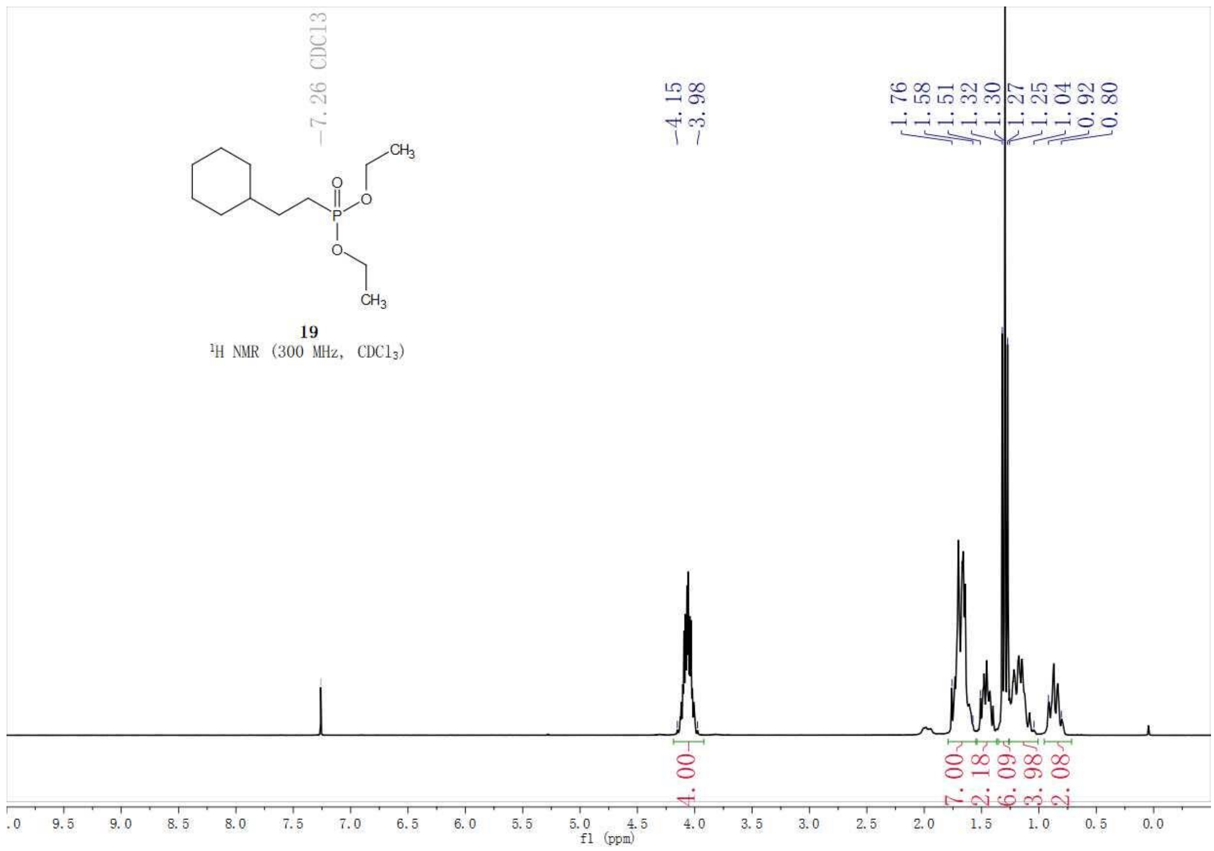

-7.26 CDCl3
4.15
3.98
1.76
1.58
1.51
1.32
1.30
1.27
1.25
1.04
0.92
0.80
CH3
O
P
O
O
CH3
19
1H NMR (300 MHz, CDCl3)
4.00
7.00
2.18
6.09
3.98
2.08
fl (ppm)

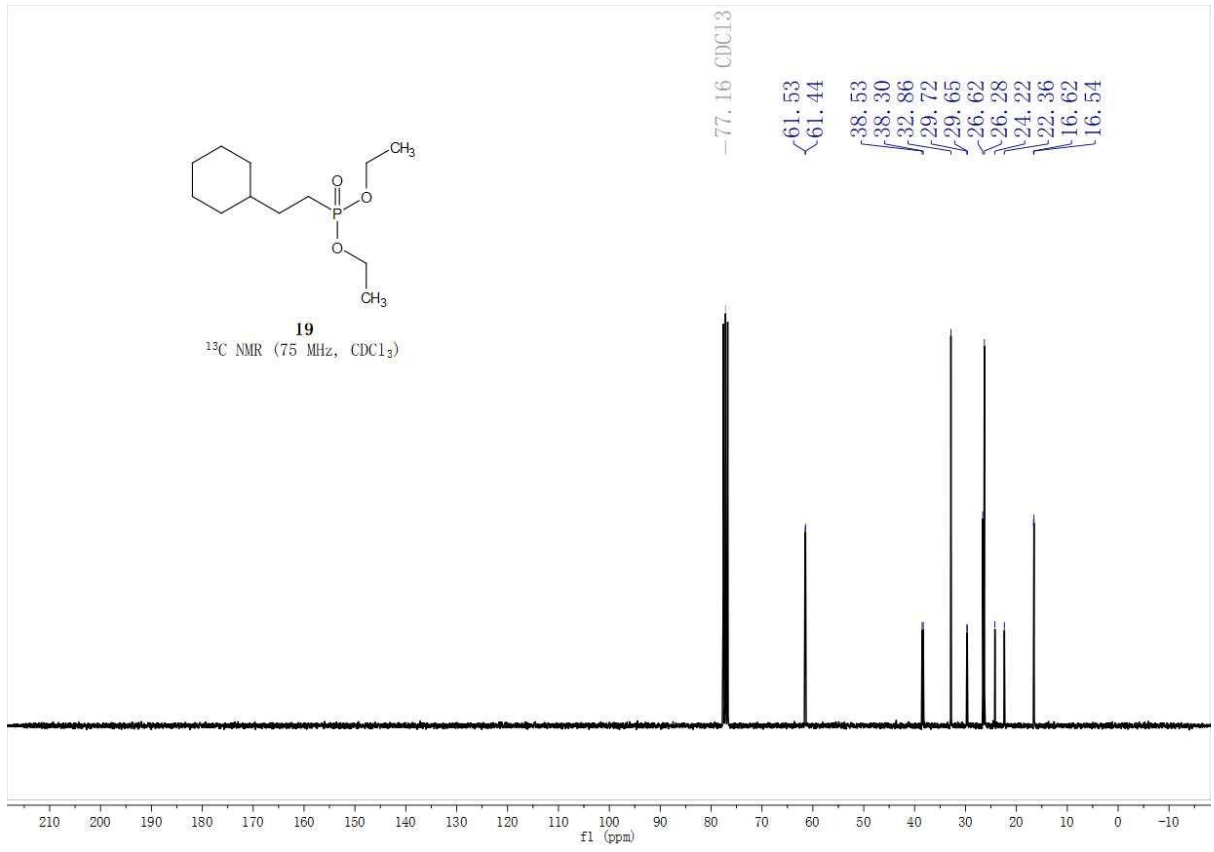

-77.16 CDCl3
61.53
61.44
38.53
38.30
32.86
29.72
29.65
26.62
26.28
24.22
22.36
16.62
16.54
CH3
O
P
O
O
CH3
19
13C NMR (75 MHz, CDCl3)
fl (ppm)

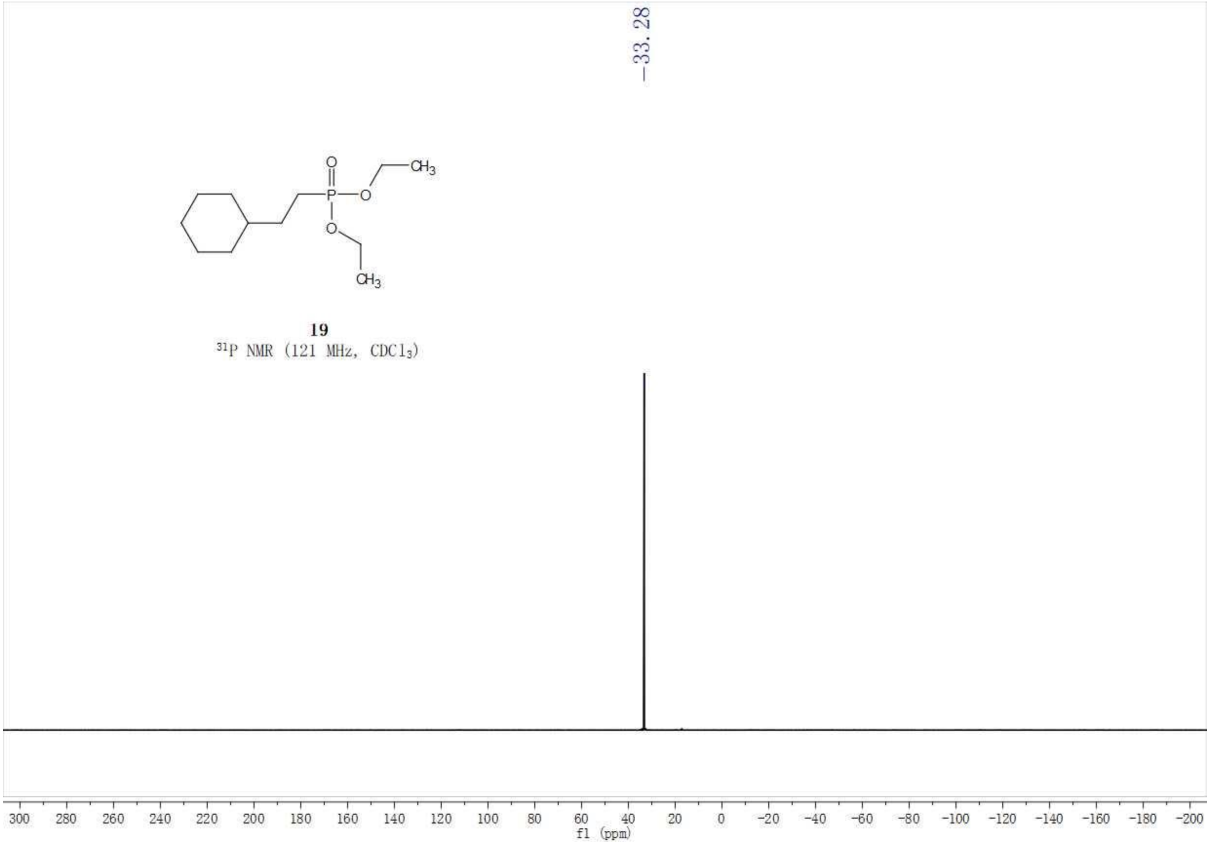

33.28
19
^{31}P NMR (121 MHz, CDCl$_3$)
300
280
260
240
220
200
180
160
140
120
100
80
60
40
20
0
-20
-40
-60
-80
-100
-120
-140
-160
-180
-200
f1 (ppm)

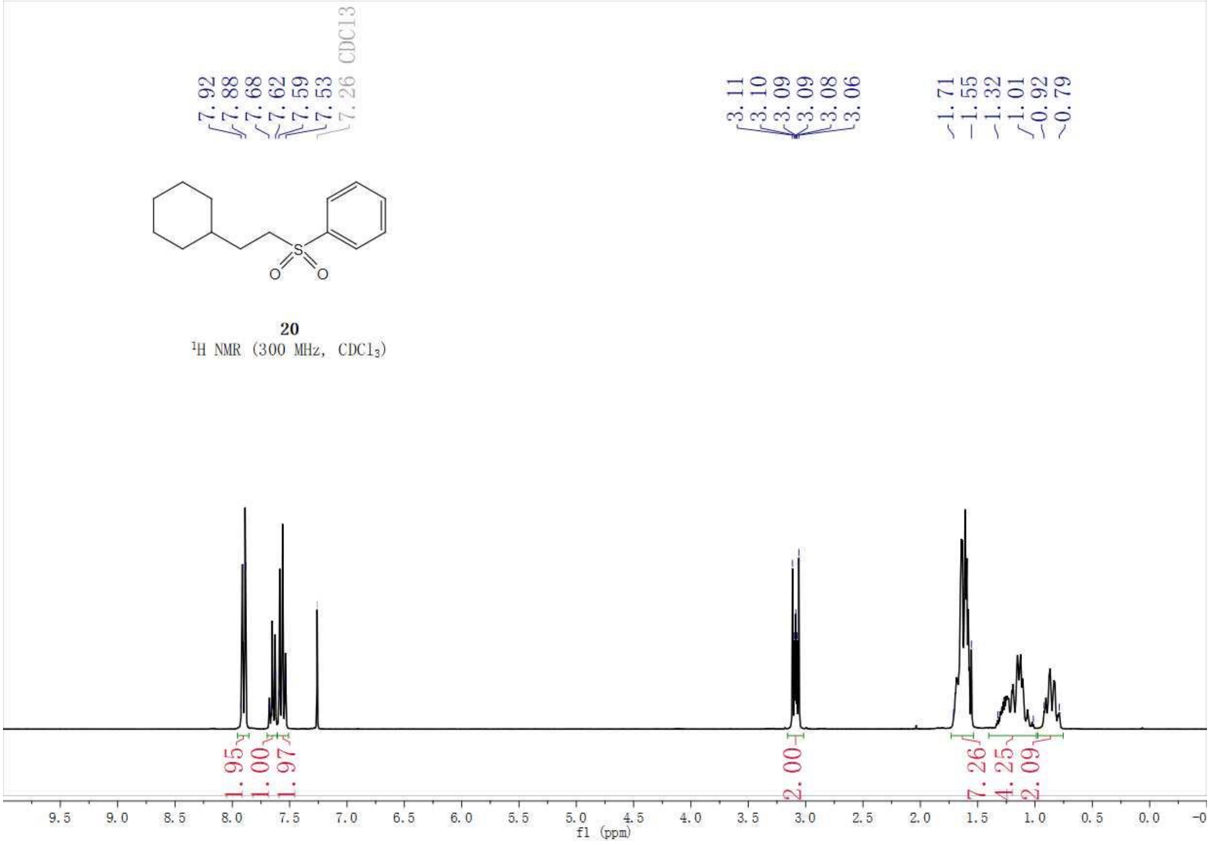
7.92
7.88
7.68
7.62
7.59
7.53
7.26 CDCl3
3.11
3.10
3.09
3.09
3.08
3.06
1.71
1.55
1.32
1.01
0.92
0.79
O
S
O
20
^{1}H NMR (300 MHz, CDCl$_3$)
1.95
1.00
1.97
2.00
7.26
4.25
2.09
9.5 9.0 8.5 8.0 7.5 7.0 6.5 6.0 5.5 5.0 4.5 4.0 3.5 3.0 2.5 2.0 1.5 1.0 0.5 0.0
f1 (ppm)

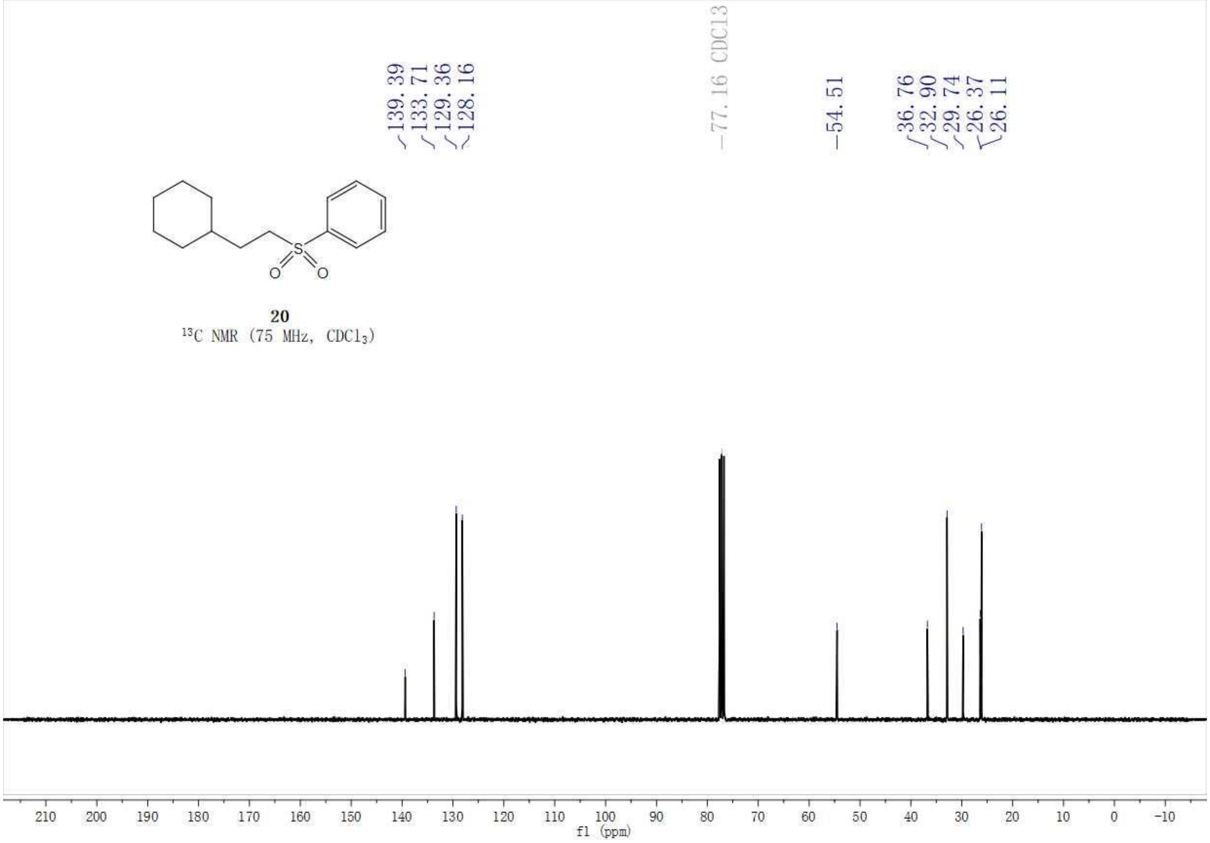
139.39
133.71
129.36
128.16
77.16 CDCl3
54.51
36.76
32.90
29.74
26.37
26.11
O
S
O
20
^{13}C NMR (75 MHz, CDCl$_3$)
210 200 190 180 170 160 150 140 130 120 110 100 90 80 70 60 50 40 30 20 10 0 -10
f1 (ppm)

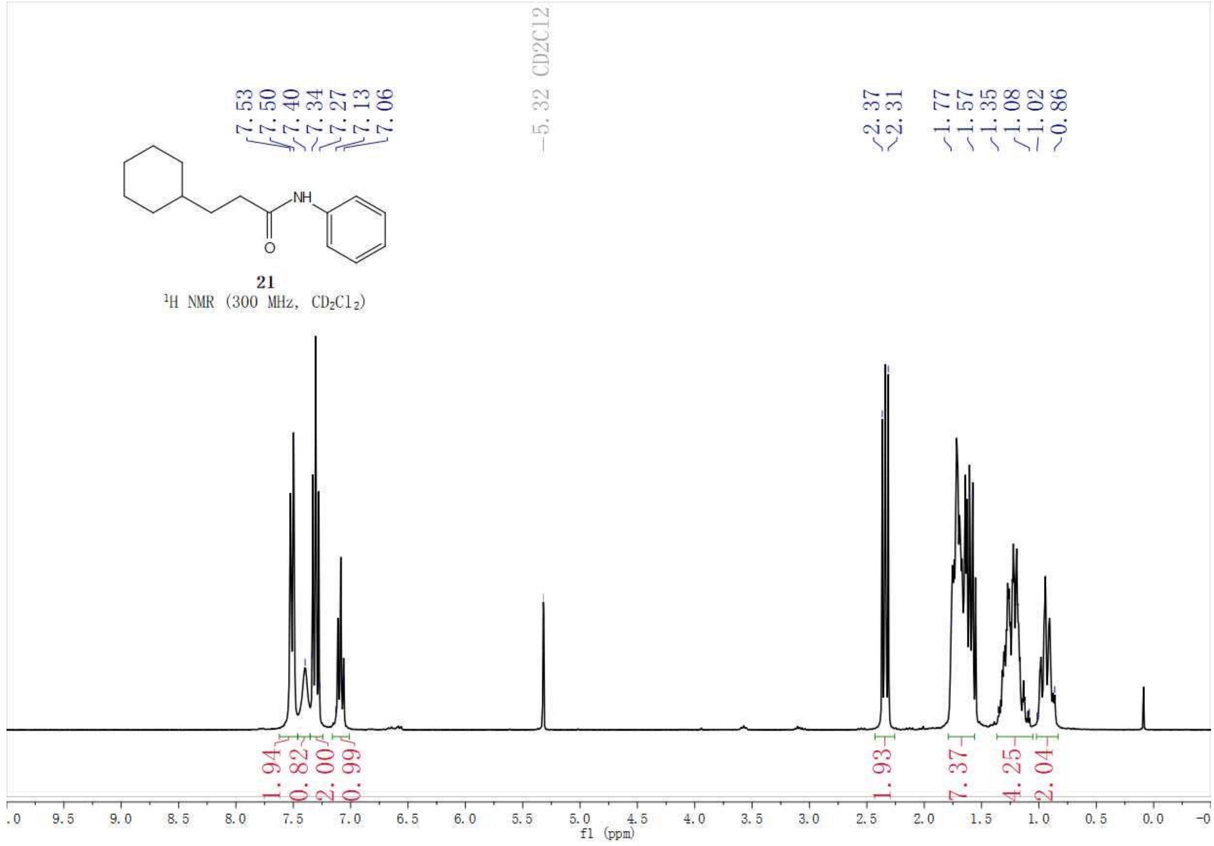
7.53
7.50
7.40
7.34
7.27
7.13
7.06
5.32 CD2Cl2
2.37
2.31
1.77
1.57
1.35
1.08
1.02
0.86
NH
O
21
^{1}H NMR (300 MHz, CD_2Cl_2)
1.94
0.82
2.00
0.99
1.93
7.37
4.25
2.04
f1 (ppm)

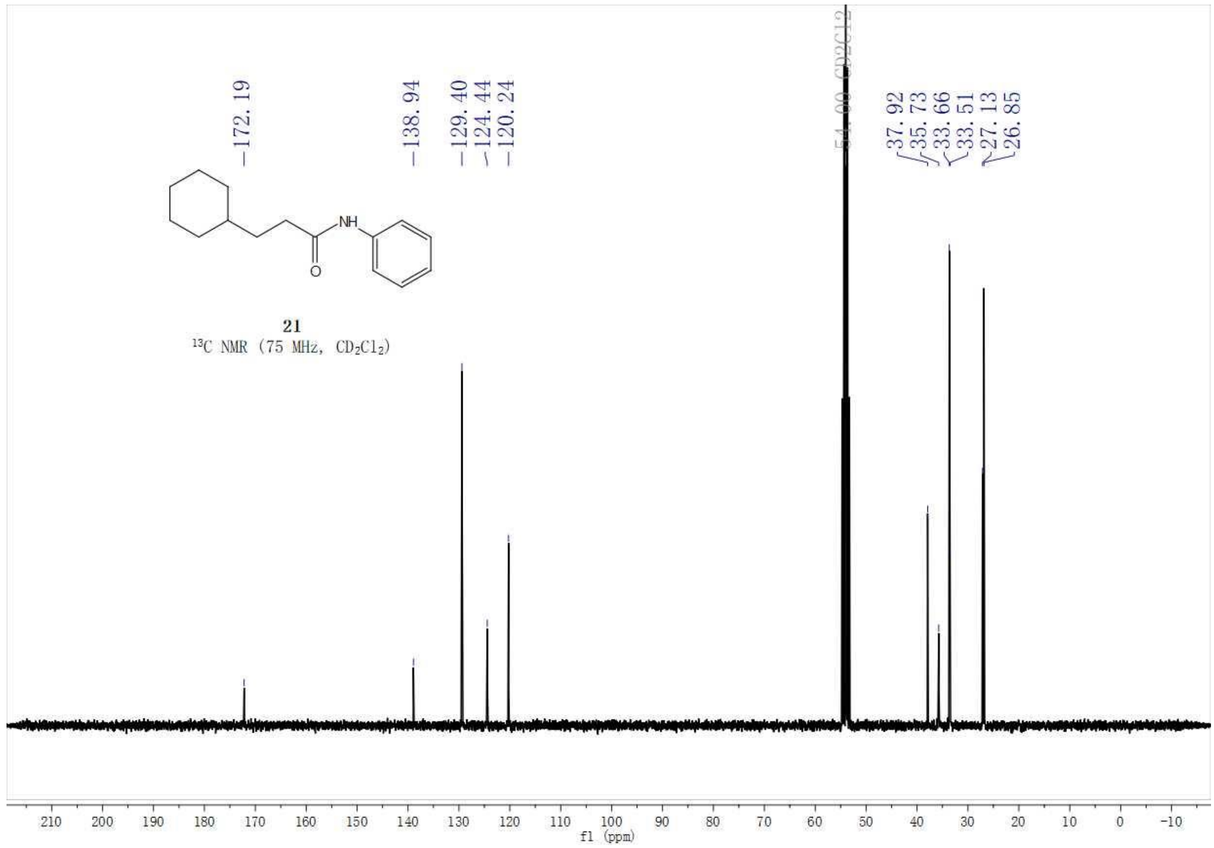
172.19
138.94
129.40
124.44
120.24
54.00 CD2Cl2
37.92
35.73
33.66
33.51
27.13
26.85
NH
O
21
^{13}C NMR (75 MHz, CD_2Cl_2)
f1 (ppm)

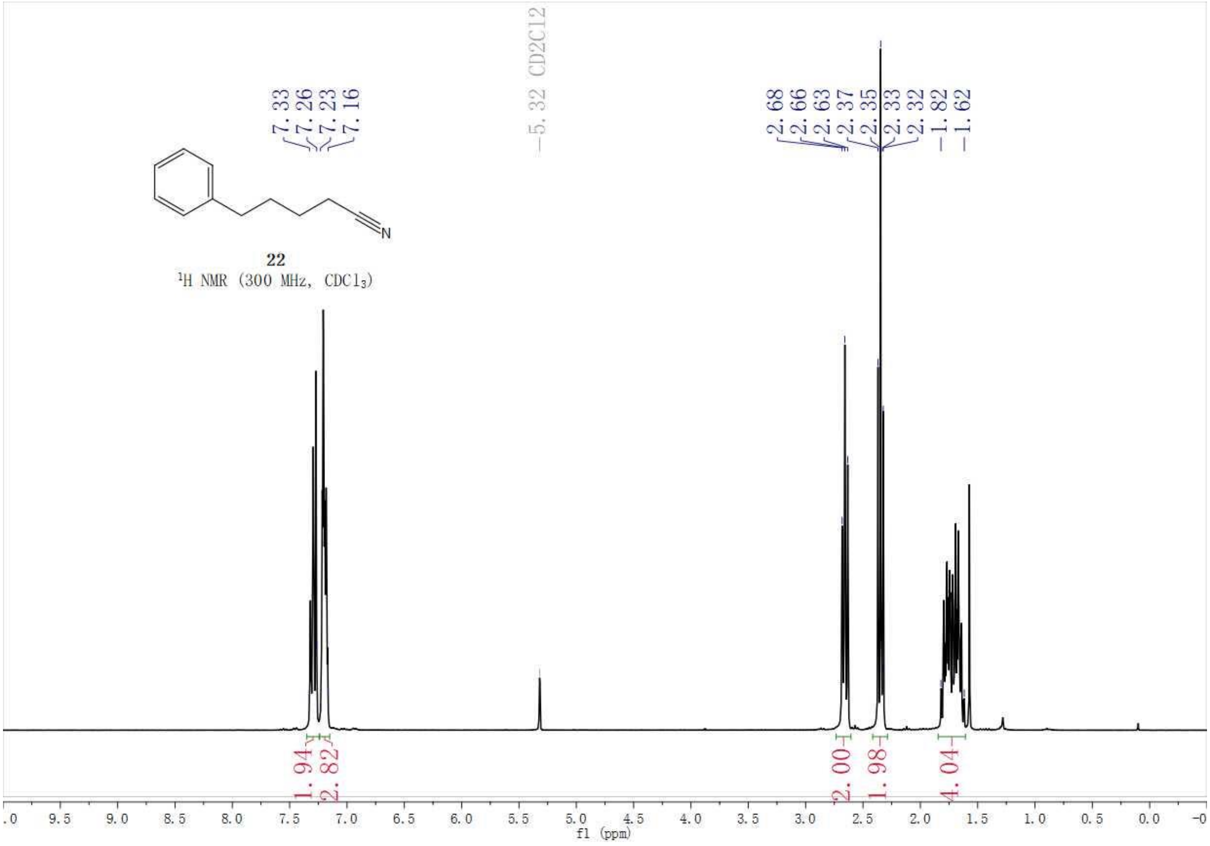
7.33
7.26
7.23
7.16
5.32 CD2Cl2
2.68
2.66
2.63
2.37
2.35
2.33
2.32
1.82
1.62
N
22
1H NMR (300 MHz, CDCl3)
1.94
2.82
2.00
1.98
4.04
f1 (ppm)

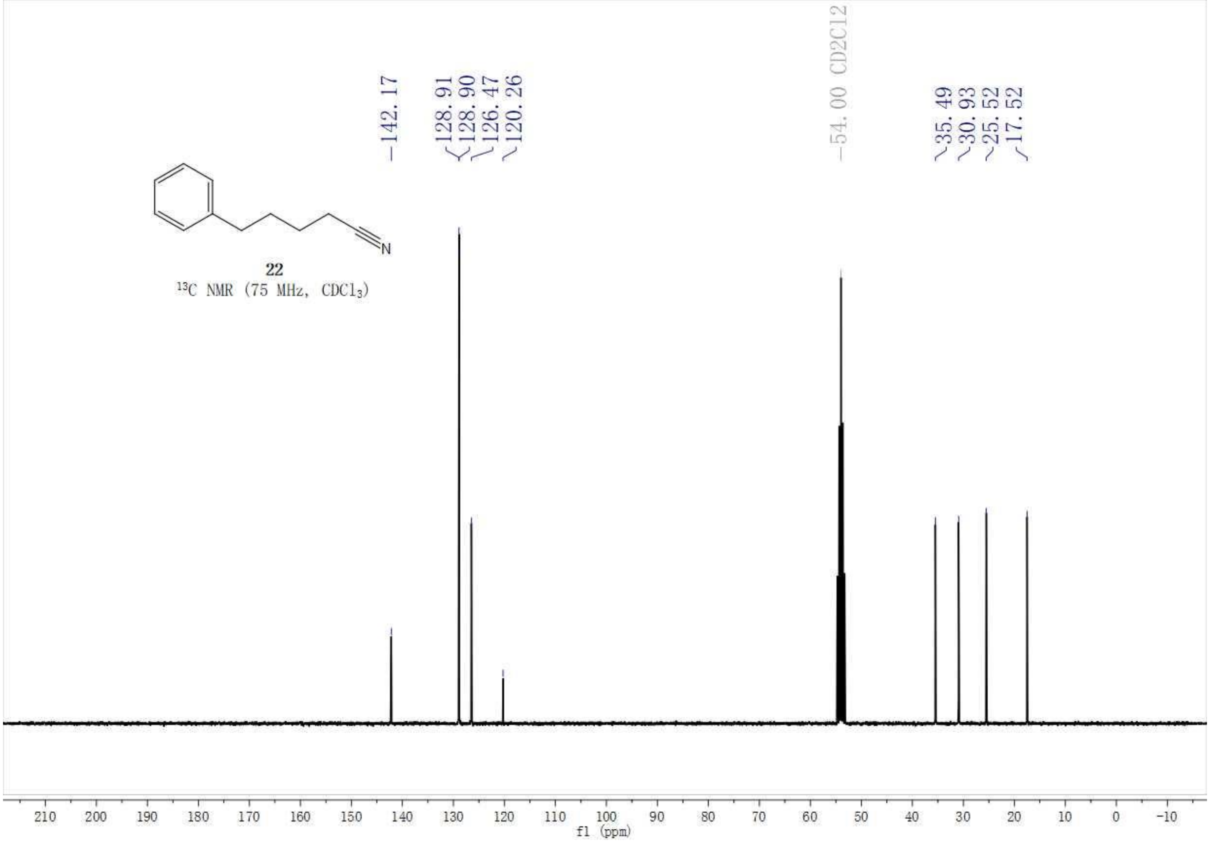
142.17
128.91
128.90
126.47
120.26
54.00 CD2Cl2
35.49
30.93
25.52
17.52
N
22
13C NMR (75 MHz, CDCl3)
f1 (ppm)

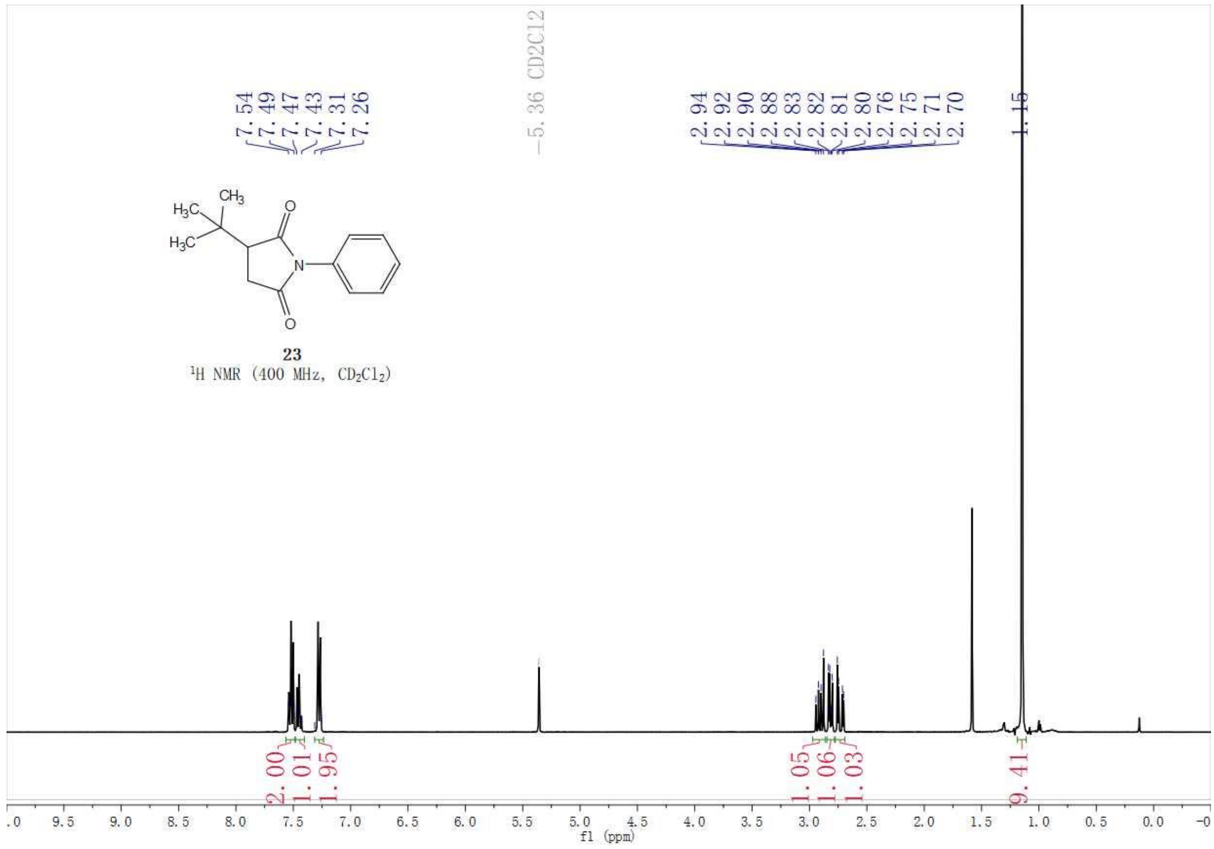
7.54
7.49
7.47
7.43
7.31
7.26
5.36 CD2Cl2
2.94
2.92
2.90
2.88
2.83
2.82
2.81
2.80
2.76
2.75
2.71
2.70
1.15
23
¹H NMR (400 MHz, CD₂Cl₂)
2.00
1.01
1.95
1.05
1.06
1.03
9.41
fl (ppm)

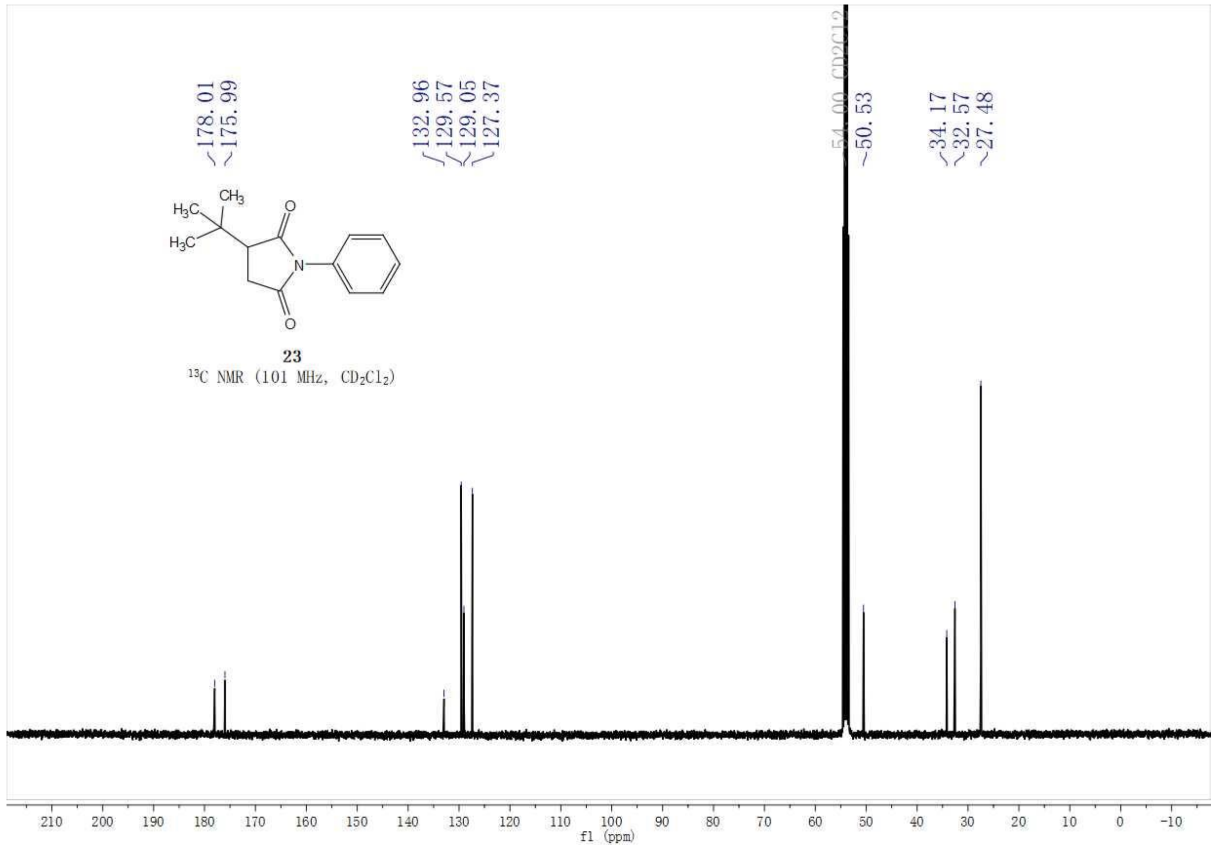
178.01
175.99
132.96
129.57
129.05
127.37
54.00 CD2Cl2
50.53
34.17
32.57
27.48
23
¹³C NMR (101 MHz, CD₂Cl₂)
fl (ppm)

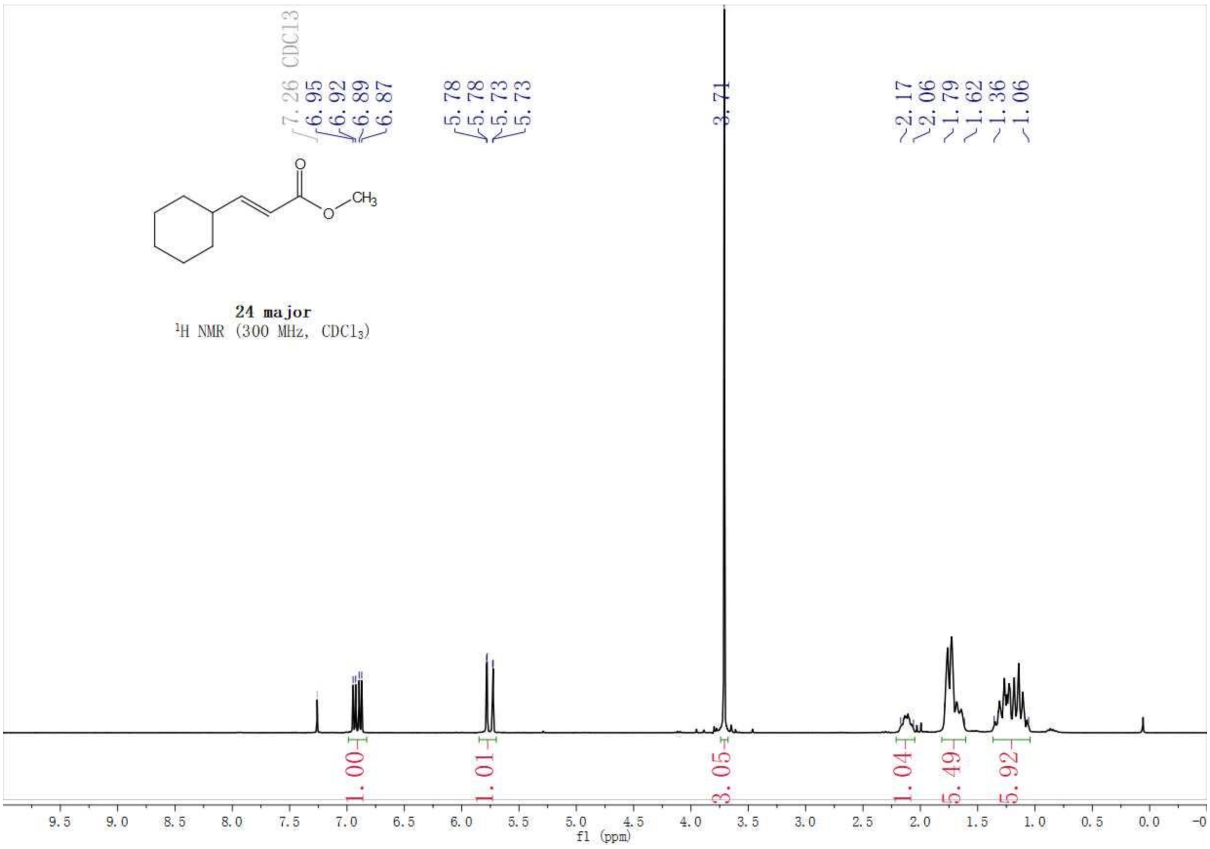
24 major
1H NMR (300 MHz, CDCl3)
7.26 CDCl3
6.95
6.92
6.89
6.87
5.78
5.78
5.73
5.73
3.71
2.17
2.06
1.79
1.62
1.36
1.06
1.00
1.01
3.05
1.04
5.49
5.92
f1 (ppm)

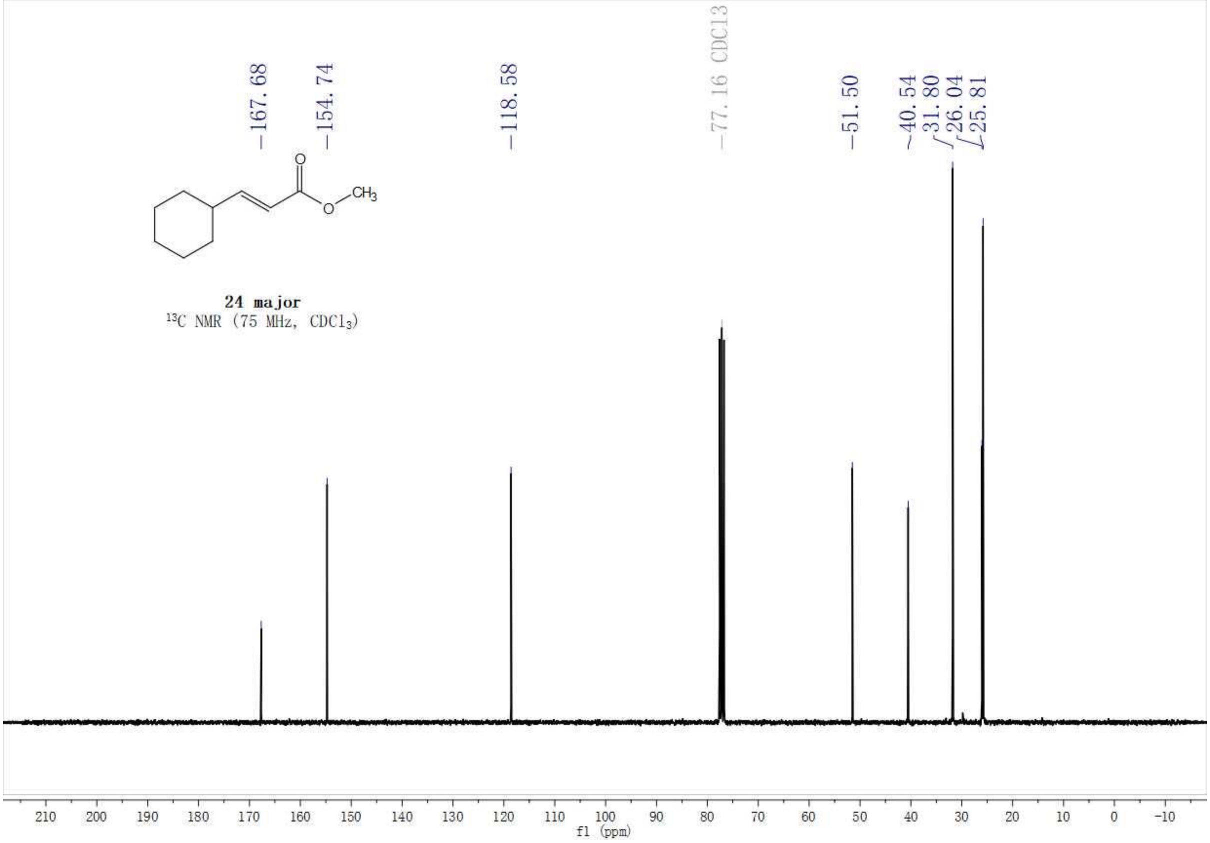
24 major
13C NMR (75 MHz, CDCl3)
167.68
154.74
118.58
77.16 CDCl3
51.50
40.54
31.80
26.04
25.81
f1 (ppm)

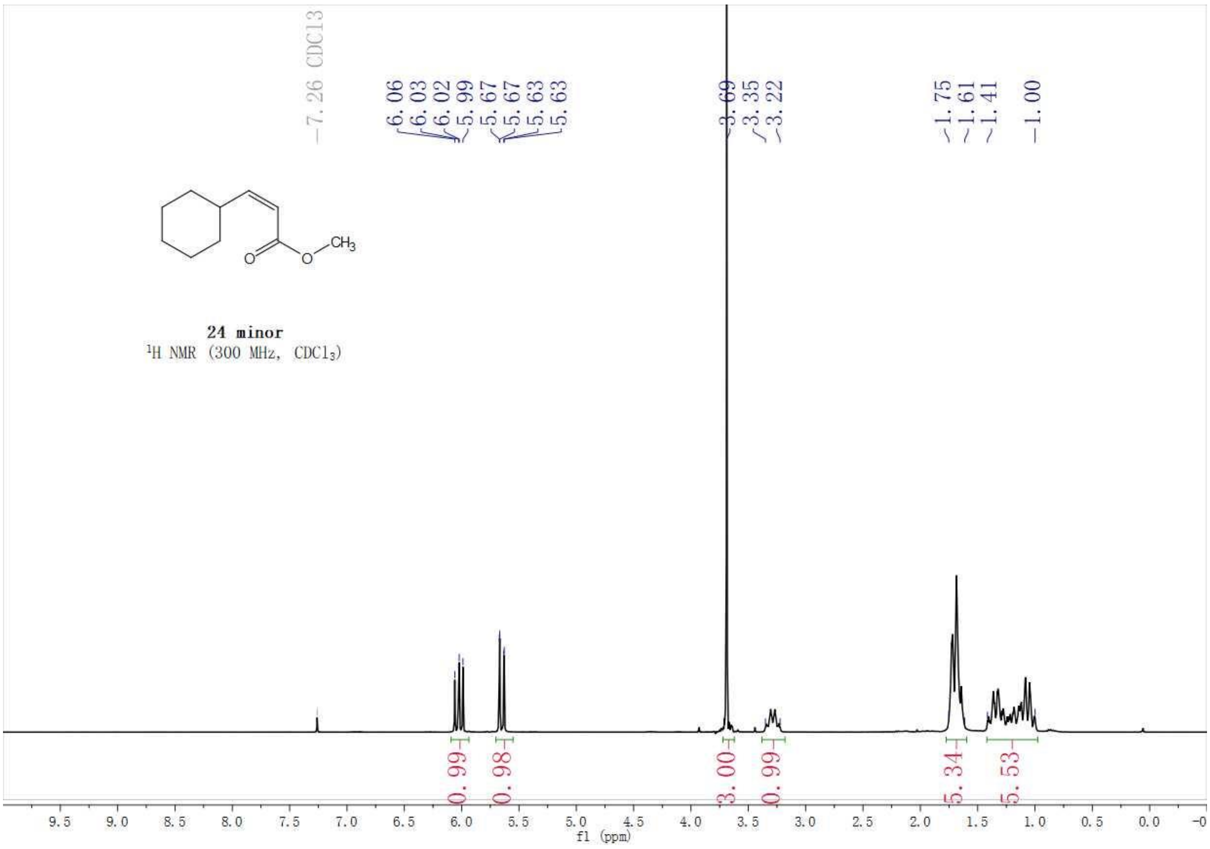
7.26 CDCl3
6.06
6.03
6.02
5.99
5.67
5.67
5.63
5.63
3.69
3.35
3.22
1.75
1.61
1.41
1.00
24 minor
1H NMR (300 MHz, CDCl3)
0.99
0.98
3.00
0.99
5.34
5.53
9.5
9.0
8.5
8.0
7.5
7.0
6.5
6.0
5.5
5.0
4.5
4.0
3.5
3.0
2.5
2.0
1.5
1.0
0.5
0.0
f1 (ppm)

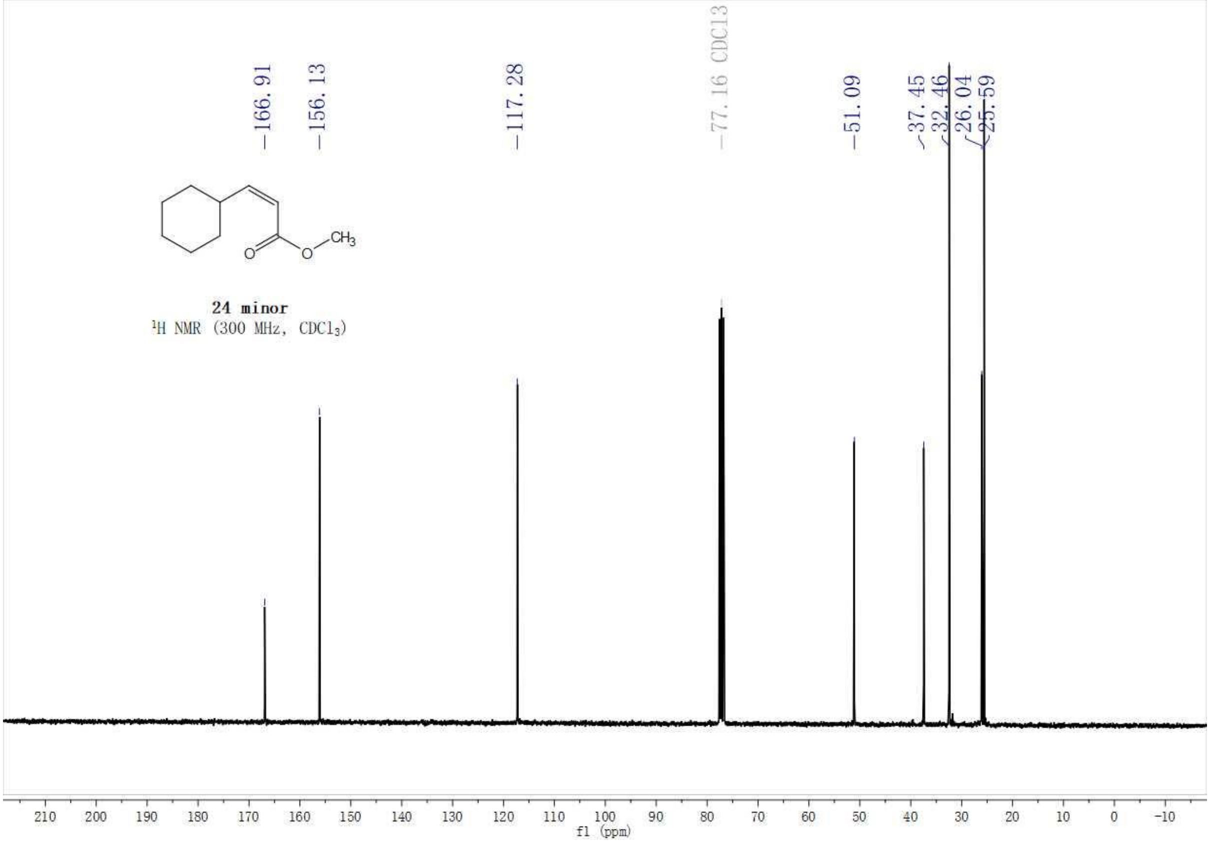
166.91
156.13
117.28
77.16 CDCl3
51.09
37.45
32.46
26.04
25.59
24 minor
1H NMR (300 MHz, CDCl3)
210
200
190
180
170
160
150
140
130
120
110
100
90
80
70
60
50
40
30
20
10
0
-10
f1 (ppm)
